# Deciphering a Novel Necroptosis-Related miRNA Signature for Predicting the Prognosis of Clear Cell Renal Carcinoma

**DOI:** 10.1155/2022/2721005

**Published:** 2022-04-25

**Authors:** Jia-hao Bao, Jiang-bo Li, Han-sen Lin, Wen-jin Zhang, Bing-yan Guo, Jun-jie Li, Liang-min Fu, Yang-peng Sun

**Affiliations:** ^1^Hospital of Stomatology, Guanghua School of Stomatology, Sun Yat-sen University, Guangdong Provincial Key Laboratory of Stomatology, Guangzhou, China; ^2^Department of Urology, The First Affiliated Hospital of Sun Yat-sen University, Guangzhou, China; ^3^Institute of Precision Medicine, The First Affiliated Hospital of Sun Yat-sen University, Guangzhou, China

## Abstract

Clear cell renal cell carcinoma (ccRCC) is the most common histological and devastating subtype of renal cell carcinoma. Necroptosis is a form of programmed cell death that causes prominent inflammatory responses. miRNAs play a significant role in cancer progression through necroptosis. However, the prognostic value of necroptosis-related miRNAs remains ambiguous. In this study, 39 necroptosis-related miRNAs (NRMs) were extracted and 17 differentially expressed NRMs between normal and tumor samples were identified using data form The Cancer Genome Atlas (TCGA). After applying univariate Cox proportional hazard regression analysis and LASSO Cox regression model, six necroptosis-related miRNA signatures were identified in the training cohort and their expression levels were verified by qRT-PCR. Using the expression levels of these miRNAs, all patients were divided into the high- and low-risk groups. Patients in the high-risk group showed poor overall survival (*P* < 0.0001). Time-dependent ROC curves confirmed the good performance of our signature. The results were verified in the testing cohort and the entire TCGA cohort. Univariate and multivariate Cox regression models demonstrated that the risk score was an independent prognostic factor. Additionally, a predictive nomogram with good performance was constructed to enhance the implementation of the constructed signature in a clinical setting. We then employed miRBD, miRTarBase, and TargetScan to predict the target genes of six necroptosis-related miRNAs. Gene ontology and Kyoto Encyclopedia of Genes and Genomes analyses indicated that 392 potential target genes were enriched in cell proliferation-related biological processes. Six miRNAs and 59 differentially expressed target genes were used to construct an miRNA–mRNA interaction network, and 11 hub genes were selected for survival and tumor infiltration analysis. Drug sensitivity analysis revealed potential drugs that may contribute to cancer management. Hence, necroptosis-related genes play an important role in cancer biology. We developed, for the first time, a necroptosis-related miRNA signature to predict ccRCC prognosis.

## 1. Introduction

Renal cancer is the leading cause of cancer-related deaths worldwide. In 2019 in the United States, 73,820 people were diagnosed with renal cancer, among which 14,770 patients died as a result [1]. Renal cell carcinoma (RCC) accounts for approximately 85% of renal cancers. The most common histological subtype of RCC is clear cell RCC (ccRCC), accounting for 75–80% of all RCC patients [2]. The 5-year survival rate of patients with ccRCC diagnosed early when the tumor is localized is >90%, whereas it decreases to 12% in patients with distant metastasis [3]. Currently, surgical resection combined with adjuvant systemic therapy is the primary treatment for patients with ccRCC [4, 5]. Although remarkable progress has been made in the management of ccRCC in the last decade, approximately 20%–30% of patients with ccRCC present initially with cancer metastasis, and an additional 20% will present it after radical surgical resection, which is associated with poor prognosis [6]. Therefore, it is necessary to identify reliable prognostic biomarkers to guide clinicians when choosing the optimal treatment.

Necroptosis, once considered merely as an accidental uncontrolled form of cell death, is now recognized as a form of programmed cell death, which is regulated by a set of molecular mechanisms [7, 8]. Unlike apoptosis, necroptosis causes prominent inflammatory responses and triggers adaptive immunity [9]. Necroptosis is initiated by death receptors, such as tumor necrosis factor receptor 1, and depends on the activation of receptor-interacting protein kinase 1 (RIPK1) and protein mixed lineage kinase domain-like (MLKL) [7, 10]. Accumulating evidence suggests that necroptosis is a pivotal process in tumorigenesis, cancer progression, and metastasis [11]. However, the results are controversial, as evidence supports both anti- and prometastasis roles of necroptosis [11]. Hence, the prognostic value of necroptosis in ccRCC remains unclear and should be elucidated.

MicroRNAs (miRNAs) are small single-stranded noncoding RNAs that negatively regulate gene expression by binding to the target gene 3′-untranslated region. An increasing number of studies indicate that miRNAs are involved in numerous biological processes in cancer, including tumor occurrence, development, and prognosis [12]. Additionally, several miRNAs play a significant role in regulating necroptosis in cancer by targeting various components of the involved signaling pathways [13]. For example, miR-874 induces necroptosis in colorectal cancer by targeting caspase-8 [14, 15]. miR-210 promotes breast cancer metastasis by targeting E-cadherin [16]. It was also demonstrated that miR-381-3p inhibits RIPK3 and MLKL in patients with RCC, thereby inhibiting necroptosis and potentially leading to a poor prognosis [17]. The prognostic value of necroptosis-related miRNAs in ccRCC has not yet been investigated, and no molecular signature-related necroptosis has been established to date.

In the current study, we first performed an integrated analysis of the expression levels of necroptosis-related miRNAs in The Cancer Genome Atlas (TCGA) cohort. We established a novel necroptosis-related miRNA (NRM) prognostic risk signature to predict the prognosis of patients with ccRCC and contribute to the management of patients. Additionally, an applicable nomogram was constructed to apply our signature better. We also explored the target genes of these miRNAs and provided a new understanding of their role in ccRCC.

## 2. Methods

### 2.1. Collection of Data

miRNA expression profiles, recorded as reads per million mapped values, and the corresponding clinical follow-up information of 545 patients with ccRCC and 71 healthy controls were downloaded from TGCA using the GDC Data Portal (https://portal.gdc.cancer.gov/). In total, 2435 miRNAs were identified. The mRNA expression profiles of HTSeq-fragments per kilobase per million and clinical information of 539 patients with ccRCC and 72 healthy controls were obtained and converted into transcripts per million reads for subsequent bioinformatics analysis. Moreover, somatic mutation data and copy number variation (CNV) data for ccRCC were acquired from TCGA database. Data analysis was conducted using R software (version 4.1.1). The clinicopathological characteristics of the cohorts of patients with ccRCC, including sex, age, grade, stage, TMN stage, overall survival (OS), disease-specific survival, and progression-free interval, are shown in Supplementary Table 1.

### 2.2. Identification of Overall Survival-Related Differentially Expressed NRMs (DE-NRMs)

Consulting a previous literature, we identified 39 NRMs (Supplementary Table 2) [13, 15, 18]. After matching the expression levels of the aforementioned miRNAs, the remaining miRNAs were retained for a further study. DE-NRMs between tumor and normal tissues were identified using the R package “Limma” [19]. The significance threshold was defined at a false discovery rate (FDR) < 0.05 to avoid overscreening. Then, univariate Cox proportional hazard regression analysis was conducted using the R packages “survival” and “forest” with the significance threshold set to FDR < 0.05. Subsequently, the R package “caret” was applied to divide the samples into training and testing cohorts.

### 2.3. Development and Validation of Necroptosis-Related miRNA Signature

The “glmnet” R package was used to perform the least absolute shrinkage and selection operator (LASSO) Cox regression analysis in the training cohort using the penalty parameter *λ* and 10-fold cross-validation [20]. The miRNA signature was established based on the results of LASSO Cox regression analysis. Then, selected miRNAs were fit in multivariate Cox proportional hazard regression analysis using the R package “survival.” The risk score of each patient was computed using the formula: Risk_score = ∑_i=1_^*n*^Coef_*i*_ × Exp_*i*_, in which Coef refers to the coefficient and Exp refers to the expression level of the selected miRNAs. Patients were separated into the high- and low-risk groups based on the median value of the risk score. Principal component analysis (PCA) was applied to explore the distribution of patients in the training cohort, the test cohort, and the whole TCGA cohort using the function “prcomp” of the “stats” R package. Kaplan–Meier (K-M) analysis was conducted using the R packages “survival” and “survminer.” The “timeROC” R package was applied to perform 1-, 3-, and 5-year receiver operating characteristic (ROC) analyses.

### 2.4. Independent Prognostic Significance and Clinical Subgroup Analysis of NRM Signature

Univariate and multivariable Cox regression analyses were used to explore whether the NRM signature could be an independent prognostic factor. We extracted clinical information, including age, sex, laterality, histological grade, and pathologic stage, and analyzed them together. The R package “survival” was used for analyses, and “forest” was used for visualization. Dataset stratification analysis were performed according to age, pathological stage, and histological grade in order to explore the prognostic value of risk score signature in clinical subgroups.

### 2.5. Establishment and Evaluation of the Predictive Nomogram

A predictive nomogram was established using the R package “rms,” and its prognostic accuracy was assessed using ROC analysis. Additionally, the discrimination ability of the predictive nomogram was evaluated using the R packages “rms” and “survival.”

### 2.6. Exploration and Functional Analysis of Target Genes of miRNAs

Target genes of six miRNAs, namely, hsa-miR-101-3p, hsa-miR-193a-3p, hsa-miR-200a-5p, hsa-miR-214-3p, hsa-miR-221-3p, and hsa-miR-223-3p, were identified using miRDB (http://www.mirdb.org/miRDB/) [21], TargetScan (http://www.targetscan.org/) [22], and miRTarBase (https://mirtarbase.cuhk.edu.cn/~miRTarBase/miRTarBase_2019/php/index.php) [23]. The R package “VennDiagram” was used to screen and plot potential target genes that were present in the three prediction databases. The Bioconductor package ClusterProfiler R package (v4.1.4) was used to perform gene ontology (GO) and Kyoto Encyclopedia of Genes and Genomes (KEGG) enrichment analysis [24].

### 2.7. Construction of miRNA–mRNA Interaction Network

The “Limma” package was used to identify differentially expressed target genes between tumor and normal samples [19]. FDR < 0.05 and |log_2_FoldChange| > 1 were set as the cutoff values. The interaction network between the selected miRNAs and differentially expressed target genes was visualized using Cytoscape software (version 3.7.2) [25].

### 2.8. Identification and Tumor Infiltration and Drug Sensitivity Analysis of Hub Genes

The Cytoscape plug-in Cytohubba was applied to identify hub genes within the target genes [26]. The top 20 hub genes were ranked using maximal clique centrality (MCC), edge percolated component (EPC), and connection degree. The hub genes simultaneously identified by the three methods were verified by LASSO Cox regression with 10-fold cross-validation. The R package “survival” was used to perform survival analysis of the hub genes. The web server TIMER was employed to detect the tumor immune infiltration status of the hub genes [27]. The drug sensitivity analysis was conducted using the Gene Set Cancer Analysis (GSCA) database, which contains the drug information from Genomics of Drug Sensitivity in Cancer (GDSC) and The Cancer Therapeutics Response Portal (CTRP) [28].

### 2.9. RNA Extraction and Real-Time PCR

Total RNA from the tumor and adjacent normal samples was extracted form 12 KIRC patients using the Universal microRNA Purification Kit (EZB-miRN1) (EZBioscience, Shanghai, China) according to the manufacturer's instructions. We also collected the total RNA from the renal epithelial cell line (HK2) and 6 human renal cancer cell lines (786O, 769P, ACHN, CAKI-1, and A498) using Universal microRNA Purification Kit (EZB-miRN1) (EZBioscience, Shanghai, China). The expression of the signature-related miRNAs was further examined by qRT-PCR. The complementary DNA (cDNA) was synthesized with miRNA 1st strand cDNA synthesis kit (Accurate Biology, Changsha, China). The qRT-PCR was performed on Applied Biosystems™ QuantStudio™ 5 Real-Time PCR System using 2×SYBR Green qPCR Master Mix (ROX2 plus) (A0012-R2) (EZBioscience, Shanghai, China). U6 was introduced as an internal control small RNA to normalize miRNA levels. Expression levels of each miRNA were calculated using the 2-*ΔΔ*Ct method. All trials were conducted in triplicate. All specific primers used in the study are listed in Supplementary Table 3. The studies involving human participants were reviewed and approved by the Institutional Ethics Committee for Clinical Research and Animal Trials of the First Affiliated Hospital of Sun Yat-sen University [(2021)144].

### 2.10. Statistical Analysis

All statistical analyses were conducted using R software (v4.1.1) and GraphPad Prism (v9.0.0). The Wilcoxon rank-sum test was used to compare expression levels between normal and tumor samples. For the comparison of expression levels among cell lines, the one-way analysis of variance (ANOVA) was employed. K-M analysis and the log-rank test were used to compare the OS time. Univariate and multivariate Cox regression models were used to explore the independent prognostic value of the NRM signature. Statistical significance was set at *P* < 0.05.

## 3. Results

### 3.1. Identification of OS-Related DE-NRMs

**Figure 1:**
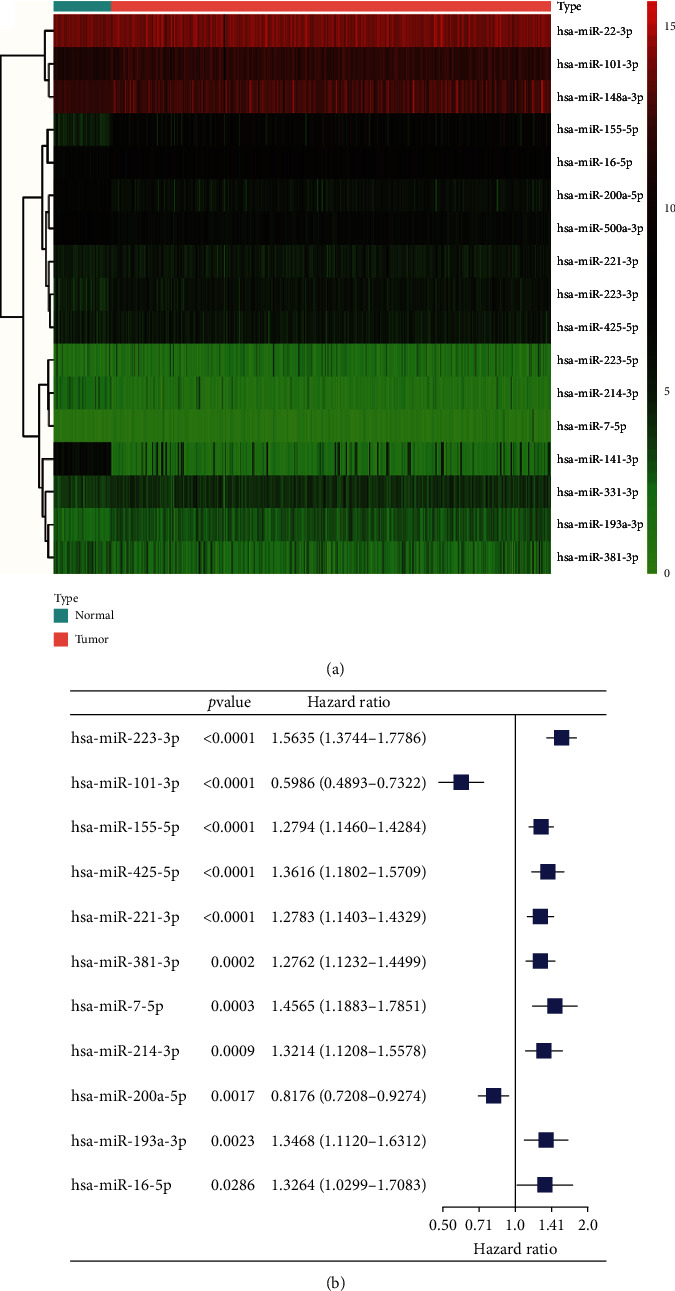
Identification of overall survival- (OS-) related differentially expressed necroptosis-related miRNAs (NRMs). (a) Heat map showing expression levels of differentially expressed NRMs. Green: low expression level; red: high expression level. (b) Forest plot of univariate Cox proportional hazard regression analysis revealed 11 OS-related differentially expressed necroptosis-related miRNAs.

Analysis of the expression levels of NRMs identified 20 DE-NRMs between normal and tumor samples, of which 17 met the statistical criteria of FDR < 0.05. These included 12 upregulated miRNAs (hsa-miR-155-5p, hsa-miR-7-5p, hsa-miR-193a-3p, hsa-miR-223-3p, hsa-miR-223-5p, hsa-miR-16-5p, hsa-miR-331-3p, hsa-miR-221-3p, hsa-miR-101-3p, hsa-miR-425-5p, hsa-miR-148a-3p, and hsa-miR-22-3p) and five downregulated miRNAs (hsa-miR-500a-3p, hsa-miR-200a-5p, hsa-miR-381-3p, hsa-miR-214-3p, and hsa-miR-141-3p) in tumor samples compared with normal samples (Figure 1(a)). Univariate Cox proportional hazard regression analysis with a threshold of adjusted *P* value < 0.05 was used to identify the DE-NRMs associated with OS. Eleven OS-related DE-NRMs were retained and depicted using a forest plot (Figure 1(b)). These DE-NRMs included hsa-miR-223-3p (HR = 1.5635, 95%CI = 1.3744–1.7786, *P* < 0.0001), hsa-miR-101-3p (HR = 0.5986, 95%CI = 0.4893–0.7322, *P* < 0.0001), hsa-miR-155-5p (HR =1.2794, 95% CI =1.1460-1.4284, *P* < 0.0001), hsa-miR-425-5p (HR = 1.3616, 95%CI = 1.1802-1.5709, *P* < 0.0001), hsa-miR-221-3p (HR = 1.2783, 95%CI = 1.1403-1.4329, *P* < 0.0001), hsa-miR-381-3p (HR = 1.2762, 95%CI = 1.1232-1.4499, *P* = 0.0002), hsa-miR-7-5p (HR = 1.4565, 95%CI = 1.1883-1.7851, *P* = 0.0003), hsa-miR-214-3p (HR = 1.3214, 95%CI = 1.1208-1.5578, *P* = 0.0009), hsa-miR-200a-5p (HR = 0.8176, 95%CI = 0.7208-0.9274, *P* = 0.0017), hsa-miR-193a-3p (HR = 1.3468, 95%CI = 1.1120-1.6312, *P* = 0.0023), and hsa-miR-16-5p (HR = 1.3264, 95%CI = 1.0299-1.7083, *P* = 0.0286).

### 3.2. Development and Validation of Necroptosis-Related miRNA Signature

**Figure 2:**
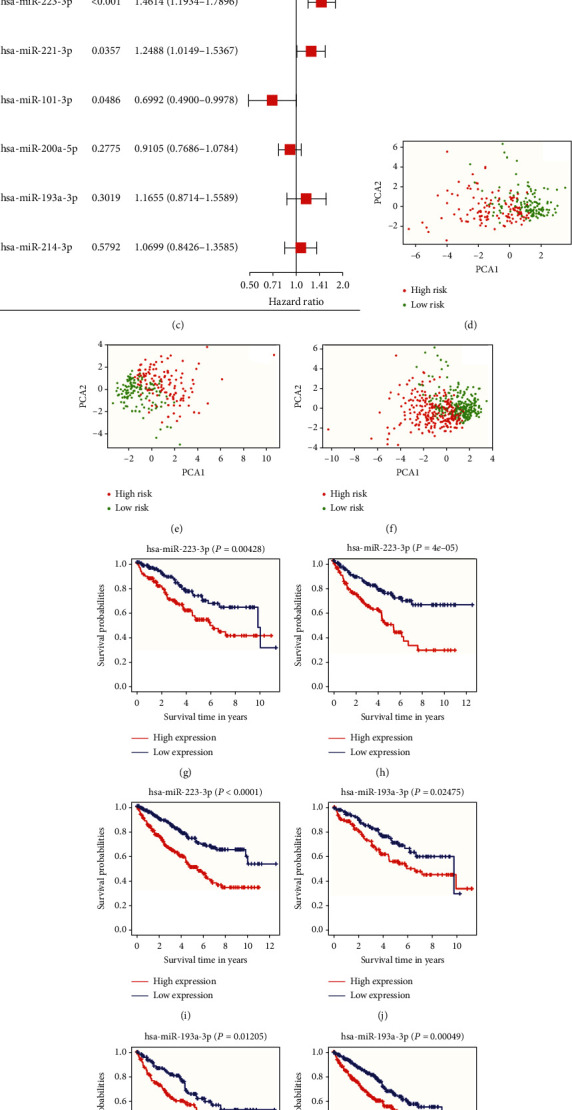
Development of necroptosis-related miRNA (NRM) signature in TCGA training cohort. (a) LASSO Cox regression analysis of the 11 overall survival- (OS-) related NRMs. (b) Plot of 10-fold cross-validation error rates in the LASSO Cox regression analysis. (c) Hazard ratio of the six mRNAs identified. The principal component analysis (PCA) plot of the distribution between the high- and low-risk groups in (d) the training cohort, (e) the testing cohort, and (f) the whole TGCA cohort. Kaplan-Meier plot of has-miR-223-3p in (g) the training cohort, (h) the testing cohort, and (i) the whole TCGA cohort. Kaplan-Meier plot of has-miR-193-3p in (j) the training cohort, (k) the testing cohort, and (l) the whole TCGA cohort.

Patients from TCGA for whom data on miRNA expression levels and clinical information were available were randomly partitioned into training and test cohorts at a ratio of 0.5. The LASSO Cox regression analysis was performed in the training cohort using 10-fold cross-validation. Among the 11 OS-related NRMs, six miRNAs (hsa-miR-101-3p, hsa-miR-193a-3p, hsa-miR-200a-5p, hsa-miR-214-3p, hsa-miR-221-3p, and hsa-miR-223-3p) were associated with OS (Figures 2(a) and 2(b)). Multivariate Cox regression analysis was performed to calculate the coefficient of each miRNA. hsa-miR-223-3p (HR = 1.4614, 95%CI = 1.1934-1.7896, *P* < 0.001), hsa-miR-221-3p (HR = 1.2488, 95%CI = 1.0149-1.5367, *P* = 0.0357), and hsa-miR-101-3p (HR = 0.6992, 95%CI = 0.4900-0.9978, *P* = 0.486) were independent prognostic factors (Figure 2(c)). The NRM signature was developed based on the LASSO Cox regression and multivariate Cox regression analyses, and the risk score was calculated according to the following formula: risk score = (0.15316∗expression level of hsa‐miR‐193a‐3p) − (0.35780∗expression level of hsa‐miR‐101‐3p) − (0.09381∗expression level of hsa‐miR‐200a − 5p) + (0.06757∗expression level of hsa‐miR‐214‐3p) + (0.22222∗expression level of hsa‐miR‐221‐3p) + (0.37938∗expression level of hsa‐miR‐223‐3p). Subsequently, we computed the risk score of each sample and classified patients into the high- and low-risk groups based on the median risk score value in the training, testing, and whole TCGA cohorts. Then, to detect the distribution of samples in the two groups, we conducted PCA in the three cohorts and found a distinct distribution between the high- and low-risk groups (Figures 2(d)–2(f)). Additionally, K-M analysis in three cohorts revealed that hsa-miR-193a-3p and hsa-miR-223-3p were correlated with poor prognosis in patients with ccRCC. Moreover, K-M indicated that high expression levels of hsa-miR-193a-3p and hsa-miR-223-3p were associated with a shorter lifetime (Figures 2(g)–2(l)).

**Figure 3:**
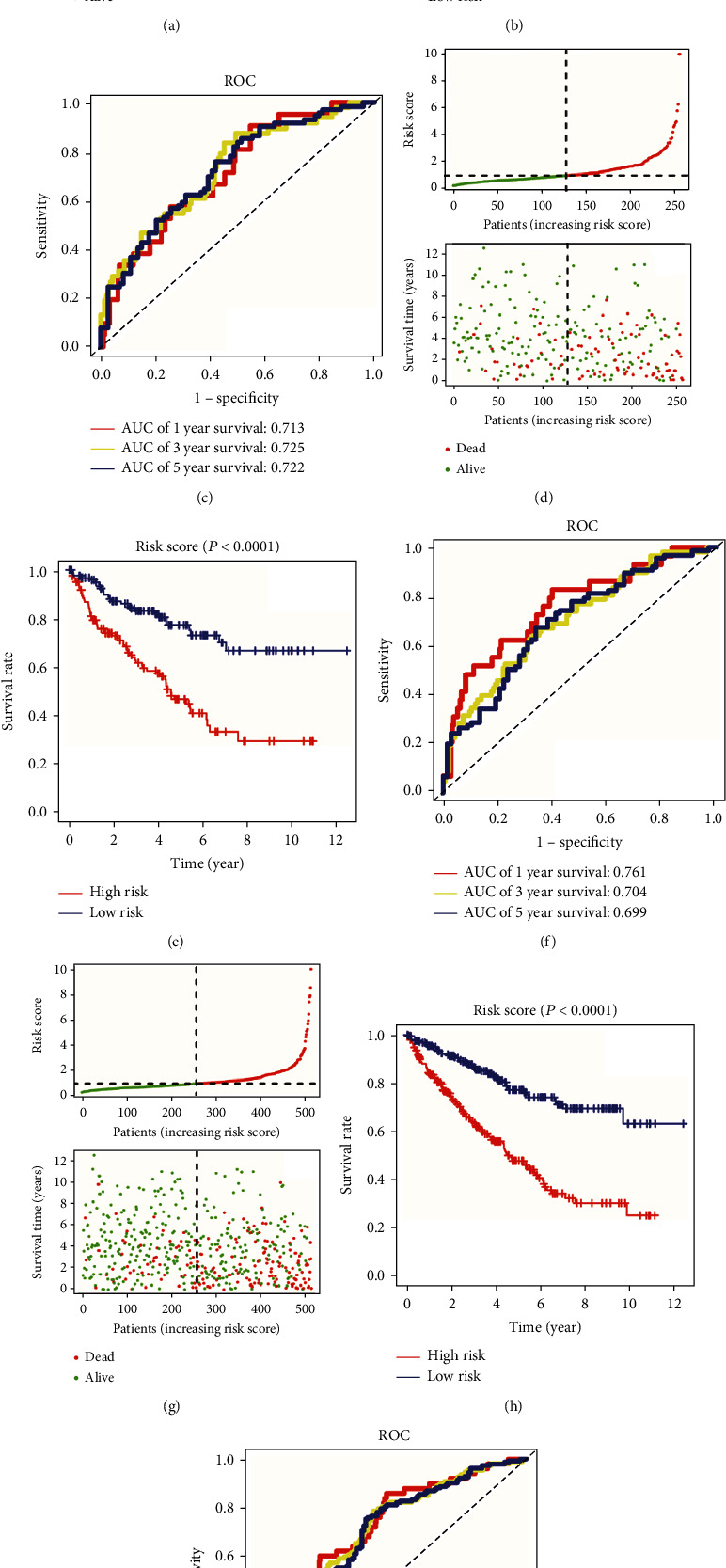
Prognostic analysis of necroptosis-related miRNA (NRM) signature in training, testing, and whole TCGA cohorts. (a) Risk line and risk point plots show the survival rate and risk score of each patient in the training cohort. (b) Kaplan–Meier plot of patients in the high- and low-risk groups in the training cohort. (c) ROC curve shows the prognostic performance of the NRM signature in the training cohort. (d) Risk line and risk point plots show the survival rate and risk score of each patient in the testing cohort. (e) Kaplan–Meier plot of patients in the high- and low-risk groups in the testing cohort. (f) ROC curve shows the prognostic performance of the NRM signature in the testing cohort. (g) Risk line and risk point plots show the survival rate and risk score of each patient in the entire TCGA cohort. (h) Kaplan–Meier plot of patients in the high- and low-risk groups in the entire TCGA cohort. (i) The ROC curve shows the prognostic performance of the NRM signature in the entire TCGA cohort.

We then investigated the prognostic value of our risk signature in the training, testing, and whole TCGA cohorts. The risk-line plot and risk-point plot showed that patients with a high-risk score were associated with a higher probability of death and shorter survival time in the training cohort (Figure 3(a)). Additionally, the K-M analysis indicated that high-risk patients with ccRCC had a poor prognosis (*P* < 0.0001, Figure 3(b)). Time-dependent ROC analysis conducted in the training cohort indicated a good efficiency of the NRM signature, with an area under curve (AUC) value of 0.713 for predicting 1-year survival, 0.725 for predicting 3-year survival, and 0.722 for predicting 5-year survival (Figure 3(c)). The results in the test cohort (Figure 3(d)) and the entire TCGA cohort (Figure 3(g)) were consistent. Additionally, K-M analysis confirmed that patients classified into the high-risk group had a lower survival rate than those in the low-risk group, in both the testing (*P* < 0.0001, Figure 3(e)) and whole TCGA (*P* < 0.0001, Figure 3(h)) cohorts. The AUC value in the testing cohort was 0.761 for predicting 1-year survival, 0.704 for predicting 3-year survival, and 0.699 for predicting 5-year survival (Figure 3(f)). Consistent with individual analyses, the AUC value in the entire TCGA cohort was 0.721 for predicting 1-year survival, 0.711 for predicting 3-year survival, and 0.703 for predicting 5-year survival (Figure 3(f)).

### 3.3. Independent Prognostic Significance and Clinical Subgroup Analysis of NRM Signature

**Figure 4:**
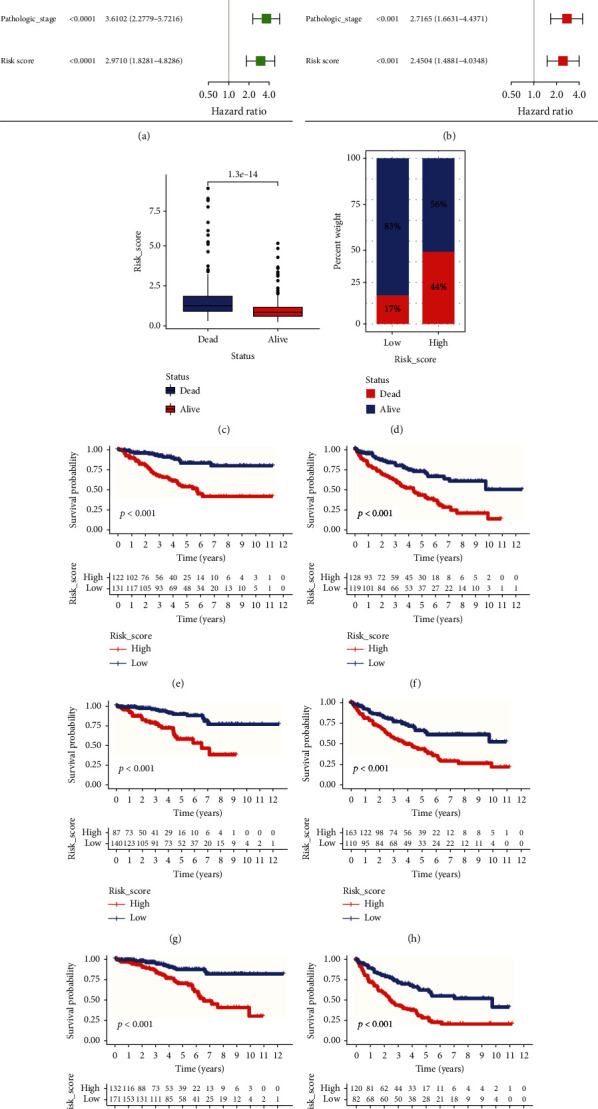
Independent prognostic significance and clinical subgroup analysis of clear cell renal cell carcinoma (ccRCC) signature. (a) Forest plot of the results of the univariate Cox regression analysis of overall survival for risk score and clinical characteristics in the training cohort. (b) Forest plot of the results of the multivariate Cox regression analysis of overall survival for risk score and clinical characteristics in the training cohort. (c) Risk score levels in the dead and alive groups. (d) Percentage of patients' status in the low- and high-risk groups. Kaplan–Meier plot of patients with different clinical features: (e) age ≤ 60; (f) age > 60; (g) histological grades 1-2; (h) histological grades 3-4; (i) pathological stages I-II; (j) pathological stages III-IV.

To investigate whether the established NRM signature was an independent prognostic factor for OS, univariate and multivariate Cox regression analyses were conducted in the training cohort. Univariate Cox regression analysis revealed that risk score (HR = 2.9710, 95%CI = 1.8281-4.8266, *P* < 0.0001), pathological stage (HR = 3.6102, 95%CI = 2.2779-5.7216, *P* < 0.0001), histological grade (HR = 2.8547, 95%CI = 1.6703-4.8787, *P* < 0.0001), laterality (HR = 0.5546, 95%CI = 0.3558-0.8644, *P* = 0.0092), and age (HR = 1.8237, 95%CI = 1.1589-2.8700, *P* = 0.0094) were significantly related to OS (Figure 4(a)) and independent prognostic factors. Multivariate Cox regression analysis confirmed that the NRM signature was an independent prognostic factor (HR = 2.4504, 95%CI = 1.4881-4.0348, *P* < 0.001) after correcting for other confounding factors (Figure 4(b)). Additionally, the risk score of NRM signature was an independent factor in the testing and entire TCGA cohorts (Supplementary Figure 1). Moreover, patients in the death group had higher risk scores than those in the alive group, and the percentage of deaths in the high-risk group (44%) was higher than that in the low-risk group (17%, Figures 4(c) and 4(d)).

Subsequently, we used dataset stratification analysis to investigate the independent prognostic value of our risk score signature according to age, histological grade, and pathological stage. In all clinical subgroups divided by age, grade, and stage, patients in the high-risk group had a lower survival probability predicted by K-M analysis (*P* < 0.001). This result suggested that our risk score signature predicts the prognosis of patients with ccRCC who have different clinical characteristics well (Figures 4(e)–4(j)).

### 3.4. Establishment and Evaluation of the Predictive Nomogram in TCGA Cohort

**Figure 5:**
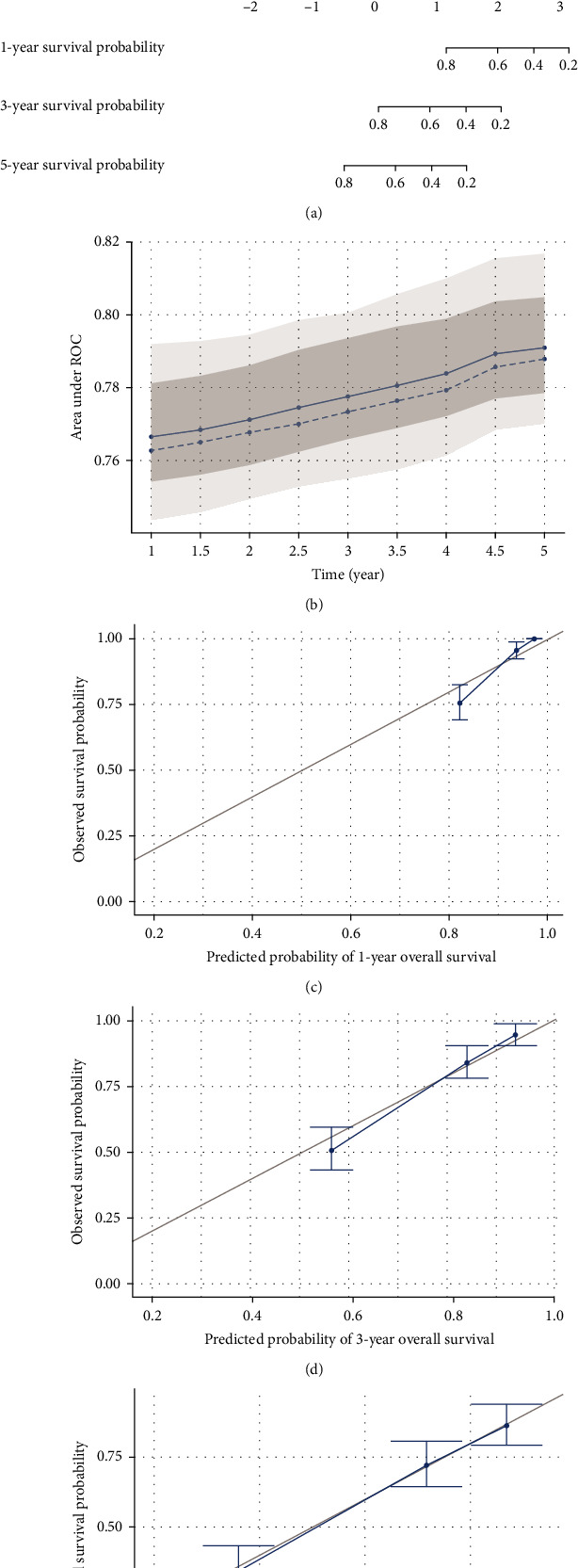
Establishment and assessment of the predictive nomogram. (a) Nomogram to predict the 1-, 3-, and 5-year OS of patients with clear cell renal cell carcinoma (ccRCC). (b) Relationship between the area under the curve (AUC) value and predicted survival time. Calibration curves verified the accuracy of the nomogram for predicting (c) 1-year overall survival, (d) 3-year overall survival, and (e) 5-year overall survival.

To provide a useful model for clinicians and patients, we created a nomogram based on data from the entire TCGA cohort. Combining the risk score of the NRM signature and some easy-to-obtain clinical information, our nomogram can be used to predict the 1-, 3-, and 5-year OS probabilities (Figure 5(a)). The accuracy of the nomogram was assessed using ROC analysis. The AUC value ranged from 0.76 to 0.79 from 1- to 5-year (Figure 5(b)). Moreover, the calibration analysis showed the excellent performance of the predictive nomogram in the entire TCGA cohort (Figures 5(c)–5(e)).

### 3.5. Exploration and Functional Enrichment Analysis of the Target Genes of miRNAs

**Figure 6:**
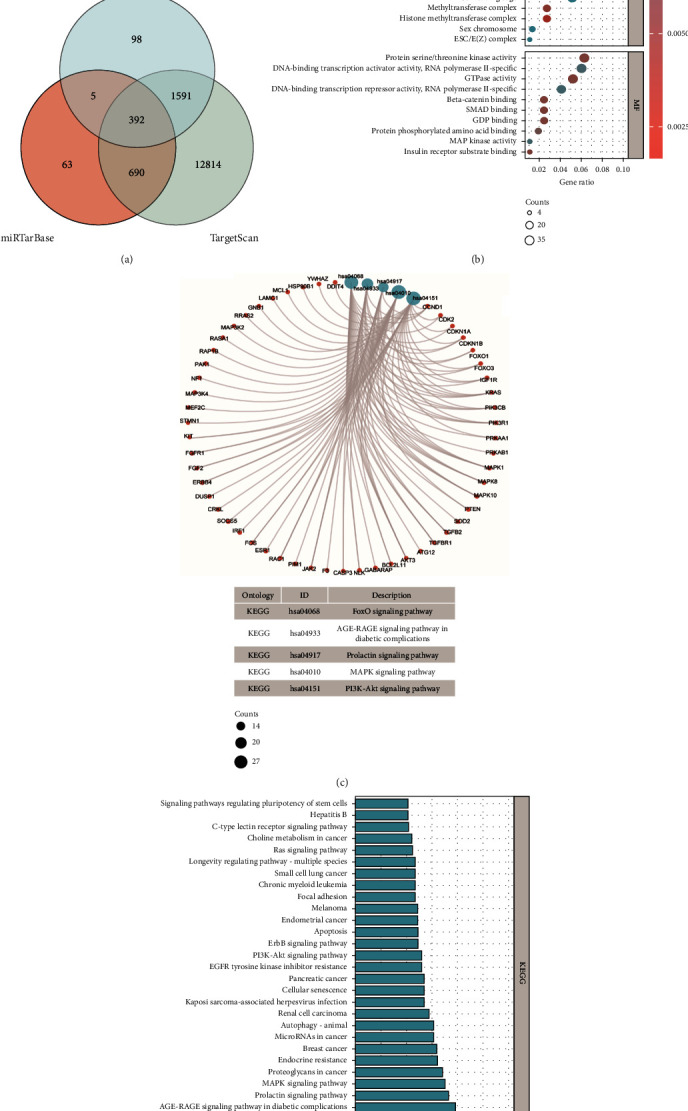
Exploration and functional enrichment analysis of the miRNAs target genes. (a) Venn diagram of target genes for six miRNAs from NRM signature based on miRDB, miRTarBase, and TargetScan. (b) Dot plot showing the results of gene ontology (GO) enrichment analysis of 392 potential target genes differentially expressed between tumor samples and normal samples. (c) Circle network plot showing the relationships between the top five enriched pathways of Kyoto Encyclopedia of Genes and Genomes (KEGG) enrichment analysis and their associated target genes. (d) Bar plot showing the results of KEGG enrichment analysis. Construction of miRNA–mRNA interaction network and identification of hub genes.

To further elucidate the potential functions associated with the NRM signature, we predicted the target genes of the selected miRNAs using three databases, miRDB, miRTarBase, and TargetScan. In total, 2086, 1150, and 15487 target genes were identified in miRDB, miRTarBase, and TargetScan, respectively. Only 392 target genes were identified in all three databases (Figure 6(a) and Supplementary Table 4).

We then conducted GO and KEGG enrichment analyses based on the 392 potential target genes. GO-enrichment analysis of biological function terms showed that the miRNAs' potential target genes were enriched in signal transduction, cell proliferation, and cell response-related biological processes, including Ras protein signal transduction, epithelial cell proliferation, cardiac muscle cell proliferation, cellular response to drugs, response to oxygen levels, response to glucocorticoids, and response to corticosteroids. Cellular component-enriched terms included focal adhesion, cell-substrate adherens junction, cell-substrate junction, methyltransferase complex, cell-cell junction, ESC/E(Z) complex, sex chromosome, and cell leading edge, histone methyltransferase complex, and nuclear chromatin. Finally, molecular function terms enriched included GDP binding, SMAD binding, protein serine/threonine kinase activity, GTPase activity-catenin binding, insulin receptor substrate binding, protein phosphorylated amino acid binding, DNA-binding transcription activator activity RNA polymerase II-specific, MAP kinase activity, and DNA-binding transcription repressor activity RNA polymerase II-specific (Figure 6(b)).

The top five enriched pathways of KEGG enrichment analysis were FoxO signaling pathway, AGE-RAGE signaling pathway in diabetic complications, prolactin signaling pathway, MAPK signaling pathway, and PI3K-Akt signaling pathway. The relationships between the enriched pathways and their target genes are illustrated in Figure 6(c). The top 30 KEGG results are shown in Figure 6(d).

### 3.6. Construction of miRNA–mRNA Interaction Network and Identification of Hub Genes

**Figure 7:**
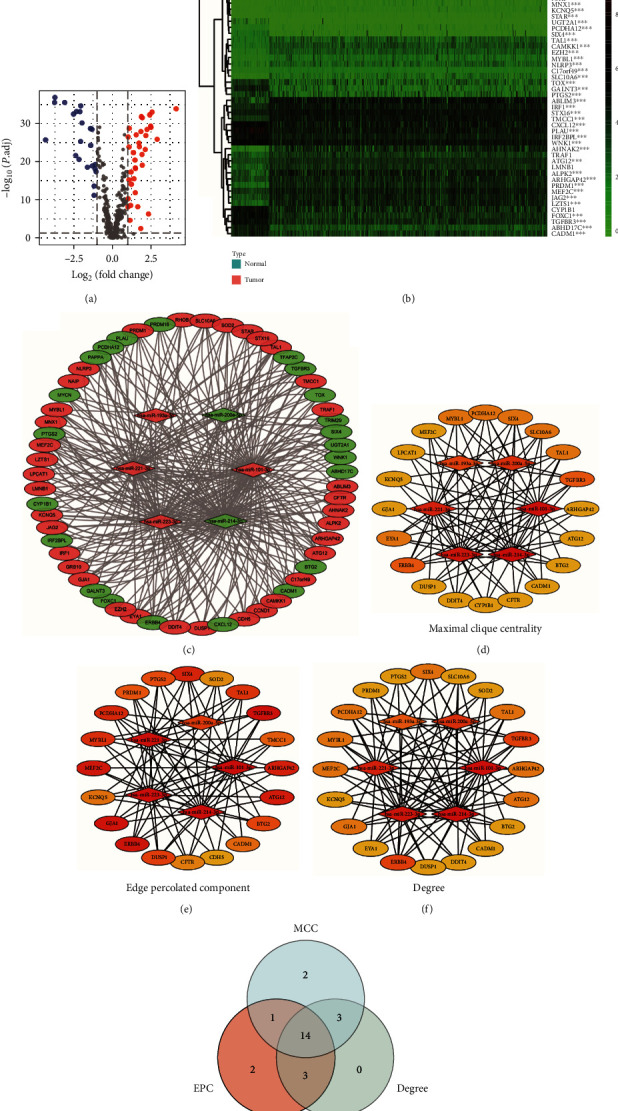
Construction of miRNA-mRNA interaction network and identification of hub genes. (a) Volcano plot showing differentially expressed target genes in clear cell renal cell carcinoma (ccRCC) (red: upregulated; blue: downregulated). (b) Heat map showing the expression profiles of 59 differentially expressed target genes (red: high expression levels; green: low expression levels; ns: *P* ≥ 0.05; ^∗^*P* < 0.05; ^∗∗^*P* < 0.01; ^∗∗∗^*P* < 0.001). (c) Interaction network shows the relationship of six miRNAs and 59 differential expressed target genes (red: upregulated; green: downregulated). (d) Interaction network plotted using the MCC method and Cytohubba. (e) Interaction network plotted using the EPC method and Cytohubba. (f) Interaction network plotted using the degree method and Cytohubba. (g) Venn diagram of hub genes.

To further investigate the functions of the dysregulated miRNAs, we identified their target genes that were differentially expressed. Using the statistical criteria of FDR < 0.05 and |log_2_FC| > 1, we identified 59 differentially expressed target genes (Figures 7(a) and 7(b)). Then, the possible interaction between the six NRM included in the risk signature and the 59 differentially expressed target genes was investigated using Cytoscape, and hub genes were identified using the plugin Cytohubba (Figure 7(c)). Hub genes were ranked using MCC, EPC, and the connection degree algorithms (Figures 7(d)–7(f)). Based on the three algorithms, 14 overlapping genes (ERBB4, TGFBR3, MYBL1, PCDHA12, SIX4, TAL1, ARHGAP42, ATG12, BTG2, CADM1, DUSP1, GJA1, KCNQ5, and MEF2C) were identified and LASSO Cox regression with 10-fold cross-validation was conducted (Supplementary Figure 2). After verification, CADM1, GJA1, and MEF2C were excluded and the remaining 11 genes were regarded as hub genes for the next analysis (Figure 7(g)).

### 3.7. Gene Alterations and Survival Analysis of Hub Genes

**Figure 8:**
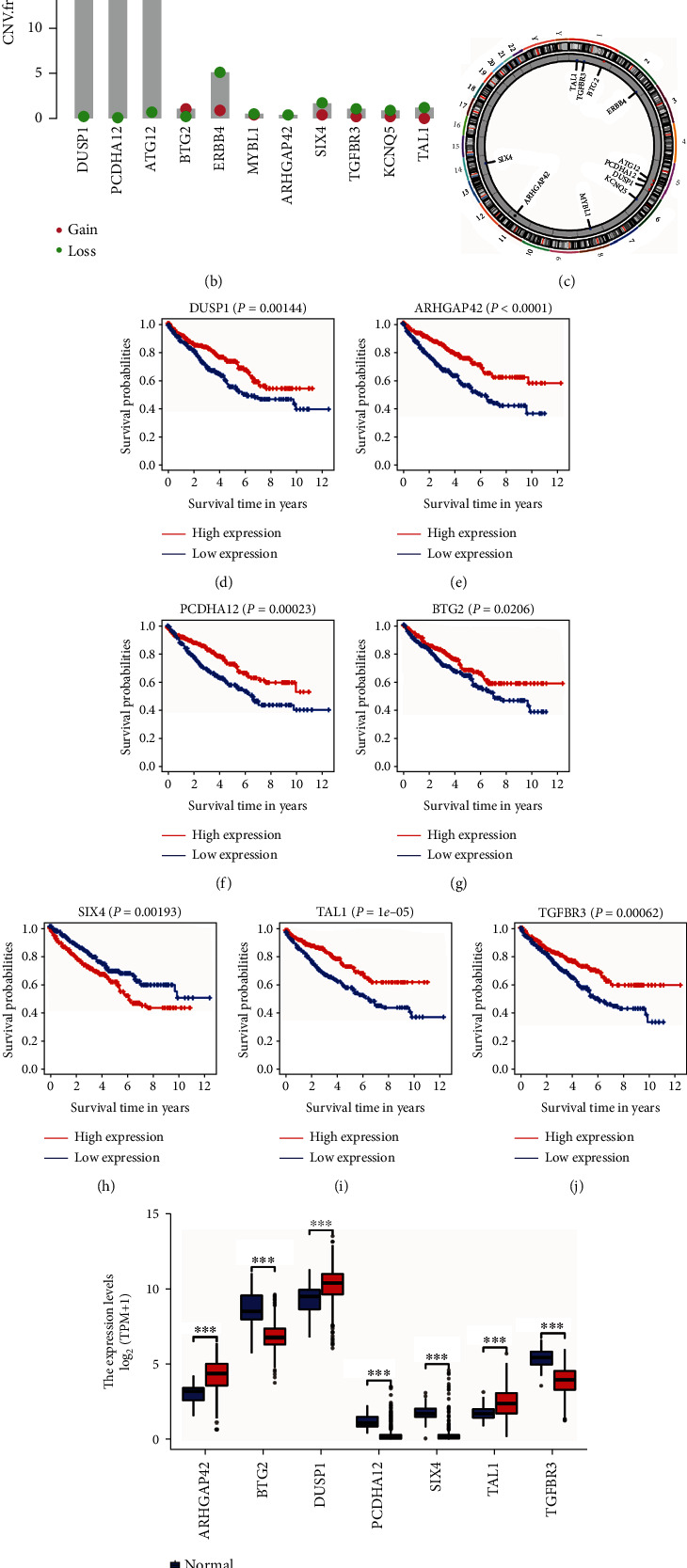
Gene alterations and survival analysis of hub genes. (a) Mutation landscape of 11 hub genes in 336 patients with ccRCC from TCGA dataset. (b) Copy number variation (CNV) frequency of 11 hub genes in TCGA cohort. The height of the columns represents the alteration frequency. (c) Location of CNV alteration of 11 hub genes. (d–j) Kaplan-Meier survival curves show seven hub genes associated with patient prognosis. Red: high expression; blue: low expression. (k) Expression differences of seven hub genes in tumor tissues and unpaired normal tissues. (l) Expression differences of seven hub genes in tumor tissues and paired normal tissues. Red: tumor; blue: normal; ns: *P* ≥ 0.05; ^∗^*P* < 0.05;  ^∗∗^*P* < 0.01;  ^∗∗∗^*P* < 0.001.

First, we analyzed the genetic changes of the 11 hub genes. Sixteen (4.76%) of 336 samples showed gene mutations, and ERBB4 showed the highest alteration frequency (Figure 8(a)). CNV frequencies were computed, and those of DUSP1, PCDHA12, and ATG12 were more than 15% (Figure 8(b)). The locations of CNV alterations on chromosomes are presented in Figure 8(c). Next, to further explore the prognostic value of the 11 hub genes, we conducted a K-M analysis. High expression of ARHGAP42 (*P* < 0.0001), BTG2 (*P* = 0.0206), DUSP1 (*P* = 0.00144), PCDHA12 (*P* = 0.00023), TAL1 (*P* = 1*e* − 05), and TGFBR3 (*P* = 0.00062) was associated with poor patient prognosis, whereas high expression of SIX4 (*P* = 0.00193) correlated with a better prognosis (Figures 8(d)–8(j)). The differential expression of seven OS-related hub genes between tumor tissues and unpaired normal tissues is illustrated in Figure 8(k). Furthermore, differences in expression between tumor tissues and paired normal tissues are shown in Figure 8(l).

### 3.8. Tumor Infiltration and Drug Sensitivity Analyses

**Figure 9:**
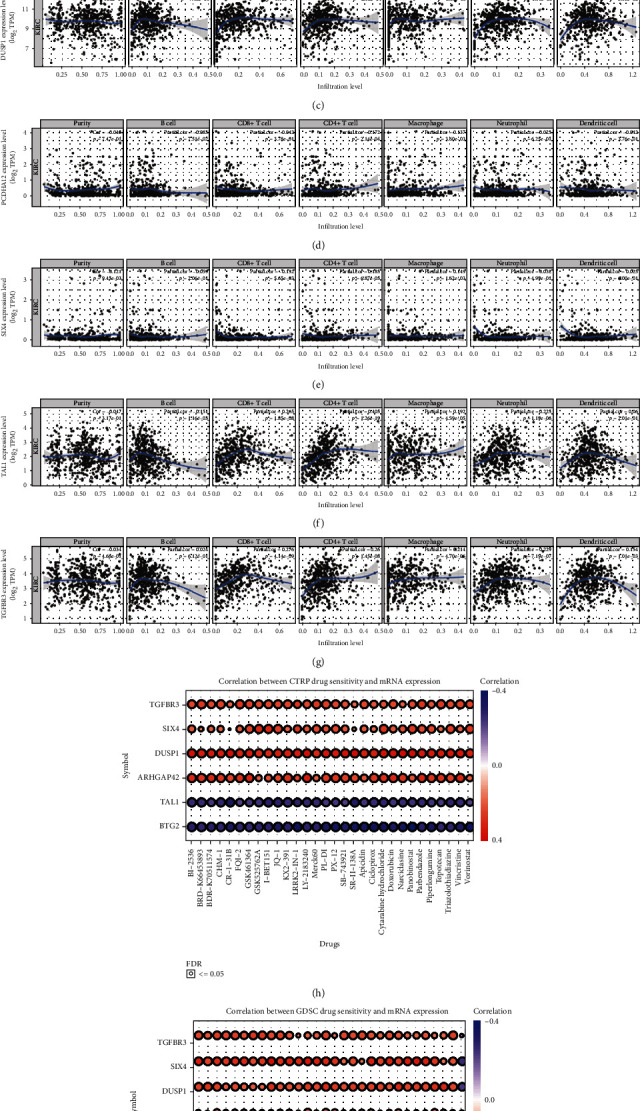
Tumor infiltration and drug sensitivity analysis of OS-related hub genes. (a–g) The tumor immune infiltration status of seven OS-related hub genes analyzed by TIMER. (h) Correlation between the expression of OS-related hub genes and CTRP drug sensitivity. (i) Correlation between the expression of OS-related hub genes and GDSC drug sensitivity.

We explored the relationship between the immune infiltration status and expression levels of the seven OS-related hub genes using TIMER, which suggested that the mechanism of hub genes is associated with tumor immunity (Figures 9(a)–9(g)).

To explore the possible treatment targets, we investigated the correlation between OS-related hub genes and existing drugs in a pan-cancer analysis using the GSCA database, which integrated genomic data from TCGA and over 750 drugs from CTRP and GDSC. In the current study, drug sensitivity analysis represented the correlation between gene expression and sensitivity to drugs from CTRP and GDSC (Figures 9(h) and 9(i)). TGFBR3, SIX4, DUSP1, and ARHGAP42 were positively correlated, whereas TAL1, and BTG2 were negatively correlated, with drug sensitivity.

### 3.9. Prediction and Verification of Expression Levels of 6 Necroptosis-Related miRNAs

**Figure 10:**
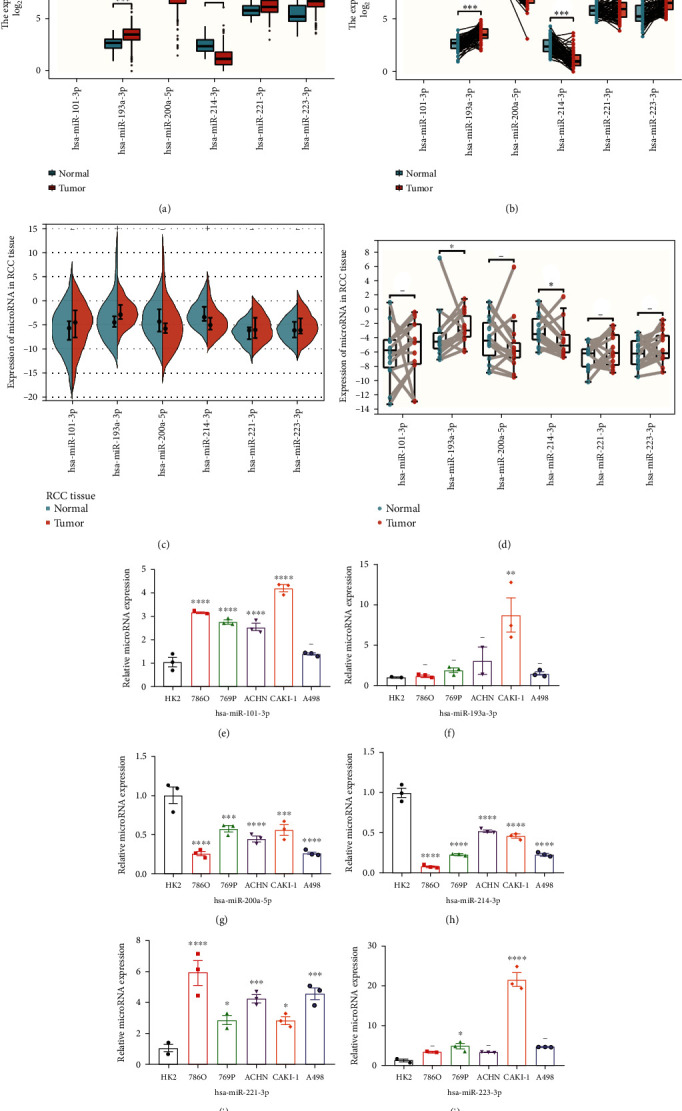
Prediction and verification of expression levels of 6 necroptosis-related miRNAs. (a, b) The expression levels of 6 necroptosis-related miRNAs between normal and tumor tissues predicted in TCGA-KIRC. (c, d) The expression levels of 6 necroptosis-related miRNAs in RCC tissues verified by RT-qPCR. (e–j) The expression levels of each miRNA compared to U6 by RT-qPCR in the renal epithelial cell line (HK2) and 6 human renal cancer cell lines (786O, 769P, ACHN, CAKI-1 and A498). (^∗∗∗^*P* < 0.001;  ^∗∗^*P* < 0.01;  ^∗^*P* < 0.05; -: no significance).

By analyzing the miRNA expression profiles from TCGA-KIRC, we explored the expression levels of tumor and unpaired normal tissues of 6 necroptosis-related miRNAs in our risk signature (Figure 10(a)) and between tumor and adjacent normal tissues (Figure 10(b)). To validate the expression tendency of them, we conducted RT-qPCR in tumor samples and cell lines. In our validation results in 12 paired RCC samples, we found has-miR-193a-3p was significantly high expressed in RCC patients and has miR-214-3p was significantly low expressed (Figures 10(c) and 10(d)). Meanwhile, in the verification results in cell lines, we found the expression tendency of 6 miRNAs was consistent with prediction results (Figures 10(e)–10(j)).

## 4. Discussion

Necroptosis, a caspase-independent form of programmed cell death, has a dual effect on cancer progression and metastasis. The exact role of necroptosis in the regulation of cancer remains controversial, and seems to be highly dependent on the tumor stage [18]. Although some studies have suggested that necroptosis can promote cancer progression and metastasis [29], others have reported that it can exert anti-tumor effects by compensating for apoptotic resistance [30, 31]. Hence, the exact prognostic value of necroptosis is still unclear, and no necroptosis-related molecular signature has been constructed until now. In this study, we explored the expression levels of miRNAs associated with necroptosis in ccRCC. By applying univariate Cox proportional hazard regression analysis and LASSO Cox regression analysis, we constructed a six-miRNA risk signature, which exhibited good performance in the training, testing, and entire cohort. Additionally, we showed that our signature was an independent prognosis predictor factor and established a predictive nomogram that can be used to apply our signature to a clinical setting. Both ROC and calibration analyses showed the good performance of our model. However, additional data are needed to verify the feasibility before implementing its use.

In the current study, we established a signature and nomogram and also explored the potential function of key miRNAs, which can lay a preliminary foundation for further in vivo or in vitro studies aiming to elucidate the role of programmed cell death in cancer, and, specifically, in ccRCC [32]. To the best of our knowledge, we are the first to establish an NRM signature in ccRCC and inform about potentially relevant genes and miRNAs using it.

In our study, six key miRNAs were identified, among which miR-193a-3p and miR-223-3p were markedly significant in the K-M analysis. The expression levels of 6 miRNAs were verified in RCC tissue samples and cell lines, which showed the consistent tendency with predicted results. The function of miR-193a-3p and miR-223-3p in cancer progression has been reported in various cancers that they have a role in tumorigenesis, progression, and metastasis. Although miR-193a-3p and miR-223-3p are characterized as oncogenes in certain cancers and tumor suppressors in others, their role in RCC was consistent according to existing researches.

Many studies revealed the oncogene function of miR-193-3p in renal cancer. Liu et al. first demonstrated that miR-193a-3p is upregulated in RCC tissues and cell lines and knockdown of miR-193a-3p can significantly inhibited cell proliferation and migration by directly targeting PTEN [33]. Pan et al. also found high expression level of miR-193a-3p and overexpression of miR-193a-3p can activate PI3K/Akt pathway and function as oncogenic by targeting ST3GalIV in RCC [34]. Moreover, Yu et al. showed that miR-193a-3p functions as a tumor inhibitor by targeting the ERBB signaling pathway in non-small-cell lung cancer [35]. In terms of miR-223-3p, Xiao et al. found miR-223-3p was highly expressed in ccRCC patients and patients with higher expression level of miR-223-3p always had higher tumor stages and grades and poorer prognosis. The results revealed miR-223-3p could bind directly to solute carrier family 4, member 4 (SLC4A4) mRNA, and reduced SLC4A4 mRNA and protein expression, which was associated with KRAS signaling and epithelial-mesenchymal transition [36]. Besides, Zhang et al.'s study confirmed the carcinogenesis of miR-223-3p and found RASA1 may play a key role in the progression of RCC by decreasing miR-223-3p and subsequently increasing FBXW7 expression [37].

Many molecules have been identified to facilitate or suppress cancer prognosis via miR-193a-3p. For example, LncRNA ZNFX1-AS1 targets miR-193a-3p/SDC1 to regulate cell proliferation, migration, and invasion of bladder cancer cells [38]. Similarly, molecules including long noncoding RNA SLCO4A1-AS1, Hsa_circ_0003159, and circle RNA circABCB10 which modulates PFN2 were found to interact with miR-223-3p to regulate cancer progression [39–41]. However, the upstream mechanism of these 2 microRNAs is still unclear in ccRCC. Jia et al. found miR-193a-3p is an androgen receptor target gene, whose expression level can be regulated by androgen and promotes prostate cancer cell migration through its direct target AJUBA gene [42]. Yang et al. observed that H. pylori infection contributed to higher expression level of miR-223-3p in gastric cancer via NF-*κ*B-dependent pathway. NF-*κ*B directly bound to the promoter of miR-223-3p so that the expression of miR-223-3p was stimulated, and miR-223-3p played the oncogenic role in gastric cancer by directly targeting ARID1A. They suggested miR-223-3p might act as a “bridge” to link H. pylori-induced chronic inflammation and carcinogenesis [43]. In summary, the factors contributing to high expression of miR-193a-3p and miR-223-3p in ccRCC should be addressed in further studies.

Furthermore, another study suggested that the invasion and deterioration of breast cancer are strongly associated with the high expression of miR-221-3p, which demonstrates the poor prognosis and advanced stage of BC [44]. Cao et al. demonstrated that miR-101-3p can be inhibited by LINC01303 and then enhances gastric cancer progression [45], and Wang et al. indicated that the expression of miR-200a-5p downregulates the antitumor gene FOXD1 in high-grade serous ovarian carcinoma [46]. Although these studies show the importance of miRNAs in cancer, the role of necroptosis-related miRNAs has not yet been fully studied. GO and KEGG enrichment analyses of the target genes of the six miRNAs identified indicated that these genes were mostly involved in cell proliferation and oxygen response-related biological processes. We hypothesize that these target genes participate in the production of reactive oxygen species (ROS), which have been widely suggested to form a positive pathological feedback loop with necroptosis under pathophysiological conditions [47]. FoxO, MAPK, and PI3K/AKT signaling pathways are activated when the ROS concentration is high. Additionally, MAPK and PI3K/AKT signaling pathways are important targets in regulating necroptosis in in vivo and in vitro experiments [48]. Hence, we hypothesized that these pathways might mediate the crosstalk between necroptosis and ROS production. However, ROS are also associated with other cell death pathways, including apoptosis, autophagy, and ferroptosis, and the role of ROS in metastatic cancers remains controversial. The diversity of ROS effects on different tumor cell types could underlie the dual roles of necroptosis in cancer metastasis. Additionally, our results suggest that necroptosis is involved in the regulation of the tumor immune response.

Finally, we analyzed the tumor infiltration and drug sensitivity status of hub genes. Surprisingly, the correlation between the expression of hub genes and drug sensitivity to drugs from the CTRP and GDSC databases was remarkable, which reveals that miRNAs and their target genes might be new therapeutic targets in cancer. By analyzing the correlation between drug sensitivity and hub genes' mRNA expression levels, our study could improve drug tolerance and developing new drugs for cancer, which should be validated by in vivo and in vitro experiments in the future.

Our study had several limitations, first, both our training and testing cohorts were obtained from TCGA. Because data of miRNA expression with the corresponding clinical information and sufficient sample size are not available besides those from TCGA, we could not conduct external validation for the developed signature. Thus, more ccRCC samples are required for further validation of the risk signature. Second, we preliminarily explored the prognostic value of these miRNAs and their target genes. However, owing to the insufficient literature on the topic, the relationship between the identified molecules (six selected miRNAs and potential target genes) and response to cancer therapy could not be investigated. The mechanisms underlying the relationship between cancer development and the signature miRNAs and their target genes are still unclear, and further research is required. Nonetheless, our study provides an informed starting point for further studies.

## 5. Conclusion

In summary, we established a novel necroptosis-related miRNA signature and nomogram for predicting the prognosis of patients with ccRCC, both of which achieved good prediction accuracy. Additionally, six miRNAs (hsa-miR-101-3p, hsa-miR-193a-3p, hsa-miR-200a-5p, hsa-miR-214-3p, hsa-miR-221-3p, and hsa-miR-223-3p) and seven hub target genes (ARHGAP42, BTG2, DUSP1, PCDHA12, TAL1, TGFBR3, and SIX4) were dysregulated in ccRCC.

## Data Availability

The analyzed data could be obtained at TGCA using the GDC Data Portal (https://portal.gdc.cancer.gov/). The code applied in the study is available from the corresponding authors on reasonable request.

## Supplementary Materials

Supplementary Materials Supplementary Figure 1: univariable Cox regression and multivariate Cox regression analysis in testing cohort and entire TCGA cohort. (a and b) The result in the testing cohort; (c and d) the result in the entire TCGA cohort. Supplementary Figure 2: LASSO regression analysis of the 14 overlapping genes identified by 3 algorithms. (a) LASSO regression of the 14 overlapping genes. (b) Tenfold cross-validation for tuning the parameter selection in the LASSO regression. Supplementary Table 1: clinicopathological characteristics of patients with ccRCC in TCGA. Supplementary Table 2: necroptosis-related miRNAs. Supplementary Table 3: oligo sequences used in quantitative real-time PCR. Supplementary Table 4: exploration of target genes of miRNAs.
