# Supplementary material for: Deciphering a Novel Necroptosis-Related miRNA Signature for Predicting the Prognosis of Clear Cell Renal Carcinoma
Source: Anal Cell Pathol (Amst). 2022 Apr 25;2022:2721005. doi: 10.1155/2022/2721005 (PMC9061065; doi:10.1155/2022/2721005)
Supplement: Supplementary Materials — Supplementary Figure 1: univariable Cox regression and multivariate Cox regression analysis in testing cohort and entire TCGA cohort. (a and b) The result in the testing cohort; (c and d) the result in the entire TCGA cohort. Supplementary Figure 2: LASSO regression analysis of the 14 overlapping genes identified by 3 algorithms. (a) LASSO regression of the 14 overlapping genes. (b) Tenfold cross-validation for tuning the parameter selection in the LASSO regression. Supplementary Table 1: clinicopathological characteristics of patients with ccRCC in TCGA. Supplementary Table 2: necroptosis-related miRNAs. Supplementary Table 3: oligo sequences used in quantitative real-time PCR. Supplementary Table 4: exploration of target genes of miRNAs. [file 2721005.f1.doc]

## Supplementary Materials

## Supplementary Figures


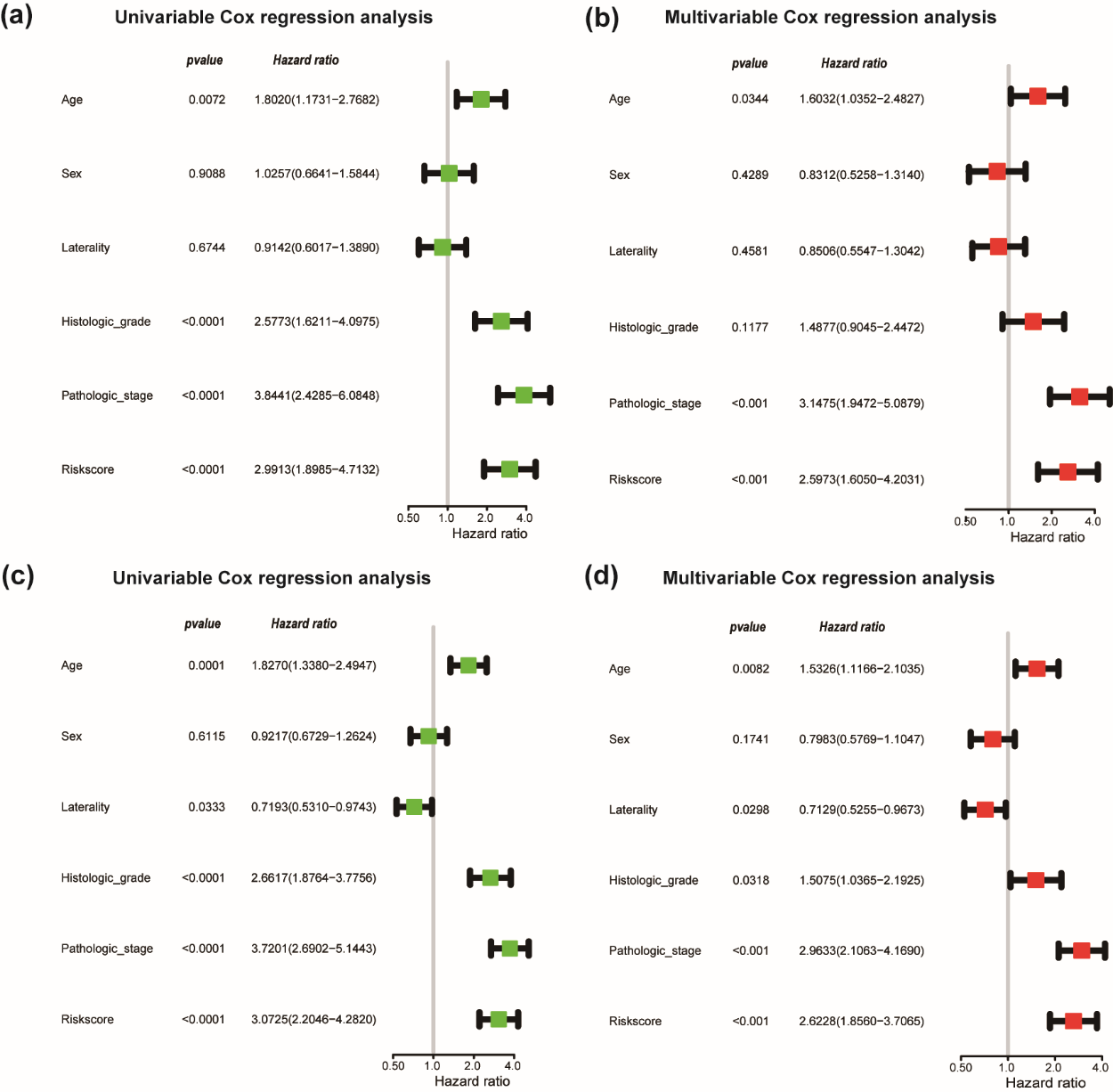


**Supplementary Figure 1.** Univariable Cox regression and multivariate Cox regression analysis in testing cohort and entire TCGA cohort. a and b, the result in testing cohort; c and d, the result in entire TCGA cohort.


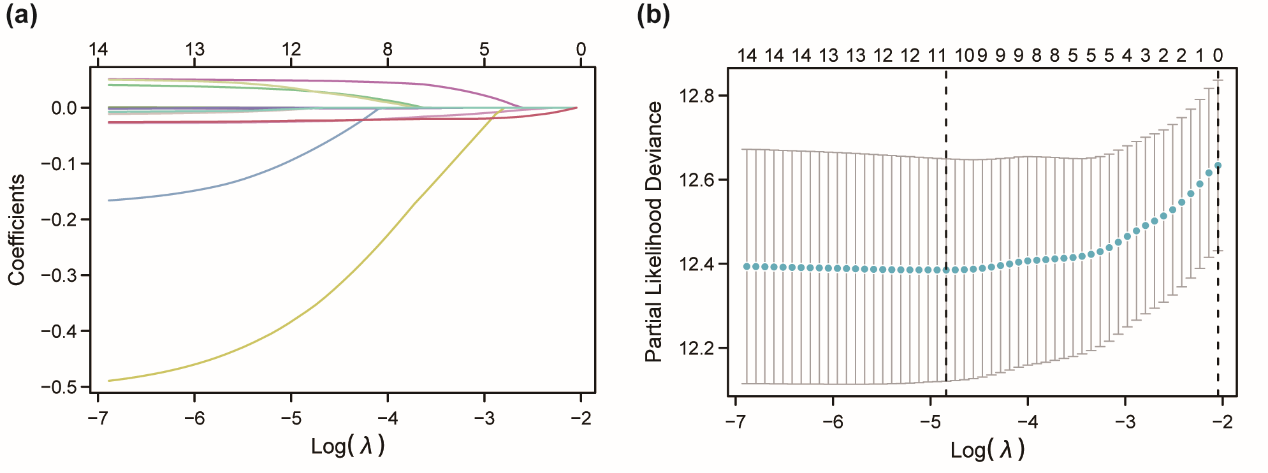


**Supplementary Figure 2.** LASSO regression analysis of the 14 overlapping genes identified by 3 algorithms. a, LASSO regression of the 14 overlapping genes. b, Ten-fold cross-validation for tuning the parameter selection in the LASSO regression.

## Supplementary Tables

**Supplementary Table 1.** Clinicopathological characteristics of patients with ccRCC in TCGA

| **Table S1 The clinicopathological characteristics of ccRCC patients in TCGA** | | | |
| --- | --- | --- | --- |
| Characteristic | levels | TCGA-miRNA | TCGA-mRNA |
| n |  | 545 | 539 |
| Gender, n (%) | Female | 188 (34.5%) | 186 (34.5%) |
|  | Male | 357 (65.5%) | 353 (65.5%) |
| Age, n (%) | <=60 | 274 (50.3%) | 269 (49.9%) |
|  | >60 | 271 (49.7%) | 270 (50.1%) |
| Histologic grade, n (%) | G1 | 15 (2.8%) | 14 (2.6%) |
|  | G2 | 237 (44.3%) | 235 (44.3%) |
|  | G3 | 208 (38.9%) | 207 (39%) |
|  | G4 | 75 (14%) | 75 (14.1%) |
| Pathologic stage, n (%) | Stage I | 275 (50.7%) | 272 (50.7%) |
|  | Stage II | 60 (11.1%) | 59 (11%) |
|  | Stage III | 125 (23.1%) | 123 (22.9%) |
|  | Stage IV | 82 (15.1%) | 82 (15.3%) |
| T stage, n (%) | T1 | 281 (51.6%) | 278 (51.6%) |
|  | T2 | 72 (13.2%) | 71 (13.2%) |
|  | T3 | 181 (33.2%) | 179 (33.2%) |
|  | T4 | 11 (2%) | 11 (2%) |
| N stage, n (%) | N0 | 237 (93.3%) | 241 (93.8%) |
|  | N1 | 17 (6.7%) | 16 (6.2%) |
| M stage, n (%) | M0 | 434 (84.8%) | 428 (84.6%) |
|  | M1 | 78 (15.2%) | 78 (15.4%) |
| OS event, n (%) | Alive | 371 (68.1%) | 366 (67.9%) |
|  | Dead | 174 (31.9%) | 173 (32.1%) |
| DSS event, n (%) | Alive | 425 (79.7%) | 420 (79.5%) |
|  | Dead | 108 (20.3%) | 108 (20.5%) |
| PFI event, n (%) | Alive | 383 (70.3%) | 378 (70.1%) |
|  | Dead | 162 (29.7%) | 161 (29.9%) |

**Supplementary Table 2.** Necroptosis-related miRNAs

**TableS2. Necroptosis-Related-miRNAs**

| **Necroptosis-Related-miRNAs** |
| --- |
| miR-92a-3p |
| miR-874 |
| miR-7-5p |
| miR-512-3p |
| miR-500a-3p |
| miR-499 |
| miR-495 |
| miR-48-5p |
| miR-425-5p |
| miR-383 |
| miR-381-3p |
| miR-351 |
| miR-331-3p |
| miR-29b-3p |
| miR-22-3p |
| miR-223-3p |
| miR-221-3p |
| miR-218 |
| miR-214-3p |
| miR-210 |
| miR-21 |
| miR-200a-5p |
| miR-193a-3p |
| miR-19 |
| miR-181b-1 |
| miR-181-5p |
| miR-16-5p |
| miR-15a |
| miR-155-5p |
| miR-155 |
| miR-148a-3p |
| miR-146 |
| miR-141-3p |
| miR-128a |
| miR-101-3p |
| miR-155-5p |
| miR-325-3p |
| miR-223-5p |
| miR-223-3p |

**Supplementary Table 3.** Oligo sequences used in quantitative real-time PCR

| **Table S3 Oligo sequences used in quantitative real-time PCR** | | | | |
| --- | --- | --- | --- | --- |
| **species** | **miRNA name** | **miRNA sequences** | **Oligo sequences** | |
| *Homo sapiens* | hsa-miR-101-3p | UACAGUACUGUGAUAACUGAA | Forward | GCGGCGTACTGTGATAACTGA |
| *Homo sapiens* | hsa-miR-193a-3p | AACUGGCCUACAAAGUCCCAGU | Forward | TGGGAACTGGCCTACAAAGTC |
| *Homo sapiens* | hsa-miR-200a-5p | CAUCUUACCGGACAGUGCUGGA | Forward | TGGCATCTTACCGGACAGTG |
| *Homo sapiens* | hsa-miR-214-3p | ACAGCAGGCACAGACAGGCAGU | Forward | GGACAGCAGGCACAGACAG |
| *Homo sapiens* | hsa-miR-221-3p | AGCUACAUUGUCUGCUGGGUUUC | Forward | TGGGTGCTACATTGTCTGCTG |
| *Homo sapiens* | hsa-miR-223-3p | UGUCAGUUUGUCAAAUACCCCA | Forward | GGTGGGTGTCAGTTTGTCAAATAC |

**Supplementary Table 4.** Exploration of target genes of miRNAs

| **Table S4. Exploration of miRNAs' target genes** | | | |
| --- | --- | --- | --- |
| **miRDB** | **miRTarBase** | **TargetScan** | **Target Genes** |
| NDUFB5 | FZD6 | DCAF10 | NDUFB5 |
| NAV1 | WNT7A | ARRB1 | RANBP9 |
| RANBP9 | EZH2 | CFL2 | RAB5A |
| SGMS2 | FKBP14 | HNRNPF | RAC1 |
| RAB5A | LTN1 | KCNA6 | NACA2 |
| RAC1 | BIRC5 | DNAJC12 | NAIP |
| RANBP1 | C10orf88 | CRTAP | NDFIP1 |
| STAU2 | LEFTY1 | ACSM4 | RORA |
| NACA2 | RMI1 | CRTC1 | SACM1L |
| NAIP | PTGS2 | KLF5 | RNF219 |
| RIPK1 | MKLN1 | KIF1A | TAL1 |
| NDFIP1 | ZFP36L2 | AAMP | TBC1D12 |
| NDST3 | DSC1 | DCAF12 | TET2 |
| RNF38 | KIF2C | ASH1L | TGFBR1 |
| ROBO2 | DYNC1LI2 | DGCR2 | TGFBR3 |
| RORA | MPPE1 | AGTPBP1 | NEK7 |
| RRM1 | NCKAP1 | DBI | NF1 |
| RSF1 | MORC3 | ZNF844 | NLK |
| RXRB | EXTL3 | ASB14 | NOTCH1 |
| SACM1L | REL | ASAP1 | NUPL2 |
| SASH1 | ANKRD11 | ADAM28 | OTUD4 |
| SCN2A | DIDO1 | B3GALTL | PANK3 |
| SCN8A | PPP1CC | AP5M1 | SEPT11 |
| SEL1L | IPO7 | CRNKL1 | SUB1 |
| RNF19A | KDM3B | CRP | RAB15 |
| RNF219 | CDK8 | ACTR3 | RAB1A |
| SGK1 | TBX18 | ZNF780A | RAB39B |
| SULT4A1 | VEGFC | ATG5 | RAP1B |
| SUPT7L | TSN | ARPP19 | RAP2C |
| SYNCRIP | PLAG1 | ZRANB1 | ARID1A |
| SYT4 | CDKN1A | ANKRD32 | STMN1 |
| TAGAP | KPNA2 | ATG4D | STX6 |
| TAL1 | SREK1IP1 | AC079602.1 | BAZ2A |
| TBC1D12 | APP | JAKMIP3 | SGPL1 |
| RANBP6 | PAFAH1B1 | ACACA | SLC30A7 |
| TDG | INO80D | AC135178.1 | SLC38A2 |
| TET2 | PGBD4 | ASAH1 | SLC39A6 |
| TEX2 | FNDC3A | KCNJ8 | SMARCD1 |
| TFB2M | PDK1 | CRTC3 | SMN2 |
| TGFBR1 | SOX9 | ADCY5 | SOCS5 |
| TGFBR3 | DNMT3A | CYSLTR1 | SPATA2 |
| NEGR1 | AMMECR1L | KIF27 | STAMBP |
| NEK4 | CBFA2T2 | ARL4C | STAR |
| NEK7 | ZNF124 | ACADSB | ATXN1 |
| NEMP2 | RAC1 | ZNRF2 | ATXN1L |
| NF1 | NACC1 | ASH2L | B3GALNT2 |
| NLK | JCAD | DCDC2 | PIK3C2B |
| NOTCH1 | RNF219 | AC023632.1 | PIP5K1C |
| NOVA1 | GNB1 | ARRDC4 | POGZ |
| NPNT | CD46 | AMMECR1L | PPARGC1B |
| NR1D2 | KCNQ5 | ADCK1 | PPM1L |
| NRK | NOTCH1 | KCNK1 | PRKAA1 |
| NSD1 | MCL1 | B3GALT6 | PRRC2C |
| NUPL2 | LCOR | ZSWIM2 | PTBP3 |
| OGT | AP3M1 | ADARB2 | ABHD17C |
| OTUD3 | C1orf52 | ATG2B | ABLIM3 |
| OTUD4 | MRPL42 | ASCC3 | AEBP2 |
| PABPC5 | HNRNPF | ACSBG1 | AGAP1 |
| PACRG | IL20RB | ACSL3 | AKT3 |
| PANK3 | TGIF2 | ARNTL2 | ANKRD11 |
| PAPOLG | SPIRE1 | DNAJB9 | ANKRD17 |
| PBX3 | BZW1 | KCNJ9 | AP1S3 |
| PCDH20 | NR2F2 | ASAP2 | ARAP2 |
| STC1 | CCNF | DCP2 | EYA1 |
| SELENOI | ZNF827 | B3GAT2 | EZH2 |
| SEPT11 | BCL2L11 | BEX5 | FAM103A1 |
| SUB1 | TOR1AIP1 | AGPAT6 | FAR1 |
| RAB15 | MBTD1 | ASAH2B | FBN2 |
| RAB1A | CADM1 | AGXT2 | FBXW7 |
| RAB27A | PHF3 | ACTR3B | SIX4 |
| RAB39B | LDB1 | HMGCR | FOS |
| RAB4A | RPS6KA5 | ADCY6 | FZD6 |
| ADAMTS17 | NKX3-2 | ATP13A4 | GJA1 |
| ADAMTS3 | AP1G1 | ANKRD34C | GNB1 |
| TBRG1 | ZDHHC24 | ADAMTS3 | GRSF1 |
| ADH5 | TBX20 | CXCL6 | CDH5 |
| ADRB1 | PEX5L | ATP5B | CDK8 |
| RAP1B | XPO7 | ATP5A1 | CDKN1A |
| RAP2C | RAB5A | ADAM12 | CERS2 |
| RAPH1 | TTC37 | ATP5F1 | TMED5 |
| RASD2 | DCAF12L2 | ATP5G1 | TMEM170B |
| RASGRP3 | MNX1 | ATP5G3 | TNKS2 |
| RBBP7 | TSPAN12 | DCTN4 | TNPO1 |
| RBM25 | FAM217B | ASPN | PTGS2 |
| RCN2 | GFPT2 | ADAM9 | UBE2A |
| REV3L | GLRX5 | KCNMB1 | UBN2 |
| RFPL4B | SLC11A2 | ZNF776 | LRCH2 |
| RFX3 | DIMT1 | ARSJ | MAML3 |
| RHOT1 | ZNF431 | ADAMTS20 | MAP3K2 |
| RIC1 | LANCL3 | ATAD2 | MAPK1 |
| RIN2 | SUB1 | KCNK12 | MBNL1 |
| ARFGEF3 | CTR9 | ADAMTS5 | MFSD6 |
| ARHGAP32 | ADO | ADAMTSL3 | MLEC |
| ARHGEF10 | PPP1R15B | KCNK5 | MNX1 |
| ARHGEF3 | AMD1 | ARRDC3 | MOB4 |
| ARID1A | TMED5 | NDUFA10 | MPPE1 |
| ARNTL2 | MYCN | KCNJ6 | MRGBP |
| ASAP1 | TOR1AIP2 | ATF7IP2 | MRPL42 |
| ASCC3 | NOP2 | ZNF841 | MTSS1L |
| ASPN | UBE2D3 | ACSL4 | MYCN |
| ATP11B | MTOR | ADAM19 | N4BP1 |
| ATP8A1 | SNRNP35 | ANXA2 | NACA |
| ATRNL1 | UBN2 | HNRNPD | BICD2 |
| ATRX | RAP1B | GALNT3 | C1orf52 |
| STMN1 | DDIT4 | ASB15 | CADM1 |
| STX6 | MRPL44 | ACTRT3 | CAMKK1 |
| PYGO2 | C8orf4 | ART4 | CAPN2 |
| B3GNT3 | GRIK3 | CRYBG3 | CASP3 |
| BAZ2A | ZNF800 | AVPR1A | CBFA2T2 |
| BBS7 | LIN28B | ALDH1L2 | HNRNPA0 |
| BBX | ZC3H11A | DCHS2 | HNRNPF |
| SGPL1 | ANKRD17 | HNRNPH2 | HSPE1-MOB4 |
| SH2B3 | KCNG3 | ANKRD34B | ICK |
| SHISA6 | USP36 | DBX2 | INO80D |
| ADAMTSL3 | PRKAB1 | ASCC1 | JAK2 |
| SLC12A2 | ZNF100 | ANKRD29 | CTCF |
| SLC19A2 | INA | AC137932.1 | DCAF7 |
| SLC1A1 | TMEM168 | CSF2RA | DCBLD2 |
| SLC25A4 | ZCCHC2 | ARHGAP15 | DDIT4 |
| SLC2A13 | FMR1 | AP4M1 | DNMT3A |
| SLC30A7 | MAP3K4 | AP5B1 | DSC1 |
| SLC38A2 | OTUD4 | DLGAP5 | DUSP1 |
| SLC39A10 | GPAM | GRAPL | EED |
| SLC39A6 | G3BP1 | ASIC1 | ZFP36L2 |
| SLC7A11 | PCCB | CPSF2 | ZMAT3 |
| SLTM | EEA1 | HMGCS1 | ZNF207 |
| SMARCA1 | VHL | ALG5 | BCL2L11 |
| SMARCA4 | KLF12 | AMOT | BCL9 |
| SMARCD1 | LRRC1 | DDIT4 | KDM3B |
| SMN1 | SLC25A33 | DDAH1 | KDM6B |
| SMN2 | DCBLD2 | AKAP12 | DENR |
| SOAT1 | CTNNB1 | KCNJ15 | KIAA1586 |
| SOCS5 | AP1S3 | KCNJ3 | KLF6 |
| SPATA2 | STAMBP | KIAA1715 | ZCCHC2 |
| SPATS2L | VAPA | MYRIP | LCOR |
| SPG11 | BTRC | ATRX | LIN7C |
| SPOP | MBNL1 | ZNF827 | LMNB1 |
| SPRED1 | COX2 | ARPC4 | LANCL3 |
| SRPK2 | MFSD6 | ATG12 | ZEB1 |
| SSBP2 | STYX | ANGEL1 | ZBTB21 |
| ST7 | ELAVL3 | ANGEL2 | ZNF654 |
| STAG2 | ATXN1 | AC007375.1 | DKK2 |
| STAMBP | RAP2C | ASAH2 | DCTN5 |
| STAR | TNPO1 | AC074091.13 | FAM84A |
| ATXN1 | UBE2A | ASB1 | IRF1 |
| ATXN1L | NANOGNB | ZRANB2 | DYRK2 |
| B3GALNT2 | ARID1A | DKFZP434E1119 | E2F6 |
| PCK1 | FGF2 | DNAJC18 | EIF2AK1 |
| PDE4D | CARNMT1 | AGMO | ERBB4 |
| PDP1 | SMARCA5 | ZNHIT6 | PHLDA2 |
| PDS5B | ATG4D | CYP24A1 | PLAU |
| PHACTR2 | NXT2 | ACVR1 | PLEKHA2 |
| PHF20L1 | DAZAP2 | DCSTAMP | PMAIP1 |
| PHTF2 | ZNF350 | ALG10B | PRR14L |
| PIEZO1 | C1orf147 | ACVR2B | GDF11 |
| PIK3C2B | JUN | AP3S2 | RAB27B |
| PIKFYVE | ITGA3 | DCTN2 | RGMA |
| PIP5K1C | RAB33B | KCND2 | RRAS2 |
| PITPNB | IER5 | JAK3 | SLC10A6 |
| PKD2 | DNAJA1 | ASB5 | STX16 |
| PLA2R1 | GPR135 | KCNE1 | TAPT1 |
| PLEKHG1 | HSP90B1 | AP4E1 | TGFB2 |
| PLXNA2 | FBXW7 | DCTN3 | TMEM30A |
| PMPCB | ATP5B | APAF1 | TMPPE |
| PNISR | DYRK2 | ZSWIM5 | TNFAIP1 |
| POGK | CLIC4 | B3GNT3 | IRF2BPL |
| POGZ | NLK | ATF1 | KCNQ5 |
| POLR3K | CPS1 | ATE1 | KIT |
| POMP | BTG2 | AMER2 | KMT2A |
| PPARGC1B | PPP2R2A | ATP6V1G1 | KRAS |
| PPFIA1 | SPATA2 | KCNJ13 | LAMC1 |
| PPFIA2 | RPL7L1 | AMIGO2 | LIMS1 |
| PPM1L | HSPA13 | AMPD3 | LPCAT1 |
| PPP1R2 | RNF44 | ATF7IP | LUZP1 |
| PPTC7 | PLEKHA1 | ACOX1 | LYRM2 |
| PRELID3B | PIK3CB | ARMS2 | MAPK8 |
| PRKAA1 | MEIS1 | AKAP14 | MDH2 |
| PRKCE | STMN1 | NDUFA9 | MSANTD2 |
| PRKD3 | PSPC1 | DNAJB4 | NQO2 |
| PRPF4B | RORA | KIAA1614 | CAPRIN1 |
| PRR11 | TMEM192 | AGA | CCND1 |
| PRRC2C | TGFBR3 | ANGPT1 | ARMC1 |
| PTBP3 | NAP1L1 | KBTBD2 | BEND4 |
| PTCH1 | RBM12B | CROT | BTRC |
| PCDH7 | VEZT | AGPS | TWF1 |
| PCDH8 | ATG12 | BET1L | SRSF2 |
| PCGF5 | ZBTB7A | ZNHIT3 | MCL1 |
| PYGO1 | TGFBR1 | CRY2 | YWHAZ |
| ABCC5 | DUSP1 | ATP13A3 | ZBTB5 |
| ABHD17B | TGFBR2 | ABCA13 | ZC3H11A |
| ABHD17C | STX16 | ALDH3A2 | ZKSCAN8 |
| ABLIM3 | PIM1 | BFAR | C5orf22 |
| ACAD9 | DNAJC28 | DCLK1 | ABI2 |
| UGGT1 | N4BP1 | B3GALT2 | WDR82 |
| UNC79 | MAP2K1 | ZRSR2 | ADRA2B |
| UNKL | BLOC1S6 | ADAL | HMGB1 |
| USP38 | SPAG1 | ACYP1 | FOXC1 |
| USP47 | TMEM170B | ANKRD27 | GXYLT1 |
| AEBP2 | RNF152 | ASB4 | KLF9 |
| AGAP1 | HSP90AA1 | ATP2C1 | RIF1 |
| AGFG1 | AGO4 | DEPTOR | SNX4 |
| AJAP1 | TVP23C | DERL2 | SP3 |
| AKT3 | ZNF223 | DESI2 | THAP5 |
| ANKRD11 | RAB11FIP1 | ZSWIM4 | ZNF675 |
| ANKRD17 | ZFX | ATP5S | NAMPT |
| ANKRD44 | NACA2 | ASPA | NFIA |
| ANKZF1 | PLEKHA3 | ATAD2B | WNK1 |
| ANXA10 | ANKDD1A | ATAD1 | ZNF254 |
| AP1S3 | VEGFA | DHRS4 | DICER1 |
| AP3D1 | BICD2 | ASXL3 | PAFAH1B1 |
| AP3S1 | CDC42EP4 | ADAMTS19 | UBFD1 |
| ARAP2 | JAK2 | AMER3 | PAX6 |
| EXOC5 | GAN | AMIGO3 | ATP6V1E1 |
| EYA1 | HFE | ATF6 | ARL2 |
| EZH2 | CD180 | A1CF | ATG12 |
| FA2H | PABPC1L2A | ADAMTS4 | ALPK2 |
| FAM103A1 | SRF | IBTK | AMER1 |
| FAM114A1 | CDH5 | NOVA1 | AMMECR1L |
| FAM122C | TAF13 | A4GNT | ARHGAP42 |
| FAM169A | ZNF284 | AAGAB | CAPN5 |
| FAM214A | AEBP2 | AKIP1 | BTG2 |
| FAM216B | KDM6B | AGAP1 | C10orf76 |
| FAM46A | RRM2 | ACSM3 | C17orf49 |
| FAM53B | ZNF207 | ACSS3 | CACFD1 |
| FAM60A | XIAP | ACTN4 | ABLIM1 |
| FAM78A | QDPR | ANP32E | XBP1 |
| FAM83B | L2HGDH | DIXDC1 | ZBTB10 |
| FAR1 | FBXO11 | KRT222 | ZNF641 |
| FAT3 | ARID5B | CPD | ZNRF1 |
| FBN2 | MOB4 | CEBPD | DOCK9 |
| FBXO30 | RUNX1 | ARCN1 | ELFN2 |
| FBXW11 | RHOA | AXIN2 | ERC1 |
| FBXW7 | CMTM6 | ALG10 | FAM49B |
| FGA | KCTD14 | AHCTF1 | FGFR1 |
| FKTN | MLEC | AZIN1 | FLOT2 |
| SIX4 | FAR1 | ASB3 | GABARAP |
| FMNL3 | RTN4 | DCLRE1B | GALNT7 |
| FOS | ACVR2B | DCP1A | GAN |
| FOXN2 | PANK1 | ASPHD2 | GRB10 |
| FRYL | DENND5B | ARL4A | AHNAK2 |
| FUCA2 | RARS2 | FBXW2 | CRKL |
| FYTTD1 | RAB8B | ASPM | CS |
| FZD4 | CBX4 | DLL4 | CTNNB1 |
| FZD6 | MYO9A | JAZF1 | TFAP2C |
| GAB1 | RNF213 | ZNF780B | TGOLN2 |
| GABBR2 | UGT2A1 | ASXL2 | TMEM248 |
| GABRB2 | SZRD1 | ASZ1 | TOR1AIP2 |
| GCNT1 | ZNF654 | ZWINT | TRAF1 |
| GCNT3 | MAML3 | ATP6V1B2 | TRIM29 |
| GDE1 | PIK3CD | ZXDA | TSKU |
| GFRA1 | PRDM16 | DACH1 | UGT2A1 |
| GID4 | NAA30 | ZXDC | UGT2A2 |
| GJA1 | HSPE1-MOB4 | KLC2 | UHMK1 |
| GLCCI1 | SGPL1 | AKAP11 | VANGL1 |
| GLIPR1L1 | PAPD7 | ZNF81 | VAV2 |
| GLRA2 | SUZ12 | ACOT9 | VEZT |
| GNB1 | PPP4R1 | KAT2B | WASF2 |
| GPR85 | GMEB2 | KIAA1598 | NAGPA |
| GRIN2A | TMTC3 | ZNF84 | NAP1L4 |
| GRSF1 | TET2 | AKT3 | NCKAP1 |
| GSK3B | MKNK2 | ZNF852 | NFIC |
| H2AFV | PTGER4 | AC007405.2 | NUFIP2 |
| HAS2 | ATXN1L | AC008443.1 | PAPPA |
| HELZ | DICER1 | DKK3 | CDC42SE1 |
| HIVEP3 | GOLGA7 | KBTBD13 | CDK2 |
| CD86 | ZNF567 | ACSS2 | CNIH1 |
| CDH11 | COX10 | KBTBD6 | CPEB1 |
| CDH5 | USP25 | OPN4 | PSMD10 |
| CDK5R1 | BEND4 | ACTR1A | PTEN |
| CDK8 | TFAP4 | AREL1 | QKI |
| CDKN1A | CAPN2 | IL20RA | RAB1B |
| CDYL | FBN2 | AZI2 | RNF111 |
| CEBPA | POU2F1 | KCNA5 | SCAMP4 |
| CEP126 | PPP2R5E | ALG13 | SEMA4D |
| CEP350 | TBC1D12 | AC132872.2 | SESN3 |
| CEP63 | ZEB2 | B3GALT1 | SLC7A5 |
| CERS2 | LIN7C | AICDA | SNX24 |
| CERS6 | NEK7 | AP1S3 | HDGF |
| CHAC2 | AFF4 | KCNC4 | HNRNPU |
| CIR1 | TSC22D2 | DCT | ING4 |
| CISD2 | CCDC125 | CD34 | IPO7 |
| CLDN11 | FRMD6 | NUDT21 | JAG2 |
| CNIH3 | CHAMP1 | ADAM22 | KCTD15 |
| COL10A1 | TNFAIP1 | ADAM23 | KIF16B |
| COTL1 | HNRNPAB | ZNF788 | LTF |
| TIA1 | HOXA9 | ATP6V1D | LZTS1 |
| TIMM17A | SLC35F5 | ATP6V1H | MAP3K4 |
| TKTL1 | NUFIP2 | KIR2DL3 | MEF2C |
| TKTL2 | TRERF1 | ANKRD44 | MFN2 |
| TLK2 | PIP5K1C | ADAMTS16 | MIEF2 |
| TMED5 | PIAS1 | ADAMTS17 | MPDU1 |
| TMEM132D | RAB39B | ZYG11A | SPIRE1 |
| TMEM161B | MITF | ZNF808 | SSX2IP |
| TMEM167A | LZIC | ZNF804A | TBPL1 |
| TMEM170B | NACA | NDST3 | PGPEP1 |
| TMEM201 | AKAP11 | API5 | PHF21A |
| TMEM231 | LIFR | EIF4B | PIK3CB |
| TMEM65 | SMN2 | APLF | PIM1 |
| TMEM68 | EIF4G2 | APLN | PNPLA6 |
| TMF1 | KLF6 | ACPP | PRDM16 |
| TNKS2 | ALG14 | AASDHPPT | PRKAB1 |
| TNPO1 | GRSF1 | AC007390.5 | YOD1 |
| TNRC18 | RANBP9 | ZNF860 | ZEB2 |
| TOGARAM1 | MTSS1L | KIAA2018 | ZFP30 |
| TRIM24 | ICK | ALDH1L1 | ZNF275 |
| PTGS2 | PIK3C2B | ATP1A2 | TOX |
| PTPRJ | CERK | ANK3 | TRPC3 |
| PURG | ABHD17C | ADH5 | TRPS1 |
| UBE2A | PURB | ADHFE1 | TSPAN13 |
| UBE2D1 | DCTD | KCMF1 | TUB |
| UBE2D2 | CDC123 | AVL9 | UBE2J1 |
| UBE2F | SIX4 | KCNA4 | SMARCA5 |
| UBN2 | PRDM1 | ATP1B2 | SOD2 |
| UBR7 | WNK1 | AP000350.4 | STYX |
| LRCH2 | CERS2 | ACVR1B | TDRP |
| LRP2 | ZNF645 | ACVR1C | TIMP3 |
| LRRC4 | PRPF38B | ADSL | TMCC1 |
| LRRN1 | GPR50 | DFNA5 | CCSAP |
| MAB21L3 | HNRNPU | ANKRD13C | CDKN1B |
| MAD2L1 | SLC7A2 | ANKRD17 | CHORDC1 |
| MAGI1 | KIAA1456 | AFAP1L1 | CHSY1 |
| MAK | ORAI2 | HSPBAP1 | CREBZF |
| MAML3 | SLC38A2 | ANKRD20A4 | CXCL12 |
| MAP10 | REEP5 | KCNG4 | CYP1B1 |
| MAP3K13 | TRIB1 | ZNF79 | ESR1 |
| MAP3K2 | BMT2 | ASTN1 | FAM35A |
| MAP3K21 | ZEB1 | ATP6V1A | FBXO28 |
| MAP3K9 | THRB | ZWILCH | FMR1 |
| MAPK1 | UGT2A2 | DGKI | FNDC3A |
| MARK1 | B3GALNT2 | DHX33 | GALNT3 |
| MBNL1 | FOS | ADAMTS18 | HECTD2 |
| MED13 | LBR | APC | HMBOX1 |
| MED14OS | CPEB1 | APEX2 | HNRNPD |
| METAP1 | MRGBP | ICA1L | ARF4 |
| MFSD6 | LMNB1 | ATP8A1 | ARNT |
| MGAT4A | TGOLN2 | KPNA1 | BRWD1 |
| MIGA1 | SLC30A5 | ATPAF1 | LYPLA1 |
| MKL2 | ATM | NPAT | MAPK10 |
| MLEC | RNF111 | GREB1 | MAT2A |
| MMGT1 | CAPZB | GRM6 | MIDN |
| MNX1 | MET | KATNBL1 | MYBL1 |
| MOB4 | ZNF480 | DKK1 | MYLIP |
| MON2 | FAM69A | DKFZP667F0711 | NAP1L1 |
| MORN4 | ZNF490 | DKFZP779L1853 | PAFAH1B2 |
| MPHOSPH9 | NUPL2 | AC012215.1 | PAIP2 |
| MPPE1 | SLC39A6 | CSN1S1 | PAK1 |
| MRGBP | FRS2 | ATXN7 | PCDHA1 |
| MRPL42 | MBNL2 | ATXN7L1 | PCDHA11 |
| MTCL1 | NR2F6 | AVPI1 | PCDHA12 |
| MTMR2 | PRKAA1 | ACTR2 | PCDHA13 |
| MTMR4 | EYA1 | AC079354.2 | PCDHA2 |
| MTSS1L | UBE2B | KRTAP10-7 | PCDHA3 |
| MTX3 | ZBTB21 | AXL | PCDHA4 |
| MYCN | FAM84B | B3GALNT1 | PCDHA5 |
| MYRIP | RREB1 | ATP2B2 | PCDHA6 |
| N4BP1 | TNRC18P2 | CXCL11 | PCDHA7 |
| N4BP2 | EED | DLG3 | PCDHAC1 |
| NAA15 | CFTR | ARL14EP | PCDHAC2 |
| NACA | ANKFY1 | CCDC85A | PIK3R1 |
| BICD2 | ARAP2 | ARL4D | PPP6C |
| BICRA | GCLC | ARL5B | INSIG1 |
| BTBD3 | FOXP4 | ANKRD33B | KDSR |
| BTLA | QSER1 | ATP6V0E1 | KIAA1841 |
| C11orf70 | PAK3 | CPSF4L | INA |
| C16orf72 | SREBF2 | CSGALNACT2 | CDC27 |
| C1orf52 | ELAVL2 | ACKR3 | CFTR |
| C2orf88 | DDX19B | GRIA4 | CNEP1R1 |
| C3orf58 | ZNF792 | ATP7A | CYB5A |
| C7orf73 | SSFA2 | ATXN1 | DENND5B |
| C8orf76 | E2F3 | ANKRD50 | ECT2 |
| C9orf72 | KLHL23 | DAGLA | EPB41L3 |
| CACNB2 | KIAA1586 | DAG1 | F3 |
| CADM1 | SEPT11 | ARNTL | FGF2 |
| CADM2 | SNRNP27 | ARPC2 | FOXO1 |
| CAMKK1 | NKAP | DAOA | FOXO3 |
| CAMTA1 | SNHG1 | KCNK10 | HSP90B1 |
| CAPN2 | CNEP1R1 | GREM2 | IGF1R |
| CAPS2 | MSH2 | KB-1507C5.2 | IL6ST |
| CASP3 | CD81 | ZNF99 | LMO2 |
| CAV3 | LYSMD3 | KIAA1737 | ACSL3 |
| CBFA2T2 | LRCH2 | KIAA1841 | AK2 |
| CCDC126 | SHMT1 | ARSB | POLR3G |
| CCDC68 | SMARCD1 | DCAF12L2 | PRDM1 |
| CCDC88A | SACM1L | ARSK | PTBP2 |
| CCNJ | ZDHHC15 | KIAA2026 | RAB8B |
| CCNT2 | PIP4K2A | DCAF5 | RASA1 |
| CCSER1 | BCL9 | BET1 | RHOB |
| HNRNPA0 | NT5C3A | GALNT11 | SEPT2 |
| HNRNPF | FAM103A1 | ALDOB | ZNF365 |
| HSPE1-MOB4 | CDC7 | BEX2 | NLRP3 |
| HTRA3 | RIOK2 | AP3S1 | NUP210 |
| ICK | MMS22L | BHLHB9 | PHF19 |
| IFFO2 | CCND1 | ACVR2A |  |
| IKZF2 | PTK2 | ALG2 |  |
| IKZF4 | KIAA1841 | CD2AP |  |
| IL1R1 | MED21 | KCND3 |  |
| IMPA1 | UTP18 | AP3B1 |  |
| ING3 | NAPEPLD | KCNE3 |  |
| INO80D | TET3 | ALKBH1 |  |
| INPP4B | ELMO2 | ALPK3 |  |
| INPP5F | DNAJB9 | ACBD3 |  |
| IPO5 | HHLA1 | CPSF4 |  |
| IQGAP3 | CLPB | ADAMTS1 |  |
| JAK2 | MMP14 | ADAMTS13 |  |
| JAKMIP2 | NF1 | ADAMTS15 |  |
| JDP2 | EBAG9 | AMDHD2 |  |
| KAT6B | WDR82 | ETS1 |  |
| CPEB2 | PRAP1 | DDHD1 |  |
| CPEB3 | SVOP | DACT3 |  |
| CREBRF | ZMAT3 | DDI2 |  |
| CRISPLD1 | HYOU1 | AMMECR1 |  |
| CRLS1 | ZNF384 | DDIT4L |  |
| CSNK1G3 | KDSR | DDO |  |
| CTCF | CTC1 | DDX10 |  |
| CTDSPL | C6orf47 | AMPH |  |
| CTNND2 | SMIM14 | DAXX |  |
| CTTNBP2 | DCTN5 | ARPC5 |  |
| CYB561D2 | LPCAT1 | AL360004.1 |  |
| DAG1 | STARD7 | KBTBD11 |  |
| DCAF5 | RTKN | ATM |  |
| DCAF7 | MAPK8 | ATP11B |  |
| DCBLD2 | STIP1 | ALDH1B1 |  |
| DCLRE1B | C5orf22 | ATP1A3 |  |
| DCUN1D1 | KRAS | ANGPTL5 |  |
| DDIT4 | IRF1 | ANK2 |  |
| DDX3X | UBFD1 | NDUFV3 |  |
| TRPC4 | TNFRSF1B | ATP1B4 |  |
| TSHZ3 | YWHAZ | ABCA9 |  |
| TULP4 | IGFBP5 | ATP1B1 |  |
| DESI2 | MRRF | NEDD1 |  |
| DIP2B | DCAF7 | GALNT15 |  |
| DIP2C | BAZ2A | ATP2B1 |  |
| DISC1 | MAP3K3 | ANKRA2 |  |
| DLG5 | DDAH1 | CBLL1 |  |
| DMXL2 | SYNRG | ANKRD11 |  |
| DNM1L | TLN1 | AEBP2 |  |
| DNMT3A | SENP5 | AEN |  |
| DOT1L | TMPPE | ANKRD20A1 |  |
| DPY19L2 | E2F6 | ATP5G2 |  |
| DR1 | YY1 | HSPB3 |  |
| DSC1 | RAB27B | ALS2 |  |
| DTD2 | NAGPA | CR1 |  |
| DUSP1 | NQO2 | ARHGAP29 |  |
| EDEM3 | CIAO1 | DGKB |  |
| EED | MSANTD2 | DHX36 |  |
| ELOC | PTEN | DDHD2 |  |
| EMP1 | PHLDA2 | ATP8B4 |  |
| EMP2 | TGFB2 | IARS2 |  |
| ENPP2 | ARMC1 | ANKRD6 |  |
| ENY2 | RBL1 | CDKN2AIP |  |
| EPB41L5 | PGM3 | ATP9A |  |
| ERBIN | ASB3 | ICK |  |
| ERO1B | PMAIP1 | ANAPC13 |  |
| ETNK1 | KLHL42 | DDX21 |  |
| ETV5 | ALKBH5 | KIAA1671 |  |
| EVI5 | LAMC1 | ADAT1 |  |
| ZFHX4 | SLC10A6 | DIO2 |  |
| ZFP36L2 | CACFD1 | ATXN3L |  |
| ZIC1 | GXYLT1 | DISC1 |  |
| ZIK1 | ACTN4 | ATXN3 |  |
| ZMAT3 | TCF7L2 | DIS3 |  |
| ZNF207 | TSKU | CDYL |  |
| ZNF217 | RPS21 | ATXN7L3 |  |
| ZNF235 | PLEKHA2 | AP2B1 |  |
| ZNF24 | ABI2 | ATXN7L3B |  |
| ZNF260 | RNF146 | AUTS2 |  |
| ZNF33A | CPSF2 | AC090186.1 |  |
| ZNF385B | LYRM2 | AP3M1 |  |
| ZNF451 | HMGB1 | AP3D1 |  |
| ZNF469 | PARP15 | B3GALNT2 |  |
| ZNF510 | RGMA | AC119673.1 |  |
| ZNF518A | RAB32 | FBXW11 |  |
| ZNF532 | MEX3D | NIPAL1 |  |
| ZNF549 | IRF2BPL | NIPSNAP1 |  |
| ZNF557 | ERBB2 | NIPSNAP3B |  |
| BCL2L11 | FAM221B | NIT2 |  |
| BCL9 | MDH2 | NKAIN1 |  |
| BDP1 | SPRTN | CPE |  |
| BEAN1 | PLAU | APBA1 |  |
| BEGAIN | BUB1 | APBA2 |  |
| FLRT3 | PPIF | B3GAT3 |  |
| KDM3B | AURKA | ABCG2 |  |
| KDM6B | TIGAR | ACER3 |  |
| KHDRBS2 | LSM14B | LARP1 |  |
| UTS2B | COQ7 | KIAA1467 |  |
| VSX1 | C6orf106 | DHRSX |  |
| WDR72 | OPHN1 | DNAH10OS |  |
| WWC3 | LYPLA1 | ZNF800 |  |
| KAT7 | TUT1 | ZNF804B |  |
| KBTBD8 | TYMS | ZNF805 |  |
| KCNA1 | SELENON | ZNF83 |  |
| KCNE1 | GDF11 | ZNF831 |  |
| KCNH7 | SHISA9 | AFTPH |  |
| KCTD6 | CDK12 | EXOC1 |  |
| DENND1B | RNASEH1 | HECTD3 |  |
| DENND2C | CEP89 | ZNF85 |  |
| DENR | E2F1 | DNAJC10 |  |
| KIAA1462 | SEMA3D | AGFG1 |  |
| KIAA1586 | PPP2R5C | CGRRF1 |  |
| KIF2A | AK2 | ZNFX1 |  |
| KIF5B | C1QBP | DNAJC16 |  |
| KLF2 | LYST | AR |  |
| KLF3 | TAPT1 | ARAP2 |  |
| KLF6 | CCDC8 | AGO1 |  |
| KLHDC1 | KMT2A | AGO2 |  |
| KPNB1 | TMEM30A | AGO3 |  |
| KTI12 | ERBB4 | ZRSR1 |  |
| ZC3H7A | RPS6KB2 | IL24 |  |
| ZCCHC2 | SNRPB2 | AHRR |  |
| ZDHHC21 | MAP3K1 | ALG14 |  |
| LCOR | SLC7A5 | ZSCAN21 |  |
| LGI2 | PTPN9 | ZSCAN22 |  |
| LHFP | TRAFD1 | CD300E |  |
| LIN7C | SRSF2 | ALG8 |  |
| LMNB1 | ZNF675 | ALG9 |  |
| LONRF1 | ATAD5 | ARHGAP18 |  |
| LRAT | KLF10 | AHSA2 |  |
| LRCH1 | C11orf74 | NUDT4 |  |
| ZSWIM6 | STAR | ALS2CR11 |  |
| FLRT2 | ZMAT4 | DCUN1D1 |  |
| ZFAND3 | CKS2 | AMBN |  |
| XPO5 | ZNF138 | AMD1 |  |
| LANCL3 | ZNF90 | ARHGAP24 |  |
| ZEB1 | APOL6 | AK3 |  |
| XKR6 | SRXN1 | AK4 |  |
| ZBED4 | WNT16 | LIMA1 |  |
| ZBTB21 | MDM4 | ARHGAP31 |  |
| ZBTB34 | FOXC1 | DPF2 |  |
| ZNF654 | ALDOA | ARHGAP4 |  |
| ZNF746 | METTL7A | AKAP2 |  |
| DDX43 | ZNF117 | ARHGAP5 |  |
| DKK2 | CYP4F11 | AACS |  |
| DEF8 | GRHL1 | DNAJA1 |  |
| HOXD13 | HHIP | EXOC2 |  |
| DNAAF4 | UHMK1 | DPY19L1 |  |
| DCTN5 | YAE1D1 | ATL3 |  |
| DHRS7B | ZNF639 | ANGPT4 |  |
| IDE | IL12RB2 | ATOH7 |  |
| IL17RD | ZNF254 | ABAT |  |
| F9 | PLEKHG7 | ABCA10 |  |
| ETV6 | SOX17 | ABCA12 |  |
| FAT4 | GRPEL2 | ANKFY1 |  |
| FCHSD2 | CLIP1 | ANKH |  |
| FGFR1OP | RGL2 | ABCA6 |  |
| FHDC1 | THAP5 | ABCB10 |  |
| FLI1 | OTX1 | ATP2A2 |  |
| FUNDC2 | SKIL | ANKLE2 |  |
| ETV1 | PMEPA1 | ANKMY2 |  |
| GNAO1 | SOD2 | ATP2B3 |  |
| GPR20 | PPA1 | ABCC8 |  |
| GRB7 | FCHO2 | ANKRD12 |  |
| FAM168B | XBP1P1 | DEPDC1B |  |
| FAM84A | CS | AF131215.5 |  |
| HEG1 | COBLL1 | ANKRD20A2 |  |
| HHAT | CEBPG | ARHGAP23 |  |
| HNRNPUL2 | IGF2BP1 | ANKRD28 |  |
| NAV3 | ZNF99 | ATP6AP2 |  |
| NECAP2 | BICRA | CDK18 |  |
| NEDD9 | OLA1 | DGCR6 |  |
| ING1 | CDCA4 | ANAPC1 |  |
| ING5 | WWTR1 | DGKH |  |
| IRF1 | SLC5A8 | ANKRD45 |  |
| DNAJC13 | KCNK5 | CDKAL1 |  |
| DUSP7 | CCL5 | CSE1L |  |
| DYRK2 | BCL2L13 | HYPK |  |
| E2F6 | POR | ANGPTL1 |  |
| EARS2 | JAG2 | ANGPTL2 |  |
| EIF2AK1 | ELFN2 | DIAPH1 |  |
| EN2 | C9orf3 | DIAPH2 |  |
| ENAM | GNAL | KPNA4 |  |
| EPHA5 | TNFSF15 | ARHGEF12 |  |
| ERBB4 | PPM1L | DNAJA4 |  |
| PHF6 | BCAM | ANO6 |  |
| PHLDA2 | FZR1 | AC008964.1 |  |
| PIAS2 | CLEC2D | ATXN1L |  |
| PIGA | ASF1B | AC011897.1 |  |
| PLAU | AMER1 | LRCH3 |  |
| PLEKHA2 | PARS2 | CWF19L2 |  |
| PLXNC1 | RASSF4 | AC020907.1 |  |
| PMAIP1 | SH3BP4 | CEBPG |  |
| PREB | RETSAT | AC079210.1 |  |
| PRR14L | LUZP1 | AP1S2 |  |
| PSMA5 | C9orf78 | NIN |  |
| PTPN21 | FLOT1 | IYD |  |
| GDF11 | LTF | NIPA1 |  |
| RAB27B | TMEM189-UBE2V1 | AP4B1 |  |
| RAB6B | HEYL | NIPA2 |  |
| RALGAPB | FAM49B | CXADR |  |
| HACD2 | TMED9 | C9orf153 |  |
| HACE1 | GSR | C9orf156 |  |
| MYO18B | CPD | CXCL12 |  |
| NAGA | TMEM189 | CXCL5 |  |
| DAAM2 | PSMD10 | C9orf47 |  |
| REST | CDK6 | ACAP2 |  |
| RGMA | MTHFD2 | ASTE1 |  |
| RNF222 | HSP90AB1 | ACBD5 |  |
| RPGR | MIEF2 | B3GNT5 |  |
| RPL27A | CNIH1 | APBB2 |  |
| RRAS2 | KIF1B | ACER2 |  |
| RTN4IP1 | LZTS1 | JPH3 |  |
| RUNX1T1 | SCN4B | JUNB |  |
| SCN1A | NCOA3 | ACLY |  |
| SCP2 | MPDU1 | ACN9 |  |
| SCYL3 | TP53 | ACO1 |  |
| SELENON | SRD5A3 | ACOT13 |  |
| SEPT8 | FLOT2 | CXXC4 |  |
| SETD7 | NCAN | CXXC5 |  |
| SIAH1 | BCL2L2 | ACP2 |  |
| SIRT7 | AGO2 | ACPL2 |  |
| SLC10A6 | LRTM2 | AAK1 |  |
| SLC16A6 | CRKL | KATNAL1 |  |
| SLC39A5 | C16orf58 | CYB5D1 |  |
| SLC9A7 | KLHL15 | CYB5R4 |  |
| SNX27 | BAX | CYBB |  |
| SON | AP3B1 | CYCS |  |
| SOS2 | TFAP2A | ANO4 |  |
| SP4 | SUFU | KBTBD3 |  |
| SPATA13 | C17orf49 | CYFIP1 |  |
| SRSF6 | CALU | KBTBD8 |  |
| ST6GALNAC5 | KMT2B | CYLD |  |
| STKLD1 | RAB1B | CALCRL |  |
| PXYLP1 | TRAF1 | CYP19A1 |  |
| STX16 | HOMER2 | CYP20A1 |  |
| STX5 | AP2B1 | GSN |  |
| SVEP1 | ERGIC2 | KCNAB2 |  |
| SYF2 | CASKIN1 | KCNB1 |  |
| TAOK1 | MAP2K3 | KCNB2 |  |
| TAPT1 | PAPPA | KCNC1 |  |
| TBL1XR1 | ADRA2B | CSRP2 |  |
| TCF4 | MAPK1 | PAK7 |  |
| TGFB2 | NDUFAF3 | ZSCAN31 |  |
| TIMM8B | TAF1D | CYR61 |  |
| TMEM30A | TNFSF9 | AP3M2 |  |
| TMEM41B | SLIT3 | CAMSAP2 |  |
| TMPPE | FAM109A | CR2 |  |
| TNFAIP1 | PPARGC1B | ADAMTS10 |  |
| TNFRSF21 | KCTD15 | APBB1IP |  |
| IRF2BPL | STX6 | EIF2S1 |  |
| JADE2 | SOCS5 | EIF3A |  |
| JMY | ABCC12 | KCNJ2 |  |
| KCNC2 | SIK1 | EIF3H |  |
| KCNQ5 | SEMA4D | MYRFL |  |
| KIAA0825 | CAPN5 | CAPZA1 |  |
| KIAA1549L | LDLRAD4 | CAPZA2 |  |
| KIT | TWIST1 | KAT7 |  |
| KMT2A | XBP1 | APOBEC4 |  |
| KRAS | SHOC2 | APOF |  |
| LAMC1 | GDPD5 | AL021546.6 |  |
| LAMC2 | PDXK | AASS |  |
| LAMP2 | ZBTB10 | KCNMB2 |  |
| LIMS1 | ZBTB8B | ADD3 |  |
| LLGL1 | POU4F2 | APPBP2 |  |
| LPCAT1 | NRAS | APPL1 |  |
| LRP4 | DKK3 | ABCA5 |  |
| LUZP1 | ALPK2 | ADNP2 |  |
| LYRM2 | ZNF417 | ADRA1A |  |
| MAPK8 | SUGT1 | CRYZ |  |
| MARCKSL1 | PRR14L | ABCB7 |  |
| MARF1 | MINK1 | ADRA2B |  |
| MB21D2 | ARL2 | DENND6A |  |
| MDH2 | ARL8B | ADRBK2 |  |
| MMP19 | PLXNB1 | FEZ1 |  |
| MSANTD2 | VAV2 | DEPDC5 |  |
| MTF1 | TPM3 | AFAP1 |  |
| MTHFD1 | PIGQ | HSPA8 |  |
| MTMR12 | NPTN | CDK14 |  |
| ALOX5 | CDC42SE1 | CDK17 |  |
| ANAPC15 | ERC1 | FABP2 |  |
| AP2M1 | MED23 | AMN1 |  |
| AREL1 | KLF16 | ARHGAP28 |  |
| ARHGEF12 | WSB2 | AFF4 |  |
| ARID3B | PPP6R3 | FADS3 |  |
| NQO2 | UBE2V1 | AGBL2 |  |
| NRIP1 | KMT5A | FAH |  |
| NSF | BTBD19 | CDKL2 |  |
| NT5E | TRAF7 | AGGF1 |  |
| NUP50 | ABCC6 | AGL |  |
| OLFML3 | TWF1 | AGK |  |
| OSMR | NAP1L4 | ATP9B |  |
| P2RX5 | SFMBT2 | APLP2 |  |
| PAK4 | FOXI2 | ATRNL1 |  |
| PDCD2 | SCAMP4 | KIAA0232 |  |
| PER3 | C10orf76 | ANP32B |  |
| PEX13 | PAFAH1B2 | DIP2C |  |
| PHF24 | TFAP2C | ARID2 |  |
| CALB1 | SMDT1 | ARID3B |  |
| CALHM1 | NFIC | ARID4A |  |
| CAPRIN1 | RAB15 | AC021218.2 |  |
| CARF | ZNF641 | CEACAM6 |  |
| CBX7 | BCL2L2-PABPN1 | KIAA0753 |  |
| CCDC28A | FGFR1 | AGRN |  |
| CCND1 | SPEN | DLD |  |
| CD244 | ING4 | AC114546.1 |  |
| CDC42EP2 | CPNE7 | FBXO48 |  |
| CECR2 | VASP | ARL2BP |  |
| CEND1 | AHNAK2 | HIST1H2BH |  |
| CERS1 | TRIM29 | ARL15 |  |
| CLOCK | IPP | CRIPT |  |
| CLTC | GABARAP | ARL5A |  |
| CNBP | GALNT7 | CRISPLD1 |  |
| COMMD9 | CNNM2 | ARL6IP1 |  |
| CSRNP1 | RUBCN | ARL5C |  |
| CSTF1 | WASF2 | ARL6IP5 |  |
| CTDSPL2 | MAPK14 | ACBD7 |  |
| CYP26B1 | PNPLA6 | CRLS1 |  |
| RBMXL1 | FGFR4 | CRMP1 |  |
| ZNF346 | SRGAP1 | KIAA1462 |  |
| ZNF562 | ARHGAP10 | DAB1 |  |
| ARMC1 | PCGF3 | ABHD3 |  |
| ARPC5 | LAMA4 | ABHD5 |  |
| ATF6 | DOCK9 | DACT1 |  |
| ATF7IP2 | ABLIM3 | CCNG2 |  |
| BEND4 | HDGF | ARMC8 |  |
| BHLHE41 | PGAM4 | ARMCX3 |  |
| BRPF1 | TSEN54 | KIAA1586 |  |
| BTRC | SRGAP2 | AKTIP |  |
| TP53INP1 | WDR81 | AL009178.1 |  |
| TRIM27 | HIGD1A | DAZ4 |  |
| TSC1 | STK10 | DAZAP2 |  |
| TTC26 | PABPN1 | CCP110 |  |
| TTPAL | ENTPD1 | CCPG1 |  |
| TWF1 | CD274 | CCR10 |  |
| TXLNA | MEF2C | CCR6 |  |
| TXNRD2 | BANP | ALCAM |  |
| UBA52 | PHLPP2 | ALDH1A3 |  |
| UBP1 | RLIM | HNF4A |  |
| UPRT | JAG1 | DCAF7 |  |
| USP13 | AHSA1 | DCBLD2 |  |
| USP53 | CPEB4 | DCC |  |
| VASH1 | RAB5B | HNRNPA2B1 |  |
| VN1R1 | UBE2I | HNRNPA3 |  |
| WDR7 | TMEM248 | HNRNPAB |  |
| SRSF2 | MAP2K5 | CCSAP |  |
| WFDC8 | TBPL1 | PEG10 |  |
| XPNPEP1 | RASA1 | ALG6 |  |
| MCL1 | TRPS1 | CYP8B1 |  |
| YIF1B | POLQ | HNRNPLL |  |
| YWHAZ | PCBD2 | HNRNPR |  |
| ZBTB40 | DAPK1 | HNRNPU |  |
| ZBTB5 | ARHGAP19-SLIT1 | BLOC1S5 |  |
| ZBTB6 | YWHAQ | DCTN5 |  |
| ZC3H11A | SLC39A11 | ALX1 |  |
| ZKSCAN8 | ASH1L | ALX4 |  |
| ZNF248 | ZBTB33 | DCUN1D4 |  |
| AIMP2 | ATF4 | ETNK1 |  |
| AJUBA | ZNRF1 | DCUN1D5 |  |
| C5orf22 | INSIG1 | ETS2 |  |
| CAB39 | FSCN1 | ETV1 |  |
| XPR1 | QKI | ETV3 |  |
| ABCB8 | HSPD1 | CDC14A |  |
| ABI2 | NABP2 | ETV7 |  |
| C15orf32 | CDKN1C | EVI5 |  |
| ADARB1 | HECTD2 | EVI5L |  |
| ADCYAP1R1 | CDKN1B | EVX1 |  |
| C11orf44 | BBC3 | EVX2 |  |
| ADGRE5 | SIRT1 | EXD2 |  |
| C19orf53 | MAT2A | CDC42 |  |
| C1QC | CASP3 | CDC42BPA |  |
| WDR82 | C5orf51 | ANGPT2 |  |
| AGPAT1 | ICAM1 | EXOC6B |  |
| RAPGEF6 | RPS7 | ATOH8 |  |
| RBM20 | DIRAS3 | EXOSC1 |  |
| ADRA2B | RACGAP1 | EXOSC2 |  |
| ENKUR | SMCHD1 | EXOSC6 |  |
| FLVCR2 | TMEM245 | ANKDD1B |  |
| ENOPH1 | MIEN1 | ANKEF1 |  |
| EIF1AX | ABLIM1 | NECAP1 |  |
| EIF4ENIF1 | BRWD1 | EYA1 |  |
| ETFDH | OIP5 | ANKIB1 |  |
| EPM2AIP1 | HIST1H2AE | EYS |  |
| HMGB1 | ARIH2 | EZH2 |  |
| HMGCS1 | ZBTB37 | F11R |  |
| FAM102B | CIAPIN1 | F13A1 |  |
| HCFC2 | HIST1H3D | F2 |  |
| FZD1 | CDC25C | ARFIP1 |  |
| GDAP1 | TOB2 | HSPA6 |  |
| FOXC1 | ARF4 | ARG2 |  |
| FOXD1 | USP18 | ANKRD20A3 |  |
| GPHA2 | DDX6 | FAAH |  |
| GXYLT1 | APAF1 | CTNND2 |  |
| GLUL | HMGXB4 | ARHGAP27 |  |
| GOLGA7B | PTBP3 | FABP4 |  |
| EID1 | TBK1 | FABP5 |  |
| KCNS3 | TIMP3 | FADS1 |  |
| KIAA0355 | GRB10 | ARHGAP32 |  |
| KIAA1217 | NME2 | HTR2A |  |
| KLF9 | CREBZF | AGBL3 |  |
| KLHL24 | NT5DC2 | AGER |  |
| KMT2E | HIST1H2AC | FAHD2A |  |
| LIMCH1 | ZNF571 | CDKN1A |  |
| FAM160A1 | CTCF | FAM103A1 |  |
| HAPLN1 | DKK2 | ANKRD63 |  |
| EFR3A | BRAP | ARHGEF11 |  |
| FAM208B | YOD1 | DIDO1 |  |
| FGF13 | RBM39 | ATRN |  |
| RBPJ | WDR34 | DIO1 |  |
| REEP3 | IQCE | ANO3 |  |
| RELL1 | PPP1R14C | ANO5 |  |
| RFTN2 | MDM2 | PIEZO2 |  |
| RGS7BP | PRPS1L1 | DIRC2 |  |
| RIF1 | ASZ1 | ANTXR2 |  |
| RIMKLB | ANXA1 | AGR2 |  |
| FAM199X | SAPCD2 | ADAT2 |  |
| FAM208A | GLYR1 | AC110781.3 |  |
| SDR39U1 | PGPEP1 | DLEC1 |  |
| SEMA3E | PAK1 | KIAA1161 |  |
| SEMA5A | SOCS1 | ARL10 |  |
| SERINC3 | PPP6C | DLEU7 |  |
| HMP19 | CCT3 | DLG2 |  |
| HNRNPR | PXN | CENPF |  |
| HOXA10 | RPS24 | DLG5 |  |
| HPDL | TSPAN13 | DLGAP3 |  |
| HTR2C | EXO5 | ZNF770 |  |
| IFT52 | ESR1 | FAM162A |  |
| IL1RAP | NDUFS1 | ZNF772 |  |
| RUFY2 | PELO | CPEB1 |  |
| SCOC | PHF21A | CPEB2 |  |
| ITCH | PALB2 | APBB1 |  |
| KCNE3 | SNX4 | CPEB4 |  |
| SNTB2 | PEG10 | CPED1 |  |
| SNX4 | NDFIP1 | LARP1B |  |
| SP100 | LGTN | APH1B |  |
| SP3 | XRCC6 | ZNF80 |  |
| SRI | UTP14A | DMXL2 |  |
| SRSF7 | KIT | CEP57L1 |  |
| ST6GALNAC3 | PEX19 | AFG3L2 |  |
| STK17B | KIF16B | AFP |  |
| STK4 | GJA1 | DNAH9 |  |
| STRBP | KPNA6 | APOPT1 |  |
| STX2 | NFYA | APP |  |
| SULT1C2 | SF1 | AGBL4 |  |
| TBC1D32 | SLC25A36 | CFTR |  |
| THAP5 | UHRF1 | AGBL5 |  |
| THBS1 | CPSF6 | SCN2A |  |
| TIAM2 | LIMS1 | DNAJC15 |  |
| TLL2 | FLNA | AGMAT |  |
| TRAF3IP2 | CCDC142 | CHAC2 |  |
| TRIM2 | ZYX | NOP9 |  |
| TTC22 | KLHL8 | NOS1 |  |
| TUBGCP5 | TLE4 | NOS3 |  |
| TXLNB | MDFIC | ZRANB3 |  |
| TXNRD3 | SOCS3 | AGPAT2 |  |
| UBE3A | PNRC2 | IL33 |  |
| HAUS6 | DNAJB14 | ZSCAN2 |  |
| UBQLN2 | EIF2S3 | AGPAT5 |  |
| UBR3 | KLHDC10 | NUDT11 |  |
| SHPRH | CNOT1 | ZSCAN23 |  |
| SIRT5 | NAIP | HNRNPH3 |  |
| SLC39A14 | SELE | ZSCAN30 |  |
| SLC6A6 | NCL | NUDT19 |  |
| SLCO1A2 | ETS1 | DNTT |  |
| SMAD5 | SLC19A3 | DOCK11 |  |
| INSIG2 | BMF | AIDA |  |
| IRF2BP2 | AKT3 | DOCK4 |  |
| DMP1 | CSTF2T | ZSWIM6 |  |
| DNAJC27 | TRAT1 | ZSWIM7 |  |
| ZNF420 | PITPNM1 | AJAP1 |  |
| ZNF440 | AP2A1 | ARHGAP26 |  |
| ZNF493 | SEPHS1 | IMPAD1 |  |
| ZNF675 | MKKS | AKAP1 |  |
| ZNF763 | RNF4 | AKAP10 |  |
| ZNF770 | VANGL1 | DPEP1 |  |
| ZNF85 | ADAMTS6 | INHBB |  |
| ZNF91 | NFYC | ARHGAP42 |  |
| ZSCAN32 | HOXB5 | INO80 |  |
| LMO7DN | PKM | LIN28B |  |
| LMTK2 | LAMTOR5 | AKAP8 |  |
| LOC389831 | BNIP3L | ARHGEF18 |  |
| LRRC57 | FOXO3 | ARHGEF2 |  |
| LRRC8B | MYBL1 | AASDH |  |
| MCC | RPLP0 | DPY30 |  |
| MCUR1 | CORO1A | AL138847.1 |  |
| MFAP3L | ARHGAP42 | ARHGEF38 |  |
| MIA2 | MBD2 | ABCA1 |  |
| MIER3 | EVL | CPM |  |
| MOBP | RB1 | ARHGEF5 |  |
| MSTN | KHSRP | CPNE3 |  |
| NAMPT | MMP2 | IPO5 |  |
| NCF2 | PDIK1L | CPNE4 |  |
| NFIA | MEOX2 | NXT2 |  |
| USP22 | ABHD3 | ALDH6A1 |  |
| VSIG10 | NDUFB5 | IPP |  |
| WNK1 | ZFP30 | ABCC1 |  |
| ZAK | ACIN1 | ABCC5 |  |
| ZBTB41 | PCDHA4 | ABCD2 |  |
| ZC3H12C | ARNT | F2R |  |
| ZNF254 | CROT | ARFIP2 |  |
| ZNF367 | ARL6IP1 | F2RL3 |  |
| ZNF396 | HIST2H2AC | ARHGAP19-SLIT1 | |
| PITPNM3 | USP28 | ABHD17C |  |
| PKP2 | STAT5A | ABHD17B |  |
| PLPP3 | ATP2A2 | CD58 |  |
| PPM1E | CNRIP1 | AMOTL1 |  |
| PPRC1 | PLP2 | DGKE |  |
| PRDM6 | GID8 | ABI3BP |  |
| PRPS2 | EXOC8 | FAF2 |  |
| PTPN4 | CABYR | HTRA3 |  |
| RAB11B | DERL2 | ABRACL |  |
| RAB22A | FICD | ABTB2 |  |
| RAB6C | PSMD4 | ANKRD9 |  |
| RANBP3 | UBC | DICER1 |  |
| BEST3 | ADD1 | DIMT1 |  |
| BTBD1 | DDX3Y | ITGB1 |  |
| BTBD7 | HIST2H3D | ITGA9 |  |
| C8orf34 | SPAG5 | AC010336.1 |  |
| CABP5 | SGTA | ORC3 |  |
| CD2BP2 | RAB5C | ORC4 |  |
| CD36 | GALNT3 | ANXA11 |  |
| CDC73 | VPS53 | AC026310.1 |  |
| CHD7 | ADGRL2 | AC068987.1 |  |
| CHL1 | GPR107 | CECR2 |  |
| CKAP2 | DYRK3 | AC104472.1 |  |
| CNR1 | ASXL2 | DLEU1 |  |
| COPS8 | FAM84A | NIP7 |  |
| CRIM1 | RUNDC3B | IWS1 |  |
| CRLF3 | RAD51 | OSTN |  |
| CST9 | ACTB | ANKRD13B |  |
| CTSV | PLOD2 | ANKRD18B |  |
| CWH43 | MGMT | CXCR4 |  |
| CYP4F3 | ZNF275 | CXCR6 |  |
| DCAF10 | CCSAP | ANKRD22 |  |
| DICER1 | BRD1 | CPS1 |  |
| DLK2 | AGAP1 | ACE2 |  |
| PATJ | FBXO28 | GRHL1 |  |
| SMC5 | EIF4G3 | OXSM |  |
| ADI1 | UQCR10 | CXorf23 |  |
| ADNP2 | STN1 | CXorf61 |  |
| AFF3 | HECTD1 | GRID2 |  |
| AHR | FUS | FAM98A |  |
| ALX4 | FAM35A | GRIK2 |  |
| AMMECR1 | FBXL18 | ANKRD46 |  |
| ARC | LRP6 | ACP1 |  |
| ARHGEF38 | RAB1A | GRIN3A |  |
| ARL13B | POU3F2 | CYB561D2 |  |
| ARL5B | CXCL12 | CYB5B |  |
| ATAD2 | TMED7 | ANKS1A |  |
| ATG10 | ANKRD28 | ANKZF1 |  |
| ATL3 | B4GALT2 | CADM2 |  |
| ATP6V0A2 | HSPA1B | GRPEL2 |  |
| ATP8B4 | UBE2N | GPR126 |  |
| NPHP3 | RBMS2 | ANTXR1 |  |
| NR4A1 | ACSL3 | ANXA10 |  |
| NRBF2 | NUF2 | PAG1 |  |
| NSD2 | FARSA | ANXA4 |  |
| ORC2 | CHCHD2 | GPR155 |  |
| OTUD6B | MYLIP | CSRNP2 |  |
| PAFAH1B1 | RNPS1 | AP005482.1 |  |
| UBFD1 | ATL2 | AP1AR |  |
| UBQLN1 | ZKSCAN8 | AP1G1 |  |
| PAX6 | SSX2IP | GPR176 |  |
| ATP2B4 | ZBTB5 | GTF2A2 |  |
| ATP6V1E1 | TNKS2 | CYSLTR2 |  |
| ARL2 | PIK3R1 | CYTH2 |  |
| ASXL1 | RPL15 | KCNE4 |  |
| ATG12 | BECN1 | CYTH3 |  |
| ARVCF | FBN3 | AP4S1 |  |
| ATG4B | SPRYD3 | EIF2B2 |  |
| ATP2A3 | ZNF805 | EIF2B3 |  |
| ALPK2 | TMCC1 | EIF2B5 |  |
| ATG13 | BAG3 | KCNIP4 |  |
| AMER1 | ZFP1 | KCNJ1 |  |
| AMMECR1L | TRAF4 | MYPN |  |
| ANKRD33B | YWHAE | GPRIN3 |  |
| ANKRD40 | SRP68 | GRAMD1C |  |
| ANKRD6 | RBM33 | GRAMD4 |  |
| ANKS1A | TUB | MYT1L |  |
| ANKS4B | ZNF652 | APOBEC3F |  |
| ANO6 | SLC30A7 | EIF5 |  |
| APOBEC2 | RBM6 | ADCYAP1 |  |
| ARHGAP17 | EIF2AK1 | ADCYAP1R1 |  |
| ARHGAP18 | TNIP1 | ADD2 |  |
| ARHGAP31 | COG5 | APOOL |  |
| ARHGAP35 | ACTG1 | VPS26A |  |
| ARHGAP42 | HMBOX1 | NAA35 |  |
| ARHGAP6 | PSMB5 | ADK |  |
| CAND1 | PANK3 | ADNP |  |
| CAPN11 | MFN2 | APTX |  |
| CAPN5 | HNRNPA0 | ADO |  |
| CARTPT | UBE2J1 | AQP7 |  |
| CASR | HIVEP1 | ADRA2A |  |
| CBL | EEF1A1 | AQR |  |
| CBR4 | NOP58 | ADRB1 |  |
| CBX2 | TUBA1C | KCTD2 |  |
| CCDC149 | TMEM2 | HDAC2 |  |
| CCDC42 | PCDHA7 | F2RL1 |  |
| CCND2 | WEE1 | HDAC8 |  |
| ATP8B2 | PCDHAC1 | F9 |  |
| BAHD1 | LYSMD1 | HSPA9 |  |
| BCL11A | DYNC1H1 | AFF1 |  |
| BHLHE40 | TIAM1 | AFF2 |  |
| BSDC1 | CHORDC1 | AFF3 |  |
| BTG2 | PCDHA13 | CREB1 |  |
| C10orf76 | PAIP2 | AGAP9 |  |
| C11orf57 | PCDHA5 | KDR |  |
| C14orf105 | PDGFA | KDSR |  |
| C16orf70 | C15orf40 | KERA |  |
| C17orf49 | SOX11 | KHDRBS2 |  |
| C1orf226 | BNIP3 | KHDRBS3 |  |
| C1QTNF5 | CLIC1 | ANKS1B |  |
| C1RL | TNFSF10 | ANGPTL7 |  |
| C3orf18 | TOMM20 | AGO4 |  |
| C5orf63 | TMEM132B | AGPAT3 |  |
| C6orf222 | CENPT | AGPAT4 |  |
| CACFD1 | ZNF35 | CSNK1A1 |  |
| CACNB1 | LYN | ARHGEF9 |  |
| CAMK2A | SLC10A7 | ARID1A |  |
| ABCB10 | HOXC10 | ITPRIP |  |
| ABHD2 | SERPINH1 | FAM134B |  |
| ABLIM1 | CXorf38 | FBXO47 |  |
| ABR | MIDN | KIAA1024L |  |
| ACAP3 | C19orf12 | ARL13B |  |
| ACER3 | RPL21 | KIAA1143 |  |
| ACLY | POGZ | CCDC80 |  |
| ACPP | RNF20 | HIST1H3B |  |
| ACSL1 | ZNF236 | CRISP1 |  |
| ACVR1B | SKI | KIAA1210 |  |
| ACVRL1 | TIPARP | CRISPLD2 |  |
| ADAM11 | TMEM183A | CRK |  |
| ADGRA1 | CDC27 | CRLF3 |  |
| ADGRF2 | YWHAB | ABCF1 |  |
| ADGRG3 | SMC2 | CCNB1IP1 |  |
| ADGRL1 | DHX15 | ARL6IP6 |  |
| ADORA2A | PCDHA6 | ARL8B |  |
| ADRA1D | ARHGEF18 | ARMC1 |  |
| AFMID | DVL2 | ARMC10 |  |
| AGO1 | ADAM1A | ARMC2 |  |
| AHCYL2 | RECK | ARMC3 |  |
| AHDC1 | WDR61 | HM13 |  |
| ALS2 | TMEM64 | HMBOX1 |  |
| AKAP13 | DENR | DALRD3 |  |
| XBP1 | CAMKK1 | HMGB1 |  |
| YAF2 | PHF12 | AL078585.1 |  |
| ZBTB10 | MKI67 | AL162407.1 |  |
| ZBTB20 | SF3B3 | AL354993.1 |  |
| ZCCHC11 | AMOT | AL357673.1 |  |
| ZCCHC3 | POLG | AL590452.1 |  |
| ZFYVE28 | NHSL1 | ALAS1 |  |
| ZHX3 | SLC6A9 | HMGXB4 |  |
| ZKSCAN3 | APOL2 | ALDH18A1 |  |
| ZNF12 | TDRP | ALDH1A2 |  |
| ZNF2 | GTF2E1 | DCAF4L1 |  |
| ZNF212 | PCDHA1 | KIAA2022 |  |
| ZNF214 | UNC13B | HELZ |  |
| ZNF322 | ASXL3 | KIDINS220 |  |
| ZNF395 | PCDHA10 | KIF11 |  |
| ZNF445 | PTPRF | KIF13A |  |
| ZNF48 | SPTSSA | KIF14 |  |
| ZNF609 | KLF9 | KIF16B |  |
| ZNF641 | LHFPL2 | HERC2 |  |
| ZNF710 | NR2C2AP | CCSER1 |  |
| ZNFX1 | NUP205 | CCSER2 |  |
| ZNRF1 | HNRNPD | KIF20A |  |
| ZNRF2 | NUP210 | KIF21A |  |
| ATP6V0E2 | PEX1 | KIF26B |  |
| DGKG | PCDHA8 | HESX1 |  |
| DHRS12 | MAP3K2 | HOMER1 |  |
| DHRS4L1 | TICAM1 | KIF3A |  |
| DIAPH1 | TCEAL1 | DCTN6 |  |
| DLGAP1 | CHSY1 | HOXA2 |  |
| DMWD | TRIM28 | ETFDH |  |
| DNAJB5 | PCDHA2 | HOXA9 |  |
| DNM1 | ATP6V1E1 | KIN |  |
| DOCK9 | TRPC3 | CD84 |  |
| DOK4 | GCN1 | CD86 |  |
| DOLPP1 | MAPK10 | ETV5 |  |
| DPYSL5 | PCDHAC2 | CD99L2 |  |
| DSTYK | GATAD2B | PRR24 |  |
| DUSP15 | PCDHA3 | CDC20B |  |
| DVL1 | PCDHA12 | CDC25A |  |
| ECEL1 | PCDHA11 | CDC25C |  |
| EDF1 | LDHB | DDX20 |  |
| EGFLAM | CYP1B1 | KLF15 |  |
| EIF3B | MSMO1 | KLF2 |  |
| EIF5AL1 | ZNF365 | EXOC5 |  |
| ELFN2 | SNX24 | HIST1H3H |  |
| ELMSAN1 | RRAS2 | EXOC8 |  |
| EMCN | SLC12A7 | ANGPTL3 |  |
| ENDOD1 | RANGAP1 | KLF8 |  |
| EPC1 | CHMP2B | DDX52 |  |
| ERC1 | IGF1R | DDX53 |  |
| ERC2 | SP1 | EXOSC7 |  |
| ESPNL | SECISBP2L | EXPH5 |  |
| ESYT2 | NAMPT | EXTL3 |  |
| EXOC2 | CACNG8 | DEF8 |  |
| EZH1 | CDS1 | EYA4 |  |
| F2R | PRRC2C | DENND2C |  |
| F8 | BAG2 | DENND2D |  |
| FAF2 | FOXO1 | DENND3 |  |
| FAM133B | RHOB | DENND5B |  |
| FAM156A | HEXIM1 | CDH7 |  |
| FAM156B | WASL | KCTD21 |  |
| FAM163A | LATS2 | DEPDC1 |  |
| FAM189A1 | IL6ST | KCTD4 |  |
| FAM19A4 | NOVA2 | KCTD9 |  |
| FAM20B | ITGB1 | FA2H |  |
| FAM210A | CARM1 | CDK13 |  |
| FAM234B | HAX1 | KLHL9 |  |
| FAM49B | LMO2 | CD59 |  |
| FAM53C | F3 | CD69 |  |
| FBLN5 | TAL1 | HSPE1-MOB4 |  |
| FBXO17 | ECT2 | FADS2 |  |
| FBXO32 | C9orf40 | KLRD1 |  |
| FEM1A | CDK2 | HTR3C |  |
| FERMT3 | ZBTB18 | KLRG1 |  |
| FGF14 | POTEG | CDKL1 |  |
| FGFR1 | ZNF460 | ANKRD52 |  |
| FHL1 | GPATCH8 | HUWE1 |  |
| FIBCD1 | SPPL2A | HVCN1 |  |
| FLOT2 | PDZD8 | CDC42EP4 |  |
| FLYWCH1 | PHF19 | ANO1 |  |
| FNDC5 | TMEM67 | ICMT |  |
| FOXJ3 | PARP1 | ICOS |  |
| FOXO4 | SINHCAF | DIP2B |  |
| FOXP2 | ABCB1 | PIFO |  |
| FSD2 | PAX4 | IDH2 |  |
| GABARAP | POLR3G | IDH3B |  |
| GALNT10 | MTRF1L | IDNK |  |
| GALNT18 | SYNGR2 | CDYL2 |  |
| GALNT7 | PAX6 | FAM13B |  |
| GAN | FABP7 | ARID5B |  |
| GFI1B | SCARB1 | CECR6 |  |
| GHITM | NSUN3 | FAM150B |  |
| GIGYF1 | STAT1 | FAM151B |  |
| GINS3 | POTEM | FAM155A |  |
| GIT1 | ARTN | FAM160B1 |  |
| GLOD5 | TRPV2 | FAM161A |  |
| GLYCTK | PTBP2 | FAM161B |  |
| GNAQ | NMNAT2 | CENPW |  |
| GON4L | NFIA | NKAIN2 |  |
| GOSR2 | RIF1 | NKAP |  |
| GPAT4 | CYB5A | NKAPL |  |
| GPATCH1 | CHUK | CPEB3 |  |
| GPD1L | SESN3 | FAM169B |  |
| GPR17 | SEPT2 | FAM171A1 |  |
| GPSM1 | NFIX | ZNF790 |  |
| GRB10 | LIF | ZNF791 |  |
| GRIN1 | SP3 | ZNF8 |  |
| AHNAK2 | LGALS8 | FAM175A |  |
| CPXM2 | CAPRIN1 | FAM177A1 |  |
| CRACR2A | SLC2A4 | DNAH14 |  |
| CRAT | MYL9 | DNAH3 |  |
| CRB2 | STAT3 | DNAH5 |  |
| CREBL2 | SEMA3A | IKZF3 |  |
| CRKL | NLRP3 | IKZF4 |  |
| CRTC1 | MAFB | DNAJB13 |  |
| CRTC3 | CCL3 | DNAJB14 |  |
| CS | TOX | IL12RB2 |  |
| CSF1 | IL6 | CERS6 |  |
| CTBP1 | CXCL2 | AQP4 |  |
| CTNNB1 | EPB41L3 | IL1R1 |  |
| CTSS |  | CHAMP1 |  |
| CYB5D2 |  | DNAJC2 |  |
| CYTH2 |  | IL20 |  |
| CYTH3 |  | ARGLU1 |  |
| DAGLA |  | ARHGAP1 |  |
| DAPK2 |  | NUDT12 |  |
| DBNDD1 |  | NUDT13 |  |
| DBNDD2 |  | ZSCAN29 |  |
| DCAF1 |  | ARHGAP17 |  |
| DCAKD |  | FAM222B |  |
| DERA |  | ARHGAP19 |  |
| DEUP1 |  | DOCK3 |  |
| DGAT1 |  | ARHGAP22 |  |
| DGCR8 |  | IMMP2L |  |
| DGKD |  | AIMP1 |  |
| TEK |  | AK2 |  |
| TFAP2C |  | IMPACT |  |
| TGFBRAP1 |  | LIG4 |  |
| TGOLN2 |  | ICOSLG |  |
| THRAP3 |  | LIMCH1 |  |
| THSD7A |  | INIP |  |
| TIGD5 |  | LIMS1 |  |
| TIMMDC1 |  | AKAP7 |  |
| TLE3 |  | ARHGEF10 |  |
| TLR5 |  | DPP8 |  |
| TMEM101 |  | AKR7A2 |  |
| TMEM110 |  | INSIG2 |  |
| TMEM127 |  | INTS2 |  |
| TMEM18 |  | DPYSL2 |  |
| TMEM19 |  | AL161915.1 |  |
| TMEM225 |  | IPMK |  |
| TMEM248 |  | ARHGEF6 |  |
| TMEM258 |  | ARHGEF7 |  |
| TMEM263 |  | CPNE8 |  |
| TMEM33 |  | IPO9 |  |
| TMEM43 |  | ARID1B |  |
| TMEM63A |  | ALDH8A1 |  |
| TMEM86A |  | OBFC1 |  |
| TMIE |  | DENND6B |  |
| TMPRSS13 |  | CDHR1 |  |
| TMPRSS6 |  | CDIPT |  |
| TNFAIP8L1 |  | AMER1 |  |
| TOLLIP |  | LNPEP |  |
| TOR1AIP2 |  | ABHD2 |  |
| TPPP |  | ABI2 |  |
| TRA2B |  | ABI1 |  |
| TRABD2B |  | ABL2 |  |
| TRAF1 |  | AMFR |  |
| TRAF3 |  | ABLIM1 |  |
| TRAF5 |  | ANG |  |
| TRAK1 |  | FBLL1 |  |
| TRAPPC3 |  | ABLIM3 |  |
| TRIM29 |  | ABRA |  |
| TRIM44 |  | OR14J1 |  |
| TRIM59 |  | AC002451.1 |  |
| TRIM66 |  | CDC5L |  |
| TRIM7 |  | AC005003.1 |  |
| TRIP10 |  | ORAI2 |  |
| TSKU |  | ORC1 |  |
| TSPAN11 |  | LRCH1 |  |
| TSPAN9 |  | LRCH2 |  |
| TTC38 |  | CHRM2 |  |
| TTC39B |  | ORMDL2 |  |
| TTLL1 |  | ANKDD1A |  |
| TTYH2 |  | CSNK1G1 |  |
| TTYH3 |  | DLC1 |  |
| TUBA4A |  | FBXO42 |  |
| TUBB8 |  | CECR5 |  |
| TUBGCP2 |  | LRPPRC |  |
| TVP23A |  | LRRC1 |  |
| TXN2 |  | LRRC10 |  |
| TYRO3 |  | LRRC15 |  |
| UBE2H |  | LRRC2 |  |
| UBE2O |  | LRRC20 |  |
| UBE2R2 |  | ASPH |  |
| UBL4B |  | PPAPDC1B |  |
| UGGT2 |  | FDFT1 |  |
| UGT2A1 |  | OXNAD1 |  |
| UGT2A2 |  | OXTR |  |
| UHMK1 |  | P2RX2 |  |
| UMOD |  | P2RX3 |  |
| UNC5C |  | ANKRD40 |  |
| USP15 |  | OGFRL1 |  |
| USP19 |  | CSPG5 |  |
| USP20 |  | ANKRD49 |  |
| USP24 |  | KAT6B |  |
| USP3 |  | ANKRD53 |  |
| USP46 |  | CSPP1 |  |
| USP9X |  | ATG7 |  |
| VANGL1 |  | GPR124 |  |
| VANGL2 |  | ATMIN |  |
| VASN |  | ATP10A |  |
| VAV2 |  | OPRL1 |  |
| VDR |  | OR11A1 |  |
| VEZT |  | OR2A4 |  |
| VPS26A |  | OR2B6 |  |
| VWA1 |  | AP000708.1 |  |
| VWA2 |  | CSRNP1 |  |
| WASF2 |  | OR7A17 |  |
| WDR86 |  | CSRNP3 |  |
| WDTC1 |  | CSRP3 |  |
| WNT3 |  | GPR183 |  |
| WWOX |  | GPR20 |  |
| MPP2 |  | GPR22 |  |
| MRPL22 |  | GPR26 |  |
| MRPL37 |  | ATP5C1 |  |
| MTA3 |  | KCNG3 |  |
| MTHFR |  | GPR34 |  |
| MTM1 |  | KCNH1 |  |
| MTMR3 |  | KCNH5 |  |
| MTR |  | KCNH7 |  |
| MYDGF |  | KCNH8 |  |
| MYO18A |  | ATP6V0D1 |  |
| MYO1D |  | ATP6V0E2 |  |
| NAA11 |  | EIF3F |  |
| NAA50 |  | KCNJ11 |  |
| NADK |  | CSTF2 |  |
| NAGPA |  | CSTF2T |  |
| NAP1L4 |  | GRAP2 |  |
| NAT8L |  | GRASP |  |
| NCKAP1 |  | GRB10 |  |
| NCS1 |  | APOL6 |  |
| NDOR1 |  | GRHL2 |  |
| NDUFAF4 |  | GRHPR |  |
| NECAB3 |  | CASD1 |  |
| NECTIN1 |  | CASK |  |
| NEK6 |  | FNDC7 |  |
| NEO1 |  | VPS29 |  |
| NEURL1B |  | APPL2 |  |
| NFATC1 |  | APRT |  |
| NFATC2 |  | KCNQ3 |  |
| NFIC |  | AQP1 |  |
| NIPAL4 |  | ELMO2 |  |
| NMB |  | KCTD12 |  |
| NOL6 |  | KCTD16 |  |
| NOP16 |  | KCTD20 |  |
| NOS1AP |  | KCTD3 |  |
| NOS3 |  | AFAP1L2 |  |
| NPAS3 |  | KDELR1 |  |
| NPTX2 |  | GSPT1 |  |
| NRG4 |  | CREB3L2 |  |
| NRIP2 |  | CREB5 |  |
| NSUN4 |  | CREBRF |  |
| NUAK2 |  | CREBZF |  |
| NUCB1 |  | CREG2 |  |
| NUFIP2 |  | ARHGAP30 |  |
| NUMA1 |  | CREM |  |
| NYAP2 |  | ARHGAP36 |  |
| OXR1 |  | GTF2H5 |  |
| P2RX6 |  | GTF3C3 |  |
| P4HTM |  | PCDHB14 |  |
| PACS2 |  | KIAA0100 |  |
| PAIP2B |  | ARHGEF28 |  |
| PALM2 |  | ARHGEF3 |  |
| PAN2 |  | ARHGEF33 |  |
| PAPD4 |  | ARHGEF39 |  |
| PAPPA |  | KIAA0513 |  |
| PARD3B |  | KIAA0556 |  |
| PARP16 |  | FBXO32 |  |
| PCLO |  | PTP4A1 |  |
| PDE11A |  | ITSN1 |  |
| PDE5A |  | KIAA1024 |  |
| PDLIM5 |  | CTD-2228K2.5 |  |
| PDP2 |  | HIST1H2BF |  |
| PDPK1 |  | CTD-2545M3.6 |  |
| PDRG1 |  | HAPLN1 |  |
| PEBP1 |  | KIAA1191 |  |
| CCNL2 |  | KIAA1199 |  |
| CD247 |  | CTDSPL2 |  |
| CD27 |  | HIST1H4I |  |
| CD276 |  | HIVEP3 |  |
| CD320 |  | KIAA1279 |  |
| CD7 |  | KIAA1328 |  |
| CD84 |  | KIAA1377 |  |
| CDC14A |  | KIAA1430 |  |
| CDC42BPB |  | HLA-DQA2 |  |
| CDC42SE1 |  | HLA-DRB1 |  |
| CDIP1 |  | CCNDBP1 |  |
| CDIPT |  | CCNE2 |  |
| CDK2 |  | HLX |  |
| CDKL1 |  | KIAA1522 |  |
| CELF1 |  | KIAA1524 |  |
| CELSR2 |  | CCNL1 |  |
| CEMIP |  | KIAA1549L |  |
| CEP170B |  | KIAA1551 |  |
| CEP44 |  | HMGB2 |  |
| CEP85 |  | HMGB3 |  |
| CERKL |  | CCR2 |  |
| CHD2 |  | HMGN3 |  |
| CHFR |  | HMGN5 |  |
| CHRD |  | HECW1 |  |
| CIPC |  | HMX2 |  |
| CIT |  | DCAF4 |  |
| CKAP5 |  | HEG1 |  |
| CLASP1 |  | MCOLN3 |  |
| CLDN23 |  | GALNT1 |  |
| CLEC16A |  | MCTS1 |  |
| CLIC5 |  | GALNT13 |  |
| CLSTN1 |  | GALNT14 |  |
| CMTM4 |  | MDGA1 |  |
| CNIH1 |  | GALNT2 |  |
| CNIH4 |  | PTEN |  |
| CNOT2 |  | KIF1B |  |
| CNOT9 |  | KIF1C |  |
| CNTN2 |  | BICD1 |  |
| CNTNAP1 |  | BICD2 |  |
| COA7 |  | BIRC6 |  |
| COL4A5 |  | BIRC7 |  |
| COL8A2 |  | KIF2A |  |
| COLQ |  | GAS2 |  |
| COMT |  | KIF3B |  |
| COPS7B |  | KIF5A |  |
| CPEB1 |  | KIF5B |  |
| CPNE4 |  | KIF6 |  |
| CPSF4 |  | BMP10 |  |
| CPTP |  | KIR2DL1 |  |
| PSD3 |  | HIBCH |  |
| PSD4 |  | KIT |  |
| PSEN1 |  | CD9 |  |
| PSMD10 |  | KL |  |
| PSMD2 |  | KLB |  |
| PTAFR |  | KLC1 |  |
| PTEN |  | HIPK3 |  |
| PTER |  | KLF11 |  |
| PTPA |  | KLF12 |  |
| PTPRT |  | GCC1 |  |
| PUM1 |  | BNIP3 |  |
| PVRIG |  | KLF3 |  |
| PWWP2A |  | GCLC |  |
| PYGB |  | KLF6 |  |
| QKI |  | EXOG |  |
| QSOX2 |  | GCNT1 |  |
| RAB14 |  | KLF9 |  |
| RAB1B |  | KLHDC1 |  |
| RAB4B |  | DDX58 |  |
| RAP1GAP2 |  | DDX6 |  |
| RAPGEFL1 |  | DECR2 |  |
| RASEF |  | KLHL18 |  |
| RASGEF1C |  | DENND2A |  |
| RASSF5 |  | KLHL24 |  |
| RBM34 |  | KLHL28 |  |
| RC3H1 |  | KLHL29 |  |
| RCSD1 |  | KLHL3 |  |
| REEP4 |  | KLHL31 |  |
| REM1 |  | KLHL32 |  |
| RERG |  | KLHL4 |  |
| RETREG2 |  | KLHL42 |  |
| RFWD2 |  | KLHL5 |  |
| RFX7 |  | CDK12 |  |
| RHBDD1 |  | KLHL7 |  |
| RHPN1 |  | GFM2 |  |
| RIMS3 |  | KLK3 |  |
| RMND5B |  | KLLN |  |
| RNASE6 |  | KLRB1 |  |
| RNF111 |  | HSPH1 |  |
| RNF115 |  | GGA3 |  |
| RNF150 |  | KLRF1 |  |
| RNF169 |  | GGPS1 |  |
| RNF216 |  | KMO |  |
| RNFT2 |  | HUNK |  |
| RPH3A |  | KMT2B |  |
| RPIA |  | KMT2E |  |
| RPS6KL1 |  | KNSTRN |  |
| RSAD1 |  | CDC42SE1 |  |
| RTN4RL1 |  | HNRNPH1 |  |
| RUBCN |  | CDC73 |  |
| RUNX3 |  | CDCA7 |  |
| SALL1 |  | HNRNPL |  |
| SAMD10 |  | KPNA5 |  |
| SAR1A |  | KPNA6 |  |
| SARM1 |  | IDE |  |
| SCAMP4 |  | KRAS |  |
| SCN3A |  | KRBOX4 |  |
| SDAD1 |  | KRR1 |  |
| SEC14L4 |  | HOXA10 |  |
| SEC24C |  | IER3IP1 |  |
| SEMA4D |  | CEBPA |  |
| SEMA4F |  | PTHLH |  |
| SEPT4 |  | ITPRIPL2 |  |
| SERF2 |  | KRT80 |  |
| SERINC5 |  | KRTAP10-12 |  |
| SESN1 |  | HOXB8 |  |
| SESN3 |  | CELF2 |  |
| SGCA |  | CELF4 |  |
| SGCB |  | CELF6 |  |
| SGSM1 |  | IFRG15 |  |
| SIDT1 |  | CENPJ |  |
| SIGMAR1 |  | CENPP |  |
| SIN3B |  | L3MBTL3 |  |
| SIT1 |  | CEP112 |  |
| SLAIN2 |  | CEP120 |  |
| SLC12A6 |  | DLX6 |  |
| SLC13A3 |  | NKRF |  |
| SLC18B1 |  | DMD |  |
| SLC1A4 |  | DMGDH |  |
| SLC22A15 |  | APEX1 |  |
| SLC25A25 |  | FAM174A |  |
| SLC25A39 |  | DMRTC1B |  |
| SLC25A42 |  | DMXL1 |  |
| SLC27A1 |  | IGSF11 |  |
| SLC35B4 |  | NMI |  |
| SLC35C2 |  | IKZF2 |  |
| SLC36A1 |  | LCMT2 |  |
| SLC39A3 |  | LCOR |  |
| SLC5A7 |  | IL10 |  |
| SLC6A12 |  | IL10RA |  |
| SLC7A4 |  | IL15 |  |
| SLC7A5 |  | CGGBP1 |  |
| SLC7A6OS |  | SCN3B |  |
| SLC7A8 |  | CH25H |  |
| SLC8A1 |  | LEFTY1 |  |
| SLC9A3R2 |  | LEMD2 |  |
| SLF2 |  | LEO1 |  |
| SLX1A |  | IL1RL1 |  |
| SLX1B |  | NOTCH1 |  |
| SMARCC1 |  | IL20RB |  |
| SMARCC2 |  | CDK19 |  |
| SMOC1 |  | CDK5R1 |  |
| SMYD5 |  | IL5RA |  |
| SNX11 |  | IL6ST |  |
| SNX12 |  | IL7 |  |
| SNX18 |  | NUDT16 |  |
| SNX24 |  | IL8 |  |
| SNX8 |  | ILDR2 |  |
| SOGA1 |  | ILF2 |  |
| SORBS1 |  | ILF3 |  |
| SORCS1 |  | ARHGAP20 |  |
| GRK2 |  | PLEKHH1 |  |
| HAGH |  | MPP5 |  |
| HAMP |  | LHX9 |  |
| HCLS1 |  | LIFR |  |
| HDGF |  | CUL2 |  |
| HDLBP |  | CUL3 |  |
| HIP1 |  | CUL4A |  |
| HMG20A |  | CUL4B |  |
| HMGN4 |  | CUL5 |  |
| HNF1A |  | LIMK2 |  |
| HNRNPA3 |  | C1orf52 |  |
| HNRNPU |  | LIMS2 |  |
| HOXA3 |  | C1orf95 |  |
| HPS4 |  | IFI44L |  |
| HPSE2 |  | LIN52 |  |
| HR |  | LIN54 |  |
| HS2ST1 |  | ARHGEF35 |  |
| HSD17B8 |  | LINC00955 |  |
| HSPA12A |  | ARHGEF4 |  |
| HTR3E |  | LIPT2 |  |
| HTT |  | MRPL30 |  |
| ICOSLG |  | C2orf15 |  |
| IFT46 |  | C2orf73 |  |
| IGSF3 |  | GPCPD1 |  |
| IKBKB |  | GPD1L |  |
| ILDR2 |  | CUX2 |  |
| ING4 |  | LMNB1 |  |
| INO80C |  | GPLD1 |  |
| IPO7 |  | ABCG1 |  |
| IQSEC2 |  | ABHD10 |  |
| ITPK1 |  | ABHD13 |  |
| ITPRIPL2 |  | GPNMB |  |
| ITSN1 |  | MRPS6 |  |
| JAG2 |  | CPOX |  |
| JPH1 |  | ISCA1 |  |
| JUP |  | GPR137B |  |
| KANSL3 |  | OPN3 |  |
| KATNAL1 |  | OR11H6 |  |
| KCNAB2 |  | FAXDC2 |  |
| KCNB1 |  | MSANTD4 |  |
| KCNC4 |  | MSH3 |  |
| KCNJ14 |  | ANGPTL4 |  |
| KCTD15 |  | CSMD3 |  |
| KIAA0930 |  | AC006455.1 |  |
| KIAA1644 |  | MSR1 |  |
| KIF13A |  | LRAT |  |
| KIF16B |  | C6orf62 |  |
| KIF21B |  | FAM227B |  |
| KIF24 |  | LRIF1 |  |
| KIF7 |  | LRIG2 |  |
| KLF13 |  | CSNK1D |  |
| KLHL18 |  | C8A |  |
| KMT2D |  | CSNK1G3 |  |
| KMT5A |  | FAM133A |  |
| KMT5B |  | FBXO36 |  |
| KPNA1 |  | FBXO40 |  |
| KPNA3 |  | GPR85 |  |
| KRT78 |  | C9orf129 |  |
| KRTAP10-11 |  | GPR89B |  |
| KRTAP4-4 |  | GPR89C |  |
| KY |  | GPRASP1 |  |
| KYAT3 |  | GPSM2 |  |
| LAMC3 |  | FAM81A |  |
| LARP1 |  | GREB1L |  |
| LARP1B |  | GREM1 |  |
| LDOC1 |  | MTO1 |  |
| LEPROTL1 |  | GRIA2 |  |
| LETM1 |  | FAM8A1 |  |
| LFNG |  | FAM91A1 |  |
| LGSN |  | FAM96A |  |
| LHX6 |  | FAM98B |  |
| LIMK2 |  | GRIK3 |  |
| LMOD1 |  | CPT1A |  |
| LMX1B |  | GRIN2A |  |
| LPGAT1 |  | GRIN2B |  |
| LRRC38 |  | CCDC59 |  |
| LRRC55 |  | GRM1 |  |
| LSM12 |  | GRM5 |  |
| LTF |  | FAR2 |  |
| LURAP1 |  | CYBRD1 |  |
| LY6E |  | GRPEL1 |  |
| LYNX1 |  | MXD4 |  |
| LZTS1 |  | GRPR |  |
| MAFG |  | GRSF1 |  |
| MAFK |  | GSC2 |  |
| MAK16 |  | GSE1 |  |
| MAP3K4 |  | GSK3B |  |
| MAP4 |  | GSKIP |  |
| MAP6 |  | MYC |  |
| MAP9 |  | CYP27B1 |  |
| MAPK1IP1L |  | CYP27C1 |  |
| MAPRE1 |  | CYP2U1 |  |
| MARCH6 |  | CYP39A1 |  |
| MARVELD1 |  | CYP4F12 |  |
| MASP1 |  | CYP51A1 |  |
| MBLAC2 |  | MYLK3 |  |
| MEAF6 |  | MYLK4 |  |
| MED10 |  | MYNN |  |
| MED19 |  | CAMLG |  |
| MED20 |  | CAMSAP1 |  |
| MEF2C |  | MYO16 |  |
| MESDC2 |  | MYO1E |  |
| MFN2 |  | CYTIP |  |
| MFSD4A |  | MYO6 |  |
| MGLL |  | OSBPL8 |  |
| MICU1 |  | MYOCD |  |
| MIEF1 |  | MYOM3 |  |
| MIEF2 |  | KCNJ10 |  |
| MLLT10 |  | MYOZ2 |  |
| MLXIP |  | MYOZ3 |  |
| MMAB |  | CNNM2 |  |
| MMP24 |  | CNNM3 |  |
| MPDU1 |  | CNNM4 |  |
| SOX8 |  | MYSM1 |  |
| SP7 |  | LRR1 |  |
| SPIRE1 |  | EIF5A2 |  |
| SPPL3 |  | EIF5B |  |
| SPTBN2 |  | APOLD1 |  |
| SRGAP3 |  | VPS13C |  |
| SRM |  | VPS18 |  |
| SRRM3 |  | NAA15 |  |
| SSX2IP |  | NAA25 |  |
| ST6GALNAC1 |  | KCNMB3 |  |
| STAC |  | KCNN3 |  |
| STK32B |  | NAA38 |  |
| SUN2 |  | KCNQ5 |  |
| SUSD6 |  | KCNS1 |  |
| SV2B |  | KCNS2 |  |
| SYS1 |  | NAALADL2 |  |
| TANC2 |  | KCTD14 |  |
| TATDN2 |  | CBL |  |
| TBC1D10B |  | HCN4 |  |
| TBC1D16 |  | NACA2 |  |
| TBC1D24 |  | NACC1 |  |
| TBC1D5 |  | HDAC5 |  |
| TBPL1 |  | NAGA |  |
| TCN2 |  | KCTD5 |  |
| TDRD1 |  | CRBN |  |
| TDRKH |  | NANOGNB |  |
| PPP1R3D |  | KDELC2 |  |
| PELI3 |  | LRRTM4 |  |
| PFKFB4 |  | KDM1B |  |
| PGF |  | KDM3A |  |
| PGGT1B |  | KDM3B |  |
| PGPEP1 |  | KDM5A |  |
| PGRMC2 |  | KDM6A |  |
| PHACTR3 |  | KDM6B |  |
| PHF21A |  | HELT |  |
| PID1 |  | GTF2E2 |  |
| PIK3CB |  | HEMK1 |  |
| PIM1 |  | GTF3C6 |  |
| PISD |  | KHNYN |  |
| PITHD1 |  | KIAA0040 |  |
| PITPNM2 |  | HES4 |  |
| PKD1 |  | KIAA0101 |  |
| PLA2G3 |  | KIAA0141 |  |
| PLAGL2 |  | GUCY1A3 |  |
| PLBD2 |  | KIAA0319 |  |
| PLCB2 |  | KIAA0355 |  |
| PLCB4 |  | KIAA0408 |  |
| PLD2 |  | CTC-241N9.1 |  |
| PLEKHO2 |  | CTCF |  |
| PLXDC1 |  | PCSK1 |  |
| PNPLA2 |  | GPR75-ASB3 |  |
| PNPLA6 |  | PCYOX1 |  |
| PPIH |  | ARID4B |  |
| PPIL2 |  | HINT3 |  |
| PPM1A |  | HIPK1 |  |
| PPM1F |  | KIAA1033 |  |
| PPM1H |  | NCR1 |  |
| PPME1 |  | NCR3LG1 |  |
| PPP1R12B |  | NCS1 |  |
| PPP1R16B |  | CTDSPL |  |
| PPP1R1A |  | NDE1 |  |
| PRDM16 |  | KIAA1211 |  |
| PPP1R3F |  | KIAA1217 |  |
| PPP1R8 |  | KIAA1244 |  |
| PPP2CB |  | NDNL2 |  |
| PROX1 |  | NDRG3 |  |
| PRICKLE2 |  | NDRG4 |  |
| PRKAB1 |  | KIAA1432 |  |
| PRKAR1A |  | KIAA1456 |  |
| PRR15L |  | HDAC4 |  |
| PRR23B |  | KIAA1468 |  |
| PRRC2B |  | NDUFAF6 |  |
| TMEM25 |  | NDUFB5 |  |
| USP6NL |  | KIAA1549 |  |
| VAPB |  | NDUFB9 |  |
| VEZF1 |  | NDUFC2 |  |
| VGLL4 |  | HEATR1 |  |
| WDR35 |  | HEATR4 |  |
| WDR47 |  | HEATR5A |  |
| XIRP2 |  | KIAA1644 |  |
| YOD1 |  | HECTD2 |  |
| YWHAG |  | WT1 |  |
| ZADH2 |  | NEDD9 |  |
| ZC3H13 |  | WWC1 |  |
| ZEB2 |  | KIAA1958 |  |
| ZFP30 |  | NEK3 |  |
| ZFP90 |  | NEK4 |  |
| ZFPM2 |  | NEK6 |  |
| ZFYVE16 |  | NEK7 |  |
| ZNF181 |  | NEK9 |  |
| ZNF25 |  | NEMF |  |
| ZNF275 |  | NETO1 |  |
| ZNF385A |  | NETO2 |  |
| ZNF615 |  | NEUROD1 |  |
| ZNF624 |  | NEUROD4 |  |
| ZNF74 |  | GAPDH |  |
| ZNF93 |  | NFAM1 |  |
| TOX |  | NFASC |  |
| TRPC3 |  | NFATC2IP |  |
| TRPS1 |  | NFE2L2 |  |
| TSPAN13 |  | NFIB |  |
| TUB |  | HOOK3 |  |
| TUBA1A |  | NFYB |  |
| TYMSOS |  | DCUN1D3 |  |
| UBE2J1 |  | NHLRC1 |  |
| UBR5 |  | DCX |  |
| URI1 |  | NHP2L1 |  |
| USP27X |  | KITLG |  |
| ACTR2 |  | NID2 |  |
| SEC24B |  | NIF3L1 |  |
| SEMA3B |  | GBAS |  |
| SEMA3C |  | NINL |  |
| SENP7 |  | GBP2 |  |
| SLC4A7 |  | DDX11 |  |
| SLC6A4 |  | NIPAL3 |  |
| SMARCA5 |  | DDX26B |  |
| SNAP29 |  | DDX3X |  |
| SNCA |  | KLF7 |  |
| SNCB |  | DDX46 |  |
| SNRNP48 |  | DDX5 |  |
| SOD2 |  | HS6ST2 |  |
| STK24 |  | KLHL11 |  |
| STK38L |  | KLHL12 |  |
| STYX |  | KLHL14 |  |
| SVIP |  | HSD17B10 |  |
| SYBU |  | KLHL23 |  |
| SYT10 |  | HSD17B13 |  |
| TCF12 |  | HSDL1 |  |
| TDRP |  | HSH2D |  |
| TFG |  | HSP90B1 |  |
| TIMP3 |  | BROX |  |
| TMCC1 |  | HSPA13 |  |
| C6orf118 |  | HSPA1B |  |
| C8orf22 |  | BRSK2 |  |
| CAPRIN2 |  | HSPA4 |  |
| CASZ1 |  | KLHL6 |  |
| CBFB |  | GFM1 |  |
| CBWD1 |  | CD55 |  |
| CBWD2 |  | NMT2 |  |
| CBWD3 |  | NMUR1 |  |
| CBWD5 |  | NNT |  |
| CBWD6 |  | KLRC4 |  |
| CCDC148 |  | NOD2 |  |
| CCDC18 |  | NOG |  |
| CCSAP |  | NOL10 |  |
| CD164 |  | NOL4 |  |
| CD4 |  | KMT2A |  |
| CDK19 |  | NONO |  |
| CDKN1B |  | GID8 |  |
| CEP41 |  | KNTC1 |  |
| CHD8 |  | NOTCH2 |  |
| CHORDC1 |  | KPNA2 |  |
| CHSY1 |  | KPNA3 |  |
| CLGN |  | NPAS2 |  |
| CLVS2 |  | ZNF664 |  |
| CPNE8 |  | NPBWR1 |  |
| CREBZF |  | KPNB1 |  |
| CRX |  | NPFFR1 |  |
| CXCL12 |  | NPHP1 |  |
| CYLD |  | NPHP3 |  |
| CYP1B1 |  | NPHS1 |  |
| DCAF12 |  | KRT32 |  |
| DCUN1D4 |  | KRT6A |  |
| DDX42 |  | KRT6C |  |
| DIRAS2 |  | PTN |  |
| DMRT3 |  | NPTX1 |  |
| DNAJC6 |  | NPVF |  |
| DPH6 |  | NPY1R |  |
| DPP8 |  | KRTAP4-4 |  |
| DPY19L3 |  | KSR1 |  |
| EIF3J |  | KSR2 |  |
| EIF5A2 |  | KTI12 |  |
| EML6 |  | L2HGDH |  |
| ENTPD7 |  | L3MBTL2 |  |
| EOGT |  | NR4A2 |  |
| ERCC4 |  | L3MBTL4 |  |
| ESR1 |  | LACE1 |  |
| ETV3 |  | LAMA4 |  |
| EVI2A |  | DMBX1 |  |
| FAM160B1 |  | LAMP2 |  |
| FAM35A |  | LANCL1 |  |
| FBXO28 |  | DMRT1 |  |
| FERMT2 |  | DMRTA1 |  |
| FGF9 |  | DMRTC1 |  |
| FKBP9 |  | HS3ST3B1 |  |
| FMR1 |  | LARP4B |  |
| FNDC3A |  | LARS2 |  |
| FXN |  | IGSF9B |  |
| GABRA1 |  | IKBKAP |  |
| GABRG1 |  | IKZF1 |  |
| GALNT3 |  | LBR |  |
| GDF9 |  | CTXN2 |  |
| GNAI2 |  | NSF |  |
| GNAI3 |  | LCP1 |  |
| GPBP1 |  | NSUN4 |  |
| GPD2 |  | NSUN5 |  |
| GPM6A |  | AQP3 |  |
| GUCY1A2 |  | LDLRAD4 |  |
| HECTD2 |  | LEF1 |  |
| HIPK1 |  | NT5DC1 |  |
| HIPK3 |  | NT5DC3 |  |
| HLTF |  | NT5E |  |
| HMBOX1 |  | LEPREL1 |  |
| HNRNPD |  | NTPCR |  |
| HNRNPH3 |  | ARF1 |  |
| HOOK1 |  | ARF6 |  |
| IGF2BP2 |  | NPAS3 |  |
| ADAM22 |  | ARHGAP11A |  |
| ADAMTS6 |  | ARHGAP12 |  |
| AGTPBP1 |  | FAM222A |  |
| AKAP5 |  | LHFPL2 |  |
| ANGPTL2 |  | LHFPL5 |  |
| ANKIB1 |  | NUDT3 |  |
| ANKRD12 |  | MFN1 |  |
| ANKS1B |  | NUDT9 |  |
| AP3B2 |  | NUF2 |  |
| AQP3 |  | NUFIP2 |  |
| ARF4 |  | NUMB |  |
| ARNT |  | NUP107 |  |
| ASB4 |  | NUP133 |  |
| ASB7 |  | NUP153 |  |
| ASPA |  | NUP155 |  |
| ATF2 |  | NUP160 |  |
| BICDL1 |  | NUP188 |  |
| BRWD1 |  | NUP210 |  |
| BRWD3 |  | NUP35 |  |
| C11orf87 |  | NUP37 |  |
| C3orf70 |  | NUP43 |  |
| C6 |  | NUP50 |  |
| LYPLA1 |  | NUP54 |  |
| MAN2A1 |  | NUP62CL |  |
| MAPK10 |  | NUP93 |  |
| MAT2A |  | NUPL2 |  |
| MCMDC2 |  | NUPR1L |  |
| MED1 |  | NUS1 |  |
| MEGF9 |  | NXF1 |  |
| MESDC1 |  | NXN |  |
| MEX3A |  | NXPE2 |  |
| MIA3 |  | NXPH1 |  |
| MIDN |  | NXPH2 |  |
| MRAP |  | NXPH3 |  |
| MRAP2 |  | MITF |  |
| MSL2 |  | NYAP2 |  |
| MTMR6 |  | NYNRIN |  |
| MYBL1 |  | MKLN1 |  |
| MYLIP |  | OCA2 |  |
| MYO10 |  | OCIAD2 |  |
| NAA25 |  | GPR12 |  |
| NAP1L1 |  | IRX2 |  |
| NAP1L5 |  | GNG10 |  |
| NBPF3 |  | GNG12 |  |
| NFATC3 |  | CPPED1 |  |
| NFYB |  | MMGT1 |  |
| NRG1 |  | MMP13 |  |
| NRXN1 |  | OR12D3 |  |
| NSMCE4A |  | ISX |  |
| NTF3 |  | OR2T33 |  |
| NXPH1 |  | AC004899.1 |  |
| OSTM1 |  | OR4N4 |  |
| PAF1 |  | OR51E1 |  |
| PAFAH1B2 |  | OR9Q1 |  |
| PAIP1 |  | MMS22L |  |
| PAIP2 |  | ITGA6 |  |
| PAK1 |  | ITGA8 |  |
| PARP9 |  | GOLGA7 |  |
| PCDHA1 |  | ITGB3 |  |
| PCDHA11 |  | ITGB3BP |  |
| PCDHA12 |  | ITGB8 |  |
| PCDHA13 |  | ITGBL1 |  |
| PCDHA2 |  | ITK |  |
| PCDHA3 |  | ITM2B |  |
| PCDHA4 |  | ITPR1 |  |
| PCDHA5 |  | OSBPL9 |  |
| PCDHA6 |  | OSGIN2 |  |
| PCDHA7 |  | IVD |  |
| PCDHA9 |  | OSTF1 |  |
| PCDHAC1 |  | GON4L |  |
| PCDHAC2 |  | JAGN1 |  |
| PCMTD1 |  | JAK2 |  |
| PDCD10 |  | GORAB |  |
| PGPEP1L |  | JAKMIP2 |  |
| PHACTR4 |  | GOSR1 |  |
| PHEX |  | JAM3 |  |
| PHF2 |  | GPALPP1 |  |
| PI15 |  | JDP2 |  |
| PIK3R1 |  | JHDM1D |  |
| PLCL2 |  | JKAMP |  |
| PLPPR1 |  | JMJD1C |  |
| PLSCR4 |  | JMY |  |
| POLR3E |  | JPH1 |  |
| PPP3R1 |  | USP33 |  |
| PPP6C |  | KAL1 |  |
| PRDM2 |  | KANK4 |  |
| RAB18 |  | KANSL1L |  |
| RALGAPA1 |  | KANSL3 |  |
| RASGRP1 |  | KARS |  |
| RBM24 |  | GPM6B |  |
| RDX |  | KAT6A |  |
| RFX8 |  | GPR107 |  |
| RGS6 |  | GPR111 |  |
| RIT2 |  | GPR114 |  |
| RPS3 |  | GPR115 |  |
| RSBN1L |  | GPR123 |  |
| SBK1 |  | PACRG |  |
| SCN11A |  | GPR125 |  |
| INSIG1 |  | PACS1 |  |
| IRF2 |  | GPR135 |  |
| IRX5 |  | GPR137C |  |
| ITGB3 |  | KCNA1 |  |
| KCNK2 |  | GPR151 |  |
| KCNQ3 |  | PAICS |  |
| KDSR |  | GPR156 |  |
| KIAA0586 |  | GPR158 |  |
| KIAA1841 |  | GPR161 |  |
| KIF20A |  | GPR173 |  |
| KLC1 |  | GPR174 |  |
| L3MBTL1 |  | PAK4 |  |
| LDHAL6B |  | MSN |  |
| LOC100506388 |  | PALD1 |  |
| LRFN2 |  | PALM2 |  |
| LRP10 |  | PALM2-AKAP2 |  |
| LRRCC1 |  | PAM16 |  |
| LRRTM2 |  | PANK1 |  |
| LUZP2 |  | PANK2 |  |
| INA |  | PANK3 |  |
| CBX5 |  | PANX1 |  |
| CCDC96 |  | PANX2 |  |
| CCKBR |  | PAOX |  |
| CDC27 |  | PAPD4 |  |
| CDK17 |  | PAPD5 |  |
| CEP72 |  | PAPOLA |  |
| CFTR |  | PAPOLB |  |
| CLDN8 |  | PAPOLG |  |
| C6orf136 |  | PAPPA |  |
| CMPK1 |  | PAPPA2 |  |
| CNEP1R1 |  | PAPSS1 |  |
| CNOT6L |  | PAQR3 |  |
| CNTN5 |  | VNN2 |  |
| CXXC4 |  | CASC4 |  |
| CYB5A |  | NAA11 |  |
| CYBRD1 |  | PARD3B |  |
| DBT |  | PARL |  |
| DENND5B |  | NAA30 |  |
| DERL1 |  | CNOT6L |  |
| DTNA |  | PARP14 |  |
| DUSP10 |  | NAA50 |  |
| EBF2 |  | PARP8 |  |
| EBNA1BP2 |  | PARP9 |  |
| ECT2 |  | PARPBP |  |
| EPB41L3 |  | PARS2 |  |
| EPHA3 |  | PARVA |  |
| F3 |  | PATE2 |  |
| FAM120C |  | PATL1 |  |
| FAM13C |  | PAWR |  |
| FAM98A |  | PAX1 |  |
| FBXO8 |  | PAX3 |  |
| FEZ2 |  | PAX4 |  |
| FGF2 |  | KCTD6 |  |
| FKBP5 |  | KCTD7 |  |
| FOXO1 |  | NAMPT |  |
| FOXO3 |  | PBRM1 |  |
| G0S2 |  | PBX3 |  |
| CLEC14A |  | PCBD2 |  |
| GDPD1 |  | PCCB |  |
| GPR171 |  | PCDH10 |  |
| GPX8 |  | PCDH11X |  |
| GRM6 |  | PCDH11Y |  |
| GTPBP8 |  | GTF2E1 |  |
| HARBI1 |  | NAPG |  |
| HIKESHI |  | PCDH19 |  |
| HSP90B1 |  | PCDH20 |  |
| IGF1R |  | HEPH |  |
| IL6ST |  | HERC3 |  |
| INHBB |  | HERPUD1 |  |
| INPP4A |  | PCDHB13 |  |
| INPP5B |  | HEXA |  |
| KAT6A |  | HEY1 |  |
| KBTBD6 |  | HEY2 |  |
| KDM4E |  | HEYL |  |
| KHDRBS1 |  | PCDHB5 |  |
| KIAA1468 |  | KIAA0391 |  |
| LACC1 |  | CTC1 |  |
| LCORL |  | PCK1 |  |
| LMO2 |  | PCM1 |  |
| LOC401052 |  | KIAA0586 |  |
| LRIG1 |  | PCSK5 |  |
| LRRC19 |  | KIAA0895 |  |
| LYZL6 |  | KIAA1009 |  |
| MARCH10 |  | NAA10 |  |
| MARCH3 |  | PDCD4 |  |
| MEF2D |  | AGTR1 |  |
| MINDY3 |  | AGTR2 |  |
| ABCD4 |  | NDC1 |  |
| ACSL3 |  | NDN |  |
| ACVR2A |  | NDNF |  |
| ADCY7 |  | PDE5A |  |
| AK2 |  | NDST4 |  |
| ALCAM |  | NDUFA5 |  |
| ANAPC10 |  | NDUFAF4 |  |
| ANKRD10 |  | NDUFAF5 |  |
| ARMCX1 |  | AKAP13 |  |
| ARRDC3 |  | NDUFB6 |  |
| ATG7 |  | AKR1D1 |  |
| ATP10D |  | AKT2 |  |
| ATP1B1 |  | NDUFC2-KCTD14 | |
| ATP7A |  | NDUFS1 |  |
| AUTS2 |  | NDUFS6 |  |
| BET1L |  | NDUFV2 |  |
| BRMS1L |  | NECAB1 |  |
| C18orf54 |  | PDPK1 |  |
| PHIP |  | NEFM |  |
| PKN2 |  | NEGR1 |  |
| PKNOX1 |  | PDS5A |  |
| PLEKHH1 |  | PDS5B |  |
| POLR3G |  | PDXDC1 |  |
| PRDM1 |  | PDXK |  |
| PRSS57 |  | PDZD11 |  |
| PTBP2 |  | PDZD2 |  |
| PTPN2 |  | PDZD3 |  |
| RAB8B |  | PDZD8 |  |
| RABGAP1L |  | PDZK1 |  |
| RALGPS2 |  | PEA15 |  |
| RAP2A |  | NF1 |  |
| RASA1 |  | PEBP1 |  |
| RASSF6 |  | NCKAP5 |  |
| RBSN |  | PELI1 |  |
| RHOB |  | PELI2 |  |
| RIBC1 |  | PELO |  |
| RILPL1 |  | PENK |  |
| RNF14 |  | PET117 |  |
| RNF34 |  | PEX1 |  |
| RPS6KB1 |  | PEX11A |  |
| RUBCNL |  | PEX12 |  |
| RWDD4 |  | PEX13 |  |
| SACS |  | PEX19 |  |
| SCAF8 |  | PEX3 |  |
| SEPT10 |  | PEX5L |  |
| SEPT2 |  | NHS |  |
| SEPT6 |  | PFDN6 |  |
| SLC23A2 |  | PFKFB2 |  |
| SLC25A32 |  | PGAM1 |  |
| SLC26A7 |  | PGAP1 |  |
| SLC29A1 |  | PGAP2 |  |
| SLC35G2 |  | PGAP3 |  |
| SLC39A1 |  | PGBD4 |  |
| SLC4A4 |  | PGGT1B |  |
| SMAD1 |  | PGK1 |  |
| SMIM15 |  | DDX3Y |  |
| SNX6 |  | PGM2L1 |  |
| SREK1 |  | PGM3 |  |
| SRP54 |  | PGPEP1 |  |
| STIM1 |  | HS6ST3 |  |
| STK39 |  | HSBP1 |  |
| TBC1D17 |  | HSD11B2 |  |
| TMEM143 |  | PHACTR2 |  |
| TMEM178B |  | HSD17B12 |  |
| TMEM47 |  | PHC1 |  |
| TMPO |  | PHC3 |  |
| TRIL |  | PHEX |  |
| TSPAN7 |  | PHF10 |  |
| TUBA1B |  | HSPA12A |  |
| ULK2 |  | PHF14 |  |
| USP16 |  | PHF15 |  |
| USP40 |  | HSPA1L |  |
| VNN1 |  | PHF20L1 |  |
| WBP1L |  | HSPA4L |  |
| YPEL1 |  | CD47 |  |
| ZBTB42 |  | PHF3 |  |
| ZCCHC14 |  | PHF6 |  |
| ZFHX3 |  | PHF7 |  |
| ZNF365 |  | PHF8 |  |
| ZNF500 |  | NOD1 |  |
| ZNF706 |  | PHKA1 |  |
| ZNF772 |  | PHKB |  |
| MPP5 |  | PHLDA1 |  |
| MTPN |  | PHLDA2 |  |
| MTSS1 |  | NOLC1 |  |
| MYH10 |  | PHLDB2 |  |
| MYO5B |  | PHTF1 |  |
| NAIF1 |  | PHYHIP |  |
| NDNF |  | NOTCH2NL |  |
| NETO1 |  | ZNF652 |  |
| NFIB |  | PI4K2B |  |
| NIPAL3 |  | PIAS1 |  |
| NLRP3 |  | PIAS4 |  |
| NPY1R |  | NPC2 |  |
| NUP160 |  | PIEZO1 |  |
| NUP210 |  | NFKBIZ |  |
| NXPE3 |  | NFXL1 |  |
| OGN |  | PIGH |  |
| OXTR |  | NPHS2 |  |
| PARD6B |  | NPLOC4 |  |
| PAX5 |  | NPM1 |  |
| PELI1 |  | KRT77 |  |
| PEX3 |  | PIGT |  |
| PHF19 |  | PIGW |  |
|  |  | PIGX |  |
|  |  | NPY2R |  |
|  |  | NQO2 |  |
|  |  | NR1D2 |  |
|  |  | NR1H4 |  |
|  |  | NR2F2 |  |
|  |  | NR3C2 |  |
|  |  | PIK3CD |  |
|  |  | NR4A3 |  |
|  |  | NR5A2 |  |
|  |  | NR6A1 |  |
|  |  | LAMC1 |  |
|  |  | NRBF2 |  |
|  |  | NRCAM |  |
|  |  | LANCL3 |  |
|  |  | LAP3 |  |
|  |  | HS3ST3A1 |  |
|  |  | NRG4 |  |
|  |  | NRIP3 |  |
|  |  | NRK |  |
|  |  | LATS1 |  |
|  |  | LAX1 |  |
|  |  | LBP |  |
|  |  | NRXN3 |  |
|  |  | NSD1 |  |
|  |  | PKLR |  |
|  |  | NSUN2 |  |
|  |  | NSUN3 |  |
|  |  | PLA2G12A |  |
|  |  | PLA2G15 |  |
|  |  | NSUN7 |  |
|  |  | NT5C1A |  |
|  |  | NT5C2 |  |
|  |  | NT5C3A |  |
|  |  | PLAA |  |
|  |  | PLAC8 |  |
|  |  | PLAT |  |
|  |  | NTNG1 |  |
|  |  | PLCB4 |  |
|  |  | NTRK2 |  |
|  |  | NUBPL |  |
|  |  | NUCKS1 |  |
|  |  | NUDCD1 |  |
|  |  | NUDCD2 |  |
|  |  | NUDCD3 |  |
|  |  | METTL8 |  |
|  |  | METTL9 |  |
|  |  | MEX3B |  |
|  |  | MEX3C |  |
|  |  | MFAP3 |  |
|  |  | MFAP3L |  |
|  |  | PLEKHG1 |  |
|  |  | PLEKHG2 |  |
|  |  | PLEKHG4B |  |
|  |  | NPNT |  |
|  |  | PLEKHH2 |  |
|  |  | PLEKHM3 |  |
|  |  | PLEKHO2 |  |
|  |  | PLEKHS1 |  |
|  |  | PLG |  |
|  |  | PLGLB1 |  |
|  |  | PLGLB2 |  |
|  |  | PLK2 |  |
|  |  | PLK4 |  |
|  |  | PLN |  |
|  |  | PLRG1 |  |
|  |  | PLS3 |  |
|  |  | PLSCR1 |  |
|  |  | PLXDC2 |  |
|  |  | PLXNA1 |  |
|  |  | PLXNA2 |  |
|  |  | PLXNB1 |  |
|  |  | PLXNC1 |  |
|  |  | PM20D1 |  |
|  |  | PM20D2 |  |
|  |  | PMAIP1 |  |
|  |  | PML |  |
|  |  | PMM2 |  |
|  |  | PMPCB |  |
|  |  | PMVK |  |
|  |  | PNISR |  |
|  |  | PNLDC1 |  |
|  |  | PNLIPRP3 |  |
|  |  | LMF1 |  |
|  |  | PNN |  |
|  |  | ARL14EPL |  |
|  |  | ARL3 |  |
|  |  | IRGQ |  |
|  |  | FBLIM1 |  |
|  |  | ITCH |  |
|  |  | ITFG1 |  |
|  |  | LPIN2 |  |
|  |  | ARSG |  |
|  |  | ASAP3 |  |
|  |  | ASB11 |  |
|  |  | ASB18 |  |
|  |  | ASB6 |  |
|  |  | ASB7 |  |
|  |  | ASCL1 |  |
|  |  | ASF1A |  |
|  |  | ASNSD1 |  |
|  |  | NUP88 |  |
|  |  | PPARA |  |
|  |  | PPARGC1A |  |
|  |  | NWD1 |  |
|  |  | FDXACB1 |  |
|  |  | ASUN |  |
|  |  | ASXL1 |  |
|  |  | FEM1B |  |
|  |  | ATF2 |  |
|  |  | ATF3 |  |
|  |  | ATG16L2 |  |
|  |  | ATG4C |  |
|  |  | FGF5 |  |
|  |  | DPH1 |  |
|  |  | FGF7 |  |
|  |  | ATIC |  |
|  |  | ATL1 |  |
|  |  | FGFR1OP |  |
|  |  | FGFR3 |  |
|  |  | OPA3 |  |
|  |  | ATP11A |  |
|  |  | ATP11C |  |
|  |  | ATP13A1 |  |
|  |  | FIBIN |  |
|  |  | OR52N2 |  |
|  |  | ORMDL1 |  |
|  |  | OSBPL10 |  |
|  |  | ATP6V0A1 |  |
|  |  | ATP6V0A4 |  |
|  |  | ATP6V0B |  |
|  |  | OSBPL3 |  |
|  |  | OSBPL6 |  |
|  |  | ATP6V1C1 |  |
|  |  | FMN2 |  |
|  |  | FMNL2 |  |
|  |  | FMNL3 |  |
|  |  | FMO3 |  |
|  |  | PAQR4 |  |
|  |  | PAQR5 |  |
|  |  | PAQR7 |  |
|  |  | PAQR9 |  |
|  |  | PARD3 |  |
|  |  | FNDC3A |  |
|  |  | ATR |  |
|  |  | PARN |  |
|  |  | PARP11 |  |
|  |  | PARP15 |  |
|  |  | FOXB1 |  |
|  |  | FOXD3 |  |
|  |  | FOXE1 |  |
|  |  | FOXF1 |  |
|  |  | FOXG1 |  |
|  |  | AURKB |  |
|  |  | FOXM1 |  |
|  |  | FOXN2 |  |
|  |  | FOXN3 |  |
|  |  | FOXO6 |  |
|  |  | PAX5 |  |
|  |  | PAX6 |  |
|  |  | PAX8 |  |
|  |  | PAX9 |  |
|  |  | B3GALT5 |  |
|  |  | PCDH17 |  |
|  |  | PCDH18 |  |
|  |  | FRMD5 |  |
|  |  | PCDH8 |  |
|  |  | PCDH9 |  |
|  |  | PCDHB12 |  |
|  |  | B4GALT1 |  |
|  |  | PCDHB15 |  |
|  |  | PCDHB17 |  |
|  |  | PCDHB2 |  |
|  |  | CTBP2 |  |
|  |  | PCDP1 |  |
|  |  | PCGF5 |  |
|  |  | FST |  |
|  |  | FSTL5 |  |
|  |  | PCMT1 |  |
|  |  | H1FX |  |
|  |  | PCNX |  |
|  |  | N4BP3 |  |
|  |  | FUBP3 |  |
|  |  | CTD-2140B24.4 |  |
|  |  | FUNDC2 |  |
|  |  | CTD-2368P22.1 |  |
|  |  | FUT10 |  |
|  |  | CTD-3074O7.11 |  |
|  |  | AHCYL2 |  |
|  |  | BBS7 |  |
|  |  | BBX |  |
|  |  | AIFM2 |  |
|  |  | BCCIP |  |
|  |  | BCL10 |  |
|  |  | BCL11A |  |
|  |  | BCL11B |  |
|  |  | AK5 |  |
|  |  | CTGF |  |
|  |  | BCL6B |  |
|  |  | AKIRIN1 |  |
|  |  | BCL9 |  |
|  |  | BCL9L |  |
|  |  | PDK4 |  |
|  |  | PDLIM2 |  |
|  |  | PDLIM3 |  |
|  |  | PDLIM5 |  |
|  |  | PDP1 |  |
|  |  | PDP2 |  |
|  |  | BECN1 |  |
|  |  | PDPR |  |
|  |  | PDRG1 |  |
|  |  | BEND3 |  |
|  |  | BEND4 |  |
|  |  | BEND7 |  |
|  |  | BEST3 |  |
|  |  | PTBP3 |  |
|  |  | PTCD2 |  |
|  |  | BEX1 |  |
|  |  | PTCH1 |  |
|  |  | PTCHD1 |  |
|  |  | GALNT7 |  |
|  |  | PEAK1 |  |
|  |  | GALNT8 |  |
|  |  | GAN |  |
|  |  | PTGER2 |  |
|  |  | PTGER3 |  |
|  |  | PTGER4 |  |
|  |  | PTGES3 |  |
|  |  | BLCAP |  |
|  |  | PTGIS |  |
|  |  | BLOC1S6 |  |
|  |  | BLVRA |  |
|  |  | BLZF1 |  |
|  |  | BMF |  |
|  |  | BMP2K |  |
|  |  | BMP5 |  |
|  |  | PEX7 |  |
|  |  | GATM |  |
|  |  | GATSL2 |  |
|  |  | PFKM |  |
|  |  | BMPR1B |  |
|  |  | PGAM4 |  |
|  |  | BMS1 |  |
|  |  | BMX |  |
|  |  | GCA |  |
|  |  | ENOX2 |  |
|  |  | BOD1L1 |  |
|  |  | BNIP3L |  |
|  |  | PGM2 |  |
|  |  | BOD1L2 |  |
|  |  | BPHL |  |
|  |  | GCNT3 |  |
|  |  | PGR |  |
|  |  | PGRMC2 |  |
|  |  | PHACTR1 |  |
|  |  | GCSAML |  |
|  |  | PHACTR3 |  |
|  |  | GDAP1L1 |  |
|  |  | GDE1 |  |
|  |  | GDF10 |  |
|  |  | GDF6 |  |
|  |  | PHF12 |  |
|  |  | GDI1 |  |
|  |  | BRS3 |  |
|  |  | PHF19 |  |
|  |  | GDPD5 |  |
|  |  | PHF21A |  |
|  |  | PHF21B |  |
|  |  | CTNNA1 |  |
|  |  | CTNNA3 |  |
|  |  | BTAF1 |  |
|  |  | BTBD1 |  |
|  |  | PHIP |  |
|  |  | GGA2 |  |
|  |  | BTBD7 |  |
|  |  | GGACT |  |
|  |  | BTD |  |
|  |  | PHLDA3 |  |
|  |  | BTG2 |  |
|  |  | PHLPP2 |  |
|  |  | BTLA |  |
|  |  | PHTF2 |  |
|  |  | CTR9 |  |
|  |  | PHYHIPL |  |
|  |  | PI15 |  |
|  |  | GINM1 |  |
|  |  | GINS1 |  |
|  |  | GINS2 |  |
|  |  | PICALM |  |
|  |  | GIPC2 |  |
|  |  | GIT2 |  |
|  |  | GJA1 |  |
|  |  | GJA3 |  |
|  |  | PIGK |  |
|  |  | PIGM |  |
|  |  | PIGN |  |
|  |  | C10orf76 |  |
|  |  | C11orf1 |  |
|  |  | C11orf16 |  |
|  |  | PIH1D3 |  |
|  |  | PIK3C2A |  |
|  |  | PIK3C2B |  |
|  |  | PIK3C2G |  |
|  |  | PIK3CA |  |
|  |  | PIK3CB |  |
|  |  | CTSS |  |
|  |  | PIK3R1 |  |
|  |  | PIK3R4 |  |
|  |  | PIKFYVE |  |
|  |  | NRAS |  |
|  |  | PIP5K1A |  |
|  |  | PIP5K1B |  |
|  |  | NRD1 |  |
|  |  | NREP |  |
|  |  | NRG1 |  |
|  |  | PITPNM2 |  |
|  |  | PIWIL2 |  |
|  |  | PJA2 |  |
|  |  | NRP1 |  |
|  |  | NRP2 |  |
|  |  | NRXN1 |  |
|  |  | PKIA |  |
|  |  | PKIB |  |
|  |  | C15orf41 |  |
|  |  | PKN2 |  |
|  |  | PKNOX1 |  |
|  |  | C16orf45 |  |
|  |  | C16orf46 |  |
|  |  | PLA2G16 |  |
|  |  | PLA2G4A |  |
|  |  | PLA2G7 |  |
|  |  | PLA2R1 |  |
|  |  | C17orf107 |  |
|  |  | C17orf75 |  |
|  |  | C17orf85 |  |
|  |  | PLCB1 |  |
|  |  | RALBP1 |  |
|  |  | PLCE1 |  |
|  |  | PLCG1 |  |
|  |  | PLCG2 |  |
|  |  | PLCL1 |  |
|  |  | PLCXD3 |  |
|  |  | PLD1 |  |
|  |  | PLD5 |  |
|  |  | PLEKHA1 |  |
|  |  | PLEKHA3 |  |
|  |  | PLEKHA7 |  |
|  |  | PLEKHA8 |  |
|  |  | PLEKHB2 |  |
|  |  | CUEDC2 |  |
|  |  | C1GALT1 |  |
|  |  | C1orf112 |  |
|  |  | C1orf122 |  |
|  |  | C1orf143 |  |
|  |  | C1orf145 |  |
|  |  | C1orf173 |  |
|  |  | C1orf174 |  |
|  |  | C1orf189 |  |
|  |  | C1orf198 |  |
|  |  | C1orf204 |  |
|  |  | C1orf213 |  |
|  |  | C1orf27 |  |
|  |  | C1orf50 |  |
|  |  | RASD2 |  |
|  |  | C1orf56 |  |
|  |  | RASGEF1A |  |
|  |  | C1QL3 |  |
|  |  | C1QTNF7 |  |
|  |  | C20orf112 |  |
|  |  | C20orf194 |  |
|  |  | C20orf197 |  |
|  |  | C21orf58 |  |
|  |  | C22orf23 |  |
|  |  | C22orf29 |  |
|  |  | C2CD2 |  |
|  |  | C2CD4C |  |
|  |  | C2CD5 |  |
|  |  | RAX |  |
|  |  | C2orf49 |  |
|  |  | C2orf72 |  |
|  |  | RBAK |  |
|  |  | C2orf88 |  |
|  |  | C2orf91 |  |
|  |  | C3orf33 |  |
|  |  | C3orf38 |  |
|  |  | C3orf58 |  |
|  |  | C3orf80 |  |
|  |  | C4orf29 |  |
|  |  | C4orf32 |  |
|  |  | C4orf33 |  |
|  |  | C4orf40 |  |
|  |  | C4orf45 |  |
|  |  | C4orf46 |  |
|  |  | C4orf50 |  |
|  |  | C5AR2 |  |
|  |  | C5orf24 |  |
|  |  | C5orf30 |  |
|  |  | C5orf45 |  |
|  |  | C5orf47 |  |
|  |  | C5orf51 |  |
|  |  | C5orf63 |  |
|  |  | C6orf106 |  |
|  |  | C6orf120 |  |
|  |  | C6ORF165 |  |
|  |  | C6orf195 |  |
|  |  | C6orf211 |  |
|  |  | C6orf222 |  |
|  |  | C6orf25 |  |
|  |  | C6orf47 |  |
|  |  | C6ORF50 |  |
|  |  | C6orf89 |  |
|  |  | C7 |  |
|  |  | C7orf13 |  |
|  |  | C7orf41 |  |
|  |  | C7orf55-LUC7L2 |  |
|  |  | C7orf60 |  |
|  |  | RCAN1 |  |
|  |  | C8orf37 |  |
|  |  | C8orf4 |  |
|  |  | C8orf44-SGK3 |  |
|  |  | C8orf76 |  |
|  |  | C8orf82 |  |
|  |  | RCN2 |  |
|  |  | C9orf142 |  |
|  |  | PLOD1 |  |
|  |  | PLOD2 |  |
|  |  | C9orf40 |  |
|  |  | C9orf41 |  |
|  |  | C9orf69 |  |
|  |  | C9orf72 |  |
|  |  | C9orf84 |  |
|  |  | CA12 |  |
|  |  | CA13 |  |
|  |  | CA2 |  |
|  |  | CA5B |  |
|  |  | CA8 |  |
|  |  | CAAP1 |  |
|  |  | CABLES2 |  |
|  |  | CABYR |  |
|  |  | CACHD1 |  |
|  |  | CACNA1B |  |
|  |  | CACNA1C |  |
|  |  | CACNA1D |  |
|  |  | CACNA1E |  |
|  |  | CACNA2D1 |  |
|  |  | CACNB2 |  |
|  |  | CACNB4 |  |
|  |  | CACNG2 |  |
|  |  | CACUL1 |  |
|  |  | CACYBP |  |
|  |  | CADM1 |  |
|  |  | RGPD2 |  |
|  |  | CADPS2 |  |
|  |  | CALCA |  |
|  |  | CALCOCO2 |  |
|  |  | CALCR |  |
|  |  | RGPD8 |  |
|  |  | CALD1 |  |
|  |  | CALHM2 |  |
|  |  | CALM1 |  |
|  |  | CALML4 |  |
|  |  | CALN1 |  |
|  |  | CALU |  |
|  |  | CAMK1G |  |
|  |  | CAMK2D |  |
|  |  | CAMK2G |  |
|  |  | CAMK2N1 |  |
|  |  | CAMK4 |  |
|  |  | CAMKK1 |  |
|  |  | RHBDD3 |  |
|  |  | RHBDL2 |  |
|  |  | RHNO1 |  |
|  |  | CAMTA2 |  |
|  |  | CAND1 |  |
|  |  | CANX |  |
|  |  | CAP1 |  |
|  |  | CAP2 |  |
|  |  | CAPN10 |  |
|  |  | CAPN14 |  |
|  |  | CAPN15 |  |
|  |  | CAPN2 |  |
|  |  | CAPN7 |  |
|  |  | CAPRIN1 |  |
|  |  | RIMS1 |  |
|  |  | RIMS2 |  |
|  |  | CAPZB |  |
|  |  | CARD9 |  |
|  |  | CARF |  |
|  |  | CARTPT |  |
|  |  | CASC3 |  |
|  |  | RIT1 |  |
|  |  | RLIM |  |
|  |  | RMDN1 |  |
|  |  | CASP3 |  |
|  |  | CASP9 |  |
|  |  | CASQ1 |  |
|  |  | CATSPER2 |  |
|  |  | CAV1 |  |
|  |  | CAV2 |  |
|  |  | CAV3 |  |
|  |  | CBFA2T2 |  |
|  |  | CBFB |  |
|  |  | RNF103-CHMP3 |  |
|  |  | CBLB |  |
|  |  | PPL |  |
|  |  | CBLN2 |  |
|  |  | CBX1 |  |
|  |  | CBX4 |  |
|  |  | CBX5 |  |
|  |  | CBX6 |  |
|  |  | CC2D2B |  |
|  |  | CCAR1 |  |
|  |  | CCBE1 |  |
|  |  | CCDC112 |  |
|  |  | CCDC125 |  |
|  |  | CCDC126 |  |
|  |  | CCDC127 |  |
|  |  | CCDC138 |  |
|  |  | CCDC14 |  |
|  |  | CCDC141 |  |
|  |  | CCDC144A |  |
|  |  | CCDC149 |  |
|  |  | CCDC152 |  |
|  |  | CCDC170 |  |
|  |  | CCDC171 |  |
|  |  | CCDC172 |  |
|  |  | CCDC178 |  |
|  |  | CCDC18 |  |
|  |  | HGF |  |
|  |  | CTBS |  |
|  |  | HHIP |  |
|  |  | HHLA1 |  |
|  |  | FTCDNL1 |  |
|  |  | PCMTD1 |  |
|  |  | FUBP1 |  |
|  |  | CCDC41 |  |
|  |  | CCDC47 |  |
|  |  | CCDC50 |  |
|  |  | CCDC58 |  |
|  |  | CCDC6 |  |
|  |  | CCDC68 |  |
|  |  | CCDC73 |  |
|  |  | ROMO1 |  |
|  |  | ROR1 |  |
|  |  | CCDC85C |  |
|  |  | CCDC88A |  |
|  |  | CCDC93 |  |
|  |  | CCL3L1 |  |
|  |  | CCL8 |  |
|  |  | CCNB1 |  |
|  |  | PRDM13 |  |
|  |  | CCNC |  |
|  |  | CCND2 |  |
|  |  | CCND3 |  |
|  |  | PRDM5 |  |
|  |  | PRDM6 |  |
|  |  | CCNG1 |  |
|  |  | PREX2 |  |
|  |  | CCNH |  |
|  |  | CCNJ |  |
|  |  | RP11-383H13.1 |  |
|  |  | CCNO |  |
|  |  | CCNT1 |  |
|  |  | CCNT2 |  |
|  |  | CCNY |  |
|  |  | CCNYL1 |  |
|  |  | RP11-664D7.4 |  |
|  |  | RP11-67H2.1 |  |
|  |  | RP1-170O19.20 |  |
|  |  | RP11-73M18.2 |  |
|  |  | CCT5 |  |
|  |  | CCT6A |  |
|  |  | CCT8 |  |
|  |  | CD109 |  |
|  |  | CD163 |  |
|  |  | CD164 |  |
|  |  | CD209 |  |
|  |  | CD226 |  |
|  |  | CD28 |  |
|  |  | RPF1 |  |
|  |  | RPF2 |  |
|  |  | CD302 |  |
|  |  | RPH3AL |  |
|  |  | CD38 |  |
|  |  | CD4 |  |
|  |  | CD40LG |  |
|  |  | CD44 |  |
|  |  | CD82 |  |
|  |  | PRR11 |  |
|  |  | PRR14L |  |
|  |  | PRR15L |  |
|  |  | PRR23B |  |
|  |  | RPL7 |  |
|  |  | PRR3 |  |
|  |  | PRR5L |  |
|  |  | PRRC2B |  |
|  |  | CDC27 |  |
|  |  | PRRG1 |  |
|  |  | PRRG3 |  |
|  |  | CDC42EP3 |  |
|  |  | CDCA7L |  |
|  |  | CDH1 |  |
|  |  | CDH11 |  |
|  |  | CDH13 |  |
|  |  | CDH18 |  |
|  |  | CDH19 |  |
|  |  | CDH2 |  |
|  |  | CDH20 |  |
|  |  | CDH4 |  |
|  |  | CDH5 |  |
|  |  | CDH6 |  |
|  |  | PSMB1 |  |
|  |  | CDH8 |  |
|  |  | CDH9 |  |
|  |  | PSMC6 |  |
|  |  | PSMD11 |  |
|  |  | PSMD12 |  |
|  |  | PSMD6 |  |
|  |  | PSME3 |  |
|  |  | RSPH10B2 |  |
|  |  | RSPH3 |  |
|  |  | CDK6 |  |
|  |  | CDK8 |  |
|  |  | PTAR1 |  |
|  |  | PTBP1 |  |
|  |  | PTBP2 |  |
|  |  | RUNDC1 |  |
|  |  | CDKN2AIPNL |  |
|  |  | CDKN2C |  |
|  |  | CDKN3 |  |
|  |  | CDNF |  |
|  |  | CDO1 |  |
|  |  | CDON |  |
|  |  | CDR2 |  |
|  |  | CDR2L |  |
|  |  | CDS1 |  |
|  |  | CDS2 |  |
|  |  | CDV3 |  |
|  |  | PTGFR |  |
|  |  | PTGR2 |  |
|  |  | PIGP |  |
|  |  | PTH |  |
|  |  | SALL3 |  |
|  |  | SALL4 |  |
|  |  | SAMD12 |  |
|  |  | PTPDC1 |  |
|  |  | PTPLA |  |
|  |  | PTPLAD1 |  |
|  |  | PTPLAD2 |  |
|  |  | PTPLB |  |
|  |  | PTPN1 |  |
|  |  | PTPN11 |  |
|  |  | PTPN13 |  |
|  |  | PTPN14 |  |
|  |  | PIM1 |  |
|  |  | PTPN20A |  |
|  |  | CEP135 |  |
|  |  | PIP5K1C |  |
|  |  | PITPNA |  |
|  |  | PITPNB |  |
|  |  | CEP290 |  |
|  |  | CEP350 |  |
|  |  | CEP41 |  |
|  |  | PKD2 |  |
|  |  | PKHD1 |  |
|  |  | PKHD1L1 |  |
|  |  | PTPRJ |  |
|  |  | CEP63 |  |
|  |  | CEP68 |  |
|  |  | CEP76 |  |
|  |  | CEP78 |  |
|  |  | CEP85L |  |
|  |  | CEP97 |  |
|  |  | CERK |  |
|  |  | CERS2 |  |
|  |  | PTX3 |  |
|  |  | PUM2 |  |
|  |  | SCN3A |  |
|  |  | PURB |  |
|  |  | SCN5A |  |
|  |  | SCN8A |  |
|  |  | SCN9A |  |
|  |  | SCNN1G |  |
|  |  | DNAJC21 |  |
|  |  | DNAJC22 |  |
|  |  | DNAJC27 |  |
|  |  | DNAJC28 |  |
|  |  | DNAJC3 |  |
|  |  | DNAJC6 |  |
|  |  | DNAL1 |  |
|  |  | DNALI1 |  |
|  |  | DNASE1L2 |  |
|  |  | DNM1L |  |
|  |  | DNM3 |  |
|  |  | DNMT3A |  |
|  |  | CHKA |  |
|  |  | CHL1 |  |
|  |  | CHM |  |
|  |  | CHMP1B |  |
|  |  | DOCK5 |  |
|  |  | DOCK7 |  |
|  |  | DOCK8 |  |
|  |  | DOK6 |  |
|  |  | DOPEY1 |  |
|  |  | DOT1L |  |
|  |  | DPAGT1 |  |
|  |  | CHPF2 |  |
|  |  | CHRAC1 |  |
|  |  | DPF3 |  |
|  |  | DPH3 |  |
|  |  | DPP10 |  |
|  |  | DPP6 |  |
|  |  | CHRNA7 |  |
|  |  | RAB22A |  |
|  |  | DPY19L2 |  |
|  |  | DPY19L3 |  |
|  |  | CHST11 |  |
|  |  | CHST12 |  |
|  |  | DPYSL5 |  |
|  |  | DR1 |  |
|  |  | DRAM2 |  |
|  |  | DRG1 |  |
|  |  | DROSHA |  |
|  |  | DRP2 |  |
|  |  | DSC1 |  |
|  |  | DSC2 |  |
|  |  | DSC3 |  |
|  |  | DSCAM |  |
|  |  | DSE |  |
|  |  | DSEL |  |
|  |  | OCLN |  |
|  |  | OCRL |  |
|  |  | ODF2L |  |
|  |  | OGDHL |  |
|  |  | OGFOD1 |  |
|  |  | DST |  |
|  |  | OGT |  |
|  |  | OLA1 |  |
|  |  | OLFM2 |  |
|  |  | OLFML1 |  |
|  |  | ONECUT1 |  |
|  |  | ONECUT2 |  |
|  |  | MMD2 |  |
|  |  | OPTN |  |
|  |  | MME |  |
|  |  | ISPD |  |
|  |  | IST1 |  |
|  |  | MMP15 |  |
|  |  | MMP16 |  |
|  |  | MMP19 |  |
|  |  | MMP7 |  |
|  |  | MMRN1 |  |
|  |  | DUSP6 |  |
|  |  | MN1 |  |
|  |  | ORAOV1 |  |
|  |  | MNX1 |  |
|  |  | MOB1A |  |
|  |  | MOB1B |  |
|  |  | ORC5 |  |
|  |  | MOB4 |  |
|  |  | OSBPL1A |  |
|  |  | DYRK3 |  |
|  |  | DYX1C1 |  |
|  |  | DZIP3 |  |
|  |  | OSR2 |  |
|  |  | E2F2 |  |
|  |  | OSTM1 |  |
|  |  | MORN4 |  |
|  |  | OTOGL |  |
|  |  | OTUB2 |  |
|  |  | OTUD1 |  |
|  |  | OTUD3 |  |
|  |  | OTUD4 |  |
|  |  | OTUD6A |  |
|  |  | OTUD7A |  |
|  |  | OTUD7B |  |
|  |  | OTX2 |  |
|  |  | OXCT1 |  |
|  |  | MPPE1 |  |
|  |  | MPPED2 |  |
|  |  | MPRIP |  |
|  |  | MPV17L |  |
|  |  | MPZL2 |  |
|  |  | P2RX7 |  |
|  |  | P2RY1 |  |
|  |  | P2RY12 |  |
|  |  | P2RY13 |  |
|  |  | P2RY14 |  |
|  |  | P2RY2 |  |
|  |  | P2RY6 |  |
|  |  | P4HA1 |  |
|  |  | P4HA2 |  |
|  |  | PABPC1 |  |
|  |  | PABPC4L |  |
|  |  | PABPC5 |  |
|  |  | EFCAB6 |  |
|  |  | PACRGL |  |
|  |  | EFEMP1 |  |
|  |  | PACSIN1 |  |
|  |  | PADI4 |  |
|  |  | PAFAH1B1 |  |
|  |  | PAFAH2 |  |
|  |  | MRPS9 |  |
|  |  | EFNB3 |  |
|  |  | PAIP2 |  |
|  |  | PAIP2B |  |
|  |  | PAK1IP1 |  |
|  |  | PAK2 |  |
|  |  | PAK3 |  |
|  |  | EGR3 |  |
|  |  | EHD2 |  |
|  |  | EHD4 |  |
|  |  | EHF |  |
|  |  | EHMT1 |  |
|  |  | EID3 |  |
|  |  | EIF1 |  |
|  |  | EIF1B |  |
|  |  | EIF2AK1 |  |
|  |  | EIF2AK2 |  |
|  |  | EIF2AK3 |  |
|  |  | BAZ2B |  |
|  |  | BBC3 |  |
|  |  | BBIP1 |  |
|  |  | BBS1 |  |
|  |  | BBS10 |  |
|  |  | BBS2 |  |
|  |  | EIF4E |  |
|  |  | EIF4E3 |  |
|  |  | EIF4EBP2 |  |
|  |  | EIF4G2 |  |
|  |  | COL9A1 |  |
|  |  | COL9A3 |  |
|  |  | COLCA2 |  |
|  |  | EIF6 |  |
|  |  | ELAVL1 |  |
|  |  | ELAVL2 |  |
|  |  | ELFN1 |  |
|  |  | ELFN2 |  |
|  |  | ELK1 |  |
|  |  | ELK3 |  |
|  |  | ELK4 |  |
|  |  | ELL |  |
|  |  | ELL2 |  |
|  |  | ELMOD1 |  |
|  |  | ELMOD2 |  |
|  |  | ELN |  |
|  |  | ELOVL2 |  |
|  |  | ELOVL5 |  |
|  |  | ELOVL6 |  |
|  |  | ELOVL7 |  |
|  |  | ELP2 |  |
|  |  | ELP3 |  |
|  |  | ELP4 |  |
|  |  | ELP6 |  |
|  |  | ELTD1 |  |
|  |  | EMB |  |
|  |  | EMC1 |  |
|  |  | EMC2 |  |
|  |  | EMC7 |  |
|  |  | EMCN |  |
|  |  | EML1 |  |
|  |  | EML6 |  |
|  |  | EMP1 |  |
|  |  | EMP2 |  |
|  |  | ENAH |  |
|  |  | ENC1 |  |
|  |  | ENDOD1 |  |
|  |  | ENOX1 |  |
|  |  | ENPEP |  |
|  |  | ENPP1 |  |
|  |  | ENPP2 |  |
|  |  | ENPP3 |  |
|  |  | ENPP4 |  |
|  |  | ENPP5 |  |
|  |  | ENPP6 |  |
|  |  | ENSA |  |
|  |  | ENTPD1 |  |
|  |  | ENTPD4 |  |
|  |  | ENTPD5 |  |
|  |  | ENTPD6 |  |
|  |  | ENTPD7 |  |
|  |  | ENY2 |  |
|  |  | EOMES |  |
|  |  | EP300 |  |
|  |  | EP400 |  |
|  |  | EPAS1 |  |
|  |  | EPB41 |  |
|  |  | EPB41L1 |  |
|  |  | EPB41L2 |  |
|  |  | EPB41L3 |  |
|  |  | EPB41L4A |  |
|  |  | EPB41L5 |  |
|  |  | EPC1 |  |
|  |  | EPDR1 |  |
|  |  | EPG5 |  |
|  |  | EPHA4 |  |
|  |  | EPHA5 |  |
|  |  | EPHA6 |  |
|  |  | EPHA7 |  |
|  |  | EPM2A |  |
|  |  | EPM2AIP1 |  |
|  |  | EPN2 |  |
|  |  | EPPIN |  |
|  |  | EPPIN-WFDC6 |  |
|  |  | EPS8L2 |  |
|  |  | EPSTI1 |  |
|  |  | EPT1 |  |
|  |  | EPYC |  |
|  |  | ERAP1 |  |
|  |  | ERAP2 |  |
|  |  | ERBB2 |  |
|  |  | ERBB2IP |  |
|  |  | ERBB3 |  |
|  |  | ERBB4 |  |
|  |  | ERCC4 |  |
|  |  | ERCC6 |  |
|  |  | ERCC6L |  |
|  |  | ERCC6L2 |  |
|  |  | ERCC8 |  |
|  |  | EREG |  |
|  |  | ERGIC2 |  |
|  |  | ERI1 |  |
|  |  | ERI2 |  |
|  |  | ERLEC1 |  |
|  |  | ERLIN2 |  |
|  |  | ERMAP |  |
|  |  | ERMP1 |  |
|  |  | ERN1 |  |
|  |  | ERO1L |  |
|  |  | ERO1LB |  |
|  |  | ERP44 |  |
|  |  | ERVMER34-1 |  |
|  |  | ESCO1 |  |
|  |  | ESCO2 |  |
|  |  | ESD |  |
|  |  | ESF1 |  |
|  |  | ESRP1 |  |
|  |  | ESYT2 |  |
|  |  | C11orf70 |  |
|  |  | C11orf73 |  |
|  |  | C11orf74 |  |
|  |  | C11orf84 |  |
|  |  | C11orf87 |  |
|  |  | C12orf23 |  |
|  |  | C12orf36 |  |
|  |  | C12orf39 |  |
|  |  | C12orf44 |  |
|  |  | C12orf49 |  |
|  |  | C12orf5 |  |
|  |  | C12orf61 |  |
|  |  | C12orf66 |  |
|  |  | C12orf73 |  |
|  |  | C14orf142 |  |
|  |  | C14orf166 |  |
|  |  | C14orf37 |  |
|  |  | C14orf39 |  |
|  |  | C15orf38 |  |
|  |  | C15orf38-AP3S2 |  |
|  |  | C15orf53 |  |
|  |  | C15orf54 |  |
|  |  | C15orf56 |  |
|  |  | C16orf52 |  |
|  |  | C16orf62 |  |
|  |  | C16orf72 |  |
|  |  | C16orf87 |  |
|  |  | C16orf91 |  |
|  |  | C16orf93 |  |
|  |  | C17orf100 |  |
|  |  | C17orf102 |  |
|  |  | C17orf105 |  |
|  |  | C17orf51 |  |
|  |  | C17orf70 |  |
|  |  | C17orf89 |  |
|  |  | C17orf97 |  |
|  |  | C18orf21 |  |
|  |  | C18orf25 |  |
|  |  | C18orf32 |  |
|  |  | C18orf54 |  |
|  |  | C18orf56 |  |
|  |  | C18orf63 |  |
|  |  | C19orf12 |  |
|  |  | FAM102B |  |
|  |  | C19orf48 |  |
|  |  | FAM105A |  |
|  |  | FAM107B |  |
|  |  | FAM109A |  |
|  |  | FAM110C |  |
|  |  | FAM114A1 |  |
|  |  | FAM115C |  |
|  |  | FAM117B |  |
|  |  | FAM118A |  |
|  |  | FAM120A |  |
|  |  | FAM120AOS |  |
|  |  | FAM122A |  |
|  |  | FAM122B |  |
|  |  | PTGS2 |  |
|  |  | FAM124A |  |
|  |  | FAM126A |  |
|  |  | FAM126B |  |
|  |  | FAM129A |  |
|  |  | C1orf74 |  |
|  |  | FAM149B1 |  |
|  |  | C1QTNF9 |  |
|  |  | C1QTNF9B |  |
|  |  | C1RL |  |
|  |  | PTPN2 |  |
|  |  | FAM162B |  |
|  |  | CEP170 |  |
|  |  | CEP170B |  |
|  |  | CEP19 |  |
|  |  | C2CD4A |  |
|  |  | FAM172A |  |
|  |  | CEP44 |  |
|  |  | CEP55 |  |
|  |  | CEP57 |  |
|  |  | FAM175B |  |
|  |  | FAM179A |  |
|  |  | FAM179B |  |
|  |  | FAM181B |  |
|  |  | FAM184B |  |
|  |  | FAM185A |  |
|  |  | FAM188A |  |
|  |  | FAM192A |  |
|  |  | FAM196A |  |
|  |  | FAM198A |  |
|  |  | FAM199X |  |
|  |  | FAM19A1 |  |
|  |  | FAM19A2 |  |
|  |  | FAM19A4 |  |
|  |  | FAM203A |  |
|  |  | FAM203B |  |
|  |  | FAM205A |  |
|  |  | FAM206A |  |
|  |  | FAM20A |  |
|  |  | FAM20B |  |
|  |  | FAM211A |  |
|  |  | FAM213A |  |
|  |  | FAM213B |  |
|  |  | FAM214A |  |
|  |  | FAM216A |  |
|  |  | FAM217B |  |
|  |  | FAM221A |  |
|  |  | C6 |  |
|  |  | FAM227A |  |
|  |  | FAM229B |  |
|  |  | FAM26D |  |
|  |  | FAM26E |  |
|  |  | FAM3C |  |
|  |  | FAM45A |  |
|  |  | FAM46A |  |
|  |  | FAM47E-STBD1 |  |
|  |  | FAM49B |  |
|  |  | FAM58A |  |
|  |  | FAM60A |  |
|  |  | FAM63B |  |
|  |  | FAM69A |  |
|  |  | FAM71E2 |  |
|  |  | FAM71F2 |  |
|  |  | FAM72A |  |
|  |  | FAM72B |  |
|  |  | FAM72C |  |
|  |  | FAM72D |  |
|  |  | FAM73A |  |
|  |  | FAM76A |  |
|  |  | FAM76B |  |
|  |  | FAM78A |  |
|  |  | FAM83B |  |
|  |  | FAM83F |  |
|  |  | FAM84A |  |
|  |  | FAM84B |  |
|  |  | FAM86B1 |  |
|  |  | FAM9C |  |
|  |  | FAN1 |  |
|  |  | FANCA |  |
|  |  | FANCB |  |
|  |  | FANCC |  |
|  |  | FANCF |  |
|  |  | FANCM |  |
|  |  | FASN |  |
|  |  | FASTKD5 |  |
|  |  | FAT1 |  |
|  |  | FAT3 |  |
|  |  | FAT4 |  |
|  |  | FAXC |  |
|  |  | ARNT2 |  |
|  |  | FBN2 |  |
|  |  | FBXL17 |  |
|  |  | FBXL19 |  |
|  |  | FBXL20 |  |
|  |  | FBXL3 |  |
|  |  | FBXL4 |  |
|  |  | FBXL7 |  |
|  |  | FBXO10 |  |
|  |  | FBXO11 |  |
|  |  | FBXO21 |  |
|  |  | FBXO27 |  |
|  |  | FBXO28 |  |
|  |  | FBXO3 |  |
|  |  | FBXO30 |  |
|  |  | DNAJC25 |  |
|  |  | DNAJC25-GNG10 | |
|  |  | FBXW7 |  |
|  |  | FBXW8 |  |
|  |  | FCN2 |  |
|  |  | NXNL2 |  |
|  |  | NXPE1 |  |
|  |  | OARD1 |  |
|  |  | OCLM |  |
|  |  | OFCC1 |  |
|  |  | PPIL1 |  |
|  |  | OIP5 |  |
|  |  | OIT3 |  |
|  |  | OLFML2A |  |
|  |  | OMA1 |  |
|  |  | OPA1 |  |
|  |  | PPP1R13B |  |
|  |  | OPRK1 |  |
|  |  | PPP1R15B |  |
|  |  | PPP1R16A |  |
|  |  | PPP1R16B |  |
|  |  | PPP1R17 |  |
|  |  | OR2L13 |  |
|  |  | OR51M1 |  |
|  |  | FIGNL1 |  |
|  |  | PPP1R3A |  |
|  |  | OR7D2 |  |
|  |  | FKBP1A |  |
|  |  | ORMDL3 |  |
|  |  | OS9 |  |
|  |  | FKRP |  |
|  |  | OSBPL11 |  |
|  |  | OSBPL2 |  |
|  |  | FLJ27365 |  |
|  |  | FLRT2 |  |
|  |  | PPP2R5E |  |
|  |  | OSMR |  |
|  |  | ATP7B |  |
|  |  | ATP8A2 |  |
|  |  | ATP8B1 |  |
|  |  | ATP8B2 |  |
|  |  | DUSP8 |  |
|  |  | FOS |  |
|  |  | AURKA |  |
|  |  | AVEN |  |
|  |  | AWAT1 |  |
|  |  | AWAT2 |  |
|  |  | AXIN1 |  |
|  |  | PAK6 |  |
|  |  | B3GNT9 |  |
|  |  | B4GALNT3 |  |
|  |  | FRMPD4 |  |
|  |  | B4GALT6 |  |
|  |  | BAALC |  |
|  |  | BAAT |  |
|  |  | BACE2 |  |
|  |  | BACH1 |  |
|  |  | BACH2 |  |
|  |  | BAG1 |  |
|  |  | BAG2 |  |
|  |  | BAG4 |  |
|  |  | BAIAP2L1 |  |
|  |  | BAK1 |  |
|  |  | BAMBI |  |
|  |  | BAP1 |  |
|  |  | BAZ1A |  |
|  |  | BAZ2A |  |
|  |  | BBS4 |  |
|  |  | FUT9 |  |
|  |  | FXR1 |  |
|  |  | BCAP29 |  |
|  |  | BCAS3 |  |
|  |  | BCAT1 |  |
|  |  | B4GALNT2 |  |
|  |  | B4GALT4 |  |
|  |  | BCL2 |  |
|  |  | BCL2A1 |  |
|  |  | BCL2L11 |  |
|  |  | BCL2L13 |  |
|  |  | BCL2L14 |  |
|  |  | BCL2L2 |  |
|  |  | BCL6 |  |
|  |  | PSMB4 |  |
|  |  | PSMB9 |  |
|  |  | BCLAF1 |  |
|  |  | BCO2 |  |
|  |  | BDNF |  |
|  |  | BDP1 |  |
|  |  | BEAN1 |  |
|  |  | GABRB2 |  |
|  |  | BEGAIN |  |
|  |  | BEND2 |  |
|  |  | GABRG2 |  |
|  |  | GAD1 |  |
|  |  | GAD2 |  |
|  |  | GALM |  |
|  |  | ELMO1 |  |
|  |  | ELMSAN1 |  |
|  |  | PTCHD4 |  |
|  |  | PCDHA6 |  |
|  |  | BHLHE41 |  |
|  |  | BICC1 |  |
|  |  | GAPT |  |
|  |  | GAPVD1 |  |
|  |  | GAR1 |  |
|  |  | GART |  |
|  |  | GAS2L3 |  |
|  |  | GATA2 |  |
|  |  | GATA3 |  |
|  |  | PCNXL2 |  |
|  |  | PCSK2 |  |
|  |  | BMP6 |  |
|  |  | BMP7 |  |
|  |  | BMPR1A |  |
|  |  | GBA |  |
|  |  | BMPR2 |  |
|  |  | GBE1 |  |
|  |  | BNC2 |  |
|  |  | BNIP2 |  |
|  |  | PDCL |  |
|  |  | GCC2 |  |
|  |  | PTPN9 |  |
|  |  | GCLM |  |
|  |  | GCM2 |  |
|  |  | BPIFC |  |
|  |  | BRCC3 |  |
|  |  | GCOM1 |  |
|  |  | GCSAM |  |
|  |  | BRD3 |  |
|  |  | GDA |  |
|  |  | BRD8 |  |
|  |  | BRD9 |  |
|  |  | BRI3BP |  |
|  |  | BRINP1 |  |
|  |  | GDF7 |  |
|  |  | BRMS1L |  |
|  |  | GDI2 |  |
|  |  | GDPD1 |  |
|  |  | GDPGP1 |  |
|  |  | GEMIN5 |  |
|  |  | GET4 |  |
|  |  | EPHB2 |  |
|  |  | GFPT1 |  |
|  |  | GFRA1 |  |
|  |  | GFRA2 |  |
|  |  | EPS15 |  |
|  |  | GGT6 |  |
|  |  | GHR |  |
|  |  | GID4 |  |
|  |  | GIGYF1 |  |
|  |  | GIMAP1 |  |
|  |  | GIN1 |  |
|  |  | BVES |  |
|  |  | BX255923.1 |  |
|  |  | BYSL |  |
|  |  | GINS4 |  |
|  |  | BZW2 |  |
|  |  | C10orf10 |  |
|  |  | C10orf107 |  |
|  |  | C10orf11 |  |
|  |  | GJA9 |  |
|  |  | GJB1 |  |
|  |  | FAM122C |  |
|  |  | GJC1 |  |
|  |  | GJC2 |  |
|  |  | GJD2 |  |
|  |  | GK |  |
|  |  | GK5 |  |
|  |  | GLA |  |
|  |  | GLCCI1 |  |
|  |  | GLCE |  |
|  |  | CTSO |  |
|  |  | CTSV |  |
|  |  | GLO1 |  |
|  |  | FAM163A |  |
|  |  | FAM168A |  |
|  |  | FAM169A |  |
|  |  | CTTN |  |
|  |  | CTTNBP2 |  |
|  |  | CTTNBP2NL |  |
|  |  | FAM173B |  |
|  |  | C2orf54 |  |
|  |  | C2orf69 |  |
|  |  | C14orf93 |  |
|  |  | C15orf39 |  |
|  |  | GMCL1 |  |
|  |  | C15orf61 |  |
|  |  | GMNC |  |
|  |  | GMPPB |  |
|  |  | RALA |  |
|  |  | RALB |  |
|  |  | GNAT1 |  |
|  |  | C18orf42 |  |
|  |  | C19orf10 |  |
|  |  | C19orf26 |  |
|  |  | C19orf44 |  |
|  |  | C19orf59 |  |
|  |  | C19orf73 |  |
|  |  | C1D |  |
|  |  | GOLGA3 |  |
|  |  | GOLGA4 |  |
|  |  | GOLGA6A |  |
|  |  | GOLGA6B |  |
|  |  | GOLGA6D |  |
|  |  | GOLGA6L10 |  |
|  |  | GOLGA6L4 |  |
|  |  | GOLGA6L9 |  |
|  |  | GOLGA7B |  |
|  |  | GOLGA8A |  |
|  |  | GOLGA8B |  |
|  |  | GOLGA8F |  |
|  |  | GOLGA8G |  |
|  |  | GOLGA8H |  |
|  |  | GOLGA8I |  |
|  |  | GOLGA8K |  |
|  |  | GOLGA8N |  |
|  |  | GOLGA8O |  |
|  |  | GOLM1 |  |
|  |  | GOLPH3 |  |
|  |  | GOT2 |  |
|  |  | GPAM |  |
|  |  | GPATCH2 |  |
|  |  | GPATCH2L |  |
|  |  | GPBP1 |  |
|  |  | GPC4 |  |
|  |  | GPC6 |  |
|  |  | FAM181A |  |
|  |  | GPD2 |  |
|  |  | GPI |  |
|  |  | GPKOW |  |
|  |  | GPM6A |  |
|  |  | GPN3 |  |
|  |  | FAM198B |  |
|  |  | GPR116 |  |
|  |  | FAM204A |  |
|  |  | GPR144 |  |
|  |  | GPR146 |  |
|  |  | GPR17 |  |
|  |  | GPR180 |  |
|  |  | GPR19 |  |
|  |  | GPR27 |  |
|  |  | GPR31 |  |
|  |  | SMN1 |  |
|  |  | GPR37 |  |
|  |  | GPR39 |  |
|  |  | GPR50 |  |
|  |  | GPR56 |  |
|  |  | GPR6 |  |
|  |  | GPR75 |  |
|  |  | FAM53C |  |
|  |  | GPR83 |  |
|  |  | GPR89A |  |
|  |  | FAM65B |  |
|  |  | TMEM167B |  |
|  |  | FAM83D |  |
|  |  | GRIA1 |  |
|  |  | CCDC30 |  |
|  |  | SNURF |  |
|  |  | CCDC38 |  |
|  |  | TMEM182 |  |
|  |  | TMEM183A |  |
|  |  | GRIK4 |  |
|  |  | FANCL |  |
|  |  | FAP |  |
|  |  | FAR1 |  |
|  |  | CCDC71 |  |
|  |  | GRM7 |  |
|  |  | FARSB |  |
|  |  | FASLG |  |
|  |  | FASTKD2 |  |
|  |  | FASTKD3 |  |
|  |  | GSPT2 |  |
|  |  | GSR |  |
|  |  | GSTA2 |  |
|  |  | GSTM3 |  |
|  |  | GSTM4 |  |
|  |  | GTDC1 |  |
|  |  | GTF2A1 |  |
|  |  | FBXL16 |  |
|  |  | GTF2B |  |
|  |  | GTF2H1 |  |
|  |  | GTF3C4 |  |
|  |  | GTPBP8 |  |
|  |  | GTSE1 |  |
|  |  | GUCA1A |  |
|  |  | GUCY1A2 |  |
|  |  | GUCY1B3 |  |
|  |  | GUF1 |  |
|  |  | GULP1 |  |
|  |  | GXYLT1 |  |
|  |  | GXYLT2 |  |
|  |  | GYPB |  |
|  |  | GYS2 |  |
|  |  | GZF1 |  |
|  |  | H2AFV |  |
|  |  | H3F3B |  |
|  |  | HACE1 |  |
|  |  | HARS2 |  |
|  |  | HAS1 |  |
|  |  | HAS2 |  |
|  |  | HAS3 |  |
|  |  | HAT1 |  |
|  |  | HAUS6 |  |
|  |  | HAVCR1 |  |
|  |  | HBS1L |  |
|  |  | HCAR1 |  |
|  |  | HCFC2 |  |
|  |  | HCN1 |  |
|  |  | FER1L6 |  |
|  |  | TMTC4 |  |
|  |  | FEZ2 |  |
|  |  | FGA |  |
|  |  | HDAC9 |  |
|  |  | HDGFRP3 |  |
|  |  | HDHD1 |  |
|  |  | HDHD2 |  |
|  |  | HEBP2 |  |
|  |  | HECA |  |
|  |  | CCDC132 |  |
|  |  | CCDC134 |  |
|  |  | FGF9 |  |
|  |  | FGFR1OP2 |  |
|  |  | FGG |  |
|  |  | FGL2 |  |
|  |  | FHL1 |  |
|  |  | FHL2 |  |
|  |  | FHL5 |  |
|  |  | CCDC22 |  |
|  |  | CCDC25 |  |
|  |  | CCDC3 |  |
|  |  | CCDC34 |  |
|  |  | CCDC36 |  |
|  |  | CCDC39 |  |
|  |  | HIF1A |  |
|  |  | HIF1AN |  |
|  |  | FLNA |  |
|  |  | FLRT3 |  |
|  |  | HIST1H2BJ |  |
|  |  | FLVCR2 |  |
|  |  | HIST1H3E |  |
|  |  | HIST1H3F |  |
|  |  | FMO2 |  |
|  |  | FMR1 |  |
|  |  | HJURP |  |
|  |  | HLA-A |  |
|  |  | HLA-DOA |  |
|  |  | HLA-DQA1 |  |
|  |  | FNDC8 |  |
|  |  | HLF |  |
|  |  | HLTF |  |
|  |  | FOPNL |  |
|  |  | FOSL2 |  |
|  |  | HMG20A |  |
|  |  | HMGA2 |  |
|  |  | FOXC1 |  |
|  |  | FOXJ3 |  |
|  |  | FOXL2 |  |
|  |  | HN1 |  |
|  |  | HNF1B |  |
|  |  | FOXO1 |  |
|  |  | HNF4G |  |
|  |  | HNRNPA0 |  |
|  |  | HNRNPA1 |  |
|  |  | FOXP2 |  |
|  |  | FOXP4 |  |
|  |  | FOXRED2 |  |
|  |  | FPGT |  |
|  |  | FRA10AC1 |  |
|  |  | FRAS1 |  |
|  |  | FREM1 |  |
|  |  | FREM2 |  |
|  |  | FRK |  |
|  |  | FRMD3 |  |
|  |  | FRMD4A |  |
|  |  | FRMD4B |  |
|  |  | FRMD6 |  |
|  |  | HOXA7 |  |
|  |  | FRRS1 |  |
|  |  | FRRS1L |  |
|  |  | HOXD11 |  |
|  |  | HOXD12 |  |
|  |  | HOXD13 |  |
|  |  | HOXD4 |  |
|  |  | HPGD |  |
|  |  | HPS1 |  |
|  |  | HPS4 |  |
|  |  | HPX |  |
|  |  | HRASLS5 |  |
|  |  | HRH1 |  |
|  |  | HRK |  |
|  |  | HRNR |  |
|  |  | HS2ST1 |  |
|  |  | HS3ST1 |  |
|  |  | HS6ST1 |  |
|  |  | FUT1 |  |
|  |  | FUT2 |  |
|  |  | FUT4 |  |
|  |  | FUT8 |  |
|  |  | FYCO1 |  |
|  |  | FYN |  |
|  |  | FYTTD1 |  |
|  |  | FZD1 |  |
|  |  | FZD3 |  |
|  |  | FZD4 |  |
|  |  | FZD5 |  |
|  |  | FZD6 |  |
|  |  | FZD7 |  |
|  |  | FZD8 |  |
|  |  | G3BP1 |  |
|  |  | G3BP2 |  |
|  |  | G6PC |  |
|  |  | G6PC2 |  |
|  |  | G6PC3 |  |
|  |  | GAB1 |  |
|  |  | GAB2 |  |
|  |  | GABARAPL2 |  |
|  |  | GABBR1 |  |
|  |  | GABBR2 |  |
|  |  | HTT |  |
|  |  | GABRA1 |  |
|  |  | GABRA2 |  |
|  |  | GABRA4 |  |
|  |  | GABRB1 |  |
|  |  | GABRB3 |  |
|  |  | GABRG1 |  |
|  |  | GABRQ |  |
|  |  | GABRR1 |  |
|  |  | ID4 |  |
|  |  | GALC |  |
|  |  | TSR2 |  |
|  |  | IER2 |  |
|  |  | GALNT12 |  |
|  |  | IFFO2 |  |
|  |  | IFIT1 |  |
|  |  | IFIT2 |  |
|  |  | IFIT5 |  |
|  |  | IFNA8 |  |
|  |  | IFNAR1 |  |
|  |  | IFNE |  |
|  |  | IFRD1 |  |
|  |  | GALNT9 |  |
|  |  | IFT57 |  |
|  |  | IFT74 |  |
|  |  | IFT80 |  |
|  |  | IGDCC3 |  |
|  |  | IGDCC4 |  |
|  |  | IGF1 |  |
|  |  | IGF2BP1 |  |
|  |  | IGF2BP3 |  |
|  |  | IGF2R |  |
|  |  | IGFBP5 |  |
|  |  | IGFBP7 |  |
|  |  | IGFBPL1 |  |
|  |  | IGIP |  |
|  |  | IGSF10 |  |
|  |  | GCH1 |  |
|  |  | GCNT2 |  |
|  |  | IL13 |  |
|  |  | GCNT4 |  |
|  |  | IL16 |  |
|  |  | IL17RA |  |
|  |  | IL1A |  |
|  |  | IL1RAP |  |
|  |  | IL1RAPL1 |  |
|  |  | IL7R |  |
|  |  | IMMT |  |
|  |  | IMP4 |  |
|  |  | IMPA1 |  |
|  |  | IMPG2 |  |
|  |  | INA |  |
|  |  | INCENP |  |
|  |  | INO80D |  |
|  |  | INPP4B |  |
|  |  | INPP5D |  |
|  |  | INPP5F |  |
|  |  | GLIPR1 |  |
|  |  | INSR |  |
|  |  | INTS6 |  |
|  |  | INTS8 |  |
|  |  | INTU |  |
|  |  | GLRX5 |  |
|  |  | IPO4 |  |
|  |  | GLTSCR1 |  |
|  |  | IPO7 |  |
|  |  | IPO8 |  |
|  |  | GLUL |  |
|  |  | GLYCTK |  |
|  |  | IPPK |  |
|  |  | IQCA1 |  |
|  |  | IQCB1 |  |
|  |  | IQGAP1 |  |
|  |  | IQGAP2 |  |
|  |  | IQGAP3 |  |
|  |  | IQSEC1 |  |
|  |  | IRAK1BP1 |  |
|  |  | IRAK3 |  |
|  |  | IREB2 |  |
|  |  | IRF1 |  |
|  |  | IRF2BP2 |  |
|  |  | IRF6 |  |
|  |  | IRS1 |  |
|  |  | GNB4 |  |
|  |  | UGCG |  |
|  |  | ISCU |  |
|  |  | ISOC1 |  |
|  |  | DUS2 |  |
|  |  | DUSP1 |  |
|  |  | GNPNAT1 |  |
|  |  | GNPTAB |  |
|  |  | GNS |  |
|  |  | ITGA1 |  |
|  |  | ITGA2 |  |
|  |  | ITGA3 |  |
|  |  | ITGA4 |  |
|  |  | UNC45B |  |
|  |  | GOLGA8J |  |
|  |  | GOLGA8R |  |
|  |  | GOLGB1 |  |
|  |  | GOLIM4 |  |
|  |  | GOLPH3L |  |
|  |  | GOLT1B |  |
|  |  | URGCP |  |
|  |  | GOPC |  |
|  |  | CELF1 |  |
|  |  | GORASP2 |  |
|  |  | GPATCH11 |  |
|  |  | GPATCH4 |  |
|  |  | CELSR2 |  |
|  |  | CENPA |  |
|  |  | CENPK |  |
|  |  | CENPL |  |
|  |  | CENPQ |  |
|  |  | CEP128 |  |
|  |  | GPR15 |  |
|  |  | CEP72 |  |
|  |  | THAP2 |  |
|  |  | GPR63 |  |
|  |  | GPR64 |  |
|  |  | GPRC5B |  |
|  |  | VKORC1L1 |  |
|  |  | VLDLR |  |
|  |  | VMA21 |  |
|  |  | VMP1 |  |
|  |  | VNN1 |  |
|  |  | THUMPD1 |  |
|  |  | VPS13A |  |
|  |  | VPS13B |  |
|  |  | CERS3 |  |
|  |  | CERS5 |  |
|  |  | GRIP1 |  |
|  |  | GRK5 |  |
|  |  | GRM3 |  |
|  |  | VWC2 |  |
|  |  | TLL1 |  |
|  |  | GSTK1 |  |
|  |  | GSTO2 |  |
|  |  | GSX1 |  |
|  |  | WDFY2 |  |
|  |  | WDFY3 |  |
|  |  | WDHD1 |  |
|  |  | WDR1 |  |
|  |  | WDR12 |  |
|  |  | GTPBP1 |  |
|  |  | GTPBP10 |  |
|  |  | GTPBP4 |  |
|  |  | GUCA2A |  |
|  |  | WDR37 |  |
|  |  | GZMB |  |
|  |  | WDR72 |  |
|  |  | H2AFY |  |
|  |  | HABP4 |  |
|  |  | HAO1 |  |
|  |  | HAP1 |  |
|  |  | WDYHV1 |  |
|  |  | HAUS1 |  |
|  |  | HAUS2 |  |
|  |  | HBP1 |  |
|  |  | HCCS |  |
|  |  | HCN3 |  |
|  |  | WNK3 |  |
|  |  | WNT16 |  |
|  |  | HDX |  |
|  |  | TMEM170A |  |
|  |  | CFC1 |  |
|  |  | CFC1B |  |
|  |  | TMEM178B |  |
|  |  | WTAP |  |
|  |  | TMEM181 |  |
|  |  | HECW2 |  |
|  |  | HELLS |  |
|  |  | TMEM184C |  |
|  |  | HEMGN |  |
|  |  | HEPACAM |  |
|  |  | HEPHL1 |  |
|  |  | CFHR2 |  |
|  |  | HERC4 |  |
|  |  | HES5 |  |
|  |  | HES6 |  |
|  |  | HIAT1 |  |
|  |  | HIATL1 |  |
|  |  | TMEM251 |  |
|  |  | HIGD1A |  |
|  |  | HIP1 |  |
|  |  | TMEM30B |  |
|  |  | HIST1H2AC |  |
|  |  | HIST1H2BO |  |
|  |  | HIST1H3D |  |
|  |  | HIST1H4H |  |
|  |  | HK2 |  |
|  |  | HLA-B |  |
|  |  | HLA-C |  |
|  |  | HLA-E |  |
|  |  | HLCS |  |
|  |  | HMCN1 |  |
|  |  | HMGCLL1 |  |
|  |  | HMGN4 |  |
|  |  | HMGXB3 |  |
|  |  | HMX1 |  |
|  |  | HNRNPA1L2 |  |
|  |  | HNRNPK |  |
|  |  | ZC3H12C |  |
|  |  | HOPX |  |
|  |  | ZC3H7A |  |
|  |  | HOXA13 |  |
|  |  | HOXB2 |  |
|  |  | HOXB3 |  |
|  |  | HOXB7 |  |
|  |  | ZCCHC24 |  |
|  |  | HOXC12 |  |
|  |  | HOXD8 |  |
|  |  | HP1BP3 |  |
|  |  | HPRT1 |  |
|  |  | HPSE |  |
|  |  | HR |  |
|  |  | HRAS |  |
|  |  | ZDHHC9 |  |
|  |  | ZEB1 |  |
|  |  | HS3ST5 |  |
|  |  | HSD17B11 |  |
|  |  | HSF2BP |  |
|  |  | HSF5 |  |
|  |  | LCT |  |
|  |  | LDHA |  |
|  |  | LDLR |  |
|  |  | HSPA2 |  |
|  |  | HSPD1 |  |
|  |  | LEPROT |  |
|  |  | LEPROTL1 |  |
|  |  | LETM2 |  |
|  |  | LETMD1 |  |
|  |  | LGALS8 |  |
|  |  | LGALSL |  |
|  |  | LGI2 |  |
|  |  | LGI3 |  |
|  |  | LGR4 |  |
|  |  | LHFP |  |
|  |  | HYKK |  |
|  |  | LHX1 |  |
|  |  | LHX2 |  |
|  |  | LHX4 |  |
|  |  | LHX6 |  |
|  |  | LILRA1 |  |
|  |  | LIMD1 |  |
|  |  | IDH1 |  |
|  |  | LIN28A |  |
|  |  | ZNF12 |  |
|  |  | LIN7A |  |
|  |  | LIN7C |  |
|  |  | LIN9 |  |
|  |  | LINC00998 |  |
|  |  | LIPF |  |
|  |  | LIX1 |  |
|  |  | LL22NC03-63E9.3 | |
|  |  | LLPH |  |
|  |  | LMAN1 |  |
|  |  | LMBR1 |  |
|  |  | LMBRD1 |  |
|  |  | LMBRD2 |  |
|  |  | IGFBP1 |  |
|  |  | LMNB2 |  |
|  |  | LMO3 |  |
|  |  | LMO7 |  |
|  |  | LMTK2 |  |
|  |  | LMX1A |  |
|  |  | IKBKG |  |
|  |  | LONP2 |  |
|  |  | LONRF1 |  |
|  |  | LONRF2 |  |
|  |  | LONRF3 |  |
|  |  | LPAR1 |  |
|  |  | LPAR4 |  |
|  |  | FBLN7 |  |
|  |  | FBN1 |  |
|  |  | CHORDC1 |  |
|  |  | LPL |  |
|  |  | LPP |  |
|  |  | LPPR4 |  |
|  |  | LPPR5 |  |
|  |  | CHRDL1 |  |
|  |  | CHRFAM7A |  |
|  |  | CHRM3 |  |
|  |  | CHRM5 |  |
|  |  | LRIG3 |  |
|  |  | LRP12 |  |
|  |  | LRP2BP |  |
|  |  | LRP6 |  |
|  |  | LRP8 |  |
|  |  | LRPAP1 |  |
|  |  | CHST6 |  |
|  |  | CHSY1 |  |
|  |  | CHSY3 |  |
|  |  | CHURC1 |  |
|  |  | LRRC27 |  |
|  |  | FCAR |  |
|  |  | FCHSD2 |  |
|  |  | LRRC39 |  |
|  |  | DNER |  |
|  |  | DNLZ |  |
|  |  | FECH |  |
|  |  | FEM1A |  |
|  |  | DNMBP |  |
|  |  | FEM1C |  |
|  |  | FER |  |
|  |  | FETUB |  |
|  |  | FGB |  |
|  |  | FGD3 |  |
|  |  | FGD4 |  |
|  |  | FGD6 |  |
|  |  | FGF10 |  |
|  |  | FGF12 |  |
|  |  | FGF14 |  |
|  |  | FGF2 |  |
|  |  | FGF23 |  |
|  |  | FGFBP3 |  |
|  |  | DPP4 |  |
|  |  | LTBP4 |  |
|  |  | FGFRL1 |  |
|  |  | DPYSL3 |  |
|  |  | FICD |  |
|  |  | FIG4 |  |
|  |  | FIGN |  |
|  |  | LYN |  |
|  |  | FILIP1 |  |
|  |  | FILIP1L |  |
|  |  | FIP1L1 |  |
|  |  | FKBP14 |  |
|  |  | FKBP15 |  |
|  |  | LYRM7 |  |
|  |  | FKBP7 |  |
|  |  | FKBP9 |  |
|  |  | LZIC |  |
|  |  | FKTN |  |
|  |  | FLG2 |  |
|  |  | FLI1 |  |
|  |  | MACC1 |  |
|  |  | MAD2L1 |  |
|  |  | FLVCR1 |  |
|  |  | FLYWCH2 |  |
|  |  | FMN1 |  |
|  |  | DUS4L |  |
|  |  | DUSP10 |  |
|  |  | DUSP16 |  |
|  |  | FN1 |  |
|  |  | FNBP1 |  |
|  |  | FNBP1L |  |
|  |  | FNBP4 |  |
|  |  | MAML2 |  |
|  |  | DVL1 |  |
|  |  | FNIP1 |  |
|  |  | MAN1A1 |  |
|  |  | FOXA3 |  |
|  |  | DYNC1LI2 |  |
|  |  | DYNC2LI1 |  |
|  |  | DYNLL2 |  |
|  |  | DYNLT1 |  |
|  |  | DYRK1A |  |
|  |  | DZIP1 |  |
|  |  | E2F3 |  |
|  |  | E2F8 |  |
|  |  | FOXP1 |  |
|  |  | FPR2 |  |
|  |  | FREM3 |  |
|  |  | FRG2C |  |
|  |  | EDC3 |  |
|  |  | FRMPD2 |  |
|  |  | MAPK13 |  |
|  |  | FRS2 |  |
|  |  | FRY |  |
|  |  | FRYL |  |
|  |  | FSBP |  |
|  |  | FSD1L |  |
|  |  | FSHR |  |
|  |  | EFCAB7 |  |
|  |  | FTO |  |
|  |  | FTSJ1 |  |
|  |  | EFNB2 |  |
|  |  | FUCA2 |  |
|  |  | EGFL6 |  |
|  |  | EGLN1 |  |
|  |  | MASP1 |  |
|  |  | MAST3 |  |
|  |  | G2E3 |  |
|  |  | GAB3 |  |
|  |  | GABPA |  |
|  |  | GABPB2 |  |
|  |  | GABRA6 |  |
|  |  | MCIDAS |  |
|  |  | MCM6 |  |
|  |  | MCMBP |  |
|  |  | MCMDC2 |  |
|  |  | MCTP1 |  |
|  |  | MCUR1 |  |
|  |  | MDFIC |  |
|  |  | ELOVL4 |  |
|  |  | MED1 |  |
|  |  | MED10 |  |
|  |  | MED12L |  |
|  |  | MED13 |  |
|  |  | MED14 |  |
|  |  | MED17 |  |
|  |  | MED19 |  |
|  |  | MED20 |  |
|  |  | MED21 |  |
|  |  | MED26 |  |
|  |  | GATA6 |  |
|  |  | GATAD1 |  |
|  |  | GATAD2A |  |
|  |  | GATC |  |
|  |  | MEDAG |  |
|  |  | EN2 |  |
|  |  | MEF2C |  |
|  |  | MEF2D |  |
|  |  | GBP4 |  |
|  |  | TBC1D19 |  |
|  |  | MEIS2 |  |
|  |  | MELK |  |
|  |  | MEMO1 |  |
|  |  | BPTF |  |
|  |  | BRCA1 |  |
|  |  | PTPRK |  |
|  |  | BRD4 |  |
|  |  | EOGT |  |
|  |  | BRIP1 |  |
|  |  | PDGFA |  |
|  |  | BRPF1 |  |
|  |  | EPC2 |  |
|  |  | PDHX |  |
|  |  | BRWD1 |  |
|  |  | BRWD3 |  |
|  |  | BSDC1 |  |
|  |  | BSN |  |
|  |  | BSND |  |
|  |  | PVRL3 |  |
|  |  | PWP1 |  |
|  |  | BTBD18 |  |
|  |  | BTBD3 |  |
|  |  | PYGO1 |  |
|  |  | BTC |  |
|  |  | PYROXD1 |  |
|  |  | BTF3L4 |  |
|  |  | QSER1 |  |
|  |  | BTK |  |
|  |  | R3HCC1L |  |
|  |  | BTRC |  |
|  |  | BUB3 |  |
|  |  | ERG |  |
|  |  | BZW1 |  |
|  |  | ERMN |  |
|  |  | C10orf118 |  |
|  |  | C10orf12 |  |
|  |  | C10orf54 |  |
|  |  | C10orf67 |  |
|  |  | C10orf71 |  |
|  |  | C11orf30 |  |
|  |  | C11orf53 |  |
|  |  | C11orf58 |  |
|  |  | PGD |  |
|  |  | C11orf82 |  |
|  |  | C12orf4 |  |
|  |  | C12orf50 |  |
|  |  | C13orf45 |  |
|  |  | C14orf23 |  |
|  |  | GM2A |  |
|  |  | GMEB1 |  |
|  |  | GMFB |  |
|  |  | GMPR |  |
|  |  | GMPS |  |
|  |  | GNA13 |  |
|  |  | GNA14 |  |
|  |  | GNAI1 |  |
|  |  | GNAI3 |  |
|  |  | GNAL |  |
|  |  | GNAQ |  |
|  |  | A2M |  |
|  |  | GNB1 |  |
|  |  | GNE |  |
|  |  | GNG2 |  |
|  |  | GNL1 |  |
|  |  | GNPDA1 |  |
|  |  | GNPDA2 |  |
|  |  | GNPTG |  |
|  |  | GOLGA2 |  |
|  |  | MPO |  |
|  |  | MPP2 |  |
|  |  | MPP3 |  |
|  |  | CMIP |  |
|  |  | MPP6 |  |
|  |  | MPP7 |  |
|  |  | MPZL1 |  |
|  |  | MR1 |  |
|  |  | MRC1 |  |
|  |  | MRC1L1 |  |
|  |  | MRE11A |  |
|  |  | MREG |  |
|  |  | MRGBP |  |
|  |  | MRM1 |  |
|  |  | MROH2A |  |
|  |  | MRPL17 |  |
|  |  | MRPL18 |  |
|  |  | MRPL19 |  |
|  |  | MRPL22 |  |
|  |  | MRPL27 |  |
|  |  | MRPL3 |  |
|  |  | CMPK1 |  |
|  |  | MRPL34 |  |
|  |  | MRPL35 |  |
|  |  | MRPL36 |  |
|  |  | MRPL4 |  |
|  |  | MRPL42 |  |
|  |  | MRPL50 |  |
|  |  | MRPS10 |  |
|  |  | MRPS11 |  |
|  |  | MRPS18A |  |
|  |  | MRPS18C |  |
|  |  | MRPS23 |  |
|  |  | MRPS25 |  |
|  |  | MRPS26 |  |
|  |  | MRPS27 |  |
|  |  | MRPS30 |  |
|  |  | CMSS1 |  |
|  |  | MRRF |  |
|  |  | MRS2 |  |
|  |  | MRVI1 |  |
|  |  | MS4A1 |  |
|  |  | MS4A15 |  |
|  |  | MSANTD2 |  |
|  |  | MSANTD3-TMEFF1 | |
|  |  | CMTM4 |  |
|  |  | CMTM6 |  |
|  |  | MSH6 |  |
|  |  | MSI1 |  |
|  |  | MSI2 |  |
|  |  | MSL2 |  |
|  |  | MSL3 |  |
|  |  | CMYA5 |  |
|  |  | MSRB1 |  |
|  |  | MSRB3 |  |
|  |  | MST4 |  |
|  |  | MSTN |  |
|  |  | MSX1 |  |
|  |  | MT1A |  |
|  |  | MTA3 |  |
|  |  | MTBP |  |
|  |  | MTCH2 |  |
|  |  | MTDH |  |
|  |  | MTFR1 |  |
|  |  | MTFR1L |  |
|  |  | MTHFD2 |  |
|  |  | MTHFR |  |
|  |  | MTIF2 |  |
|  |  | MTL5 |  |
|  |  | MTMR1 |  |
|  |  | MTMR10 |  |
|  |  | MTMR12 |  |
|  |  | MTMR2 |  |
|  |  | MTMR3 |  |
|  |  | MTMR4 |  |
|  |  | MTMR6 |  |
|  |  | MTMR7 |  |
|  |  | MTMR9 |  |
|  |  | MT-ND4L |  |
|  |  | MTOR |  |
|  |  | MTPAP |  |
|  |  | MTPN |  |
|  |  | MTR |  |
|  |  | MTRF1 |  |
|  |  | MTRF1L |  |
|  |  | MTRR |  |
|  |  | MTSS1L |  |
|  |  | MTTP |  |
|  |  | MTX3 |  |
|  |  | MUC19 |  |
|  |  | MUM1 |  |
|  |  | MUM1L1 |  |
|  |  | MUT |  |
|  |  | MX2 |  |
|  |  | MXD1 |  |
|  |  | MXD3 |  |
|  |  | CNEP1R1 |  |
|  |  | MXI1 |  |
|  |  | MXRA5 |  |
|  |  | MXRA7 |  |
|  |  | MYADM |  |
|  |  | MYB |  |
|  |  | MYBL1 |  |
|  |  | MYCBP |  |
|  |  | MYCN |  |
|  |  | MYEF2 |  |
|  |  | MYEOV |  |
|  |  | MYH10 |  |
|  |  | MYL2 |  |
|  |  | CNGA1 |  |
|  |  | CNGB3 |  |
|  |  | ZFP91 |  |
|  |  | MYO10 |  |
|  |  | MYO15A |  |
|  |  | CNIH1 |  |
|  |  | CNIH3 |  |
|  |  | MYO5A |  |
|  |  | MYO5B |  |
|  |  | ZFYVE9 |  |
|  |  | MYO9A |  |
|  |  | CNKSR2 |  |
|  |  | CNKSR3 |  |
|  |  | MYOZ1 |  |
|  |  | ZIC5 |  |
|  |  | ZIK1 |  |
|  |  | ZKSCAN1 |  |
|  |  | ZKSCAN2 |  |
|  |  | ZKSCAN8 |  |
|  |  | ZMAT2 |  |
|  |  | ZMAT3 |  |
|  |  | MZF1 |  |
|  |  | N4BP1 |  |
|  |  | N4BP2 |  |
|  |  | N4BP2L2 |  |
|  |  | CNOT1 |  |
|  |  | CNOT11 |  |
|  |  | CNOT2 |  |
|  |  | CNOT4 |  |
|  |  | CNOT6 |  |
|  |  | ZNF112 |  |
|  |  | CNOT7 |  |
|  |  | NAAA |  |
|  |  | NAALAD2 |  |
|  |  | CNP |  |
|  |  | NAB1 |  |
|  |  | NABP1 |  |
|  |  | NACA |  |
|  |  | CNPY1 |  |
|  |  | ZNF160 |  |
|  |  | NACC2 |  |
|  |  | CNR1 |  |
|  |  | NAGLU |  |
|  |  | NAIF1 |  |
|  |  | NAIP |  |
|  |  | NALCN |  |
|  |  | LRRTM3 |  |
|  |  | CNRIP1 |  |
|  |  | NANOS1 |  |
|  |  | NAP1L1 |  |
|  |  | NAP1L5 |  |
|  |  | NAP1L6 |  |
|  |  | NAPEPLD |  |
|  |  | CNTD1 |  |
|  |  | NARS |  |
|  |  | NAT14 |  |
|  |  | NAT16 |  |
|  |  | NAT8L |  |
|  |  | NAV1 |  |
|  |  | NAV2 |  |
|  |  | NAV3 |  |
|  |  | NBEAL1 |  |
|  |  | NBN |  |
|  |  | HGSNAT |  |
|  |  | HHLA2 |  |
|  |  | NCL |  |
|  |  | NCOA2 |  |
|  |  | NCOA6 |  |
|  |  | NCOA7 |  |
|  |  | NCOR1 |  |
|  |  | COG1 |  |
|  |  | COG2 |  |
|  |  | COG3 |  |
|  |  | COG5 |  |
|  |  | COG6 |  |
|  |  | NDFIP1 |  |
|  |  | COL10A1 |  |
|  |  | COL11A1 |  |
|  |  | COL12A1 |  |
|  |  | COL13A1 |  |
|  |  | COL19A1 |  |
|  |  | COL20A1 |  |
|  |  | COL21A1 |  |
|  |  | COL24A1 |  |
|  |  | COL28A1 |  |
|  |  | COL4A1 |  |
|  |  | COL4A3 |  |
|  |  | COL4A3BP |  |
|  |  | COL4A4 |  |
|  |  | COL4A5 |  |
|  |  | COL4A6 |  |
|  |  | COL5A2 |  |
|  |  | COL5A3 |  |
|  |  | COL6A6 |  |
|  |  | COL9A2 |  |
|  |  | COLEC10 |  |
|  |  | COLEC12 |  |
|  |  | COLGALT1 |  |
|  |  | COMMD10 |  |
|  |  | COMMD2 |  |
|  |  | COMMD3-BMI1 |  |
|  |  | COMMD6 |  |
|  |  | COMMD9 |  |
|  |  | COMTD1 |  |
|  |  | AC015987.2 |  |
|  |  | COPA |  |
|  |  | AC024257.1 |  |
|  |  | COPS7B |  |
|  |  | COQ4 |  |
|  |  | AC079612.1 |  |
|  |  | NFAT5 |  |
|  |  | CORO7 |  |
|  |  | CORO7-PAM16 |  |
|  |  | AC107021.1 |  |
|  |  | NFIL3 |  |
|  |  | NFKBIA |  |
|  |  | COTL1 |  |
|  |  | NGFRAP1 |  |
|  |  | AC132216.1 |  |
|  |  | NHLRC2 |  |
|  |  | NHLRC3 |  |
|  |  | COX10 |  |
|  |  | COX17 |  |
|  |  | COX18 |  |
|  |  | COX19 |  |
|  |  | COX7A2 |  |
|  |  | COX7A2L |  |
|  |  | COX7C |  |
|  |  | COX8C |  |
|  |  | CPA4 |  |
|  |  | CPA6 |  |
|  |  | NIPSNAP3A |  |
|  |  | NKD1 |  |
|  |  | NKIRAS1 |  |
|  |  | NKTR |  |
|  |  | NKX3-1 |  |
|  |  | NKX6-1 |  |
|  |  | NLE1 |  |
|  |  | NLGN1 |  |
|  |  | NLK |  |
|  |  | NLN |  |
|  |  | NLRP10 |  |
|  |  | NLRP2 |  |
|  |  | NMD3 |  |
|  |  | NME1 |  |
|  |  | NMNAT1 |  |
|  |  | NMRK1 |  |
|  |  | ZNF609 |  |
|  |  | ZNF611 |  |
|  |  | ZNF618 |  |
|  |  | ZNF620 |  |
|  |  | ZNF621 |  |
|  |  | ZNF622 |  |
|  |  | ZNF623 |  |
|  |  | ZNF625 |  |
|  |  | ZNF626 |  |
|  |  | ZNF627 |  |
|  |  | ZNF629 |  |
|  |  | ZNF638 |  |
|  |  | ZNF641 |  |
|  |  | ZNF644 |  |
|  |  | ZNF645 |  |
|  |  | MCL1 |  |
|  |  | ZNF654 |  |
|  |  | MCM5 |  |
|  |  | ZNF670 |  |
|  |  | ZNF671 |  |
|  |  | ZNF678 |  |
|  |  | ZNF699 |  |
|  |  | ZNF70 |  |
|  |  | ZNF701 |  |
|  |  | ZNF703 |  |
|  |  | ZNF704 |  |
|  |  | ZNF706 |  |
|  |  | ZNF708 |  |
|  |  | ZNF71 |  |
|  |  | ZNF716 |  |
|  |  | ZNF721 |  |
|  |  | ZNF726 |  |
|  |  | ZNF730 |  |
|  |  | ZNF736 |  |
|  |  | ZNF737 |  |
|  |  | ZNF74 |  |
|  |  | ZNF740 |  |
|  |  | ZNF746 |  |
|  |  | ZNF749 |  |
|  |  | ZNF75D |  |
|  |  | ZNF763 |  |
|  |  | ZNF766 |  |
|  |  | ADIPOQ |  |
|  |  | MEGF11 |  |
|  |  | MEGF9 |  |
|  |  | MEI4 |  |
|  |  | MEIS1 |  |
|  |  | MEPCE |  |
|  |  | MESDC1 |  |
|  |  | MESDC2 |  |
|  |  | MEST |  |
|  |  | MET |  |
|  |  | METAP1 |  |
|  |  | METTL14 |  |
|  |  | METTL21A |  |
|  |  | METTL23 |  |
|  |  | METTL24 |  |
|  |  | METTL2B |  |
|  |  | CHD7 |  |
|  |  | CHEK1 |  |
|  |  | CHERP |  |
|  |  | CHGA |  |
|  |  | CHIC1 |  |
|  |  | CHIC2 |  |
|  |  | MFI2 |  |
|  |  | QPCTL |  |
|  |  | MFSD2A |  |
|  |  | MFSD6 |  |
|  |  | MFSD8 |  |
|  |  | MGA |  |
|  |  | MGAT1 |  |
|  |  | MGAT2 |  |
|  |  | MGAT4A |  |
|  |  | MGAT4C |  |
|  |  | MGAT5 |  |
|  |  | MGEA5 |  |
|  |  | MGLL |  |
|  |  | MGP |  |
|  |  | MGRN1 |  |
|  |  | MIA3 |  |
|  |  | MIB1 |  |
|  |  | MICAL2 |  |
|  |  | MICAL3 |  |
|  |  | MICALCL |  |
|  |  | MICALL1 |  |
|  |  | MICU3 |  |
|  |  | MID1 |  |
|  |  | MIEF1 |  |
|  |  | MIER3 |  |
|  |  | MINA |  |
|  |  | MINOS1 |  |
|  |  | MIOS |  |
|  |  | MIPOL1 |  |
|  |  | MIXL1 |  |
|  |  | MKL2 |  |
|  |  | CINP |  |
|  |  | MKNK2 |  |
|  |  | MKRN2 |  |
|  |  | MKX |  |
|  |  | MLEC |  |
|  |  | MLF1IP |  |
|  |  | MLH3 |  |
|  |  | MLK4 |  |
|  |  | MLLT3 |  |
|  |  | MLLT4 |  |
|  |  | MLTK |  |
|  |  | MLXIP |  |
|  |  | MMAA |  |
|  |  | MMACHC |  |
|  |  | MMADHC |  |
|  |  | MMD |  |
|  |  | DUOX1 |  |
|  |  | GNPAT |  |
|  |  | DUSP18 |  |
|  |  | DUSP19 |  |
|  |  | DUSP28 |  |
|  |  | DUSP3 |  |
|  |  | DUSP5 |  |
|  |  | RAET1L |  |
|  |  | DUT |  |
|  |  | MNT |  |
|  |  | DYM |  |
|  |  | DYNAP |  |
|  |  | DYNC1I2 |  |
|  |  | MOB3B |  |
|  |  | MOCS2 |  |
|  |  | MOCS3 |  |
|  |  | MOK |  |
|  |  | MON1B |  |
|  |  | MON2 |  |
|  |  | MORC1 |  |
|  |  | MORC3 |  |
|  |  | MORF4L2 |  |
|  |  | MORN2 |  |
|  |  | E2F6 |  |
|  |  | MOSPD1 |  |
|  |  | MOSPD2 |  |
|  |  | MOXD1 |  |
|  |  | MPC2 |  |
|  |  | MPDZ |  |
|  |  | MPHOSPH6 |  |
|  |  | MPLKIP |  |
|  |  | ECEL1 |  |
|  |  | ECHDC1 |  |
|  |  | ECHDC2 |  |
|  |  | ECT2 |  |
|  |  | EDA2R |  |
|  |  | MPZL3 |  |
|  |  | MROH8 |  |
|  |  | MRPL2 |  |
|  |  | MRPL44 |  |
|  |  | MRPS16 |  |
|  |  | MSX2 |  |
|  |  | MT1E |  |
|  |  | MTF2 |  |
|  |  | MTHFS |  |
|  |  | MTM1 |  |
|  |  | MTSS1 |  |
|  |  | MTUS2 |  |
|  |  | MUC15 |  |
|  |  | MUC3A |  |
|  |  | MUS81 |  |
|  |  | MYBPC1 |  |
|  |  | MYBPC2 |  |
|  |  | MYF6 |  |
|  |  | MYH7B |  |
|  |  | MYH9 |  |
|  |  | MYL12A |  |
|  |  | MYLK |  |
|  |  | MYOD1 |  |
|  |  | MYOT |  |
|  |  | N4BP2L1 |  |
|  |  | NAA20 |  |
|  |  | PDCD6 |  |
|  |  | PDCD6IP |  |
|  |  | PDCL3 |  |
|  |  | PDE10A |  |
|  |  | PDE12 |  |
|  |  | PDE3B |  |
|  |  | PDE4A |  |
|  |  | PDE4D |  |
|  |  | PDE4DIP |  |
|  |  | PDE6C |  |
|  |  | PDE7A |  |
|  |  | PDE8B |  |
|  |  | PDE9A |  |
|  |  | PDGFC |  |
|  |  | PDHB |  |
|  |  | PDIA6 |  |
|  |  | PDK1 |  |
|  |  | PDK3 |  |
|  |  | NAPB |  |
|  |  | NARF |  |
|  |  | NARG2 |  |
|  |  | NBEA |  |
|  |  | NBR1 |  |
|  |  | NCAM2 |  |
|  |  | NCAN |  |
|  |  | NCAPD3 |  |
|  |  | NCAPG |  |
|  |  | NCAPG2 |  |
|  |  | NCAPH |  |
|  |  | NCEH1 |  |
|  |  | NCKAP1 |  |
|  |  | NCKIPSD |  |
|  |  | NCOA3 |  |
|  |  | NCOA4 |  |
|  |  | NCOA5 |  |
|  |  | NDEL1 |  |
|  |  | NDFIP2 |  |
|  |  | NDRG1 |  |
|  |  | CX3CR1 |  |
|  |  | CXCL10 |  |
|  |  | NDUFAF7 |  |
|  |  | NDUFB8 |  |
|  |  | NDUFC1 |  |
|  |  | NEBL |  |
|  |  | NEDD4L |  |
|  |  | NEK1 |  |
|  |  | NEK10 |  |
|  |  | NELL1 |  |
|  |  | NEUROD6 |  |
|  |  | NF2 |  |
|  |  | NFATC3 |  |
|  |  | NFATC4 |  |
|  |  | NFE2L1 |  |
|  |  | NFE2L3 |  |
|  |  | NFIA |  |
|  |  | C1orf147 |  |
|  |  | C1orf168 |  |
|  |  | NFYA |  |
|  |  | NGB |  |
|  |  | NGLY1 |  |
|  |  | NHEJ1 |  |
|  |  | C1QC |  |
|  |  | DCBLD1 |  |
|  |  | C21orf119 |  |
|  |  | C22orf26 |  |
|  |  | C22orf42 |  |
|  |  | NKX2-8 |  |
|  |  | NLGN3 |  |
|  |  | NLRP9 |  |
|  |  | NME7 |  |
|  |  | C3orf17 |  |
|  |  | NOL7 |  |
|  |  | NOM1 |  |
|  |  | NOV |  |
|  |  | NOVA2 |  |
|  |  | NPC1L1 |  |
|  |  | NPR3 |  |
|  |  | NPTN |  |
|  |  | NR0B1 |  |
|  |  | NR2C1 |  |
|  |  | NR2E1 |  |
|  |  | NR3C1 |  |
|  |  | NRBP2 |  |
|  |  | NRG2 |  |
|  |  | NRG3 |  |
|  |  | NRIP1 |  |
|  |  | NRIP2 |  |
|  |  | NSG1 |  |
|  |  | NSG2 |  |
|  |  | NSL1 |  |
|  |  | NT5C1B |  |
|  |  | NT5C1B-RDH14 |  |
|  |  | NT5M |  |
|  |  | NTF3 |  |
|  |  | NTN1 |  |
|  |  | NTSR1 |  |
|  |  | NUDT5 |  |
|  |  | NUDT7 |  |
|  |  | NUFIP1 |  |
|  |  | NUP62 |  |
|  |  | POU6F2 |  |
|  |  | PPAP2A |  |
|  |  | PPAP2B |  |
|  |  | DNASE2B |  |
|  |  | PPARGC1B |  |
|  |  | PPAT |  |
|  |  | PPCS |  |
|  |  | PPEF2 |  |
|  |  | PPFIA1 |  |
|  |  | PPFIA2 |  |
|  |  | PPFIA4 |  |
|  |  | PPFIBP1 |  |
|  |  | PPFIBP2 |  |
|  |  | PPIC |  |
|  |  | PPIG |  |
|  |  | DOK4 |  |
|  |  | PPIL4 |  |
|  |  | PPIL6 |  |
|  |  | PPIP5K2 |  |
|  |  | PPM1A |  |
|  |  | PPM1E |  |
|  |  | PPM1H |  |
|  |  | PPM1K |  |
|  |  | PPM1L |  |
|  |  | PPP1CB |  |
|  |  | PPP1CC |  |
|  |  | PPP1R12A |  |
|  |  | PPP1R12B |  |
|  |  | PPP1R14C |  |
|  |  | DPY19L4 |  |
|  |  | CAGE1 |  |
|  |  | PPP1R18 |  |
|  |  | PPP1R1C |  |
|  |  | PPP1R2 |  |
|  |  | PPP1R3C |  |
|  |  | PPP1R3D |  |
|  |  | PPP1R3E |  |
|  |  | PPP1R3F |  |
|  |  | PPP1R8 |  |
|  |  | PPP1R9A |  |
|  |  | PPP2CA |  |
|  |  | PPP2R1B |  |
|  |  | PPP2R2A |  |
|  |  | PPP2R2D |  |
|  |  | PPP2R3A |  |
|  |  | PPP2R5A |  |
|  |  | PPP2R5B |  |
|  |  | DTWD1 |  |
|  |  | PPP3CB |  |
|  |  | PPP3CC |  |
|  |  | PPP3R1 |  |
|  |  | PPP4R1 |  |
|  |  | PPP4R1L |  |
|  |  | PPP4R2 |  |
|  |  | PPP4R4 |  |
|  |  | PPP6C |  |
|  |  | PPP6R3 |  |
|  |  | PPTC7 |  |
|  |  | PQLC3 |  |
|  |  | PRCP |  |
|  |  | PRDM1 |  |
|  |  | PRDM16 |  |
|  |  | PRDM7 |  |
|  |  | PRDX1 |  |
|  |  | PRELID2 |  |
|  |  | PRH2 |  |
|  |  | PRICKLE1 |  |
|  |  | PRICKLE2 |  |
|  |  | PRIMA1 |  |
|  |  | PRKAA1 |  |
|  |  | PRKAA2 |  |
|  |  | PRKAB1 |  |
|  |  | PRKAB2 |  |
|  |  | PRKACA |  |
|  |  | PRKACB |  |
|  |  | PRKAR1A |  |
|  |  | PRKAR2A |  |
|  |  | PRKCA |  |
|  |  | PRKCB |  |
|  |  | PRKCE |  |
|  |  | PRKCI |  |
|  |  | PRKD1 |  |
|  |  | PRKD2 |  |
|  |  | PRKD3 |  |
|  |  | PRKG1 |  |
|  |  | PRKG2 |  |
|  |  | PRKX |  |
|  |  | PRLR |  |
|  |  | PRMT3 |  |
|  |  | PRMT6 |  |
|  |  | PRND |  |
|  |  | PROCA1 |  |
|  |  | PROK2 |  |
|  |  | PROKR2 |  |
|  |  | PROS1 |  |
|  |  | PROSER1 |  |
|  |  | PROSER2 |  |
|  |  | PROX1 |  |
|  |  | PROX2 |  |
|  |  | PRPF18 |  |
|  |  | PRPF38B |  |
|  |  | PRPF40A |  |
|  |  | PRPF4B |  |
|  |  | PRPH2 |  |
|  |  | PRPSAP1 |  |
|  |  | PRPSAP2 |  |
|  |  | PRRC1 |  |
|  |  | PRRC2C |  |
|  |  | PRRG4 |  |
|  |  | PRRX1 |  |
|  |  | PRSS12 |  |
|  |  | PRSS16 |  |
|  |  | PRUNE |  |
|  |  | PRUNE2 |  |
|  |  | PSAT1 |  |
|  |  | PSD2 |  |
|  |  | PSD3 |  |
|  |  | PSD4 |  |
|  |  | PSEN1 |  |
|  |  | PSENEN |  |
|  |  | PSMA5 |  |
|  |  | PSMA8 |  |
|  |  | PSMC2 |  |
|  |  | PSMD3 |  |
|  |  | PSMD9 |  |
|  |  | PSMF1 |  |
|  |  | PSMG4 |  |
|  |  | PSORS1C2 |  |
|  |  | PSPC1 |  |
|  |  | PSTK |  |
|  |  | PCDHA12 |  |
|  |  | PCDHA13 |  |
|  |  | PTCD3 |  |
|  |  | PCDHA3 |  |
|  |  | PCDHA4 |  |
|  |  | PTCHD3 |  |
|  |  | TAF1B |  |
|  |  | PCDHA7 |  |
|  |  | PTER |  |
|  |  | PCDHA9 |  |
|  |  | PCDHAC1 |  |
|  |  | PCDHAC2 |  |
|  |  | PTGES3L-AARSD1 | |
|  |  | PTH2R |  |
|  |  | PTK2 |  |
|  |  | PTPN20B |  |
|  |  | PTPN21 |  |
|  |  | PTPN22 |  |
|  |  | PTPN4 |  |
|  |  | PTPN5 |  |
|  |  | PTPRB |  |
|  |  | PTPRN2 |  |
|  |  | PTPRQ |  |
|  |  | PTPRS |  |
|  |  | PTPRT |  |
|  |  | PTRH1 |  |
|  |  | EPB41L4B |  |
|  |  | EPHA3 |  |
|  |  | TCEAL4 |  |
|  |  | TCEAL6 |  |
|  |  | EPHX2 |  |
|  |  | EPHX4 |  |
|  |  | TCERG1 |  |
|  |  | TCF4 |  |
|  |  | TCFL5 |  |
|  |  | TCOF1 |  |
|  |  | TDO2 |  |
|  |  | R3HDM1 |  |
|  |  | RAB11A |  |
|  |  | RAB11FIP1 |  |
|  |  | RAB11FIP4 |  |
|  |  | RAB11FIP5 |  |
|  |  | RAB13 |  |
|  |  | RAB14 |  |
|  |  | GJB7 |  |
|  |  | ERRFI1 |  |
|  |  | ESM1 |  |
|  |  | GLDC |  |
|  |  | GLG1 |  |
|  |  | GLI2 |  |
|  |  | GLI3 |  |
|  |  | GLIPR1L2 |  |
|  |  | GLIS3 |  |
|  |  | GLMN |  |
|  |  | ETV6 |  |
|  |  | GLP1R |  |
|  |  | GLRA2 |  |
|  |  | GLRA3 |  |
|  |  | GLRX3 |  |
|  |  | GLS |  |
|  |  | RABAC1 |  |
|  |  | RABEP1 |  |
|  |  | RABGAP1L |  |
|  |  | RABGEF1 |  |
|  |  | RABGGTB |  |
|  |  | RABIF |  |
|  |  | RAC1 |  |
|  |  | RAD1 |  |
|  |  | RAD18 |  |
|  |  | RAD21 |  |
|  |  | RAD51 |  |
|  |  | RAD51B |  |
|  |  | RAD54B |  |
|  |  | RAD9B |  |
|  |  | RAE1 |  |
|  |  | RAG1 |  |
|  |  | PHLPP1 |  |
|  |  | RALGAPB |  |
|  |  | RALGPS1 |  |
|  |  | RALGPS2 |  |
|  |  | RALY |  |
|  |  | RAN |  |
|  |  | RANBP1 |  |
|  |  | RANBP6 |  |
|  |  | RANBP9 |  |
|  |  | RAP1A |  |
|  |  | RAP1B |  |
|  |  | RAP1GAP2 |  |
|  |  | RAP1GDS1 |  |
|  |  | RAP2A |  |
|  |  | RAP2B |  |
|  |  | RAP2C |  |
|  |  | RAPGEF1 |  |
|  |  | RAPGEF2 |  |
|  |  | RAPGEF4 |  |
|  |  | RAPGEF5 |  |
|  |  | RAPH1 |  |
|  |  | RARB |  |
|  |  | RASA1 |  |
|  |  | RASA2 |  |
|  |  | RASA3 |  |
|  |  | RASAL2 |  |
|  |  | RASD1 |  |
|  |  | RASEF |  |
|  |  | RASGRF1 |  |
|  |  | RASGRF2 |  |
|  |  | RASGRP1 |  |
|  |  | RASGRP3 |  |
|  |  | RASL10A |  |
|  |  | RASL10B |  |
|  |  | RASL11A |  |
|  |  | RASSF3 |  |
|  |  | RASSF6 |  |
|  |  | RASSF9 |  |
|  |  | RAVER2 |  |
|  |  | RB1 |  |
|  |  | RB1CC1 |  |
|  |  | RBBP5 |  |
|  |  | RBBP6 |  |
|  |  | RBBP7 |  |
|  |  | RBBP8 |  |
|  |  | RBFOX1 |  |
|  |  | RBFOX2 |  |
|  |  | RBL2 |  |
|  |  | RBM12 |  |
|  |  | RBM12B |  |
|  |  | RBM18 |  |
|  |  | RBM20 |  |
|  |  | RBM22 |  |
|  |  | RBM23 |  |
|  |  | RBM24 |  |
|  |  | RBM25 |  |
|  |  | RBM27 |  |
|  |  | RBM28 |  |
|  |  | RBM3 |  |
|  |  | RBM33 |  |
|  |  | RBM39 |  |
|  |  | RBM41 |  |
|  |  | RBM43 |  |
|  |  | RBM45 |  |
|  |  | RBM46 |  |
|  |  | RBM47 |  |
|  |  | RBM4B |  |
|  |  | RBM7 |  |
|  |  | RBM8A |  |
|  |  | RBMS2 |  |
|  |  | RBMX2 |  |
|  |  | RBMXL1 |  |
|  |  | RBMXL2 |  |
|  |  | RBP2 |  |
|  |  | RBPJ |  |
|  |  | RBX1 |  |
|  |  | RC3H1 |  |
|  |  | PLEKHN1 |  |
|  |  | RCAN3 |  |
|  |  | RCC1 |  |
|  |  | RCCD1 |  |
|  |  | RCHY1 |  |
|  |  | RCN1 |  |
|  |  | PLLP |  |
|  |  | RCOR1 |  |
|  |  | RCOR3 |  |
|  |  | RCSD1 |  |
|  |  | RDH10 |  |
|  |  | RDX |  |
|  |  | REEP3 |  |
|  |  | REL |  |
|  |  | REPIN1 |  |
|  |  | RERGL |  |
|  |  | REST |  |
|  |  | RET |  |
|  |  | RETSAT |  |
|  |  | REV1 |  |
|  |  | REV3L |  |
|  |  | RFC2 |  |
|  |  | RFC3 |  |
|  |  | RFESD |  |
|  |  | RFK |  |
|  |  | RFPL4B |  |
|  |  | RFTN2 |  |
|  |  | RFWD3 |  |
|  |  | RFX3 |  |
|  |  | RFX4 |  |
|  |  | RFX6 |  |
|  |  | RFX7 |  |
|  |  | RFXAP |  |
|  |  | RGL1 |  |
|  |  | RGMA |  |
|  |  | RGPD1 |  |
|  |  | POFUT1 |  |
|  |  | RGPD3 |  |
|  |  | RGPD4 |  |
|  |  | RGPD5 |  |
|  |  | RGPD6 |  |
|  |  | POLG |  |
|  |  | RGR |  |
|  |  | RGS1 |  |
|  |  | RGS13 |  |
|  |  | RGS14 |  |
|  |  | RGS17 |  |
|  |  | RGS18 |  |
|  |  | RGS19 |  |
|  |  | RGS2 |  |
|  |  | RGS20 |  |
|  |  | RGS5 |  |
|  |  | RGS7BP |  |
|  |  | RGS9BP |  |
|  |  | POLR3F |  |
|  |  | POLR3G |  |
|  |  | POLR3K |  |
|  |  | RHOBTB1 |  |
|  |  | RHOBTB3 |  |
|  |  | RHOQ |  |
|  |  | RHOT1 |  |
|  |  | RHOU |  |
|  |  | RIC8B |  |
|  |  | RICTOR |  |
|  |  | RIF1 |  |
|  |  | RILPL1 |  |
|  |  | RIMBP2 |  |
|  |  | RIMKLA |  |
|  |  | POU3F3 |  |
|  |  | POU4F2 |  |
|  |  | RIN2 |  |
|  |  | RIOK2 |  |
|  |  | RIOK3 |  |
|  |  | RIPK1 |  |
|  |  | RIPK4 |  |
|  |  | PPAPDC3 |  |
|  |  | RMI1 |  |
|  |  | RMND5A |  |
|  |  | RMND5B |  |
|  |  | RNASE1 |  |
|  |  | RNASE11 |  |
|  |  | RNASE13 |  |
|  |  | RNASEH1 |  |
|  |  | RNASEH2B |  |
|  |  | RNASEH2C |  |
|  |  | RNF11 |  |
|  |  | RNF111 |  |
|  |  | RNF114 |  |
|  |  | RNF115 |  |
|  |  | RNF125 |  |
|  |  | RNF13 |  |
|  |  | RNF139 |  |
|  |  | RNF141 |  |
|  |  | RNF144A |  |
|  |  | RNF144B |  |
|  |  | RNF146 |  |
|  |  | RNF149 |  |
|  |  | RNF150 |  |
|  |  | RNF152 |  |
|  |  | RNF157 |  |
|  |  | RNF168 |  |
|  |  | RNF169 |  |
|  |  | RNF170 |  |
|  |  | RNF182 |  |
|  |  | RNF19A |  |
|  |  | RNF2 |  |
|  |  | RNF20 |  |
|  |  | RNF213 |  |
|  |  | RNF214 |  |
|  |  | RNF217 |  |
|  |  | RNF219 |  |
|  |  | RNF223 |  |
|  |  | RNF224 |  |
|  |  | RNF24 |  |
|  |  | RNF38 |  |
|  |  | RNF4 |  |
|  |  | RNF41 |  |
|  |  | RNF43 |  |
|  |  | RNF44 |  |
|  |  | RNFT1 |  |
|  |  | RNGTT |  |
|  |  | RNLS |  |
|  |  | RNMT |  |
|  |  | ROBO1 |  |
|  |  | ROBO2 |  |
|  |  | ROBO4 |  |
|  |  | ROCK1 |  |
|  |  | ROCK2 |  |
|  |  | ROR2 |  |
|  |  | RORA |  |
|  |  | RORB |  |
|  |  | ROS1 |  |
|  |  | RP11-10A14.4 |  |
|  |  | RP11-111M22.2 |  |
|  |  | RP11-116D17.1 |  |
|  |  | RP11-122A3.2 |  |
|  |  | RP11-156E8.1 |  |
|  |  | RP11-17M16.1 |  |
|  |  | RP11-192H23.4 |  |
|  |  | RP11-210M15.2 |  |
|  |  | RP11-272B17.2 |  |
|  |  | RP11-315D16.2 |  |
|  |  | RP11-366L20.2 |  |
|  |  | RP11-382J12.1 |  |
|  |  | RP11-385D13.1 |  |
|  |  | RP11-451M19.3 |  |
|  |  | RP11-455G16.1 |  |
|  |  | RP11-57H12.6 |  |
|  |  | RP11-65D24.2 |  |
|  |  | PRKAG2 |  |
|  |  | RP11-758M4.1 |  |
|  |  | RP11-766F14.2 |  |
|  |  | RP11-770J1.4 |  |
|  |  | RP11-831H9.11 |  |
|  |  | RP11-93B14.6 |  |
|  |  | RP1-241P17.4 |  |
|  |  | RP13-996F3.5 |  |
|  |  | RP2 |  |
|  |  | RP4-559A3.7 |  |
|  |  | RP4-758J18.2 |  |
|  |  | RP5-850E9.3 |  |
|  |  | RPAP2 |  |
|  |  | PROCR |  |
|  |  | RPGRIP1L |  |
|  |  | RPL13 |  |
|  |  | RPL18A |  |
|  |  | RPL22 |  |
|  |  | RPL22L1 |  |
|  |  | RPL24 |  |
|  |  | RPL34 |  |
|  |  | RPL36A-HNRNPH2 | |
|  |  | RPL37 |  |
|  |  | RPL37A |  |
|  |  | RPL39 |  |
|  |  | RPL5 |  |
|  |  | RPL7A |  |
|  |  | RPL7L1 |  |
|  |  | RPP14 |  |
|  |  | RPP40 |  |
|  |  | RPRD1A |  |
|  |  | RPS15A |  |
|  |  | RPS24 |  |
|  |  | RPS27A |  |
|  |  | RPS4X |  |
|  |  | RPS6 |  |
|  |  | RPS6KA5 |  |
|  |  | RPS6KA6 |  |
|  |  | RPS6KB1 |  |
|  |  | RPS6KC1 |  |
|  |  | RPTOR |  |
|  |  | RQCD1 |  |
|  |  | RRAGC |  |
|  |  | RRAGD |  |
|  |  | RREB1 |  |
|  |  | RRH |  |
|  |  | RRM1 |  |
|  |  | RRM2 |  |
|  |  | RRN3 |  |
|  |  | RRP36 |  |
|  |  | RRP8 |  |
|  |  | RSBN1 |  |
|  |  | RSBN1L |  |
|  |  | RSF1 |  |
|  |  | RSL1D1 |  |
|  |  | RSPH10B |  |
|  |  | STRN3 |  |
|  |  | RSPO2 |  |
|  |  | RSPO3 |  |
|  |  | RSRC2 |  |
|  |  | RTCA |  |
|  |  | RTCB |  |
|  |  | RTKN2 |  |
|  |  | RTN4 |  |
|  |  | RUFY2 |  |
|  |  | RUNX1 |  |
|  |  | RUNX1T1 |  |
|  |  | RUNX3 |  |
|  |  | RWDD1 |  |
|  |  | RWDD2A |  |
|  |  | RXRB |  |
|  |  | RYBP |  |
|  |  | RYK |  |
|  |  | RYR3 |  |
|  |  | S100A7A |  |
|  |  | S1PR3 |  |
|  |  | SACM1L |  |
|  |  | SACS |  |
|  |  | SAFB2 |  |
|  |  | SALL1 |  |
|  |  | GALNT18 |  |
|  |  | SAMD14 |  |
|  |  | SAMD8 |  |
|  |  | SAP18 |  |
|  |  | SAP30 |  |
|  |  | SAP30L |  |
|  |  | SAR1A |  |
|  |  | SAR1B |  |
|  |  | SARM1 |  |
|  |  | SART3 |  |
|  |  | SASH1 |  |
|  |  | SASS6 |  |
|  |  | SATB1 |  |
|  |  | SATB2 |  |
|  |  | SAV1 |  |
|  |  | SBF2 |  |
|  |  | SBNO1 |  |
|  |  | SBSPON |  |
|  |  | SC5D |  |
|  |  | SCAF4 |  |
|  |  | SCAF8 |  |
|  |  | SCAI |  |
|  |  | SCAMP1 |  |
|  |  | SCAND3 |  |
|  |  | SCARB1 |  |
|  |  | SCARB2 |  |
|  |  | SCG2 |  |
|  |  | SCIMP |  |
|  |  | SCIN |  |
|  |  | SCML1 |  |
|  |  | SCML2 |  |
|  |  | SCML4 |  |
|  |  | SCN1A |  |
|  |  | PURA |  |
|  |  | GDAP2 |  |
|  |  | PURG |  |
|  |  | PUS10 |  |
|  |  | PUS7 |  |
|  |  | PVRL1 |  |
|  |  | CHCHD3 |  |
|  |  | CHD1 |  |
|  |  | CHD2 |  |
|  |  | CHD5 |  |
|  |  | CHD6 |  |
|  |  | SZRD1 |  |
|  |  | QPCT |  |
|  |  | QSOX2 |  |
|  |  | CHMP2B |  |
|  |  | CHMP3 |  |
|  |  | CHMP5 |  |
|  |  | CHN1 |  |
|  |  | CHN2 |  |
|  |  | CHODL |  |
|  |  | RAB12 |  |
|  |  | RAB1A |  |
|  |  | CHRNB1 |  |
|  |  | CHRNB2 |  |
|  |  | CHST1 |  |
|  |  | RAB27A |  |
|  |  | RAB27B |  |
|  |  | CHST2 |  |
|  |  | CHST3 |  |
|  |  | CHST9 |  |
|  |  | CHTOP |  |
|  |  | CHUK |  |
|  |  | CIAO1 |  |
|  |  | CIAPIN1 |  |
|  |  | CIR1 |  |
|  |  | CIRBP |  |
|  |  | CIRH1A |  |
|  |  | CISD1 |  |
|  |  | CISD2 |  |
|  |  | DSG2 |  |
|  |  | DSG3 |  |
|  |  | DSTN |  |
|  |  | DSTYK |  |
|  |  | DTL |  |
|  |  | DTNA |  |
|  |  | DTWD2 |  |
|  |  | DTX3L |  |
|  |  | UGGT1 |  |
|  |  | CLCN6 |  |
|  |  | CLCN7 |  |
|  |  | GOLGA5 |  |
|  |  | UNC45A |  |
|  |  | UNC5C |  |
|  |  | DYRK1B |  |
|  |  | DYRK2 |  |
|  |  | CLNK |  |
|  |  | CLOCK |  |
|  |  | CLPB |  |
|  |  | CLSTN1 |  |
|  |  | UQCRB |  |
|  |  | EAF1 |  |
|  |  | EAPP |  |
|  |  | EBAG9 |  |
|  |  | EBF1 |  |
|  |  | EBF2 |  |
|  |  | EBF3 |  |
|  |  | EBF4 |  |
|  |  | EBPL |  |
|  |  | ECE1 |  |
|  |  | USP27X |  |
|  |  | USP3 |  |
|  |  | USP30 |  |
|  |  | USP31 |  |
|  |  | USP32 |  |
|  |  | EDEM3 |  |
|  |  | EDN1 |  |
|  |  | EDN3 |  |
|  |  | EDNRB |  |
|  |  | EEA1 |  |
|  |  | EED |  |
|  |  | EEF1A1 |  |
|  |  | EEF2K |  |
|  |  | EFCAB1 |  |
|  |  | EFCAB11 |  |
|  |  | EFCAB14 |  |
|  |  | RASSF7 |  |
|  |  | EFHD1 |  |
|  |  | EFNA2 |  |
|  |  | EFNA5 |  |
|  |  | EFNB1 |  |
|  |  | UTP23 |  |
|  |  | CNTN1 |  |
|  |  | EFR3A |  |
|  |  | EFR3B |  |
|  |  | EGFR |  |
|  |  | EGR1 |  |
|  |  | VASH2 |  |
|  |  | BARD1 |  |
|  |  | RBM48 |  |
|  |  | RBM5 |  |
|  |  | BCKDHB |  |
|  |  | BCL2L15 |  |
|  |  | BCL7A |  |
|  |  | BCORL1 |  |
|  |  | BCR |  |
|  |  | BEND6 |  |
|  |  | BEST1 |  |
|  |  | BGN |  |
|  |  | BHMT |  |
|  |  | BIRC3 |  |
|  |  | BLOC1S2 |  |
|  |  | WDR13 |  |
|  |  | BMI1 |  |
|  |  | BMP3 |  |
|  |  | BMP8A |  |
|  |  | BMP8B |  |
|  |  | WDR7 |  |
|  |  | WDR70 |  |
|  |  | TMED8 |  |
|  |  | BNC1 |  |
|  |  | BOLL |  |
|  |  | BRD7 |  |
|  |  | BRIX1 |  |
|  |  | BTBD11 |  |
|  |  | BTF3 |  |
|  |  | BTG3 |  |
|  |  | BTNL9 |  |
|  |  | BUB1 |  |
|  |  | C10orf105 |  |
|  |  | XPNPEP3 |  |
|  |  | C10orf126 |  |
|  |  | C10orf131 |  |
|  |  | C10orf25 |  |
|  |  | C10orf88 |  |
|  |  | C11orf48 |  |
|  |  | CWC27 |  |
|  |  | YIPF5 |  |
|  |  | YKT6 |  |
|  |  | YLPM1 |  |
|  |  | CXCL14 |  |
|  |  | CXorf38 |  |
|  |  | CXorf56 |  |
|  |  | CYB561D1 |  |
|  |  | ZAR1L |  |
|  |  | ZBED6 |  |
|  |  | ZBED6CL |  |
|  |  | CYFIP2 |  |
|  |  | CYGB |  |
|  |  | CYP1A1 |  |
|  |  | CYP1B1 |  |
|  |  | CYP2B6 |  |
|  |  | CYP2C18 |  |
|  |  | CYP3A43 |  |
|  |  | CYP7B1 |  |
|  |  | C19orf25 |  |
|  |  | C1orf110 |  |
|  |  | C1orf116 |  |
|  |  | C1orf123 |  |
|  |  | C1orf131 |  |
|  |  | ZC3H12D |  |
|  |  | ZC3H13 |  |
|  |  | C1orf21 |  |
|  |  | C1orf210 |  |
|  |  | C1orf226 |  |
|  |  | C1orf233 |  |
|  |  | ZCCHC2 |  |
|  |  | TNRC6A |  |
|  |  | ZCCHC6 |  |
|  |  | ZCCHC9 |  |
|  |  | ZDBF2 |  |
|  |  | ZDHHC11 |  |
|  |  | ZDHHC14 |  |
|  |  | ZDHHC15 |  |
|  |  | ZDHHC17 |  |
|  |  | ZDHHC2 |  |
|  |  | ZDHHC20 |  |
|  |  | ZDHHC21 |  |
|  |  | ZDHHC23 |  |
|  |  | DCUN1D2 |  |
|  |  | C3AR1 |  |
|  |  | ZFHX4 |  |
|  |  | C3orf55 |  |
|  |  | C3orf62 |  |
|  |  | C3orf65 |  |
|  |  | C3orf67 |  |
|  |  | C3orf70 |  |
|  |  | C4orf19 |  |
|  |  | TRA2A |  |
|  |  | TRA2B |  |
|  |  | HSPE1 |  |
|  |  | C5orf15 |  |
|  |  | C5orf20 |  |
|  |  | C5orf46 |  |
|  |  | C5orf64 |  |
|  |  | C7orf49 |  |
|  |  | C7orf73 |  |
|  |  | C8orf46 |  |
|  |  | C9orf114 |  |
|  |  | C9orf64 |  |
|  |  | C9orf66 |  |
|  |  | C9orf91 |  |
|  |  | CA14 |  |
|  |  | CA3 |  |
|  |  | CAB39 |  |
|  |  | CAB39L |  |
|  |  | DLAT |  |
|  |  | DLGAP2 |  |
|  |  | DLK1 |  |
|  |  | DLX1 |  |
|  |  | CABP4 |  |
|  |  | CABP7 |  |
|  |  | SPG11 |  |
|  |  | DMKN |  |
|  |  | DMRT3 |  |
|  |  | DMTF1 |  |
|  |  | DNAJA2 |  |
|  |  | DNAJC11 |  |
|  |  | CACNG8 |  |
|  |  | SREK1IP1 |  |
|  |  | LRRC34 |  |
|  |  | CIC |  |
|  |  | LRRC4 |  |
|  |  | SDC4 |  |
|  |  | DNM2 |  |
|  |  | CAD |  |
|  |  | DNMT3B |  |
|  |  | DNPH1 |  |
|  |  | DOK2 |  |
|  |  | LRRTM2 |  |
|  |  | DONSON |  |
|  |  | SSBP2 |  |
|  |  | DPM1 |  |
|  |  | CADPS |  |
|  |  | LUC7L3 |  |
|  |  | LURAP1L |  |
|  |  | LUZP1 |  |
|  |  | LUZP2 |  |
|  |  | LY75 |  |
|  |  | DRAXIN |  |
|  |  | DRD1 |  |
|  |  | LYPD6 |  |
|  |  | DSN1 |  |
|  |  | DSP |  |
|  |  | DTD1 |  |
|  |  | MAFA |  |
|  |  | SERPINB5 |  |
|  |  | DUSP22 |  |
|  |  | DYNC1H1 |  |
|  |  | DYNC1LI1 |  |
|  |  | E2F7 |  |
|  |  | EBI3 |  |
|  |  | ECD |  |
|  |  | EDEM1 |  |
|  |  | EDNRA |  |
|  |  | EFCAB2 |  |
|  |  | EFHC2 |  |
|  |  | CARD8 |  |
|  |  | EHBP1 |  |
|  |  | EHD3 |  |
|  |  | EI24 |  |
|  |  | EIF2S2 |  |
|  |  | MBTPS2 |  |
|  |  | EIF4G3 |  |
|  |  | ELF1 |  |
|  |  | CASC10 |  |
|  |  | CASP2 |  |
|  |  | MDM2 |  |
|  |  | TAF1D |  |
|  |  | CAST |  |
|  |  | TAF5 |  |
|  |  | TAGAP |  |
|  |  | EMC3 |  |
|  |  | EMC6 |  |
|  |  | EML3 |  |
|  |  | EML4 |  |
|  |  | TAS2R4 |  |
|  |  | TAS2R5 |  |
|  |  | EMX1 |  |
|  |  | TAX1BP1 |  |
|  |  | TBC1D12 |  |
|  |  | TBC1D30 |  |
|  |  | TBC1D4 |  |
|  |  | PTPRC |  |
|  |  | PTPRE |  |
|  |  | PTPRG |  |
|  |  | TBCK |  |
|  |  | TBL1XR1 |  |
|  |  | TBL3 |  |
|  |  | TBP |  |
|  |  | PTPRZ1 |  |
|  |  | TBPL2 |  |
|  |  | PTRH2 |  |
|  |  | PTS |  |
|  |  | TBX15 |  |
|  |  | PUF60 |  |
|  |  | PUS7L |  |
|  |  | PVR |  |
|  |  | PWWP2A |  |
|  |  | PXDNL |  |
|  |  | PYGO2 |  |
|  |  | QKI |  |
|  |  | R3HCC1 |  |
|  |  | TDRD1 |  |
|  |  | TDRD15 |  |
|  |  | TDRP |  |
|  |  | RAB11FIP2 |  |
|  |  | TEAD3 |  |
|  |  | TECRL |  |
|  |  | TECTA |  |
|  |  | TECTB |  |
|  |  | RAB15 |  |
|  |  | RAB18 |  |
|  |  | RAB21 |  |
|  |  | RAB28 |  |
|  |  | RAB2B |  |
|  |  | RAB30 |  |
|  |  | RAB31 |  |
|  |  | RAB32 |  |
|  |  | RAB35 |  |
|  |  | RAB36 |  |
|  |  | RAB39A |  |
|  |  | RAB39B |  |
|  |  | RAB3C |  |
|  |  | RAB3GAP1 |  |
|  |  | RAB3GAP2 |  |
|  |  | RAB3IP |  |
|  |  | RAB4A |  |
|  |  | RAB5A |  |
|  |  | RAB5B |  |
|  |  | RAB6A |  |
|  |  | RAB7A |  |
|  |  | RAB8B |  |
|  |  | RAB9A |  |
|  |  | RAB9B |  |
|  |  | EXOC6 |  |
|  |  | EXT1 |  |
|  |  | EXTL1 |  |
|  |  | EYA3 |  |
|  |  | EZR |  |
|  |  | FAF1 |  |
|  |  | FAIM |  |
|  |  | FAM104A |  |
|  |  | FAM104B |  |
|  |  | FAM107A |  |
|  |  | FAM120B |  |
|  |  | FAM131B |  |
|  |  | FAM134A |  |
|  |  | FAM134C |  |
|  |  | FAM135A |  |
|  |  | FAM135B |  |
|  |  | FAM167A |  |
|  |  | FAM168B |  |
|  |  | FAM171B |  |
|  |  | FAM174B |  |
|  |  | TLL2 |  |
|  |  | TLR2 |  |
|  |  | FAM184A |  |
|  |  | SMAD6 |  |
|  |  | CCBL1 |  |
|  |  | TMC7 |  |
|  |  | FAM208A |  |
|  |  | FAM212B |  |
|  |  | FAM216B |  |
|  |  | FAM217A |  |
|  |  | FAM219A |  |
|  |  | TMEM104 |  |
|  |  | TMEM117 |  |
|  |  | FAM43B |  |
|  |  | FAM53B |  |
|  |  | TMEM135 |  |
|  |  | SMYD2 |  |
|  |  | FAM64A |  |
|  |  | CCDC174 |  |
|  |  | CCDC177 |  |
|  |  | FAM89A |  |
|  |  | TMEM179 |  |
|  |  | TMEM179B |  |
|  |  | FAM9B |  |
|  |  | CCDC43 |  |
|  |  | SNX24 |  |
|  |  | TMEM19 |  |
|  |  | FARP1 |  |
|  |  | CCDC82 |  |
|  |  | CCDC9 |  |
|  |  | CCDC90B |  |
|  |  | CCDC96 |  |
|  |  | FBLN1 |  |
|  |  | FBLN5 |  |
|  |  | FBN3 |  |
|  |  | FBRSL1 |  |
|  |  | SOS1 |  |
|  |  | FBXL2 |  |
|  |  | FBXL5 |  |
|  |  | FBXO25 |  |
|  |  | FBXO31 |  |
|  |  | FBXO33 |  |
|  |  | FBXO34 |  |
|  |  | FBXO9 |  |
|  |  | FCHO2 |  |
|  |  | FCHSD1 |  |
|  |  | FCRL2 |  |
|  |  | FDX1 |  |
|  |  | CCL4 |  |
|  |  | CCL4L1 |  |
|  |  | CCL4L2 |  |
|  |  | FGD5 |  |
|  |  | FGF11 |  |
|  |  | FGF13 |  |
|  |  | FGF20 |  |
|  |  | CCNA2 |  |
|  |  | CCNF |  |
|  |  | FIS1 |  |
|  |  | CCNJL |  |
|  |  | FKBP1B |  |
|  |  | FLJ20306 |  |
|  |  | TOP2A |  |
|  |  | TOP2B |  |
|  |  | SRPR |  |
|  |  | SRPRB |  |
|  |  | SRPX2 |  |
|  |  | FNDC1 |  |
|  |  | SRSF10 |  |
|  |  | SRSF11 |  |
|  |  | FOCAD |  |
|  |  | SRSF6 |  |
|  |  | SRSF7 |  |
|  |  | SSB |  |
|  |  | SSFA2 |  |
|  |  | SSH2 |  |
|  |  | SSNA1 |  |
|  |  | CCR4 |  |
|  |  | CCR9 |  |
|  |  | FOXN4 |  |
|  |  | SSU72 |  |
|  |  | FOXO3 |  |
|  |  | ST3GAL6 |  |
|  |  | ST6GALNAC3 |  |
|  |  | ST6GALNAC5 |  |
|  |  | ST6GALNAC6 |  |
|  |  | ST7L |  |
|  |  | ST8SIA2 |  |
|  |  | ST8SIA3 |  |
|  |  | ST8SIA4 |  |
|  |  | STAG1 |  |
|  |  | STAG2 |  |
|  |  | STAM |  |
|  |  | STAM2 |  |
|  |  | STAMBPL1 |  |
|  |  | STARD13 |  |
|  |  | STARD3NL |  |
|  |  | ZNF516 |  |
|  |  | FSTL1 |  |
|  |  | FSTL3 |  |
|  |  | FSTL4 |  |
|  |  | FTSJ3 |  |
|  |  | FUK |  |
|  |  | FUNDC1 |  |
|  |  | CD160 |  |
|  |  | CD1D |  |
|  |  | CD22 |  |
|  |  | CD247 |  |
|  |  | CD274 |  |
|  |  | CD36 |  |
|  |  | STRIP1 |  |
|  |  | STRN |  |
|  |  | ACTR5 |  |
|  |  | STT3A |  |
|  |  | STX11 |  |
|  |  | CD8A |  |
|  |  | CDADC1 |  |
|  |  | GABPB1 |  |
|  |  | ADAM18 |  |
|  |  | STXBP5 |  |
|  |  | STXBP5L |  |
|  |  | STXBP6 |  |
|  |  | STYK1 |  |
|  |  | STYX |  |
|  |  | SUB1 |  |
|  |  | GABRR2 |  |
|  |  | TSPYL4 |  |
|  |  | TSPYL5 |  |
|  |  | CDCP1 |  |
|  |  | GALNT10 |  |
|  |  | SUFU |  |
|  |  | ADAP1 |  |
|  |  | ADAR |  |
|  |  | MDFI |  |
|  |  | GALNT4 |  |
|  |  | GALNT5 |  |
|  |  | SUPT7L |  |
|  |  | GAREM |  |
|  |  | GARNL3 |  |
|  |  | GAS1 |  |
|  |  | GAS2L1 |  |
|  |  | GATAD2B |  |
|  |  | TUSC2 |  |
|  |  | TVP23A |  |
|  |  | CDK15 |  |
|  |  | SYTL4 |  |
|  |  | SYVN1 |  |
|  |  | PXMP4 |  |
|  |  | PYGL |  |
|  |  | SDR42E1 |  |
|  |  | CDKN2B |  |
|  |  | GIMAP6 |  |
|  |  | TAF1A |  |
|  |  | GJB2 |  |
|  |  | TAF4B |  |
|  |  | TAF5L |  |
|  |  | GLRB |  |
|  |  | TAPT1 |  |
|  |  | CDR1as |  |
|  |  | GLUD1 |  |
|  |  | GLUD2 |  |
|  |  | CLCN4 |  |
|  |  | TBK1 |  |
|  |  | CLDN11 |  |
|  |  | CLDN19 |  |
|  |  | CLDN6 |  |
|  |  | CLDND1 |  |
|  |  | CLEC16A |  |
|  |  | CLEC4D |  |
|  |  | CLEC7A |  |
|  |  | CLIC2 |  |
|  |  | CLIC5 |  |
|  |  | CLIP1 |  |
|  |  | CLIP4 |  |
|  |  | TCEB1 |  |
|  |  | CLK2 |  |
|  |  | UNC5CL |  |
|  |  | UNC5D |  |
|  |  | UNC79 |  |
|  |  | UNC80 |  |
|  |  | UNG |  |
|  |  | UNKL |  |
|  |  | UPF3A |  |
|  |  | UPRT |  |
|  |  | UQCC1 |  |
|  |  | SGMS1 |  |
|  |  | UQCRFS1 |  |
|  |  | TDG |  |
|  |  | URI1 |  |
|  |  | USH2A |  |
|  |  | USP1 |  |
|  |  | USP13 |  |
|  |  | USP14 |  |
|  |  | USP15 |  |
|  |  | USP22 |  |
|  |  | USP26 |  |
|  |  | CMTR2 |  |
|  |  | CNDP1 |  |
|  |  | TERF1 |  |
|  |  | USP34 |  |
|  |  | USP38 |  |
|  |  | USP42 |  |
|  |  | USP44 |  |
|  |  | USP45 |  |
|  |  | USP46 |  |
|  |  | USP47 |  |
|  |  | USP49 |  |
|  |  | USP51 |  |
|  |  | USP53 |  |
|  |  | USP54 |  |
|  |  | USP6 |  |
|  |  | USP6NL |  |
|  |  | USP7 |  |
|  |  | USP8 |  |
|  |  | USP9X |  |
|  |  | USP9Y |  |
|  |  | USPL1 |  |
|  |  | CNST |  |
|  |  | UTRN |  |
|  |  | UTS2B |  |
|  |  | UVRAG |  |
|  |  | VAMP3 |  |
|  |  | VAMP4 |  |
|  |  | VANGL1 |  |
|  |  | VAPA |  |
|  |  | VAPB |  |
|  |  | CNTNAP5 |  |
|  |  | VAV2 |  |
|  |  | VAV3 |  |
|  |  | VCAM1 |  |
|  |  | VCAN |  |
|  |  | VCL |  |
|  |  | VCPIP1 |  |
|  |  | VCPKMT |  |
|  |  | VDAC2 |  |
|  |  | VEGFB |  |
|  |  | VEGFC |  |
|  |  | VEZF1 |  |
|  |  | VEZT |  |
|  |  | VGLL2 |  |
|  |  | VGLL3 |  |
|  |  | VHL |  |
|  |  | VIL1 |  |
|  |  | THPO |  |
|  |  | THRB |  |
|  |  | THSD4 |  |
|  |  | THSD7A |  |
|  |  | THSD7B |  |
|  |  | COL8A1 |  |
|  |  | THUMPD3 |  |
|  |  | TIA1 |  |
|  |  | TIAL1 |  |
|  |  | TIAM1 |  |
|  |  | TIAM2 |  |
|  |  | TICAM2 |  |
|  |  | VPS35 |  |
|  |  | VPS36 |  |
|  |  | VPS37B |  |
|  |  | VPS41 |  |
|  |  | VPS4A |  |
|  |  | VPS4B |  |
|  |  | VPS53 |  |
|  |  | VPS9D1 |  |
|  |  | VRTN |  |
|  |  | VSIG10 |  |
|  |  | VSIG4 |  |
|  |  | VSNL1 |  |
|  |  | VSTM2B |  |
|  |  | VSTM4 |  |
|  |  | VTA1 |  |
|  |  | VTI1A |  |
|  |  | VTI1B |  |
|  |  | VWA3B |  |
|  |  | TLK2 |  |
|  |  | VWDE |  |
|  |  | WAC |  |
|  |  | WARS |  |
|  |  | WARS2 |  |
|  |  | WASL |  |
|  |  | WBP11 |  |
|  |  | WBP1L |  |
|  |  | WDFY1 |  |
|  |  | TM4SF18 |  |
|  |  | TM4SF20 |  |
|  |  | TM6SF1 |  |
|  |  | TM7SF3 |  |
|  |  | TM9SF2 |  |
|  |  | WDR3 |  |
|  |  | WDR33 |  |
|  |  | WDR35 |  |
|  |  | WDR36 |  |
|  |  | TMCO1 |  |
|  |  | WDR41 |  |
|  |  | WDR47 |  |
|  |  | WDR48 |  |
|  |  | WDR53 |  |
|  |  | WDR55 |  |
|  |  | CREB3L1 |  |
|  |  | WDR76 |  |
|  |  | WDR77 |  |
|  |  | WDR78 |  |
|  |  | WDR82 |  |
|  |  | WDR89 |  |
|  |  | WDR91 |  |
|  |  | TMEM115 |  |
|  |  | WEE1 |  |
|  |  | WEE2 |  |
|  |  | WHAMM |  |
|  |  | WHSC1L1 |  |
|  |  | WIF1 |  |
|  |  | WIPF1 |  |
|  |  | WIPI2 |  |
|  |  | WISP1 |  |
|  |  | WISP3 |  |
|  |  | WLS |  |
|  |  | WNK1 |  |
|  |  | WNK2 |  |
|  |  | TMEM161B |  |
|  |  | TMEM163 |  |
|  |  | WNT2 |  |
|  |  | WNT2B |  |
|  |  | WNT3A |  |
|  |  | WNT5A |  |
|  |  | WNT7A |  |
|  |  | WNT8B |  |
|  |  | WNT9A |  |
|  |  | WRN |  |
|  |  | WSB1 |  |
|  |  | WSB2 |  |
|  |  | CTBP1 |  |
|  |  | WWC2 |  |
|  |  | WWC3 |  |
|  |  | WWP2 |  |
|  |  | WWTR1 |  |
|  |  | XIAP |  |
|  |  | XIRP1 |  |
|  |  | XKR4 |  |
|  |  | XKR6 |  |
|  |  | XKR9 |  |
|  |  | XKRX |  |
|  |  | CTH |  |
|  |  | XPO4 |  |
|  |  | XPO5 |  |
|  |  | XPO7 |  |
|  |  | XPOT |  |
|  |  | XPR1 |  |
|  |  | XRCC3 |  |
|  |  | XRCC4 |  |
|  |  | XRCC5 |  |
|  |  | XRN1 |  |
|  |  | XRRA1 |  |
|  |  | XYLB |  |
|  |  | XYLT1 |  |
|  |  | YAE1D1 |  |
|  |  | YAF2 |  |
|  |  | YAP1 |  |
|  |  | YBX1 |  |
|  |  | YES1 |  |
|  |  | YIPF4 |  |
|  |  | YPEL1 |  |
|  |  | YPEL2 |  |
|  |  | YTHDC1 |  |
|  |  | YTHDF2 |  |
|  |  | YTHDF3 |  |
|  |  | YWHAB |  |
|  |  | YWHAE |  |
|  |  | YWHAG |  |
|  |  | YY1 |  |
|  |  | YY2 |  |
|  |  | ZADH2 |  |
|  |  | ZBED3 |  |
|  |  | ZBED4 |  |
|  |  | ZBTB10 |  |
|  |  | ZBTB11 |  |
|  |  | ZBTB16 |  |
|  |  | ZBTB18 |  |
|  |  | ZBTB20 |  |
|  |  | ZBTB21 |  |
|  |  | ZBTB25 |  |
|  |  | ZBTB3 |  |
|  |  | ZBTB33 |  |
|  |  | ZBTB34 |  |
|  |  | ZBTB37 |  |
|  |  | ZBTB38 |  |
|  |  | ZBTB41 |  |
|  |  | ZBTB44 |  |
|  |  | ZBTB45 |  |
|  |  | ZBTB47 |  |
|  |  | ZBTB49 |  |
|  |  | ZBTB5 |  |
|  |  | ZBTB6 |  |
|  |  | ZBTB7A |  |
|  |  | ZBTB7C |  |
|  |  | ZBTB8A |  |
|  |  | ZBTB8B |  |
|  |  | ZC2HC1A |  |
|  |  | ZC2HC1C |  |
|  |  | ZC3H11A |  |
|  |  | ZC3H12A |  |
|  |  | ZC3H12B |  |
|  |  | TNFRSF21 |  |
|  |  | ZC3H14 |  |
|  |  | ZC3H15 |  |
|  |  | ZC3H4 |  |
|  |  | ZC3H6 |  |
|  |  | TNIK |  |
|  |  | ZC4H2 |  |
|  |  | ZCCHC11 |  |
|  |  | ZCCHC13 |  |
|  |  | ZCCHC14 |  |
|  |  | ZCCHC16 |  |
|  |  | RP11-80A15.1 |  |
|  |  | RP9 |  |
|  |  | RPA3-AS1 |  |
|  |  | DCN |  |
|  |  | ZDHHC3 |  |
|  |  | ZDHHC4 |  |
|  |  | ZDHHC6 |  |
|  |  | ZDHHC7 |  |
|  |  | TP53BP2 |  |
|  |  | TP53INP1 |  |
|  |  | ZFAND3 |  |
|  |  | ZFAND4 |  |
|  |  | ZFAND5 |  |
|  |  | ZFHX3 |  |
|  |  | ZFP1 |  |
|  |  | ZFP14 |  |
|  |  | ZFP3 |  |
|  |  | ZFP36L1 |  |
|  |  | HSPA14 |  |
|  |  | TPRA1 |  |
|  |  | TPRG1 |  |
|  |  | TPRG1L |  |
|  |  | TPST2 |  |
|  |  | DDX31 |  |
|  |  | HTN1 |  |
|  |  | HTR2C |  |
|  |  | HTR7 |  |
|  |  | DDX60L |  |
|  |  | HYAL1 |  |
|  |  | ZKSCAN3 |  |
|  |  | IARS |  |
|  |  | IBSP |  |
|  |  | CHAC1 |  |
|  |  | CHAF1B |  |
|  |  | ZMYND10 |  |
|  |  | ZMYND12 |  |
|  |  | CHCHD4 |  |
|  |  | CHCHD5 |  |
|  |  | CHCHD7 |  |
|  |  | IFIT3 |  |
|  |  | IFLTD1 |  |
|  |  | CHDH |  |
|  |  | IFNW1 |  |
|  |  | IGF1R |  |
|  |  | IGSF6 |  |
|  |  | LNX1 |  |
|  |  | LNX2 |  |
|  |  | LOH12CR1 |  |
|  |  | CHMP1A |  |
|  |  | LPAR6 |  |
|  |  | LPCAT2 |  |
|  |  | LPGAT1 |  |
|  |  | LPHN2 |  |
|  |  | LPHN3 |  |
|  |  | TRUB1 |  |
|  |  | TSC1 |  |
|  |  | TSC22D1 |  |
|  |  | TSC22D2 |  |
|  |  | TSG101 |  |
|  |  | TSGA10 |  |
|  |  | TSHZ1 |  |
|  |  | TSHZ2 |  |
|  |  | TSHZ3 |  |
|  |  | TSKU |  |
|  |  | TSN |  |
|  |  | TSNAX |  |
|  |  | TSPAN11 |  |
|  |  | TSPAN12 |  |
|  |  | TSPAN13 |  |
|  |  | TSPAN14 |  |
|  |  | TSPAN3 |  |
|  |  | TSPAN5 |  |
|  |  | TSPAN6 |  |
|  |  | TSPAN7 |  |
|  |  | TSPAN8 |  |
|  |  | TSPAN9 |  |
|  |  | TSPYL1 |  |
|  |  | LRRC28 |  |
|  |  | SRF |  |
|  |  | SRFBP1 |  |
|  |  | SRGAP1 |  |
|  |  | LRRC40 |  |
|  |  | LRRC48 |  |
|  |  | LRRC4C |  |
|  |  | LRRC55 |  |
|  |  | LRRC58 |  |
|  |  | LRRC7 |  |
|  |  | LRRC8B |  |
|  |  | LRRC8C |  |
|  |  | LRRFIP2 |  |
|  |  | LRRN1 |  |
|  |  | LRRN3 |  |
|  |  | LRTM1 |  |
|  |  | LSM10 |  |
|  |  | LSM12 |  |
|  |  | LSM14A |  |
|  |  | LSM14B |  |
|  |  | LSM5 |  |
|  |  | LSM6 |  |
|  |  | LSS |  |
|  |  | LTBP2 |  |
|  |  | LTN1 |  |
|  |  | ST13 |  |
|  |  | LUM |  |
|  |  | ST5 |  |
|  |  | LY75-CD302 |  |
|  |  | LY9 |  |
|  |  | IRS2 |  |
|  |  | LYPLA1 |  |
|  |  | LYRM1 |  |
|  |  | LYRM2 |  |
|  |  | LYRM4 |  |
|  |  | LYSMD3 |  |
|  |  | LYVE1 |  |
|  |  | LZTFL1 |  |
|  |  | MAATS1 |  |
|  |  | MAB21L3 |  |
|  |  | STAT3 |  |
|  |  | MAFK |  |
|  |  | MAGEB1 |  |
|  |  | MAGEB4 |  |
|  |  | MAGI1 |  |
|  |  | MAGI2 |  |
|  |  | MAGI3 |  |
|  |  | MAGIX |  |
|  |  | MAGOHB |  |
|  |  | MAK |  |
|  |  | MAK16 |  |
|  |  | MALSU1 |  |
|  |  | MALT1 |  |
|  |  | CKAP4 |  |
|  |  | MAML3 |  |
|  |  | MAMLD1 |  |
|  |  | CKB |  |
|  |  | MAN1A2 |  |
|  |  | MAN1C1 |  |
|  |  | MAN2A1 |  |
|  |  | MAN2A2 |  |
|  |  | MANEA |  |
|  |  | MANF |  |
|  |  | MAOA |  |
|  |  | MAOB |  |
|  |  | MAP10 |  |
|  |  | MAP1LC3B |  |
|  |  | MAP2K4 |  |
|  |  | MAP2K6 |  |
|  |  | MAP3K12 |  |
|  |  | MAP3K19 |  |
|  |  | MAP3K2 |  |
|  |  | MAP3K4 |  |
|  |  | MAP3K5 |  |
|  |  | MAP3K7 |  |
|  |  | MAP3K7CL |  |
|  |  | MAP3K8 |  |
|  |  | MAP3K9 |  |
|  |  | MAP4K2 |  |
|  |  | MAP4K3 |  |
|  |  | MAP4K4 |  |
|  |  | MAP6 |  |
|  |  | MAP7D1 |  |
|  |  | MAP7D3 |  |
|  |  | MAP9 |  |
|  |  | MAPK1 |  |
|  |  | MAPK11 |  |
|  |  | MAPK14 |  |
|  |  | MAPK1IP1L |  |
|  |  | MAPK6 |  |
|  |  | MAPK8 |  |
|  |  | MAPKAPK5 |  |
|  |  | MAPRE1 |  |
|  |  | MAPT |  |
|  |  | MARC1 |  |
|  |  | MARCH1 |  |
|  |  | MARCH3 |  |
|  |  | MARCH5 |  |
|  |  | MARCH6 |  |
|  |  | MARCH7 |  |
|  |  | MARCH8 |  |
|  |  | MARCH9 |  |
|  |  | MARCKS |  |
|  |  | MARCKSL1 |  |
|  |  | MARK1 |  |
|  |  | MARK2 |  |
|  |  | MARS2 |  |
|  |  | CKLF |  |
|  |  | MASTL |  |
|  |  | MAT2A |  |
|  |  | MAT2B |  |
|  |  | MATN2 |  |
|  |  | MATN3 |  |
|  |  | MAVS |  |
|  |  | MBD1 |  |
|  |  | MBD2 |  |
|  |  | MBD5 |  |
|  |  | MBNL1 |  |
|  |  | MBNL2 |  |
|  |  | MBNL3 |  |
|  |  | MBOAT1 |  |
|  |  | MBP |  |
|  |  | MBTD1 |  |
|  |  | MC2R |  |
|  |  | MCAT |  |
|  |  | MCC |  |
|  |  | MCF2L |  |
|  |  | MCFD2 |  |
|  |  | MCHR2 |  |
|  |  | CKS2 |  |
|  |  | MCM3AP |  |
|  |  | KCNT2 |  |
|  |  | CLASP1 |  |
|  |  | CLCC1 |  |
|  |  | CLCN3 |  |
|  |  | MDGA2 |  |
|  |  | PCDHA5 |  |
|  |  | MDM4 |  |
|  |  | MECP2 |  |
|  |  | CLDN1 |  |
|  |  | CLDN10 |  |
|  |  | CLDN12 |  |
|  |  | CLDN8 |  |
|  |  | CLEC12B |  |
|  |  | CLEC14A |  |
|  |  | MED28 |  |
|  |  | MED31 |  |
|  |  | MED4 |  |
|  |  | MED6 |  |
|  |  | MED7 |  |
|  |  | MED9 |  |
|  |  | MEF2A |  |
|  |  | CLIC4 |  |
|  |  | MEIS3 |  |
|  |  | CLK1 |  |
|  |  | CLK4 |  |
|  |  | TBCEL |  |
|  |  | METAP2 |  |
|  |  | METRNL |  |
|  |  | TBPL1 |  |
|  |  | METTL16 |  |
|  |  | TBRG1 |  |
|  |  | TBX1 |  |
|  |  | METTL21C |  |
|  |  | TBX18 |  |
|  |  | TBX19 |  |
|  |  | TBX20 |  |
|  |  | TC2N |  |
|  |  | TCAIM |  |
|  |  | TCEANC |  |
|  |  | TCEANC2 |  |
|  |  | TCP10 |  |
|  |  | TCP11L1 |  |
|  |  | CBR1 |  |
|  |  | TDP1 |  |
|  |  | MFSD9 |  |
|  |  | TEAD1 |  |
|  |  | TEDDM1 |  |
|  |  | TEK |  |
|  |  | TENM1 |  |
|  |  | TENM3 |  |
|  |  | TERF2 |  |
|  |  | CBWD1 |  |
|  |  | CBWD2 |  |
|  |  | CBWD5 |  |
|  |  | CBX3 |  |
|  |  | ESRRG |  |
|  |  | TFCP2L1 |  |
|  |  | EWSR1 |  |
|  |  | CMBL |  |
|  |  | MMP1 |  |
|  |  | CMC1 |  |
|  |  | CMC2 |  |
|  |  | MMP20 |  |
|  |  | MNS1 |  |
|  |  | MOBP |  |
|  |  | MOGAT2 |  |
|  |  | MORC4 |  |
|  |  | MORF4L1 |  |
|  |  | KRBA1 |  |
|  |  | KRT86 |  |
|  |  | KRTAP20-3 |  |
|  |  | KRTAP20-4 |  |
|  |  | KRTAP4-11 |  |
|  |  | KRTAP4-7 |  |
|  |  | KRTAP4-8 |  |
|  |  | KRTAP4-9 |  |
|  |  | KYNU |  |
|  |  | L3HYPDH |  |
|  |  | LACTB |  |
|  |  | LANCL2 |  |
|  |  | LARS |  |
|  |  | LATS2 |  |
|  |  | LBH |  |
|  |  | LCA5 |  |
|  |  | LCP2 |  |
|  |  | LDB1 |  |
|  |  | LDB2 |  |
|  |  | LDHAL6B |  |
|  |  | LDOC1L |  |
|  |  | LEMD3 |  |
|  |  | LENG9 |  |
|  |  | LEPR |  |
|  |  | LFNG |  |
|  |  | LGSN |  |
|  |  | LHFPL3 |  |
|  |  | LILRB2 |  |
|  |  | LILRB4 |  |
|  |  | LIMD2 |  |
|  |  | LINC00908 |  |
|  |  | LIPG |  |
|  |  | LMO4 |  |
|  |  | LMOD1 |  |
|  |  | LOX |  |
|  |  | LPAR2 |  |
|  |  | LPCAT1 |  |
|  |  | LRBA |  |
|  |  | LRCH4 |  |
|  |  | LRIT3 |  |
|  |  | LRP2 |  |
|  |  | LRP3 |  |
|  |  | TMEM68 |  |
|  |  | LRRC16A |  |
|  |  | LRRC19 |  |
|  |  | LRRC42 |  |
|  |  | LRRC49 |  |
|  |  | LRRC61 |  |
|  |  | LRRFIP1 |  |
|  |  | LRRK1 |  |
|  |  | LRRK2 |  |
|  |  | ZNF197 |  |
|  |  | ZNF2 |  |
|  |  | LRTM2 |  |
|  |  | LSAMP |  |
|  |  | LSG1 |  |
|  |  | ZNF226 |  |
|  |  | ZNF229 |  |
|  |  | CNTF |  |
|  |  | CNTN3 |  |
|  |  | CNTN4 |  |
|  |  | CNTN5 |  |
|  |  | CNTNAP2 |  |
|  |  | CNTNAP4 |  |
|  |  | NCBP1 |  |
|  |  | NCF2 |  |
|  |  | NCKAP1L |  |
|  |  | COA1 |  |
|  |  | COA3 |  |
|  |  | COA5 |  |
|  |  | COCH |  |
|  |  | AADACL2 |  |
|  |  | AADACL3 |  |
|  |  | AARSD1 |  |
|  |  | ABCB1 |  |
|  |  | ABCC2 |  |
|  |  | ABCE1 |  |
|  |  | ZNF366 |  |
|  |  | ZNF367 |  |
|  |  | ZNF385D |  |
|  |  | ZNF394 |  |
|  |  | ABLIM2 |  |
|  |  | AC004381.6 |  |
|  |  | ZNF431 |  |
|  |  | ZNF432 |  |
|  |  | ZNF436 |  |
|  |  | ZNF438 |  |
|  |  | ZNF440 |  |
|  |  | ZNF441 |  |
|  |  | ZNF442 |  |
|  |  | ZNF445 |  |
|  |  | ZNF45 |  |
|  |  | ZNF451 |  |
|  |  | ZNF460 |  |
|  |  | ZNF461 |  |
|  |  | ZNF462 |  |
|  |  | ZNF468 |  |
|  |  | ZNF469 |  |
|  |  | AC093157.1 |  |
|  |  | ZNF473 |  |
|  |  | AC104667.3 |  |
|  |  | ZNF486 |  |
|  |  | AC115618.1 |  |
|  |  | AC117395.1 |  |
|  |  | ZNF507 |  |
|  |  | AC137056.1 |  |
|  |  | AC140061.12 |  |
|  |  | TRIM4 |  |
|  |  | ACAD8 |  |
|  |  | ACAD9 |  |
|  |  | ACADL |  |
|  |  | ACP6 |  |
|  |  | ACRC |  |
|  |  | ACRV1 |  |
|  |  | ACSL1 |  |
|  |  | ACSL6 |  |
|  |  | ACSM2A |  |
|  |  | ACSM2B |  |
|  |  | ACTC1 |  |
|  |  | TRPV3 |  |
|  |  | ACTR6 |  |
|  |  | ACVRL1 |  |
|  |  | ACYP2 |  |
|  |  | TSLP |  |
|  |  | ADAMTS12 |  |
|  |  | MCOLN2 |  |
|  |  | MCPH1 |  |
|  |  | TSTD2 |  |
|  |  | TTC14 |  |
|  |  | TTC18 |  |
|  |  | ADCY2 |  |
|  |  | ADD1 |  |
|  |  | ADH7 |  |
|  |  | MED22 |  |
|  |  | MED23 |  |
|  |  | PTPRR |  |
|  |  | SYNPR |  |
|  |  | TUSC5 |  |
|  |  | SYT1 |  |
|  |  | TVP23B |  |
|  |  | TVP23C |  |
|  |  | TWF1 |  |
|  |  | TWIST1 |  |
|  |  | TWISTNB |  |
|  |  | PWWP2B |  |
|  |  | TXNDC15 |  |
|  |  | TXNDC16 |  |
|  |  | TXNL1 |  |
|  |  | TXNL4A |  |
|  |  | TXNRD3 |  |
|  |  | TYRO3 |  |
|  |  | TYSND1 |  |
|  |  | SDR9C7 |  |
|  |  | SEC14L5 |  |
|  |  | SEC22C |  |
|  |  | QTRTD1 |  |
|  |  | RAB10 |  |
|  |  | RAB11FIP3 |  |
|  |  | SEL1L |  |
|  |  | SELL |  |
|  |  | RAB17 |  |
|  |  | SEMA3C |  |
|  |  | SEMA3E |  |
|  |  | RAB23 |  |
|  |  | SEMA6A |  |
|  |  | SEMA6D |  |
|  |  | RAB2A |  |
|  |  | RAB37 |  |
|  |  | UBE3A |  |
|  |  | RAB3B |  |
|  |  | UBLCP1 |  |
|  |  | CKAP2 |  |
|  |  | CKAP2L |  |
|  |  | CLASP2 |  |
|  |  | CLCA2 |  |
|  |  | UFSP2 |  |
|  |  | UGP2 |  |
|  |  | UGT2A1 |  |
|  |  | UGT2A2 |  |
|  |  | UGT2A3 |  |
|  |  | UGT2B11 |  |
|  |  | UGT2B4 |  |
|  |  | UGT3A1 |  |
|  |  | UHMK1 |  |
|  |  | ULK2 |  |
|  |  | UMODL1 |  |
|  |  | UNC13D |  |
|  |  | CLMN |  |
|  |  | CLMP |  |
|  |  | CLN5 |  |
|  |  | CLN8 |  |
|  |  | RANBP3L |  |
|  |  | CLPX |  |
|  |  | CLTC |  |
|  |  | CLTCL1 |  |
|  |  | CLVS1 |  |
|  |  | CLVS2 |  |
|  |  | CMTM3 |  |
|  |  | TEF |  |
|  |  | CNGA2 |  |
|  |  | CNIH4 |  |
|  |  | CNN3 |  |
|  |  | CNOT8 |  |
|  |  | TGFA |  |
|  |  | SIAH1 |  |
|  |  | CNTN6 |  |
|  |  | TGIF2 |  |
|  |  | COA4 |  |
|  |  | COL25A1 |  |
|  |  | COL27A1 |  |
|  |  | SLC10A7 |  |
|  |  | SLC11A2 |  |
|  |  | SLC12A2 |  |
|  |  | COLGALT2 |  |
|  |  | COLQ |  |
|  |  | COPB1 |  |
|  |  | COPS3 |  |
|  |  | COPS4 |  |
|  |  | COPS8 |  |
|  |  | COQ10B |  |
|  |  | CORO1C |  |
|  |  | COX20 |  |
|  |  | COX4I1 |  |
|  |  | COX6C |  |
|  |  | COX7B |  |
|  |  | CPQ |  |
|  |  | CPSF6 |  |
|  |  | CRAMP1L |  |
|  |  | TMED5 |  |
|  |  | TMED7 |  |
|  |  | TMED7-TICAM2 |  |
|  |  | CREBBP |  |
|  |  | CREBL2 |  |
|  |  | NAA40 |  |
|  |  | CRTAM |  |
|  |  | NADK2 |  |
|  |  | NAMPTL |  |
|  |  | NAP1L2 |  |
|  |  | NAP1L3 |  |
|  |  | NAP1L4 |  |
|  |  | TMEM178A |  |
|  |  | RIMBP3B |  |
|  |  | CTC-534A2.2 |  |
|  |  | TMEM196 |  |
|  |  | CTIF |  |
|  |  | CTNNB1 |  |
|  |  | RNF128 |  |
|  |  | RNF135 |  |
|  |  | RNF138 |  |
|  |  | TMEM260 |  |
|  |  | TMEM261 |  |
|  |  | SLC6A20 |  |
|  |  | RNF145 |  |
|  |  | RNF165 |  |
|  |  | TMEM57 |  |
|  |  | TMEM63C |  |
|  |  | TMEM64 |  |
|  |  | RNF32 |  |
|  |  | RNF5 |  |
|  |  | RNF6 |  |
|  |  | RNF7 |  |
|  |  | RNMTL1 |  |
|  |  | RNPC3 |  |
|  |  | RNPEPL1 |  |
|  |  | RNPS1 |  |
|  |  | ROPN1 |  |
|  |  | DAAM1 |  |
|  |  | DAAM2 |  |
|  |  | DACH2 |  |
|  |  | TNFRSF8 |  |
|  |  | DAP |  |
|  |  | DAPK1 |  |
|  |  | DAW1 |  |
|  |  | DBF4 |  |
|  |  | DBNDD2 |  |
|  |  | DBX1 |  |
|  |  | TNPO1 |  |
|  |  | TNRC18 |  |
|  |  | SMIM13 |  |
|  |  | TNRC6B |  |
|  |  | TNRC6C |  |
|  |  | TNS1 |  |
|  |  | TNS3 |  |
|  |  | TOLLIP |  |
|  |  | TOMM6 |  |
|  |  | TOP1 |  |
|  |  | TOR1A |  |
|  |  | RPL10A |  |
|  |  | TPD52 |  |
|  |  | DDB2 |  |
|  |  | DDRGK1 |  |
|  |  | DDX17 |  |
|  |  | ZFPM1 |  |
|  |  | ZFR |  |
|  |  | ZFX |  |
|  |  | DDX4 |  |
|  |  | ZHX1 |  |
|  |  | ZIC1 |  |
|  |  | DEFB106A |  |
|  |  | DEFB106B |  |
|  |  | RSAD2 |  |
|  |  | TRAPPC11 |  |
|  |  | DENR |  |
|  |  | DERA |  |
|  |  | DESI1 |  |
|  |  | DGAT2L6 |  |
|  |  | DGKD |  |
|  |  | DHDDS |  |
|  |  | DHFR |  |
|  |  | DHTKD1 |  |
|  |  | DHX35 |  |
|  |  | DHX58 |  |
|  |  | DIP2A |  |
|  |  | PNMA1 |  |
|  |  | PNMA2 |  |
|  |  | PNP |  |
|  |  | PNPLA4 |  |
|  |  | PNPLA8 |  |
|  |  | PNPO |  |
|  |  | PNPT1 |  |
|  |  | PNRC1 |  |
|  |  | POGK |  |
|  |  | POGLUT1 |  |
|  |  | POGZ |  |
|  |  | POLA1 |  |
|  |  | POLE |  |
|  |  | POLE3 |  |
|  |  | POLH |  |
|  |  | POLI |  |
|  |  | POLR1B |  |
|  |  | POLR2D |  |
|  |  | POLR2H |  |
|  |  | POLR2K |  |
|  |  | POLR2M |  |
|  |  | POLR3D |  |
|  |  | POLR3E |  |
|  |  | POM121L2 |  |
|  |  | POMGNT1 |  |
|  |  | POMGNT2 |  |
|  |  | POMP |  |
|  |  | PON1 |  |
|  |  | PON2 |  |
|  |  | POP1 |  |
|  |  | POP7 |  |
|  |  | POSTN |  |
|  |  | POT1 |  |
|  |  | POTEG |  |
|  |  | POU2F1 |  |
|  |  | POU2F2 |  |
|  |  | POU3F1 |  |
|  |  | POU3F2 |  |
|  |  | POU4F1 |  |
|  |  | SREK1 |  |
|  |  | SCPEP1 |  |
|  |  | SRI |  |
|  |  | SRL |  |
|  |  | SRP19 |  |
|  |  | SRP9 |  |
|  |  | SRPK1 |  |
|  |  | SRPK2 |  |
|  |  | SRR |  |
|  |  | SRSF1 |  |
|  |  | SRSF12 |  |
|  |  | SRSF4 |  |
|  |  | SRXN1 |  |
|  |  | SS18 |  |
|  |  | SEC24B |  |
|  |  | SSR1 |  |
|  |  | SSTR2 |  |
|  |  | SSX2IP |  |
|  |  | ZNF423 |  |
|  |  | ST18 |  |
|  |  | IRAK4 |  |
|  |  | ST8SIA1 |  |
|  |  | ST8SIA6 |  |
|  |  | STAMBP |  |
|  |  | STAR |  |
|  |  | STARD10 |  |
|  |  | STARD4 |  |
|  |  | STARD5 |  |
|  |  | STARD8 |  |
|  |  | STAT1 |  |
|  |  | STAT6 |  |
|  |  | STATH |  |
|  |  | STAU1 |  |
|  |  | STAU2 |  |
|  |  | STC1 |  |
|  |  | STC2 |  |
|  |  | STEAP2 |  |
|  |  | OTOR |  |
|  |  | OTUD6B |  |
|  |  | OVCA2 |  |
|  |  | OXR1 |  |
|  |  | STOX2 |  |
|  |  | STRAP |  |
|  |  | PAAF1 |  |
|  |  | PACSIN2 |  |
|  |  | PAFAH1B2 |  |
|  |  | STX4 |  |
|  |  | STX6 |  |
|  |  | STX7 |  |
|  |  | STXBP3 |  |
|  |  | STXBP4 |  |
|  |  | PALMD |  |
|  |  | PAM |  |
|  |  | PANK4 |  |
|  |  | PAPD7 |  |
|  |  | PARD6B |  |
|  |  | PARD6G |  |
|  |  | PARG |  |
|  |  | PATZ1 |  |
|  |  | PAXBP1 |  |
|  |  | SYT15 |  |
|  |  | SYT16 |  |
|  |  | SYT2 |  |
|  |  | PCDHA1 |  |
|  |  | PCDHA10 |  |
|  |  | PCDHA11 |  |
|  |  | TACO1 |  |
|  |  | TACR1 |  |
|  |  | PCDHA2 |  |
|  |  | TAF1 |  |
|  |  | TAF13 |  |
|  |  | SIX1 |  |
|  |  | SIX2 |  |
|  |  | PCDHA8 |  |
|  |  | SIX6 |  |
|  |  | TAF7L |  |
|  |  | PCGF3 |  |
|  |  | TAF9B |  |
|  |  | PCMTD2 |  |
|  |  | PCNP |  |
|  |  | TAOK1 |  |
|  |  | TAP1 |  |
|  |  | TAP2 |  |
|  |  | PCYT1A |  |
|  |  | PDAP1 |  |
|  |  | PDCD10 |  |
|  |  | PDCD5 |  |
|  |  | SLC15A1 |  |
|  |  | TBC1D32 |  |
|  |  | PDE11A |  |
|  |  | PDE3A |  |
|  |  | PDE4B |  |
|  |  | TBCA |  |
|  |  | PDE6D |  |
|  |  | PDE7B |  |
|  |  | PDE8A |  |
|  |  | SLC1A5 |  |
|  |  | PDGFD |  |
|  |  | SLC20A2 |  |
|  |  | SLC22A15 |  |
|  |  | SLC24A3 |  |
|  |  | SLC24A4 |  |
|  |  | SLC25A13 |  |
|  |  | SLC25A21 |  |
|  |  | SLC25A29 |  |
|  |  | SLC25A32 |  |
|  |  | PEG3 |  |
|  |  | PERP |  |
|  |  | PEX11B |  |
|  |  | PEX26 |  |
|  |  | PEX5 |  |
|  |  | TESPA1 |  |
|  |  | TET1 |  |
|  |  | TET2 |  |
|  |  | PFKFB3 |  |
|  |  | TEX30 |  |
|  |  | PGF |  |
|  |  | TEX9 |  |
|  |  | TFAM |  |
|  |  | TFB2M |  |
|  |  | PGP |  |
|  |  | PGPEP1L |  |
|  |  | PHACTR4 |  |
|  |  | PHAX |  |
|  |  | PHF16 |  |
|  |  | PHF20 |  |
|  |  | PHKG2 |  |
|  |  | THAP9 |  |
|  |  | THBD |  |
|  |  | THBS1 |  |
|  |  | PI4KA |  |
|  |  | PIANP |  |
|  |  | PIAS2 |  |
|  |  | PIGA |  |
|  |  | PIK3AP1 |  |
|  |  | TIMM50 |  |
|  |  | PIK3CG |  |
|  |  | TIMM9 |  |
|  |  | PIK3R3 |  |
|  |  | PIP4K2A |  |
|  |  | PITPNC1 |  |
|  |  | TLE4 |  |
|  |  | PKIG |  |
|  |  | PKP1 |  |
|  |  | PKP2 |  |
|  |  | PKP4 |  |
|  |  | PLA2G2C |  |
|  |  | PLA2G4C |  |
|  |  | PLA2G4E |  |
|  |  | PLAG1 |  |
|  |  | PLCH2 |  |
|  |  | PLCL2 |  |
|  |  | PLEKHA2 |  |
|  |  | PLEKHA6 |  |
|  |  | TMEM109 |  |
|  |  | PLEKHF2 |  |
|  |  | TMEM134 |  |
|  |  | PLEKHO1 |  |
|  |  | TMEM14B |  |
|  |  | TMEM150C |  |
|  |  | TMEM154 |  |
|  |  | PLP1 |  |
|  |  | TMEM164 |  |
|  |  | PLXNA3 |  |
|  |  | PMP2 |  |
|  |  | PNO1 |  |
|  |  | PNRC2 |  |
|  |  | POC1A |  |
|  |  | POC1B-GALNT4 |  |
|  |  | POC5 |  |
|  |  | PODXL |  |
|  |  | TMEM199 |  |
|  |  | POLB |  |
|  |  | TMEM231 |  |
|  |  | POLK |  |
|  |  | POLR1D |  |
|  |  | POLR2B |  |
|  |  | POLR2C |  |
|  |  | POLR3A |  |
|  |  | TMEM33 |  |
|  |  | TMEM35 |  |
|  |  | POM121C |  |
|  |  | POMT2 |  |
|  |  | TMEM67 |  |
|  |  | SP1 |  |
|  |  | POU6F1 |  |
|  |  | PPA1 |  |
|  |  | PPAPDC2 |  |
|  |  | TMEM9B |  |
|  |  | TMF1 |  |
|  |  | TMOD2 |  |
|  |  | PPIL2 |  |
|  |  | TMTC2 |  |
|  |  | SPATS2 |  |
|  |  | PPM1B |  |
|  |  | PPM1D |  |
|  |  | PPM1F |  |
|  |  | PPME1 |  |
|  |  | PPP1R13L |  |
|  |  | PPP1R15A |  |
|  |  | PPP2R1A |  |
|  |  | PPP2R2C |  |
|  |  | PPP2R4 |  |
|  |  | PPP3CA |  |
|  |  | TOX3 |  |
|  |  | TP53BP1 |  |
|  |  | PQLC1 |  |
|  |  | PRDM10 |  |
|  |  | PRDM15 |  |
|  |  | PRDM2 |  |
|  |  | TPK1 |  |
|  |  | TPP1 |  |
|  |  | PREP |  |
|  |  | TPR |  |
|  |  | PRG4 |  |
|  |  | PRIMPOL |  |
|  |  | TRAK1 |  |
|  |  | TRAK2 |  |
|  |  | TRAM1 |  |
|  |  | TRAM1L1 |  |
|  |  | PRKCH |  |
|  |  | PRKCQ |  |
|  |  | PRKRIP1 |  |
|  |  | TRIB1 |  |
|  |  | TRIM10 |  |
|  |  | TRIM13 |  |
|  |  | TRIM33 |  |
|  |  | TRIM35 |  |
|  |  | MAP7D2 |  |
|  |  | TRIM44 |  |
|  |  | TRIM47 |  |
|  |  | TRIM49D1 |  |
|  |  | TRIM52 |  |
|  |  | TRIM59 |  |
|  |  | TRIM65 |  |
|  |  | TRIM71 |  |
|  |  | PRSS21 |  |
|  |  | PRSS23 |  |
|  |  | PRSS54 |  |
|  |  | PRTFDC1 |  |
|  |  | PSKH1 |  |
|  |  | TRPC4 |  |
|  |  | PSMB5 |  |
|  |  | TRPM7 |  |
|  |  | TRPS1 |  |
|  |  | TRPV2 |  |
|  |  | PSPH |  |
|  |  | PTDSS1 |  |
|  |  | PTGDR |  |
|  |  | SUCO |  |
|  |  | TST |  |
|  |  | MCU |  |
|  |  | TTC26 |  |
|  |  | TTC28 |  |
|  |  | TTC3 |  |
|  |  | TTC30A |  |
|  |  | TTC31 |  |
|  |  | TTC33 |  |
|  |  | TTC37 |  |
|  |  | TTC39A |  |
|  |  | TTC39B |  |
|  |  | TTC5 |  |
|  |  | TTC7A |  |
|  |  | TTC9 |  |
|  |  | TTF2 |  |
|  |  | PTPN3 |  |
|  |  | PTPRD |  |
|  |  | TUBA8 |  |
|  |  | PTPRM |  |
|  |  | TUBB8 |  |
|  |  | TUBGCP4 |  |
|  |  | TUBGCP5 |  |
|  |  | TUFT1 |  |
|  |  | TULP4 |  |
|  |  | ZFP37 |  |
|  |  | SYPL1 |  |
|  |  | ZFP42 |  |
|  |  | SYT10 |  |
|  |  | SYT11 |  |
|  |  | SYT13 |  |
|  |  | SYT14 |  |
|  |  | TXK |  |
|  |  | TXLNA |  |
|  |  | TXLNB |  |
|  |  | TXLNG |  |
|  |  | SCRG1 |  |
|  |  | SCRT2 |  |
|  |  | SCYL2 |  |
|  |  | SCYL3 |  |
|  |  | TYRP1 |  |
|  |  | SDHA |  |
|  |  | TYW5 |  |
|  |  | U2SURP |  |
|  |  | UAP1 |  |
|  |  | UBA2 |  |
|  |  | UBA5 |  |
|  |  | UBA6 |  |
|  |  | UBAC2 |  |
|  |  | UBAP2 |  |
|  |  | UBC |  |
|  |  | UBE2A |  |
|  |  | UBE2D1 |  |
|  |  | UBE2D2 |  |
|  |  | UBE2D3 |  |
|  |  | UBE2E2 |  |
|  |  | UBE2F |  |
|  |  | UBE2G1 |  |
|  |  | UBE2H |  |
|  |  | UBE2J1 |  |
|  |  | UBE2K |  |
|  |  | UBE2L6 |  |
|  |  | UBE2N |  |
|  |  | UBE2Q1 |  |
|  |  | UBE2Q2 |  |
|  |  | UBE2T |  |
|  |  | UBE2V1 |  |
|  |  | UBE2V2 |  |
|  |  | UBE2W |  |
|  |  | SENP7 |  |
|  |  | UBFD1 |  |
|  |  | UBL3 |  |
|  |  | SEPN1 |  |
|  |  | UBN2 |  |
|  |  | UBP1 |  |
|  |  | UBR1 |  |
|  |  | UBR3 |  |
|  |  | UBR7 |  |
|  |  | UBXN2A |  |
|  |  | UBXN4 |  |
|  |  | UBXN7 |  |
|  |  | UCHL3 |  |
|  |  | UCHL5 |  |
|  |  | UCK1 |  |
|  |  | UCK2 |  |
|  |  | UCN2 |  |
|  |  | UCP1 |  |
|  |  | UEVLD |  |
|  |  | SERPINE1 |  |
|  |  | RAD23B |  |
|  |  | RAD51C |  |
|  |  | RAD51L3-RFFL |  |
|  |  | RAD52 |  |
|  |  | RAD54L2 |  |
|  |  | UHRF1BP1L |  |
|  |  | SF3A1 |  |
|  |  | RAF1 |  |
|  |  | RAI1 |  |
|  |  | SF3B3 |  |
|  |  | TCF12 |  |
|  |  | TCF20 |  |
|  |  | TCHP |  |
|  |  | TCIRG1 |  |
|  |  | TCL1A |  |
|  |  | TCL1B |  |
|  |  | TCP11L2 |  |
|  |  | TCTA |  |
|  |  | TDRD6 |  |
|  |  | TDRD7 |  |
|  |  | TEC |  |
|  |  | TECPR1 |  |
|  |  | RAPGEFL1 |  |
|  |  | RARG |  |
|  |  | TENM4 |  |
|  |  | TES |  |
|  |  | TESK2 |  |
|  |  | TET3 |  |
|  |  | TEX2 |  |
|  |  | TEX22 |  |
|  |  | TFAP4 |  |
|  |  | TFB1M |  |
|  |  | TFDP2 |  |
|  |  | TFEC |  |
|  |  | TFG |  |
|  |  | TFPI2 |  |
|  |  | TG |  |
|  |  | TGFB1 |  |
|  |  | TGFB2 |  |
|  |  | TGFB3 |  |
|  |  | TGFBR1 |  |
|  |  | TGFBR2 |  |
|  |  | TGFBR3 |  |
|  |  | TGFBRAP1 |  |
|  |  | TGIF1 |  |
|  |  | TGIF2LX |  |
|  |  | TGIF2LY |  |
|  |  | TGM2 |  |
|  |  | TGOLN2 |  |
|  |  | TGS1 |  |
|  |  | THAP1 |  |
|  |  | THAP11 |  |
|  |  | THAP5 |  |
|  |  | THAP6 |  |
|  |  | THBS2 |  |
|  |  | THEGL |  |
|  |  | THEMIS |  |
|  |  | THOC2 |  |
|  |  | THOC5 |  |
|  |  | SLA |  |
|  |  | SLA2 |  |
|  |  | SLAIN1 |  |
|  |  | RBMS1 |  |
|  |  | SLAIN2 |  |
|  |  | RBMS3 |  |
|  |  | TIGD6 |  |
|  |  | TIMM10B |  |
|  |  | TIMM17A |  |
|  |  | TIMM22 |  |
|  |  | TIMM23 |  |
|  |  | TIMM8A |  |
|  |  | TIMMDC1 |  |
|  |  | TIMP3 |  |
|  |  | TIMP4 |  |
|  |  | TINAGL1 |  |
|  |  | TIPARP |  |
|  |  | TIPRL |  |
|  |  | TJP1 |  |
|  |  | TK2 |  |
|  |  | TKTL1 |  |
|  |  | TKTL2 |  |
|  |  | TLK1 |  |
|  |  | REG3G |  |
|  |  | TLN2 |  |
|  |  | TLR4 |  |
|  |  | TLR6 |  |
|  |  | TLR7 |  |
|  |  | TM2D2 |  |
|  |  | REXO1 |  |
|  |  | SLC23A2 |  |
|  |  | TM9SF3 |  |
|  |  | TM9SF4 |  |
|  |  | TMCC1 |  |
|  |  | TMCC3 |  |
|  |  | TMCO5A |  |
|  |  | TMED1 |  |
|  |  | TMED2 |  |
|  |  | TMED3 |  |
|  |  | TMEFF1 |  |
|  |  | TMEFF2 |  |
|  |  | CRIM1 |  |
|  |  | CRLF1 |  |
|  |  | TMEM133 |  |
|  |  | CS |  |
|  |  | CSDE1 |  |
|  |  | CSNK1A1L |  |
|  |  | TMEM169 |  |
|  |  | TMEM170B |  |
|  |  | TMEM175 |  |
|  |  | TMEM177 |  |
|  |  | RIMBP3 |  |
|  |  | SLC35D1 |  |
|  |  | RIMBP3C |  |
|  |  | RIMKLB |  |
|  |  | TMEM184B |  |
|  |  | TMEM185B |  |
|  |  | TMEM189 |  |
|  |  | TMEM189-UBE2V1 | |
|  |  | TMEM194B |  |
|  |  | TMEM2 |  |
|  |  | TMEM200A |  |
|  |  | TMEM200C |  |
|  |  | TMEM203 |  |
|  |  | TMEM218 |  |
|  |  | TMEM220 |  |
|  |  | TMEM222 |  |
|  |  | TMEM233 |  |
|  |  | TMEM236 |  |
|  |  | TMEM237 |  |
|  |  | TMEM245 |  |
|  |  | TMEM246 |  |
|  |  | TMEM248 |  |
|  |  | TMEM25 |  |
|  |  | SLC5A3 |  |
|  |  | TMEM256-PLSCR3 | |
|  |  | TMEM257 |  |
|  |  | TMEM26 |  |
|  |  | SLC6A14 |  |
|  |  | SLC6A15 |  |
|  |  | TMEM38B |  |
|  |  | TMEM39B |  |
|  |  | TMEM41B |  |
|  |  | TMEM43 |  |
|  |  | TMEM45A |  |
|  |  | TMEM47 |  |
|  |  | TMEM50B |  |
|  |  | TMEM55A |  |
|  |  | TMEM56 |  |
|  |  | SLC8A3 |  |
|  |  | TMEM60 |  |
|  |  | TMEM63A |  |
|  |  | SLC9A4 |  |
|  |  | SLC9A6 |  |
|  |  | TMEM65 |  |
|  |  | TMEM70 |  |
|  |  | TMEM74 |  |
|  |  | TMEM80 |  |
|  |  | TMEM87B |  |
|  |  | TMEM97 |  |
|  |  | TMOD3 |  |
|  |  | TMPO |  |
|  |  | TMPRSS11A |  |
|  |  | TMPRSS11B |  |
|  |  | TMPRSS11D |  |
|  |  | TMPRSS4 |  |
|  |  | TMPRSS5 |  |
|  |  | TMTC1 |  |
|  |  | TMTC3 |  |
|  |  | TMX1 |  |
|  |  | TMX3 |  |
|  |  | TMX4 |  |
|  |  | TNFAIP1 |  |
|  |  | TNFAIP3 |  |
|  |  | TNFAIP8 |  |
|  |  | TNFRSF10D |  |
|  |  | TNFRSF13C |  |
|  |  | TNFRSF1B |  |
|  |  | SMARCA4 |  |
|  |  | TNFRSF9 |  |
|  |  | TNFSF11 |  |
|  |  | TNFSF15 |  |
|  |  | TNFSF4 |  |
|  |  | TNFSF8 |  |
|  |  | TNKS |  |
|  |  | TNKS2 |  |
|  |  | TNMD |  |
|  |  | TNNI1 |  |
|  |  | SMG6 |  |
|  |  | SMIM12 |  |
|  |  | TEX101 |  |
|  |  | SMIM15 |  |
|  |  | SMIM17 |  |
|  |  | SMIM19 |  |
|  |  | SMIM20 |  |
|  |  | SMIM21 |  |
|  |  | SMIM6 |  |
|  |  | SMIM8 |  |
|  |  | RPAP3 |  |
|  |  | RPE |  |
|  |  | TOR1B |  |
|  |  | TOX |  |
|  |  | TOX4 |  |
|  |  | TP53INP2 |  |
|  |  | TP63 |  |
|  |  | TPBG |  |
|  |  | TPD52L2 |  |
|  |  | TPGS2 |  |
|  |  | TPH1 |  |
|  |  | TPH2 |  |
|  |  | TPI1 |  |
|  |  | ZFP36L2 |  |
|  |  | ZFP41 |  |
|  |  | ZFP62 |  |
|  |  | ZFP90 |  |
|  |  | RPS6KL1 |  |
|  |  | RPUSD4 |  |
|  |  | ZFY |  |
|  |  | ZFYVE1 |  |
|  |  | ZFYVE16 |  |
|  |  | ZFYVE21 |  |
|  |  | ZIC2 |  |
|  |  | ZIC3 |  |
|  |  | TRAM2 |  |
|  |  | ZMYM1 |  |
|  |  | ZMYM2 |  |
|  |  | ZMYM5 |  |
|  |  | ZMYM6 |  |
|  |  | ZMYM6NB |  |
|  |  | TREML2 |  |
|  |  | TRERF1 |  |
|  |  | ZMYND8 |  |
|  |  | ZNF10 |  |
|  |  | ZNF100 |  |
|  |  | ZNF106 |  |
|  |  | TRIM16 |  |
|  |  | ZNF140 |  |
|  |  | ZNF141 |  |
|  |  | ZNF143 |  |
|  |  | ZNF146 |  |
|  |  | ZNF148 |  |
|  |  | ZNF169 |  |
|  |  | ZNF17 |  |
|  |  | ZNF174 |  |
|  |  | ZNF175 |  |
|  |  | ZNF177 |  |
|  |  | ZNF180 |  |
|  |  | ZNF181 |  |
|  |  | ZNF182 |  |
|  |  | DIRAS2 |  |
|  |  | SPARCL1 |  |
|  |  | SPAST |  |
|  |  | SPATA13 |  |
|  |  | SPATA16 |  |
|  |  | SPATA18 |  |
|  |  | SPATA2 |  |
|  |  | SPATA4 |  |
|  |  | SPATA5L1 |  |
|  |  | SPATA6 |  |
|  |  | SPATA6L |  |
|  |  | SPATS2L |  |
|  |  | SPCS1 |  |
|  |  | SPCS2 |  |
|  |  | SPECC1 |  |
|  |  | SCAF11 |  |
|  |  | SPG20 |  |
|  |  | SPHAR |  |
|  |  | SPIB |  |
|  |  | SPIDR |  |
|  |  | SPIN1 |  |
|  |  | SPIRE1 |  |
|  |  | SPN |  |
|  |  | SPOCK1 |  |
|  |  | SPOP |  |
|  |  | SPOPL |  |
|  |  | SPPL2A |  |
|  |  | SPRED1 |  |
|  |  | SPRTN |  |
|  |  | SPRY1 |  |
|  |  | SPRY3 |  |
|  |  | SPRYD4 |  |
|  |  | SPRYD7 |  |
|  |  | SPTA1 |  |
|  |  | SPTBN2 |  |
|  |  | SPTLC1 |  |
|  |  | SPTLC3 |  |
|  |  | SRD5A1 |  |
|  |  | SRD5A3 |  |
|  |  | SREBF2 |  |
|  |  | ING4 |  |
|  |  | INHBA |  |
|  |  | ZNF317 |  |
|  |  | SCRN3 |  |
|  |  | SCRT1 |  |
|  |  | INPP4A |  |
|  |  | INPP5A |  |
|  |  | INSC |  |
|  |  | INSIG1 |  |
|  |  | INTS12 |  |
|  |  | INTS7 |  |
|  |  | SEC14L1 |  |
|  |  | IP6K2 |  |
|  |  | IPCEF1 |  |
|  |  | IPO11 |  |
|  |  | IQCJ |  |
|  |  | SECISBP2 |  |
|  |  | SEH1L |  |
|  |  | SELK |  |
|  |  | IRF2BPL |  |
|  |  | SEMA3D |  |
|  |  | IRS4 |  |
|  |  | ISG20 |  |
|  |  | ISL1 |  |
|  |  | SEPSECS |  |
|  |  | ITGAV |  |
|  |  | ITGB1BP1 |  |
|  |  | STEAP4 |  |
|  |  | STIL |  |
|  |  | STIM2 |  |
|  |  | STK10 |  |
|  |  | STK17B |  |
|  |  | STK24 |  |
|  |  | STK25 |  |
|  |  | STK3 |  |
|  |  | STK33 |  |
|  |  | STK36 |  |
|  |  | STK38L |  |
|  |  | STK39 |  |
|  |  | STK4 |  |
|  |  | STK40 |  |
|  |  | STMN1 |  |
|  |  | STMN3 |  |
|  |  | STON1 |  |
|  |  | STON2 |  |
|  |  | STRBP |  |
|  |  | JAM2 |  |
|  |  | STX16 |  |
|  |  | STX17 |  |
|  |  | STX2 |  |
|  |  | STX3 |  |
|  |  | JOSD1 |  |
|  |  | KALRN |  |
|  |  | KANK2 |  |
|  |  | SUCLA2 |  |
|  |  | SUCLG2 |  |
|  |  | SUCNR1 |  |
|  |  | SUDS3 |  |
|  |  | SUGT1 |  |
|  |  | SULF2 |  |
|  |  | SULT1B1 |  |
|  |  | SULT4A1 |  |
|  |  | SUMF2 |  |
|  |  | SUMO1 |  |
|  |  | SUMO2 |  |
|  |  | SUMO3 |  |
|  |  | SUMO4 |  |
|  |  | SUPT3H |  |
|  |  | SURF4 |  |
|  |  | SUSD1 |  |
|  |  | SUV39H2 |  |
|  |  | SUV420H1 |  |
|  |  | SUZ12 |  |
|  |  | SV2B |  |
|  |  | SV2C |  |
|  |  | SVIP |  |
|  |  | SVOP |  |
|  |  | SWAP70 |  |
|  |  | SYAP1 |  |
|  |  | SYN1 |  |
|  |  | SYNCRIP |  |
|  |  | SYNDIG1L |  |
|  |  | SYNGAP1 |  |
|  |  | SYNJ2 |  |
|  |  | SYNJ2BP |  |
|  |  | SYNPO2 |  |
|  |  | SYT4 |  |
|  |  | SYT7 |  |
|  |  | SYT8 |  |
|  |  | SYTL5 |  |
|  |  | T |  |
|  |  | TAB1 |  |
|  |  | TAB2 |  |
|  |  | TAB3 |  |
|  |  | TACC1 |  |
|  |  | TACC2 |  |
|  |  | ZNF813 |  |
|  |  | ZNF816 |  |
|  |  | TADA2A |  |
|  |  | ZNF830 |  |
|  |  | KDELR2 |  |
|  |  | TAF3 |  |
|  |  | KDM2A |  |
|  |  | KDM4A |  |
|  |  | TAF8 |  |
|  |  | KDM5B |  |
|  |  | SKIL |  |
|  |  | TAL1 |  |
|  |  | TALDO1 |  |
|  |  | TANC1 |  |
|  |  | TANC2 |  |
|  |  | TAPBP |  |
|  |  | SLC10A2 |  |
|  |  | SLC10A6 |  |
|  |  | TASP1 |  |
|  |  | TBC1D1 |  |
|  |  | SLC12A6 |  |
|  |  | TBC1D14 |  |
|  |  | KIAA0930 |  |
|  |  | SLC15A2 |  |
|  |  | SLC15A5 |  |
|  |  | TBC1D5 |  |
|  |  | TBC1D8B |  |
|  |  | TBC1D9 |  |
|  |  | TBC1D9B |  |
|  |  | SLC16A9 |  |
|  |  | SLC17A6 |  |
|  |  | SLC17A8 |  |
|  |  | SLC18A2 |  |
|  |  | SLC19A2 |  |
|  |  | SLC1A1 |  |
|  |  | SLC1A2 |  |
|  |  | SLC1A3 |  |
|  |  | SLC20A1 |  |
|  |  | METTL4 |  |
|  |  | SLC22A23 |  |
|  |  | SLC22A3 |  |
|  |  | SLC22A5 |  |
|  |  | SLC22A7 |  |
|  |  | MFAP2 |  |
|  |  | SLC25A1 |  |
|  |  | SLC25A12 |  |
|  |  | SLC25A15 |  |
|  |  | MFSD1 |  |
|  |  | SLC25A21-AS1 |  |
|  |  | MFSD4 |  |
|  |  | SLC25A3 |  |
|  |  | SLC25A33 |  |
|  |  | SLC25A36 |  |
|  |  | SLC25A38 |  |
|  |  | SLC25A4 |  |
|  |  | SLC25A40 |  |
|  |  | SLC25A46 |  |
|  |  | SLC25A51 |  |
|  |  | SLC25A53 |  |
|  |  | SLC26A2 |  |
|  |  | SLC26A7 |  |
|  |  | SLC27A2 |  |
|  |  | SLC2A1 |  |
|  |  | SLC2A11 |  |
|  |  | SLC2A12 |  |
|  |  | MID2 |  |
|  |  | MIEF2 |  |
|  |  | MIS18BP1 |  |
|  |  | TEX35 |  |
|  |  | MKI67 |  |
|  |  | MKKS |  |
|  |  | SLC35B4 |  |
|  |  | TFIP11 |  |
|  |  | TFPI |  |
|  |  | TFRC |  |
|  |  | TGDS |  |
|  |  | TGFB1I1 |  |
|  |  | RPS6KA1 |  |
|  |  | THAP4 |  |
|  |  | THEM4 |  |
|  |  | THG1L |  |
|  |  | THY1 |  |
|  |  | TIFA |  |
|  |  | TIFAB |  |
|  |  | TIGD4 |  |
|  |  | TIGIT |  |
|  |  | TIRAP |  |
|  |  | TLDC1 |  |
|  |  | TLDC2 |  |
|  |  | SMAD1 |  |
|  |  | SMAD2 |  |
|  |  | TLR3 |  |
|  |  | TM4SF1 |  |
|  |  | TM4SF5 |  |
|  |  | TMBIM1 |  |
|  |  | TMC4 |  |
|  |  | SMC2 |  |
|  |  | TMED10 |  |
|  |  | SMIM2 |  |
|  |  | TMEM106B |  |
|  |  | TMEM106C |  |
|  |  | SMLR1 |  |
|  |  | TMEM128 |  |
|  |  | TMEM129 |  |
|  |  | TMEM132B |  |
|  |  | TMEM132D |  |
|  |  | SMPD1 |  |
|  |  | TMEM136 |  |
|  |  | TMEM143 |  |
|  |  | TMEM144 |  |
|  |  | TMEM14A |  |
|  |  | TMEM14C |  |
|  |  | TMEM159 |  |
|  |  | TMEM165 |  |
|  |  | TMEM167A |  |
|  |  | SNN |  |
|  |  | TMEM168 |  |
|  |  | TMEM17 |  |
|  |  | SNTB1 |  |
|  |  | SNTB2 |  |
|  |  | SNX10 |  |
|  |  | SNX11 |  |
|  |  | SNX29 |  |
|  |  | TMEM192 |  |
|  |  | ZDHHC8 |  |
|  |  | TMEM217 |  |
|  |  | TMEM243 |  |
|  |  | TMEM252 |  |
|  |  | TMEM253 |  |
|  |  | TMEM255B |  |
|  |  | TMEM30A |  |
|  |  | SOX9 |  |
|  |  | TMEM81 |  |
|  |  | TMEM86A |  |
|  |  | TMEM92 |  |
|  |  | TMEM98 |  |
|  |  | ZMYND11 |  |
|  |  | ZMYND19 |  |
|  |  | TMPPE |  |
|  |  | TMPRSS11BNL |  |
|  |  | TMSB4Y |  |
|  |  | TMUB2 |  |
|  |  | TNFAIP2 |  |
|  |  | TNFAIP8L1 |  |
|  |  | TNFRSF10A |  |
|  |  | TNFRSF10B |  |
|  |  | TNFRSF11A |  |
|  |  | TNFSF18 |  |
|  |  | TNNC1 |  |
|  |  | TNNC2 |  |
|  |  | TNPO3 |  |
|  |  | TNR |  |
|  |  | TOMM22 |  |
|  |  | TOMM40 |  |
|  |  | TOMM70A |  |
|  |  | TOR1AIP1 |  |
|  |  | TOR1AIP2 |  |
|  |  | TP53RK |  |
|  |  | MAGEB3 |  |
|  |  | TPPP |  |
|  |  | TPT1 |  |
|  |  | MAMDC2 |  |
|  |  | TRABD2B |  |
|  |  | TRAF3 |  |
|  |  | TRAF3IP1 |  |
|  |  | TRAF5 |  |
|  |  | TRAF6 |  |
|  |  | MAN1B1 |  |
|  |  | TRAPPC13 |  |
|  |  | TRAPPC3L |  |
|  |  | TRAPPC6B |  |
|  |  | TRAPPC8 |  |
|  |  | TRAT1 |  |
|  |  | TRDMT1 |  |
|  |  | TRDN |  |
|  |  | TREM1 |  |
|  |  | TREML1 |  |
|  |  | TRHDE |  |
|  |  | MAP3K13 |  |
|  |  | TRIM11 |  |
|  |  | TRIM14 |  |
|  |  | TRIM2 |  |
|  |  | TRIM22 |  |
|  |  | TRIM23 |  |
|  |  | TRIM24 |  |
|  |  | TRIM25 |  |
|  |  | TRIM27 |  |
|  |  | TRIM32 |  |
|  |  | MAP7 |  |
|  |  | TRIM63 |  |
|  |  | MAPKAPK2 |  |
|  |  | TRIM9 |  |
|  |  | TRIO |  |
|  |  | TRIP11 |  |
|  |  | TRIP12 |  |
|  |  | TRIP4 |  |
|  |  | TRIP6 |  |
|  |  | TRIQK |  |
|  |  | TRMT10A |  |
|  |  | TRMT10C |  |
|  |  | TRMT11 |  |
|  |  | TRMT13 |  |
|  |  | TRMT1L |  |
|  |  | TRMT5 |  |
|  |  | TROVE2 |  |
|  |  | TRPA1 |  |
|  |  | TRPC1 |  |
|  |  | TRPM1 |  |
|  |  | TRPM3 |  |
|  |  | MATR3 |  |
|  |  | MAU2 |  |
|  |  | MBL2 |  |
|  |  | TSEN15 |  |
|  |  | TSEN34 |  |
|  |  | TSNARE1 |  |
|  |  | TSPAN2 |  |
|  |  | TSPAN33 |  |
|  |  | MCTP2 |  |
|  |  | RP11-422N16.3 |  |
|  |  | SULF1 |  |
|  |  | MDH1 |  |
|  |  | MDH1B |  |
|  |  | MDH2 |  |
|  |  | MDN1 |  |
|  |  | ME1 |  |
|  |  | MEA1 |  |
|  |  | MEAF6 |  |
|  |  | TTK |  |
|  |  | TTL |  |
|  |  | TTLL2 |  |
|  |  | TTLL7 |  |
|  |  | TTLL9 |  |
|  |  | TTN |  |
|  |  | TTPA |  |
|  |  | TTPAL |  |
|  |  | TTYH2 |  |
|  |  | TTYH3 |  |
|  |  | TUBB1 |  |
|  |  | TUBB2A |  |
|  |  | TUBB4B |  |
|  |  | SYNGR2 |  |
|  |  | SYNJ1 |  |
|  |  | SYNPO |  |
|  |  | RPS27L |  |
|  |  | KLHL21 |  |
|  |  | RPS6KA3 |  |
|  |  | SNRPD3 |  |
|  |  | SNRPN |  |
|  |  | SYT6 |  |
|  |  | SCO1 |  |
|  |  | SCOC |  |
|  |  | RRAS2 |  |
|  |  | TRAF3IP2 |  |
|  |  | SZT2 |  |
|  |  | SDE2 |  |
|  |  | SEC23A |  |
|  |  | SEC23IP |  |
|  |  | SEC24A |  |
|  |  | SEC61A2 |  |
|  |  | SEC62 |  |
|  |  | SEC63 |  |
|  |  | TAF4 |  |
|  |  | RTP1 |  |
|  |  | SELPLG |  |
|  |  | SELRC1 |  |
|  |  | SELT |  |
|  |  | SEMA3A |  |
|  |  | TAL2 |  |
|  |  | SEMA3G |  |
|  |  | SENP1 |  |
|  |  | SENP2 |  |
|  |  | SENP5 |  |
|  |  | SENP6 |  |
|  |  | TARDBP |  |
|  |  | SENP8 |  |
|  |  | SEPHS1 |  |
|  |  | SEPP1 |  |
|  |  | SEPT10 |  |
|  |  | SEPT11 |  |
|  |  | SEPT7 |  |
|  |  | SERF2 |  |
|  |  | SERINC5 |  |
|  |  | SERPINB2 |  |
|  |  | RABGAP1 |  |
|  |  | SERPINB9 |  |
|  |  | SERPINH1 |  |
|  |  | TBL2 |  |
|  |  | TBX3 |  |
|  |  | RADIL |  |
|  |  | TCEA1 |  |
|  |  | RAI14 |  |
|  |  | IL6R |  |
|  |  | RALYL |  |
|  |  | SGCZ |  |
|  |  | SGIP1 |  |
|  |  | SGK1 |  |
|  |  | ING3 |  |
|  |  | SGTB |  |
|  |  | SH2B3 |  |
|  |  | SH2D4A |  |
|  |  | SH2D4B |  |
|  |  | SH3BGRL2 |  |
|  |  | SH3BP4 |  |
|  |  | RASGEF1B |  |
|  |  | RASL11B |  |
|  |  | RASSF4 |  |
|  |  | RASSF5 |  |
|  |  | SHISA2 |  |
|  |  | RASSF8 |  |
|  |  | SHISA6 |  |
|  |  | SHMT1 |  |
|  |  | RBBP4 |  |
|  |  | RBL1 |  |
|  |  | SIK1 |  |
|  |  | RBM14 |  |
|  |  | RBM14-RBM4 |  |
|  |  | RBM15B |  |
|  |  | SIRT1 |  |
|  |  | SKIDA1 |  |
|  |  | SKOR1 |  |
|  |  | SKP1 |  |
|  |  | SKP2 |  |
|  |  | SERP1 |  |
|  |  | ITIH5 |  |
|  |  | SLAMF1 |  |
|  |  | SLC10A5 |  |
|  |  | ITPK1 |  |
|  |  | ITPKC |  |
|  |  | SLC12A5 |  |
|  |  | RCAN2 |  |
|  |  | RCBTB1 |  |
|  |  | RCC2 |  |
|  |  | RCL1 |  |
|  |  | SLC16A1 |  |
|  |  | RCVRN |  |
|  |  | RD3L |  |
|  |  | RDH14 |  |
|  |  | REEP1 |  |
|  |  | SLC19A1 |  |
|  |  | RELL2 |  |
|  |  | RELN |  |
|  |  | REPS1 |  |
|  |  | REPS2 |  |
|  |  | RER1 |  |
|  |  | SLC22A18AS |  |
|  |  | RFFL |  |
|  |  | RFPL3S |  |
|  |  | SLC25A24 |  |
|  |  | RFX5 |  |
|  |  | SLC25A44 |  |
|  |  | TMEM11 |  |
|  |  | TMEM126B |  |
|  |  | TMEM130 |  |
|  |  | TMEM131 |  |
|  |  | SLC35A1 |  |
|  |  | RIBC2 |  |
|  |  | SLC35C1 |  |
|  |  | SLC35E1 |  |
|  |  | SLC35E4 |  |
|  |  | SLC35F5 |  |
|  |  | SLC38A1 |  |
|  |  | RMDN2 |  |
|  |  | RNASEL |  |
|  |  | RND3 |  |
|  |  | SLC4A7 |  |
|  |  | SLC5A12 |  |
|  |  | SLC5A8 |  |
|  |  | SLC6A1 |  |
|  |  | SLC6A11 |  |
|  |  | SLC6A4 |  |
|  |  | SLC6A5 |  |
|  |  | SLC6A7 |  |
|  |  | SLC6A8 |  |
|  |  | SLC7A11 |  |
|  |  | SLC7A14 |  |
|  |  | SLC7A2 |  |
|  |  | SLC7A5 |  |
|  |  | SLC7A6 |  |
|  |  | SLC7A6OS |  |
|  |  | SLC8A1 |  |
|  |  | SLC16A4 |  |
|  |  | SLC8B1 |  |
|  |  | SLC9A2 |  |
|  |  | SLC9A7 |  |
|  |  | SLC9C2 |  |
|  |  | SLCO1A2 |  |
|  |  | SLCO1B3 |  |
|  |  | SLCO1C1 |  |
|  |  | SLCO2A1 |  |
|  |  | SLCO5A1 |  |
|  |  | SLFN12 |  |
|  |  | SLFN12L |  |
|  |  | SLIT1 |  |
|  |  | SLIT3 |  |
|  |  | SLITRK1 |  |
|  |  | SLITRK4 |  |
|  |  | SLK |  |
|  |  | SLMAP |  |
|  |  | SLMO1 |  |
|  |  | SLMO2 |  |
|  |  | SLTM |  |
|  |  | SLU7 |  |
|  |  | SLX4IP |  |
|  |  | SMAD4 |  |
|  |  | RP11-113D6.10 |  |
|  |  | SMARCA5 |  |
|  |  | RP11-176H8.1 |  |
|  |  | SMC4 |  |
|  |  | RP11-520P18.5 |  |
|  |  | SMG1 |  |
|  |  | SMIM14 |  |
|  |  | SLC30A6 |  |
|  |  | SLC30A9 |  |
|  |  | SLC33A1 |  |
|  |  | SLC35A5 |  |
|  |  | RPGR |  |
|  |  | SMS |  |
|  |  | SMTNL2 |  |
|  |  | SMU1 |  |
|  |  | SMUG1 |  |
|  |  | SMYD4 |  |
|  |  | SNAP29 |  |
|  |  | KLHDC8A |  |
|  |  | RPS12 |  |
|  |  | RPS21 |  |
|  |  | KLHL2 |  |
|  |  | KLHL20 |  |
|  |  | TPM1 |  |
|  |  | KLHL22 |  |
|  |  | KLHL26 |  |
|  |  | SLC41A1 |  |
|  |  | SLC41A2 |  |
|  |  | SLC43A2 |  |
|  |  | SNTG1 |  |
|  |  | RRP1B |  |
|  |  | RRP7A |  |
|  |  | SOCS2 |  |
|  |  | SOCS4 |  |
|  |  | RSPH9 |  |
|  |  | RSPRY1 |  |
|  |  | RTF1 |  |
|  |  | RTN4RL1 |  |
|  |  | RUNDC3B |  |
|  |  | RUNX2 |  |
|  |  | RWDD4 |  |
|  |  | RXRA |  |
|  |  | RYR2 |  |
|  |  | S1PR1 |  |
|  |  | SAFB |  |
|  |  | ZNF189 |  |
|  |  | ZNF20 |  |
|  |  | ZNF200 |  |
|  |  | ZNF207 |  |
|  |  | ZNF208 |  |
|  |  | ZNF215 |  |
|  |  | ZNF217 |  |
|  |  | ZNF22 |  |
|  |  | IKZF5 |  |
|  |  | IL17RB |  |
|  |  | IL18BP |  |
|  |  | ZNF275 |  |
|  |  | IMPG1 |  |
|  |  | ING1 |  |
|  |  | ING2 |  |
|  |  | ZNF318 |  |
|  |  | SCUBE3 |  |
|  |  | SDC2 |  |
|  |  | SDC3 |  |
|  |  | SDCBP |  |
|  |  | SDF4 |  |
|  |  | SDHAF1 |  |
|  |  | SDHC |  |
|  |  | SDK2 |  |
|  |  | SDPR |  |
|  |  | SEC11A |  |
|  |  | TTC8 |  |
|  |  | SEC14L2 |  |
|  |  | SEC16A |  |
|  |  | SEC22A |  |
|  |  | SEC23B |  |
|  |  | SEC24D |  |
|  |  | SEC31B |  |
|  |  | SECISBP2L |  |
|  |  | TUSC3 |  |
|  |  | SEMA5A |  |
|  |  | SEP15 |  |
|  |  | TXNRD3NB |  |
|  |  | SERBP1 |  |
|  |  | SERINC1 |  |
|  |  | SERINC3 |  |
|  |  | SOX5 |  |
|  |  | SOX6 |  |
|  |  | ZNF503 |  |
|  |  | SERPINB1 |  |
|  |  | SP110 |  |
|  |  | ITM2A |  |
|  |  | ZMYM4 |  |
|  |  | SP7 |  |
|  |  | SP8 |  |
|  |  | ITPR3 |  |
|  |  | IVNS1ABP |  |
|  |  | JAK1 |  |
|  |  | SETD9 |  |
|  |  | SETDB2 |  |
|  |  | SF3B1 |  |
|  |  | JMJD7 |  |
|  |  | SFRP2 |  |
|  |  | SFT2D2 |  |
|  |  | SFT2D3 |  |
|  |  | SFTPB |  |
|  |  | SFXN1 |  |
|  |  | SFXN4 |  |
|  |  | SFXN5 |  |
|  |  | SGCB |  |
|  |  | SGCD |  |
|  |  | SGCE |  |
|  |  | KAT2A |  |
|  |  | LTBP1 |  |
|  |  | KBTBD7 |  |
|  |  | KCNAB1 |  |
|  |  | ZNF696 |  |
|  |  | KCNJ16 |  |
|  |  | SHISA5 |  |
|  |  | SHISA9 |  |
|  |  | KCNK2 |  |
|  |  | ZNF418 |  |
|  |  | SIK3 |  |
|  |  | SIKE1 |  |
|  |  | SIM2 |  |
|  |  | SIN3A |  |
|  |  | SIPA1L1 |  |
|  |  | SIPA1L2 |  |
|  |  | SIX3 |  |
|  |  | SIX4 |  |
|  |  | SKA2 |  |
|  |  | SKAP2 |  |
|  |  | KIAA0196 |  |
|  |  | VASP |  |
|  |  | STAT5B |  |
|  |  | KIAA0825 |  |
|  |  | SLC14A1 |  |
|  |  | KIAA0947 |  |
|  |  | VEGFA |  |
|  |  | MERTK |  |
|  |  | METTL15 |  |
|  |  | METTL20 |  |
|  |  | VPS13D |  |
|  |  | MEX3A |  |
|  |  | VPS52 |  |
|  |  | MFF |  |
|  |  | MFSD3 |  |
|  |  | CLSTN2 |  |
|  |  | CLUAP1 |  |
|  |  | SMARCA2 |  |
|  |  | CLYBL |  |
|  |  | WBP2NL |  |
|  |  | SLC2A14 |  |
|  |  | SLC2A3 |  |
|  |  | SLC2A9 |  |
|  |  | SLC30A1 |  |
|  |  | SUN2 |  |
|  |  | SLC30A7 |  |
|  |  | SURF2 |  |
|  |  | MLANA |  |
|  |  | MLF1 |  |
|  |  | MLF2 |  |
|  |  | KLHDC10 |  |
|  |  | KLHDC2 |  |
|  |  | SYNDIG1 |  |
|  |  | KLHL1 |  |
|  |  | KLHL15 |  |
|  |  | SNAPIN |  |
|  |  | SNCAIP |  |
|  |  | SLC39A11 |  |
|  |  | TPM3 |  |
|  |  | SNRNP48 |  |
|  |  | KLHL8 |  |
|  |  | SYTL2 |  |
|  |  | SNX16 |  |
|  |  | SNX18 |  |
|  |  | KMT2C |  |
|  |  | XBP1 |  |
|  |  | XCL1 |  |
|  |  | XCR1 |  |
|  |  | XIRP2 |  |
|  |  | XK |  |
|  |  | XKR7 |  |
|  |  | SORD |  |
|  |  | XXYLT1 |  |
|  |  | YARS2 |  |
|  |  | YEATS2 |  |
|  |  | YIPF6 |  |
|  |  | YPEL4 |  |
|  |  | YTHDC2 |  |
|  |  | YWHAQ |  |
|  |  | YWHAZ |  |
|  |  | Z98049.1 |  |
|  |  | ZBED2 |  |
|  |  | ZBTB2 |  |
|  |  | ZBTB24 |  |
|  |  | TCEAL5 |  |
|  |  | ZBTB39 |  |
|  |  | ZBTB43 |  |
|  |  | ZBTB46 |  |
|  |  | SMOC2 |  |
|  |  | SCN4B |  |
|  |  | SNAP25 |  |
|  |  | SGK494 |  |
|  |  | SNAP47 |  |
|  |  | SNAP91 |  |
|  |  | SGPL1 |  |
|  |  | SNRPB2 |  |
|  |  | SH3BP5 |  |
|  |  | ZCCHC4 |  |
|  |  | ZCCHC7 |  |
|  |  | ZDHHC18 |  |
|  |  | ZDHHC22 |  |
|  |  | SNX6 |  |
|  |  | ZER1 |  |
|  |  | SOCS5 |  |
|  |  | ZFP30 |  |
|  |  | ZFP36 |  |
|  |  | ZFP64 |  |
|  |  | ZFP69B |  |
|  |  | SORT1 |  |
|  |  | SOS2 |  |
|  |  | SOST |  |
|  |  | SOSTDC1 |  |
|  |  | ZFYVE27 |  |
|  |  | SOX1 |  |
|  |  | ZHX2 |  |
|  |  | ZIC4 |  |
|  |  | SOX3 |  |
|  |  | SOX4 |  |
|  |  | SERPINB4 |  |
|  |  | ZMAT4 |  |
|  |  | SERPINC1 |  |
|  |  | SERPINI1 |  |
|  |  | SPAG1 |  |
|  |  | SPARC |  |
|  |  | ZNF117 |  |
|  |  | ZNF134 |  |
|  |  | ZNF157 |  |
|  |  | SLC16A10 |  |
|  |  | SPIN4 |  |
|  |  | ZNF202 |  |
|  |  | LSM11 |  |
|  |  | LTB |  |
|  |  | LTB4R2 |  |
|  |  | LTV1 |  |
|  |  | LUC7L |  |
|  |  | LUC7L2 |  |
|  |  | LUZP6 |  |
|  |  | LY6E |  |
|  |  | LYRM5 |  |
|  |  | LYSMD2 |  |
|  |  | LYST |  |
|  |  | SRP54 |  |
|  |  | MACROD2 |  |
|  |  | MAF |  |
|  |  | SRRM4 |  |
|  |  | MAGEE1 |  |
|  |  | ZNF365 |  |
|  |  | MAGOH |  |
|  |  | SRSF3 |  |
|  |  | SRSF9 |  |
|  |  | MAMDC4 |  |
|  |  | ZNF410 |  |
|  |  | SSR3 |  |
|  |  | MAP1B |  |
|  |  | MAP2 |  |
|  |  | MAP2K7 |  |
|  |  | MAP3K1 |  |
|  |  | SLC39A10 |  |
|  |  | SLC39A6 |  |
|  |  | ZNF510 |  |
|  |  | SLAMF8 |  |
|  |  | ZNF512B |  |
|  |  | SLC16A2 |  |
|  |  | VHLL |  |
|  |  | MARVELD1 |  |
|  |  | MARVELD2 |  |
|  |  | MATN1 |  |
|  |  | STRADA |  |
|  |  | STRADB |  |
|  |  | ZNF596 |  |
|  |  | ZNF597 |  |
|  |  | ZNF600 |  |
|  |  | MBLAC2 |  |
|  |  | MBOAT2 |  |
|  |  | STX18 |  |
|  |  | VSX2 |  |
|  |  | MCF2L2 |  |
|  |  | SMAP1 |  |
|  |  | SMC1A |  |
|  |  | SMC1B |  |
|  |  | KIF21B |  |
|  |  | SMC5 |  |
|  |  | SMC6 |  |
|  |  | SMCHD1 |  |
|  |  | SMEK2 |  |
|  |  | KIF3C |  |
|  |  | SLC30A10 |  |
|  |  | SLC30A5 |  |
|  |  | SLC34A2 |  |
|  |  | SLC35A3 |  |
|  |  | SYBU |  |
|  |  | SYCP2 |  |
|  |  | SYDE2 |  |
|  |  | SLC36A4 |  |
|  |  | SYNE1 |  |
|  |  | SNAPC1 |  |
|  |  | SNAPC3 |  |
|  |  | SLC39A1 |  |
|  |  | WIPI1 |  |
|  |  | SLC39A2 |  |
|  |  | SLC39A9 |  |
|  |  | SLC40A1 |  |
|  |  | ZNF419 |  |
|  |  | WNT3 |  |
|  |  | SLC44A1 |  |
|  |  | SYT9 |  |
|  |  | TRAF1 |  |
|  |  | SLC4A4 |  |
|  |  | SLC4A5 |  |
|  |  | TRAF4 |  |
|  |  | SLC6A12 |  |
|  |  | TRAPPC12 |  |
|  |  | TAF15 |  |
|  |  | TAF2 |  |
|  |  | SORL1 |  |
|  |  | ZNF502 |  |
|  |  | TRIM15 |  |
|  |  | TRIM16L |  |
|  |  | TAOK3 |  |
|  |  | TARBP1 |  |
|  |  | TAS2R13 |  |
|  |  | TAT |  |
|  |  | TATDN1 |  |
|  |  | TATDN2 |  |
|  |  | TBC1D10B |  |
|  |  | TBC1D15 |  |
|  |  | TBC1D23 |  |
|  |  | TBC1D2B |  |
|  |  | SERPINB8 |  |
|  |  | SERTAD2 |  |
|  |  | SESN3 |  |
|  |  | SESTD1 |  |
|  |  | SETBP1 |  |
|  |  | SETD3 |  |
|  |  | SETD5 |  |
|  |  | SETD7 |  |
|  |  | TBXA2R |  |
|  |  | SCFD1 |  |
|  |  | SF3A3 |  |
|  |  | SF3B5 |  |
|  |  | SFMBT1 |  |
|  |  | SFMBT2 |  |
|  |  | SFPQ |  |
|  |  | SCN4A |  |
|  |  | SLC25A45 |  |
|  |  | SGK3 |  |
|  |  | SCNN1A |  |
|  |  | SGMS2 |  |
|  |  | ZNF311 |  |
|  |  | SGPP1 |  |
|  |  | SGPP2 |  |
|  |  | SGSM2 |  |
|  |  | SGSM3 |  |
|  |  | SGTA |  |
|  |  | TTBK1 |  |
|  |  | ZNF333 |  |
|  |  | TTC29 |  |
|  |  | SH3BP2 |  |
|  |  | SH3BP5L |  |
|  |  | SH3D19 |  |
|  |  | SH3GL3 |  |
|  |  | SH3GLB1 |  |
|  |  | SH3KBP1 |  |
|  |  | SH3PXD2A |  |
|  |  | SH3PXD2B |  |
|  |  | SH3RF1 |  |
|  |  | SH3RF2 |  |
|  |  | SH3TC2 |  |
|  |  | SHANK2 |  |
|  |  | SHC3 |  |
|  |  | SHC4 |  |
|  |  | TUBGCP3 |  |
|  |  | SHISA3 |  |
|  |  | SLC35F1 |  |
|  |  | SHOC2 |  |
|  |  | SHOX |  |
|  |  | SHOX2 |  |
|  |  | SHPRH |  |
|  |  | SIAE |  |
|  |  | SIGLEC5 |  |
|  |  | SIGLEC9 |  |
|  |  | SIK2 |  |
|  |  | SIM1 |  |
|  |  | SIMC1 |  |
|  |  | SKI |  |
|  |  | ZNF500 |  |
|  |  | ZNF514 |  |
|  |  | UBASH3B |  |
|  |  | SP3 |  |
|  |  | SP4 |  |
|  |  | SERTAD4 |  |
|  |  | SLC12A7 |  |
|  |  | SLC13A4 |  |
|  |  | SLC13A5 |  |
|  |  | SLC15A4 |  |
|  |  | SETX |  |
|  |  | SLC16A12 |  |
|  |  | SLC16A14 |  |
|  |  | SLC16A7 |  |
|  |  | SLC17A5 |  |
|  |  | SLC22A1 |  |
|  |  | SLC22A10 |  |
|  |  | SLC24A1 |  |
|  |  | SLC25A16 |  |
|  |  | SLC25A27 |  |
|  |  | SLC25A30 |  |
|  |  | SH2D3C |  |
|  |  | SH3BGRL |  |
|  |  | RGS10 |  |
|  |  | SLC2A8 |  |
|  |  | RGS4 |  |
|  |  | RGS6 |  |
|  |  | RHOH |  |
|  |  | RHOJ |  |
|  |  | SLC35B2 |  |
|  |  | SIGLECL1 |  |
|  |  | SIGMAR1 |  |
|  |  | SLC35E3 |  |
|  |  | SOBP |  |
|  |  | SLC35F6 |  |
|  |  | SLC35G1 |  |
|  |  | SLC36A1 |  |
|  |  | SLC37A2 |  |
|  |  | SLC4A10 |  |
|  |  | SLC38A2 |  |
|  |  | SLC38A9 |  |
|  |  | SKA3 |  |
|  |  | SLC44A5 |  |
|  |  | SLC45A3 |  |
|  |  | ZNF490 |  |
|  |  | VANGL2 |  |
|  |  | SLAMF7 |  |
|  |  | VASH1 |  |
|  |  | VDAC3 |  |
|  |  | VDR |  |
|  |  | STK32B |  |
|  |  | VIPAS39 |  |
|  |  | VPS25 |  |
|  |  | VRK1 |  |
|  |  | VRK2 |  |
|  |  | VSTM2A |  |
|  |  | SBK1 |  |
|  |  | SMAD3 |  |
|  |  | VWA8 |  |
|  |  | SMAD9 |  |
|  |  | SMARCA1 |  |
|  |  | WAPAL |  |
|  |  | SMARCD1 |  |
|  |  | SMARCE1 |  |
|  |  | WBP4 |  |
|  |  | ZNF283 |  |
|  |  | ZNF284 |  |
|  |  | SLC30A4 |  |
|  |  | WDR20 |  |
|  |  | WDR26 |  |
|  |  | KIF5C |  |
|  |  | WDR44 |  |
|  |  | SMN2 |  |
|  |  | SMNDC1 |  |
|  |  | SMOC1 |  |
|  |  | SLC35F2 |  |
|  |  | SLC37A3 |  |
|  |  | SLC38A4 |  |
|  |  | SLC38A7 |  |
|  |  | WIPF2 |  |
|  |  | WIPF3 |  |
|  |  | SNED1 |  |
|  |  | SNIP1 |  |
|  |  | SNRNP27 |  |
|  |  | SST |  |
|  |  | ZNF426 |  |
|  |  | SLC45A4 |  |
|  |  | SOGA2 |  |
|  |  | SNX14 |  |
|  |  | SLC5A1 |  |
|  |  | SLC5A7 |  |
|  |  | TRAFD1 |  |
|  |  | WWOX |  |
|  |  | SLC6A17 |  |
|  |  | TRAPPC2 |  |
|  |  | SORCS3 |  |
|  |  | ZSCAN12 |  |
|  |  | ZSCAN20 |  |
|  |  | SOX11 |  |
|  |  | SLCO4C1 |  |
|  |  | SLITRK2 |  |
|  |  | TRIM36 |  |
|  |  | SLITRK5 |  |
|  |  | TRIM7 |  |
|  |  | SAMD5 |  |
|  |  | SAMHD1 |  |
|  |  | SAMSN1 |  |
|  |  | SARDH |  |
|  |  | UBTD1 |  |
|  |  | SMC3 |  |
|  |  | SMCR8 |  |
|  |  | TRPM8 |  |
|  |  | ZNF256 |  |
|  |  | ZNF259 |  |
|  |  | SMKR1 |  |
|  |  | SCN10A |  |
|  |  | SCN1B |  |
|  |  | SH2D1A |  |
|  |  | SCN2B |  |
|  |  | SH3BGRL3 |  |
|  |  | ZNF286B |  |
|  |  | ZNF302 |  |
|  |  | ZNF330 |  |
|  |  | SNRNP40 |  |
|  |  | ZNF33B |  |
|  |  | SNRPD1 |  |
|  |  | ZNF35 |  |
|  |  | ZNF354C |  |
|  |  | TTLL5 |  |
|  |  | ZNF41 |  |
|  |  | ZNF398 |  |
|  |  | ZNF415 |  |
|  |  | TWSG1 |  |
|  |  | SORBS1 |  |
|  |  | TXNDC12 |  |
|  |  | TXNDC17 |  |
|  |  | TXNDC5 |  |
|  |  | TXNL4B |  |
|  |  | TXNRD1 |  |
|  |  | SOX17 |  |
|  |  | SOX21 |  |
|  |  | ZNF493 |  |
|  |  | SORCS1 |  |
|  |  | ZNF518B |  |
|  |  | ZNF519 |  |
|  |  | SP140L |  |
|  |  | SP2 |  |
|  |  | UBD |  |
|  |  | ZNF532 |  |
|  |  | SESN1 |  |
|  |  | SETD8 |  |
|  |  | UBE2QL1 |  |
|  |  | ZNF562 |  |
|  |  | UBE3B |  |
|  |  | UBE3C |  |
|  |  | SFI1 |  |
|  |  | UBQLN2 |  |
|  |  | ZNF585B |  |
|  |  | UBR5 |  |
|  |  | UBTD2 |  |
|  |  | UBXN2B |  |
|  |  | SPPL3 |  |
|  |  | USP12 |  |
|  |  | SGOL1 |  |
|  |  | SPTLC2 |  |
|  |  | KIF2B |  |
|  |  | SH3GL2 |  |
|  |  | KIRREL |  |
|  |  | SH3RF3 |  |
|  |  | SHC1 |  |
|  |  | SHCBP1 |  |
|  |  | SHE |  |
|  |  | SHH |  |
|  |  | SHROOM2 |  |
|  |  | SI |  |
|  |  | ZNF385B |  |
|  |  | SOAT1 |  |
|  |  | ZNF417 |  |
|  |  | ZNF429 |  |
|  |  | SNX13 |  |
|  |  | ZNF878 |  |
|  |  | ZPLD1 |  |
|  |  | ZNF496 |  |
|  |  | SMG7 |  |
|  |  | ZNF525 |  |
|  |  | ZNF527 |  |
|  |  | STK32A |  |
|  |  | VGLL4 |  |
|  |  | STMN2 |  |
|  |  | ZNF584 |  |
|  |  | ZNF589 |  |
|  |  | ZNF592 |  |
|  |  | ZNF594 |  |
|  |  | ZNF606 |  |
|  |  | ZNF608 |  |
|  |  | STX12 |  |
|  |  | TRPC5 |  |
|  |  | WDR92 |  |
|  |  | VWA9 |  |
|  |  | VWC2L |  |
|  |  | KIF13B |  |
|  |  | WASF3 |  |
|  |  | SUCLG1 |  |
|  |  | SMOX |  |
|  |  | ZNF285 |  |
|  |  | ZNF286A |  |
|  |  | WDR17 |  |
|  |  | ZNF292 |  |
|  |  | SRP14 |  |
|  |  | ZNF3 |  |
|  |  | WDR43 |  |
|  |  | SLC35D2 |  |
|  |  | WDR73 |  |
|  |  | SPP1 |  |
|  |  | SNX2 |  |
|  |  | ZNF404 |  |
|  |  | WIZ |  |
|  |  | ZNF425 |  |
|  |  | SSTR1 |  |
|  |  | USP40 |  |
|  |  | SOD2 |  |
|  |  | SON |  |
|  |  | WSCD1 |  |
|  |  | ZNF470 |  |
|  |  | WWP1 |  |
|  |  | SLC7A1 |  |
|  |  | SOGA3 |  |
|  |  | ZNF350 |  |
|  |  | ZNF354B |  |
|  |  | SNX1 |  |
|  |  | SOWAHA |  |
|  |  | SOWAHC |  |
|  |  | SLCO2B1 |  |
|  |  | SOX30 |  |
|  |  | SLFN5 |  |
|  |  | SOX7 |  |
|  |  | TRIM72 |  |
|  |  | SMAD5 |  |
|  |  | SMARCAD1 |  |
|  |  | SMARCC1 |  |
|  |  | SPOCK3 |  |
|  |  | ZNF491 |  |
|  |  | ZNF358 |  |
|  |  | SPTB |  |
|  |  | ZNF264 |  |
|  |  | ZNF268 |  |
|  |  | SPTY2D1 |  |
|  |  | SRCAP |  |
|  |  | ZNF280C |  |
|  |  | ZNF281 |  |
|  |  | ZNF287 |  |
|  |  | SRM |  |
|  |  | SLC26A11 |  |
|  |  | SLC26A4 |  |
|  |  | SNF8 |  |
|  |  | ZNF326 |  |
|  |  | ZNF347 |  |
|  |  | ZNF34 |  |
|  |  | SNRPG |  |
|  |  | ZNF224 |  |
|  |  | ZNF225 |  |
|  |  | USH1C |  |
|  |  | SRSF2 |  |
|  |  | ZNF382 |  |
|  |  | USP28 |  |
|  |  | SNX30 |  |
|  |  | SNX4 |  |
|  |  | SOCS6 |  |
|  |  | SOCS7 |  |
|  |  | ST20-MTHFS |  |
|  |  | ZNF439 |  |
|  |  | SORBS2 |  |
|  |  | ZNF90 |  |
|  |  | ZNF471 |  |
|  |  | SOWAHB |  |
|  |  | ZNF483 |  |
|  |  | SOX13 |  |
|  |  | ZNF383 |  |
|  |  | ZSCAN25 |  |
|  |  | ZNF543 |  |
|  |  | ZNF544 |  |
|  |  | SPAG16 |  |
|  |  | SPAG9 |  |
|  |  | UGT8 |  |
|  |  | ZNF557 |  |
|  |  | ZNF566 |  |
|  |  | ZNF568 |  |
|  |  | SPHKAP |  |
|  |  | ZNF583 |  |
|  |  | ZNF585A |  |
|  |  | ZNF605 |  |
|  |  | ZNF384 |  |
|  |  | UCP2 |  |
|  |  | SPTBN1 |  |
|  |  | ZNF257 |  |
|  |  | SPTSSB |  |
|  |  | ZNF274 |  |
|  |  | ZNF783 |  |
|  |  | UNC13C |  |
|  |  | SLC27A4 |  |
|  |  | KLF10 |  |
|  |  | WDR45B |  |
|  |  | SRRT |  |
|  |  | URB1 |  |
|  |  | SS18L1 |  |
|  |  | ZNF37A |  |
|  |  | ZNF397 |  |
|  |  | ZNF548 |  |
|  |  | ZNF556 |  |
|  |  | KIAA1407 |  |
|  |  | KIAA1429 |  |
|  |  | ZNF579 |  |
|  |  | UST |  |
|  |  | VAMP1 |  |
|  |  | ZNF488 |  |
|  |  | KLF13 |  |
|  |  | ZNF345 |  |
|  |  | USO1 |  |
|  |  | ZXDB |  |
|  |  | UGT2B28 |  |
|  |  | ZNF277 |  |
|  |  | ZNF280D |  |
|  |  | KIF4A |  |
|  |  | UNC5B |  |
|  |  | SERPINA5 |  |
|  |  | SERPINA7 |  |
|  |  | SERPINB3 |  |
|  |  | ZNF587 |  |
|  |  | ZNF485 |  |
|  |  | ZPBP2 |  |
|  |  | TRRAP |  |
|  |  | ZNF250 |  |
|  |  | UFM1 |  |
|  |  | ZNF77 |  |
|  |  | ZNF536 |  |
|  |  | UBE2E1 |  |
|  |  | ZNF649 |  |
|  |  | SP6 |  |
|  |  | ZNF282 |  |
|  |  | ZNF662 |  |
|  |  | SLC29A2 |  |
|  |  | SLC2A4 |  |
|  |  | ZNF705B |  |
|  |  | ZNF711 |  |
|  |  | ZNF705G |  |
|  |  | ZNF235 |  |
|  |  | USP25 |  |
|  |  | SOX12 |  |
|  |  | ZNF391 |  |
|  |  | ZNF260 |  |
|  |  | ZNF541 |  |
|  |  | ZNF26 |  |
|  |  | SLC25A48 |  |
|  |  | SPACA4 |  |
|  |  | ZNF879 |  |
|  |  | UBQLN1 |  |
|  |  | ZNF248 |  |
|  |  | URB2 |  |
|  |  | ZNF709 |  |
|  |  | SLC30A8 |  |
|  |  | ZNF253 |  |
|  |  | ZNF24 |  |
|  |  | ZNF658 |  |
|  |  | ZNF546 |  |
|  |  | ZNF266 |  |
|  |  | ZYG11B |  |
|  |  | ZNF558 |  |
|  |  | ZNF550 |  |
|  |  | ZNF570 |  |
|  |  | ZNRD1 |  |
|  |  | ZNF567 |  |
|  |  | ZNF569 |  |
|  |  | ZNF322 |  |
|  |  | TRMT12 |  |
|  |  | TRMT61A |  |
|  |  | ZNF480 |  |
|  |  | ZNF619 |  |
|  |  | ZNF227 |  |
|  |  | SLC31A1 |  |
|  |  | ZNF521 |  |
|  |  | ZNF239 |  |
|  |  | ZNF572 |  |
|  |  | ZNF578 |  |
|  |  | ZNF25 |  |
|  |  | ZNF624 |  |
|  |  | UBE2E3 |  |
|  |  | ZNF555 |  |
|  |  | ZNF547 |  |
|  |  | KIAA1147 |  |
|  |  | ZNF549 |  |
|  |  | ZNF551 |  |
|  |  | ZNF660 |  |
|  |  | ZNF341 |  |
|  |  | UPF1 |  |
|  |  | UNC93B1 |  |
|  |  | UXS1 |  |
|  |  | ZNF695 |  |
|  |  | ZNF234 |  |
|  |  | ZNF236 |  |
|  |  | ZNF616 |  |
|  |  | ZNF684 |  |
|  |  | ZNF48 |  |
|  |  | UPK1B |  |
|  |  | ZNF681 |  |
|  |  | ZNF554 |  |
|  |  | ZNF713 |  |
|  |  | ZNF230 |  |
|  |  | ZNF705D |  |
|  |  | ZNF667 |  |
|  |  | MAPK10 |  |
|  |  | LAMC2 |  |
|  |  | GNAO1 |  |
|  |  | C6orf132 |  |
|  |  | CALB1 |  |
|  |  | KDM5C |  |
|  |  | ARHGAP39 |  |
|  |  | ARNT |  |
|  |  | TRIB2 |  |
|  |  | ZFYVE26 |  |
|  |  | LRRC8A |  |
|  |  | TCEA2 |  |
|  |  | ARHGEF15 |  |
|  |  | FHDC1 |  |
|  |  | ADCY9 |  |
|  |  | GDF11 |  |
|  |  | MAP3K3 |  |
|  |  | DUSP7 |  |
|  |  | GSG1L |  |
|  |  | CBX7 |  |
|  |  | AJUBA |  |
|  |  | IL17RD |  |
|  |  | ING5 |  |
|  |  | AL626787.1 |  |
|  |  | IGSF3 |  |
|  |  | AP2M1 |  |
|  |  | TSPAN31 |  |
|  |  | LRP4 |  |
|  |  | ZHX3 |  |
|  |  | POLR2J2 |  |
|  |  | SPECC1L |  |
|  |  | HNRNPUL2 |  |
|  |  | HYI |  |
|  |  | MRPL43 |  |
|  |  | ACAP3 |  |
|  |  | ALKBH5 |  |
|  |  | ANAPC15 |  |
|  |  | ANKRD13A |  |
|  |  | NPAS4 |  |
|  |  | CTB-54O9.9 |  |
|  |  | SLC16A6 |  |
|  |  | TPM2 |  |
|  |  | CCND1 |  |
|  |  | FAIM2 |  |
|  |  | NFATC1 |  |
|  |  | DNAJB1 |  |
|  |  | DNAJC13 |  |
|  |  | LPAR3 |  |
|  |  | PLAU |  |
|  |  | AL590483.1 |  |
|  |  | PPP2R5C |  |
|  |  | DBN1 |  |
|  |  | SLC39A5 |  |
|  |  | PCDH1 |  |
|  |  | SNX22 |  |
|  |  | LOXL4 |  |
|  |  | SNX27 |  |
|  |  | GRB7 |  |
|  |  | HIC2 |  |
|  |  | CLCN5 |  |
|  |  | PTPRF |  |
|  |  | XXbac-BPG32J3.20 | |
|  |  | RNF222 |  |
|  |  | EPHA10 |  |
|  |  | ABHD17A |  |
|  |  | CNBP |  |
|  |  | EIF4EBP1 |  |
|  |  | BPGM |  |
|  |  | ATOX1 |  |
|  |  | EIF4ENIF1 |  |
|  |  | ABCG8 |  |
|  |  | FAM127A |  |
|  |  | ANXA7 |  |
|  |  | BRPF3 |  |
|  |  | ANKRD36 |  |
|  |  | AP001652.1 |  |
|  |  | FAM13C |  |
|  |  | TOB1 |  |
|  |  | FAM127C |  |
|  |  | AP000889.3 |  |
|  |  | ABCB5 |  |
|  |  | FP15737 |  |
|  |  | ACKR4 |  |
|  |  | ABT1 |  |
|  |  | AC007040.11 |  |
|  |  | PCDHGC3 |  |
|  |  | FAM160A1 |  |
|  |  | DPPA3 |  |
|  |  | AC022532.1 |  |
|  |  | AP000322.54 |  |
|  |  | TOX2 |  |
|  |  | AP3B2 |  |
|  |  | ACSL5 |  |
|  |  | FAM117A |  |
|  |  | FGFR1 |  |
|  |  | YBX3 |  |
|  |  | APOC3 |  |
|  |  | EGF |  |
|  |  | ZBTB14 |  |
|  |  | TRIM60 |  |
|  |  | OGN |  |
|  |  | FCGR1B |  |
|  |  | ABCD3 |  |
|  |  | EDIL3 |  |
|  |  | C12orf65 |  |
|  |  | WDR31 |  |
|  |  | ZNF454 |  |
|  |  | WDR45 |  |
|  |  | FITM2 |  |
|  |  | AKAP6 |  |
|  |  | PGBD2 |  |
|  |  | ARHGAP40 |  |
|  |  | ABCB11 |  |
|  |  | AMDHD1 |  |
|  |  | FAM101B |  |
|  |  | DNAJC24 |  |
|  |  | CLU |  |
|  |  | ME2 |  |
|  |  | FAM133B |  |
|  |  | PCDHGC4 |  |
|  |  | EGLN3 |  |
|  |  | FURIN |  |
|  |  | AL583828.1 |  |
|  |  | FBXO41 |  |
|  |  | FOXK2 |  |
|  |  | AAED1 |  |
|  |  | AURKC |  |
|  |  | ELL3 |  |
|  |  | DDX51 |  |
|  |  | DEFB132 |  |
|  |  | ZNF724P |  |
|  |  | BBOX1 |  |
|  |  | ARC |  |
|  |  | ARFGEF1 |  |
|  |  | AHCYL1 |  |
|  |  | BIVM |  |
|  |  | FGF1 |  |
|  |  | FGF4 |  |
|  |  | PIGG |  |
|  |  | DNTTIP2 |  |
|  |  | CPLX2 |  |
|  |  | ATP6V1C2 |  |
|  |  | WNT9B |  |
|  |  | DTD2 |  |
|  |  | DTHD1 |  |
|  |  | XAGE3 |  |
|  |  | AADAC |  |
|  |  | DUSP9 |  |
|  |  | FOXL1 |  |
|  |  | AHR |  |
|  |  | CLSPN |  |
|  |  | C12orf71 |  |
|  |  | ABHD15 |  |
|  |  | AC008132.1 |  |
|  |  | AC012123.1 |  |
|  |  | AC011294.3 |  |
|  |  | DCAF17 |  |
|  |  | ACTL6A |  |
|  |  | ADAM10 |  |
|  |  | ADI1 |  |
|  |  | BCAR3 |  |
|  |  | ADPGK |  |
|  |  | CPAMD8 |  |
|  |  | FBXO8 |  |
|  |  | FCGR1A |  |
|  |  | RS1 |  |
|  |  | ZNF154 |  |
|  |  | AIG1 |  |
|  |  | AIM1 |  |
|  |  | PIGO |  |
|  |  | AL033381.1 |  |
|  |  | AL354808.2 |  |
|  |  | AF196779.12 |  |
|  |  | ALPK1 |  |
|  |  | XDH |  |
|  |  | AMIGO1 |  |
|  |  | CYP4F3 |  |
|  |  | ECE2 |  |
|  |  | TMEM87A |  |
|  |  | ANKMY1 |  |
|  |  | ANKRD10 |  |
|  |  | AC027763.2 |  |
|  |  | ZBTB26 |  |
|  |  | ZNF573 |  |
|  |  | ZNF613 |  |
|  |  | ZNF615 |  |
|  |  | EIF4H |  |
|  |  | RAB33B |  |
|  |  | APOBEC3A |  |
|  |  | TRIAP1 |  |
|  |  | OTC |  |
|  |  | ENOPH1 |  |
|  |  | BCAS2 |  |
|  |  | BCAS4 |  |
|  |  | RANBP3 |  |
|  |  | AC093677.1 |  |
|  |  | EPGN |  |
|  |  | ARGFX |  |
|  |  | FCF1 |  |
|  |  | ZNF138 |  |
|  |  | ARL6 |  |
|  |  | SAMD9L |  |
|  |  | DPP9 |  |
|  |  | ARSE |  |
|  |  | RERG |  |
|  |  | ALOX5AP |  |
|  |  | AMACR |  |
|  |  | PCDHGA6 |  |
|  |  | FAM120C |  |
|  |  | PCDHGB1 |  |
|  |  | PCDHGB2 |  |
|  |  | PCDHGB3 |  |
|  |  | PCDHGB4 |  |
|  |  | PCDHGB6 |  |
|  |  | PCDHGB7 |  |
|  |  | FAM186B |  |
|  |  | ATP6V1G3 |  |
|  |  | PCYT1B |  |
|  |  | FAM208B |  |
|  |  | COL23A1 |  |
|  |  | ZNF648 |  |
|  |  | RAB6B |  |
|  |  | B3GNT6 |  |
|  |  | BAZ1B |  |
|  |  | COX16 |  |
|  |  | AQP10 |  |
|  |  | AQP9 |  |
|  |  | PEX2 |  |
|  |  | FBXO45 |  |
|  |  | FEN1 |  |
|  |  | AK7 |  |
|  |  | WDR59 |  |
|  |  | WDR65 |  |
|  |  | SBDS |  |
|  |  | BST1 |  |
|  |  | SCD |  |
|  |  | BTG1 |  |
|  |  | FNDC3B |  |
|  |  | FNDC5 |  |
|  |  | FNIP2 |  |
|  |  | FOXD1 |  |
|  |  | PLEKHG7 |  |
|  |  | YOD1 |  |
|  |  | PMP22 |  |
|  |  | ZBED5 |  |
|  |  | ZBTB1 |  |
|  |  | FSD2 |  |
|  |  | DCAF6 |  |
|  |  | FAM180A |  |
|  |  | DCLRE1C |  |
|  |  | DCPS |  |
|  |  | ZC3H10 |  |
|  |  | DDX19A |  |
|  |  | DDX19B |  |
|  |  | DDX28 |  |
|  |  | DDX50 |  |
|  |  | DDX59 |  |
|  |  | ADH1B |  |
|  |  | DIEXF |  |
|  |  | ZKSCAN4 |  |
|  |  | AGBL1 |  |
|  |  | NIPAL2 |  |
|  |  | NKIRAS2 |  |
|  |  | BOD1 |  |
|  |  | FLJ45079 |  |
|  |  | DRAM1 |  |
|  |  | TMEM241 |  |
|  |  | DYNC1I1 |  |
|  |  | TMEM59 |  |
|  |  | ZNF449 |  |
|  |  | ZNF484 |  |
|  |  | PTRHD1 |  |
|  |  | TNFRSF19 |  |
|  |  | TNFSF10 |  |
|  |  | TNFSF14 |  |
|  |  | EHHADH |  |
|  |  | EIF1AX |  |
|  |  | EIF2S3 |  |
|  |  | EIF5AL1 |  |
|  |  | TPD52L3 |  |
|  |  | EMILIN2 |  |
|  |  | EMR2 |  |
|  |  | ZNF738 |  |
|  |  | ENOSF1 |  |
|  |  | ZNF850 |  |
|  |  | RAX2 |  |
|  |  | ARHGAP6 |  |
|  |  | RBM17 |  |
|  |  | RBM38 |  |
|  |  | NOA1 |  |
|  |  | ESR2 |  |
|  |  | ZNF254 |  |
|  |  | ARPC3 |  |
|  |  | EVI2B |  |
|  |  | EXO1 |  |
|  |  | TXNDC2 |  |
|  |  | TYW1B |  |
|  |  | F5 |  |
|  |  | FABP7 |  |
|  |  | FAHD1 |  |
|  |  | PCDHGA11 |  |
|  |  | ATG10 |  |
|  |  | ATG14 |  |
|  |  | ATG3 |  |
|  |  | PCDHGC5 |  |
|  |  | EID2B |  |
|  |  | FAM193A |  |
|  |  | FAM196B |  |
|  |  | RNF216 |  |
|  |  | COBL |  |
|  |  | B2M |  |
|  |  | B3GNT2 |  |
|  |  | RP11-276H1.3 |  |
|  |  | RP11-362K2.2 |  |
|  |  | RP11-47I22.4 |  |
|  |  | RP11-542P2.1 |  |
|  |  | RP11-644F5.10 |  |
|  |  | RP11-817J15.3 |  |
|  |  | RPA1 |  |
|  |  | RPL14 |  |
|  |  | RPL15 |  |
|  |  | RPL9 |  |
|  |  | CSMD1 |  |
|  |  | PIGR |  |
|  |  | PIGV |  |
|  |  | WDR5B |  |
|  |  | WDR88 |  |
|  |  | FLT1 |  |
|  |  | C10orf111 |  |
|  |  | C10orf128 |  |
|  |  | C10orf137 |  |
|  |  | C10orf32 |  |
|  |  | C12orf40 |  |
|  |  | PLXNA4 |  |
|  |  | PMEPA1 |  |
|  |  | POLD3 |  |
|  |  | POLR2F |  |
|  |  | C1GALT1C1 |  |
|  |  | ZBTB9 |  |
|  |  | GAA |  |
|  |  | ZC3HAV1 |  |
|  |  | ZCCHC8 |  |
|  |  | ZDHHC24 |  |
|  |  | ZEB2 |  |
|  |  | DIABLO |  |
|  |  | ZFPM2 |  |
|  |  | DKFZP434H0512 |  |
|  |  | DKK2 |  |
|  |  | DLK2 |  |
|  |  | PRKRIR |  |
|  |  | SLC10A1 |  |
|  |  | ZNF185 |  |
|  |  | ZNF195 |  |
|  |  | ZNF232 |  |
|  |  | LAMB3 |  |
|  |  | PSTPIP1 |  |
|  |  | ZNF331 |  |
|  |  | CC2D1B |  |
|  |  | ZNF396 |  |
|  |  | ZNF420 |  |
|  |  | CCDC121 |  |
|  |  | ZNF43 |  |
|  |  | ZNF430 |  |
|  |  | LINC00346 |  |
|  |  | CCDC62 |  |
|  |  | ZNF479 |  |
|  |  | ZNF492 |  |
|  |  | ZNF552 |  |
|  |  | ZNF559 |  |
|  |  | ZNF560 |  |
|  |  | RAB11B |  |
|  |  | CD2BP2 |  |
|  |  | GRK1 |  |
|  |  | ZNF639 |  |
|  |  | GRWD1 |  |
|  |  | ZNF655 |  |
|  |  | ZNF674 |  |
|  |  | ZNF675 |  |
|  |  | ZNF677 |  |
|  |  | CDR1 |  |
|  |  | CEP104 |  |
|  |  | ZNF91 |  |
|  |  | ZNF92 |  |
|  |  | RBBP9 |  |
|  |  | ZNRF3 |  |
|  |  | ZSWIM1 |  |
|  |  | ZW10 |  |
|  |  | ARMC4 |  |
|  |  | ARMC9 |  |
|  |  | ARPP21 |  |
|  |  | ARX |  |
|  |  | CLCA4 |  |
|  |  | ASB9 |  |
|  |  | HPS6 |  |
|  |  | CLNS1A |  |
|  |  | MDM1 |  |
|  |  | SPRED2 |  |
|  |  | MECOM |  |
|  |  | ATP1B3 |  |
|  |  | ATP5J2-PTCD1 |  |
|  |  | ATP6V0A2 |  |
|  |  | IFT52 |  |
|  |  | RP11-1055B8.7 |  |
|  |  | RP11-169F17.1 |  |
|  |  | COLCA1 |  |
|  |  | COPS2 |  |
|  |  | COX15 |  |
|  |  | MLLT1 |  |
|  |  | MLLT10 |  |
|  |  | CPVL |  |
|  |  | CRB3 |  |
|  |  | CRCP |  |
|  |  | CRISP3 |  |
|  |  | CSGALNACT1 |  |
|  |  | RWDD3 |  |
|  |  | SAMD4B |  |
|  |  | CT45A3 |  |
|  |  | CT45A4 |  |
|  |  | ITGB6 |  |
|  |  | CWH43 |  |
|  |  | CYP4F11 |  |
|  |  | MYO18A |  |
|  |  | CYTL1 |  |
|  |  | CYYR1 |  |
|  |  | DAB2IP |  |
|  |  | DAP3 |  |
|  |  | DAZL |  |
|  |  | DBT |  |
|  |  | DCAF12L1 |  |
|  |  | DCAF13 |  |
|  |  | TEX15 |  |
|  |  | THAP8 |  |
|  |  | THBS4 |  |
|  |  | GALNTL5 |  |
|  |  | ZFAT |  |
|  |  | ZFP28 |  |
|  |  | TIGD2 |  |
|  |  | TIGD7 |  |
|  |  | TIMP2 |  |
|  |  | PRDM9 |  |
|  |  | TMEM106A |  |
|  |  | TMEM107 |  |
|  |  | TMEM110 |  |
|  |  | TMEM123 |  |
|  |  | NLRC5 |  |
|  |  | TMEM141 |  |
|  |  | NOC3L |  |
|  |  | GLTSCR1L |  |
|  |  | TMEM186 |  |
|  |  | TMEM201 |  |
|  |  | TMEM215 |  |
|  |  | NPR1 |  |
|  |  | TMEM229A |  |
|  |  | TMEM230 |  |
|  |  | NR4A1 |  |
|  |  | TMEM239 |  |
|  |  | TMEM254 |  |
|  |  | TMEM39A |  |
|  |  | TMEM40 |  |
|  |  | TMEM78 |  |
|  |  | NTMT1 |  |
|  |  | TMIGD1 |  |
|  |  | TMPRSS11F |  |
|  |  | TMSB4X |  |
|  |  | TNFRSF12A |  |
|  |  | TNKS1BP1 |  |
|  |  | NUP85 |  |
|  |  | NXPE3 |  |
|  |  | LRRC32 |  |
|  |  | CD248 |  |
|  |  | TPD52L1 |  |
|  |  | OPHN1 |  |
|  |  | OR10A3 |  |
|  |  | OR10A6 |  |
|  |  | TPX2 |  |
|  |  | OR5B12 |  |
|  |  | TRIM29 |  |
|  |  | TRIM37 |  |
|  |  | TRIM39 |  |
|  |  | OXGR1 |  |
|  |  | TRIM61 |  |
|  |  | TRIM64B |  |
|  |  | TRIP13 |  |
|  |  | EPS15L1 |  |
|  |  | EPS8 |  |
|  |  | ERC2 |  |
|  |  | ERLIN1 |  |
|  |  | ESR1 |  |
|  |  | ETF1 |  |
|  |  | TYW1 |  |
|  |  | PCDHB6 |  |
|  |  | PCDHB7 |  |
|  |  | PCDHGA1 |  |
|  |  | PCDHGA10 |  |
|  |  | PCDHGA12 |  |
|  |  | PCDHGA2 |  |
|  |  | FAM105B |  |
|  |  | UBE2S |  |
|  |  | UBE4A |  |
|  |  | PCNXL4 |  |
|  |  | PCP4L1 |  |
|  |  | PCSK6 |  |
|  |  | PCSK7 |  |
|  |  | FAM218A |  |
|  |  | FAM221B |  |
|  |  | FAM32A |  |
|  |  | FAM92A1 |  |
|  |  | PDZRN3 |  |
|  |  | PER2 |  |
|  |  | PER3 |  |
|  |  | PEX10 |  |
|  |  | VCP |  |
|  |  | VIP |  |
|  |  | VPS33B |  |
|  |  | VPS37A |  |
|  |  | VPS39 |  |
|  |  | RRBP1 |  |
|  |  | RRP15 |  |
|  |  | PIGB |  |
|  |  | RXFP1 |  |
|  |  | PIK3C3 |  |
|  |  | PIP4K2B |  |
|  |  | PIP4K2C |  |
|  |  | PIRT |  |
|  |  | PITHD1 |  |
|  |  | WHSC1 |  |
|  |  | PITPNM3 |  |
|  |  | PIWIL1 |  |
|  |  | PIWIL3 |  |
|  |  | PLA2G2D |  |
|  |  | C10orf53 |  |
|  |  | PLS1 |  |
|  |  | FSIP1 |  |
|  |  | POLQ |  |
|  |  | FUT11 |  |
|  |  | FXYD5 |  |
|  |  | GADD45A |  |
|  |  | GALNS |  |
|  |  | GBP7 |  |
|  |  | TLR8 |  |
|  |  | TM2D3 |  |
|  |  | PRKAG1 |  |
|  |  | PRKAR2B |  |
|  |  | PRMT10 |  |
|  |  | PROZ |  |
|  |  | PRR18 |  |
|  |  | PRRT4 |  |
|  |  | PSIP1 |  |
|  |  | PSMB11 |  |
|  |  | PSMB2 |  |
|  |  | GNRHR |  |
|  |  | PTCD1 |  |
|  |  | PTGS1 |  |
|  |  | PTP4A2 |  |
|  |  | GPHN |  |
|  |  | PTPN7 |  |
|  |  | PTTG1IP |  |
|  |  | PYCRL |  |
|  |  | QSOX1 |  |
|  |  | R3HDM2 |  |
|  |  | GRAMD2 |  |
|  |  | CD200R1L |  |
|  |  | SLC51A |  |
|  |  | LRRC57 |  |
|  |  | CD300LB |  |
|  |  | CD80 |  |
|  |  | RAB7L1 |  |
|  |  | RABEPK |  |
|  |  | RAD50 |  |
|  |  | RANBP2 |  |
|  |  | H6PD |  |
|  |  | RARRES3 |  |
|  |  | RASA4 |  |
|  |  | HDLBP |  |
|  |  | HEATR3 |  |
|  |  | HECTD1 |  |
|  |  | RBMXL3 |  |
|  |  | RBPMS |  |
|  |  | RBPMS2 |  |
|  |  | REEP5 |  |
|  |  | RFC5 |  |
|  |  | RGL2 |  |
|  |  | HPDL |  |
|  |  | HSD11B1 |  |
|  |  | RHPN2 |  |
|  |  | RIC3 |  |
|  |  | RNF112 |  |
|  |  | IBA57 |  |
|  |  | IDO1 |  |
|  |  | SSC5D |  |
|  |  | IGF2BP2 |  |
|  |  | IGSF5 |  |
|  |  | MIER1 |  |
|  |  | IL22 |  |
|  |  | INADL |  |
|  |  | INHBE |  |
|  |  | INPP5B |  |
|  |  | RPS8 |  |
|  |  | MRPL45 |  |
|  |  | IRAK2 |  |
|  |  | MRPS28 |  |
|  |  | CST9 |  |
|  |  | CT45A1 |  |
|  |  | MSL1 |  |
|  |  | CTAGE1 |  |
|  |  | JAG1 |  |
|  |  | CTSB |  |
|  |  | MTUS1 |  |
|  |  | MYCT1 |  |
|  |  | C14orf1 |  |
|  |  | C14orf2 |  |
|  |  | C15orf32 |  |
|  |  | C17orf58 |  |
|  |  | KCNV1 |  |
|  |  | SEPHS2 |  |
|  |  | C19orf40 |  |
|  |  | C19orf66 |  |
|  |  | C1orf180 |  |
|  |  | C1QTNF2 |  |
|  |  | C21orf91 |  |
|  |  | C2orf43 |  |
|  |  | C2orf44 |  |
|  |  | C2orf83 |  |
|  |  | C3orf36 |  |
|  |  | GAS7 |  |
|  |  | C7orf25 |  |
|  |  | GDNF |  |
|  |  | PREPL |  |
|  |  | PRIM2 |  |
|  |  | CABP5 |  |
|  |  | CACNA2D2 |  |
|  |  | CACNA2D3 |  |
|  |  | CALCB |  |
|  |  | NOL12 |  |
|  |  | CALR |  |
|  |  | CARD6 |  |
|  |  | CCDC102B |  |
|  |  | CCDC109B |  |
|  |  | CCDC113 |  |
|  |  | CCDC122 |  |
|  |  | CCDC163P |  |
|  |  | CCDC169-SOHLH2 | |
|  |  | CCDC33 |  |
|  |  | LIX1L |  |
|  |  | CCDC64 |  |
|  |  | CCDC69 |  |
|  |  | CCNK |  |
|  |  | CCRN4L |  |
|  |  | CCS |  |
|  |  | CD1C |  |
|  |  | CD200R1 |  |
|  |  | LRRC3 |  |
|  |  | SLC5A10 |  |
|  |  | CD48 |  |
|  |  | OPCML |  |
|  |  | CDC7 |  |
|  |  | CDCA2 |  |
|  |  | CDCA4 |  |
|  |  | CDX2 |  |
|  |  | CDX4 |  |
|  |  | LYSMD4 |  |
|  |  | MAB21L1 |  |
|  |  | MAFG |  |
|  |  | MAGEA10 |  |
|  |  | MAGEA4 |  |
|  |  | MAGEB10 |  |
|  |  | CEP70 |  |
|  |  | CEP95 |  |
|  |  | CER1 |  |
|  |  | CETN3 |  |
|  |  | CFHR4 |  |
|  |  | CFHR5 |  |
|  |  | CGNL1 |  |
|  |  | MAPK8IP2 |  |
|  |  | CHRNA2 |  |
|  |  | CHST10 |  |
|  |  | CHST4 |  |
|  |  | CLDN16 |  |
|  |  | CLDN22 |  |
|  |  | CLEC1B |  |
|  |  | CLEC4C |  |
|  |  | CLHC1 |  |
|  |  | CLIC6 |  |
|  |  | PCDHGA9 |  |
|  |  | MESP1 |  |
|  |  | METTL7A |  |
|  |  | CNTLN |  |
|  |  | MFAP5 |  |
|  |  | MFHAS1 |  |
|  |  | COL15A1 |  |
|  |  | MGST3 |  |
|  |  | MINPP1 |  |
|  |  | PDYN |  |
|  |  | STEAP3 |  |
|  |  | MLH1 |  |
|  |  | STK17A |  |
|  |  | MLLT6 |  |
|  |  | STOML1 |  |
|  |  | MOAP1 |  |
|  |  | STRN4 |  |
|  |  | MRGPRX2 |  |
|  |  | MRPL13 |  |
|  |  | MRPL15 |  |
|  |  | SULT1C2 |  |
|  |  | SUN1 |  |
|  |  | IRF8 |  |
|  |  | MS4A2 |  |
|  |  | MS4A4E |  |
|  |  | SWT1 |  |
|  |  | MSH2 |  |
|  |  | MTRNR2L6 |  |
|  |  | KATNA1 |  |
|  |  | SDCCAG3 |  |
|  |  | MYO1B |  |
|  |  | MYO3B |  |
|  |  | MYOC |  |
|  |  | SELV |  |
|  |  | NAGS |  |
|  |  | NARS2 |  |
|  |  | NCK2 |  |
|  |  | NCR2 |  |
|  |  | SGSM1 |  |
|  |  | NDUFA2 |  |
|  |  | NDUFB3 |  |
|  |  | NECAP2 |  |
|  |  | NEIL3 |  |
|  |  | NFATC2 |  |
|  |  | NFIX |  |
|  |  | NHSL1 |  |
|  |  | NICN1 |  |
|  |  | NIPBL |  |
|  |  | NLGN4X |  |
|  |  | NME5 |  |
|  |  | NMNAT2 |  |
|  |  | GLRX |  |
|  |  | KRTAP19-2 |  |
|  |  | SLC13A1 |  |
|  |  | NOTCH4 |  |
|  |  | GNA12 |  |
|  |  | NOX5 |  |
|  |  | GNB5 |  |
|  |  | NPTX2 |  |
|  |  | NRSN1 |  |
|  |  | NSRP1 |  |
|  |  | GPHA2 |  |
|  |  | GPR112 |  |
|  |  | NUDT10 |  |
|  |  | GPRASP2 |  |
|  |  | GPRC5A |  |
|  |  | NUPL1 |  |
|  |  | OLFM3 |  |
|  |  | LRRD1 |  |
|  |  | OR2W3 |  |
|  |  | OR51E2 |  |
|  |  | ORC2 |  |
|  |  | OSBP |  |
|  |  | GVQW1 |  |
|  |  | PACS2 |  |
|  |  | PAK1 |  |
|  |  | PALLD |  |
|  |  | PAQR8 |  |
|  |  | PARP1 |  |
|  |  | HERC5 |  |
|  |  | PCDH7 |  |
|  |  | PCDHGA3 |  |
|  |  | PCDHGA4 |  |
|  |  | PCDHGA5 |  |
|  |  | PCDHGA7 |  |
|  |  | PCDHGA8 |  |
|  |  | HSDL2 |  |
|  |  | HSP90AA1 |  |
|  |  | SPTSSA |  |
|  |  | HTR1A |  |
|  |  | HTR2B |  |
|  |  | HTR6 |  |
|  |  | UHRF1BP1 |  |
|  |  | KDM2B |  |
|  |  | KDM4C |  |
|  |  | SSPN |  |
|  |  | USP10 |  |
|  |  | PDGFRA |  |
|  |  | PDGFRB |  |
|  |  | MICU2 |  |
|  |  | PDZD7 |  |
|  |  | UTP15 |  |
|  |  | PEAR1 |  |
|  |  | VBP1 |  |
|  |  | PRAF2 |  |
|  |  | C7orf76 |  |
|  |  | GFOD1 |  |
|  |  | INVS |  |
|  |  | SURF6 |  |
|  |  | HIST3H2BB |  |
|  |  | LCLAT1 |  |
|  |  | ITSN2 |  |
|  |  | MTHFD1 |  |
|  |  | TACR2 |  |
|  |  | TADA1 |  |
|  |  | GPC5 |  |
|  |  | TARS |  |
|  |  | TAS2R14 |  |
|  |  | LIPN |  |
|  |  | TATDN3 |  |
|  |  | SEMA4D |  |
|  |  | TCEB3 |  |
|  |  | SEPT3 |  |
|  |  | POLR1A |  |
|  |  | GFPT2 |  |
|  |  | PPEF1 |  |
|  |  | PPIH |  |
|  |  | PPP1R3B |  |
|  |  | PPP1R3G |  |
|  |  | PPRC1 |  |
|  |  | GEMIN6 |  |
|  |  | GIMAP8 |  |
|  |  | GIT1 |  |
|  |  | SIRT5 |  |
|  |  | HECTD4 |  |
|  |  | KRBA2 |  |
|  |  | SLC25A26 |  |
|  |  | GP6 |  |
|  |  | GPR160 |  |
|  |  | SLC35F4 |  |
|  |  | GPR37L1 |  |
|  |  | GPRIN2 |  |
|  |  | GPX6 |  |
|  |  | GRAMD1B |  |
|  |  | SLC4A1AP |  |
|  |  | STS |  |
|  |  | MFSD12 |  |
|  |  | LRRC6 |  |
|  |  | MGC10955 |  |
|  |  | GTF2F2 |  |
|  |  | GTF2H2 |  |
|  |  | GTF2H2C |  |
|  |  | GYPA |  |
|  |  | GYPE |  |
|  |  | HAUS3 |  |
|  |  | MB21D1 |  |
|  |  | SERPINA3 |  |
|  |  | MAGT1 |  |
|  |  | HEATR5B |  |
|  |  | HEATR6 |  |
|  |  | MAP1A |  |
|  |  | HIATL2 |  |
|  |  | MAT1A |  |
|  |  | SMIM11 |  |
|  |  | C9orf163 |  |
|  |  | HOXA4 |  |
|  |  | LIMK1 |  |
|  |  | MCM4 |  |
|  |  | MCM9 |  |
|  |  | SLC2A10 |  |
|  |  | SPIC |  |
|  |  | HSD17B1 |  |
|  |  | TBX22 |  |
|  |  | TBX5 |  |
|  |  | SYT5 |  |
|  |  | C9orf38 |  |
|  |  | KIAA0087 |  |
|  |  | MIA2 |  |
|  |  | MICA |  |
|  |  | UTP14C |  |
|  |  | UTS2 |  |
|  |  | STK38 |  |
|  |  | C7orf33 |  |
|  |  | C7orf71 |  |
|  |  | C9orf85 |  |
|  |  | KLRC3 |  |
|  |  | SLC12A4 |  |
|  |  | SLC17A2 |  |
|  |  | SLC17A3 |  |
|  |  | SYNM |  |
|  |  | MTCH1 |  |
|  |  | LRP1B |  |
|  |  | SMIM10 |  |
|  |  | LIG3 |  |
|  |  | SPEF2 |  |
|  |  | SLC2A5 |  |
|  |  | TBC1D25 |  |
|  |  | SLC35B3 |  |
|  |  | SLC35F3 |  |
|  |  | TCEAL1 |  |
|  |  | TCF21 |  |
|  |  | LRIG1 |  |
|  |  | SLC39A14 |  |
|  |  | SEPT8 |  |
|  |  | GDAP1 |  |
|  |  | TRMT61B |  |
|  |  | SETD6 |  |
|  |  | KIAA0430 |  |
|  |  | SIT1 |  |
|  |  | LSMEM1 |  |
|  |  | SHB |  |
|  |  | C7orf65 |  |
|  |  | MAS1 |  |
|  |  | SLC46A2 |  |
|  |  | TRNT1 |  |
|  |  | GJE1 |  |
|  |  | TTC17 |  |
|  |  | TTC19 |  |
|  |  | SNTN |  |
|  |  | SNX31 |  |
|  |  | SNX5 |  |
|  |  | MARC2 |  |
|  |  | LRP10 |  |
|  |  | UFL1 |  |
|  |  | SHROOM3 |  |
|  |  | SPATA12 |  |
|  |  | SLC26A9 |  |
|  |  | SLC28A1 |  |
|  |  | LSM3 |  |
|  |  | LST3 |  |
|  |  | LTF |  |
|  |  | KCNJ14 |  |
|  |  | SLITRK3 |  |
|  |  | SLC37A4 |  |
|  |  | LRMP |  |
|  |  | SOHLH2 |  |
|  |  | SIGLEC14 |  |
|  |  | USP24 |  |
|  |  | SLC12A3 |  |
|  |  | SLCO3A1 |  |
|  |  | SLIT2 |  |
|  |  | LYRM9 |  |
|  |  | KLHL13 |  |
|  |  | SMPD3 |  |
|  |  | KCTD15 |  |
|  |  | SPDYA |  |
|  |  | SLCO1B7 |  |
|  |  | TUB |  |
|  |  | SRRD |  |
|  |  | SHROOM1 |  |
|  |  | SIGLEC8 |  |
|  |  | SYPL2 |  |
|  |  | SLC9A5 |  |
|  |  | SNRK |  |
|  |  | SRGAP3 |  |
|  |  | KCNK17 |  |
|  |  | ST3GAL5 |  |
|  |  | SMURF1 |  |
|  |  | TYW3 |  |
|  |  | SMIM18 |  |
|  |  | SLC25A37 |  |
|  |  | SIAH3 |  |
|  |  | SLCO1B1 |  |
|  |  | KIF15 |  |
|  |  | KIF17 |  |
|  |  | KCNK9 |  |
|  |  | CHRNG |  |
|  |  | ZNF444 |  |
|  |  | RP11-195F19.5 |  |
|  |  | RNF151 |  |
|  |  | ZNF853 |  |
|  |  | RAD23A |  |
|  |  | UBE2Z |  |
|  |  | RP11-15E18.4 |  |
|  |  | RP11-497E19.2 |  |
|  |  | USF1 |  |
|  |  | RP11-114H20.1 |  |
|  |  | ZNF862 |  |
|  |  | ZNF443 |  |
|  |  | RP11-826N14.2 |  |
|  |  | RNFT2 |  |
|  |  | RNF166 |  |
|  |  | RP11-144F15.1 |  |
|  |  | SLURP1 |  |
|  |  | ZNF784 |  |
|  |  | UQCRC1 |  |
|  |  | TMPRSS3 |  |
|  |  | RDH13 |  |
|  |  | ZNF212 |  |
|  |  | ZNF114 |  |
|  |  | ZNF599 |  |
|  |  | RP11-404P21.8 |  |
|  |  | RNF34 |  |
|  |  | UBIAD1 |  |
|  |  | ZNF773 |  |
|  |  | UBL4B |  |
|  |  | ZNF672 |  |
|  |  | ZNF687 |  |
|  |  | RDH12 |  |
|  |  | RBM34 |  |
|  |  | REEP2 |  |
|  |  | SLC3A1 |  |
|  |  | WDR52 |  |
|  |  | RP11-1C1.5 |  |
|  |  | WDR27 |  |
|  |  | RPA4 |  |
|  |  | RAPGEF3 |  |
|  |  | ZNF593 |  |
|  |  | ZNF346 |  |
|  |  | ZNF839 |  |
|  |  | ZBTB22 |  |
|  |  | SMDT1 |  |
|  |  | ZNF782 |  |
|  |  | RAET1E |  |
|  |  | SEC16B |  |
|  |  | RNF220 |  |
|  |  | SLC41A3 |  |
|  |  | UCKL1 |  |
|  |  | RNF8 |  |
|  |  | COL26A1 |  |
|  |  | C11orf44 |  |
|  |  | ZNF395 |  |
|  |  | TMEM242 |  |
|  |  | ZNF646 |  |
|  |  | ZGPAT |  |
|  |  | RP11-834C11.12 |  |
|  |  | ZNF688 |  |
|  |  | ZNF69 |  |
|  |  | ZNF697 |  |
|  |  | RP11-204N11.1 |  |
|  |  | RAB8A |  |
|  |  | ZFP82 |  |
|  |  | RP11-480I12.4 |  |
|  |  | ZNF768 |  |
|  |  | RAD51D |  |
|  |  | ZFYVE20 |  |
|  |  | ZBTB7B |  |
|  |  | ZHX1-C8ORF76 |  |
|  |  | TMEM27 |  |
|  |  | WDR38 |  |
|  |  | RNF10 |  |
|  |  | RAMP3 |  |
|  |  | ZKSCAN5 |  |
|  |  | ZMAT5 |  |
|  |  | UBE2L3 |  |
|  |  | RANGAP1 |  |
|  |  | RENBP |  |
|  |  | RERE |  |
|  |  | RFC1 |  |
|  |  | ZG16 |  |
|  |  | TNFSF9 |  |
|  |  | ZNF213 |  |
|  |  | ZNF576 |  |
|  |  | ZNF581 |  |
|  |  | WNT4 |  |
|  |  | RABEP2 |  |
|  |  | UBE4B |  |
|  |  | TMEM240 |  |
|  |  | SLFNL1 |  |
|  |  | TMEM259 |  |
|  |  | ZNF689 |  |
|  |  | UQCR11 |  |
|  |  | ZFAND2B |  |
|  |  | ZFC3H1 |  |
|  |  | WNT7B |  |
|  |  | VSTM1 |  |
|  |  | SLC35C2 |  |
|  |  | ZNF362 |  |
|  |  | URM1 |  |
|  |  | USB1 |  |
|  |  | RCOR2 |  |
|  |  | TRABD2A |  |
|  |  | TRADD |  |
|  |  | TRAF2 |  |
|  |  | RAP1GAP |  |
|  |  | ZNF497 |  |
|  |  | SLC50A1 |  |
|  |  | UBE3D |  |
|  |  | ZNF512 |  |
|  |  | WFDC1 |  |
|  |  | RNF180 |  |
|  |  | UBL5 |  |
|  |  | RNF187 |  |
|  |  | TPRX1 |  |
|  |  | RNF212 |  |
|  |  | DDI1 |  |
|  |  | RNF215 |  |
|  |  | SLC25A35 |  |
|  |  | WNT1 |  |
|  |  | WNT10B |  |
|  |  | ZNF214 |  |
|  |  | ZNF23 |  |
|  |  | ZNF233 |  |
|  |  | UGDH |  |
|  |  | VOPP1 |  |
|  |  | TRIP10 |  |
|  |  | ZDHHC19 |  |
|  |  | TMEM258 |  |
|  |  | DFFB |  |
|  |  | DGCR14 |  |
|  |  | SLX1A |  |
|  |  | ZNF324 |  |
|  |  | DGCR8 |  |
|  |  | RBP1 |  |
|  |  | RBP3 |  |
|  |  | RP11-293M10.1 |  |
|  |  | RP11-295D22.1 |  |
|  |  | RABL3 |  |
|  |  | RABL5 |  |
|  |  | RP11-3B7.1 |  |
|  |  | SMARCB1 |  |
|  |  | ZFR2 |  |
|  |  | ZNF747 |  |
|  |  | ZNF764 |  |
|  |  | ZNF765 |  |
|  |  | SMG5 |  |
|  |  | TPTE |  |
|  |  | RALGDS |  |
|  |  | RANBP10 |  |
|  |  | ZBTB40 |  |
|  |  | ZBTB42 |  |
|  |  | TMEM204 |  |
|  |  | TMEM213 |  |
|  |  | ZNF575 |  |
|  |  | ZNF580 |  |
|  |  | SLC9A1 |  |
|  |  | ZC3HAV1L |  |
|  |  | SLC9A3R2 |  |
|  |  | SLC9A8 |  |
|  |  | ZNF607 |  |
|  |  | TRIOBP |  |
|  |  | ZDHHC13 |  |
|  |  | SIRPA |  |
|  |  | TRIT1 |  |
|  |  | TRMT1 |  |
|  |  | TRMT10B |  |
|  |  | SIX5 |  |
|  |  | TMEM37 |  |
|  |  | VPS37C |  |
|  |  | DFNB59 |  |
|  |  | SLX1B |  |
|  |  | DGCR6L |  |
|  |  | RP11-247C2.2 |  |
|  |  | TMEM62 |  |
|  |  | TMEM63B |  |
|  |  | RD3 |  |
|  |  | RDH11 |  |
|  |  | RECQL5 |  |
|  |  | CTF1 |  |
|  |  | SLC22A11 |  |
|  |  | SLC22A12 |  |
|  |  | SLC22A13 |  |
|  |  | ZMIZ1 |  |
|  |  | SLC23A3 |  |
|  |  | ZNF121 |  |
|  |  | ZNF124 |  |
|  |  | SLC25A11 |  |
|  |  | SLC25A14 |  |
|  |  | ZNF184 |  |
|  |  | UBR2 |  |
|  |  | RGL4 |  |
|  |  | ZNF219 |  |
|  |  | RGS11 |  |
|  |  | RGS12 |  |
|  |  | RGS16 |  |
|  |  | RGS22 |  |
|  |  | RGS3 |  |
|  |  | UGGT2 |  |
|  |  | ZCWPW2 |  |
|  |  | RGSL1 |  |
|  |  | ZDHHC16 |  |
|  |  | RHOC |  |
|  |  | RHOD |  |
|  |  | RHOF |  |
|  |  | ZNF296 |  |
|  |  | ZNF300 |  |
|  |  | UNC5A |  |
|  |  | ZNF319 |  |
|  |  | ZNF321P |  |
|  |  | RHOV |  |
|  |  | ZNF329 |  |
|  |  | ZNF343 |  |
|  |  | RIMS3 |  |
|  |  | ZFYVE28 |  |
|  |  | RLN2 |  |
|  |  | RMDN3 |  |
|  |  | RMI2 |  |
|  |  | RNASE6 |  |
|  |  | RND2 |  |
|  |  | ZNF446 |  |
|  |  | RNF122 |  |
|  |  | RNF126 |  |
|  |  | ZMIZ2 |  |
|  |  | RNF130 |  |
|  |  | RNF14 |  |
|  |  | SLC47A1 |  |
|  |  | UTF1 |  |
|  |  | UTP6 |  |
|  |  | RNF181 |  |
|  |  | RNF185 |  |
|  |  | RNF19B |  |
|  |  | VAMP8 |  |
|  |  | VARS2 |  |
|  |  | VASN |  |
|  |  | VAT1 |  |
|  |  | VAT1L |  |
|  |  | VENTX |  |
|  |  | VGLL1 |  |
|  |  | VIPR1 |  |
|  |  | VIPR2 |  |
|  |  | ROPN1B |  |
|  |  | TWF2 |  |
|  |  | RORC |  |
|  |  | RP11-1026M7.2 |  |
|  |  | RP11-1070N10.3 |  |
|  |  | RP11-108K14.8 |  |
|  |  | PPIA |  |
|  |  | SLX4 |  |
|  |  | ZNF705A |  |
|  |  | DGKG |  |
|  |  | ZNF710 |  |
|  |  | ZNF720 |  |
|  |  | RP11-47I22.3 |  |
|  |  | RP11-625H11.1 |  |
|  |  | RP11-650K20.3 |  |
|  |  | SMEK1 |  |
|  |  | RP11-723O4.6 |  |
|  |  | DIAPH3 |  |
|  |  | RP11-934B9.3 |  |
|  |  | RALGAPA2 |  |
|  |  | TSPAN4 |  |
|  |  | RP6-24A23.6 |  |
|  |  | TMLHE |  |
|  |  | TMOD1 |  |
|  |  | RPE65 |  |
|  |  | ZNF845 |  |
|  |  | RANGRF |  |
|  |  | TSSC4 |  |
|  |  | TSSK1B |  |
|  |  | RPL10 |  |
|  |  | TTC1 |  |
|  |  | RAPGEF6 |  |
|  |  | RAPSN |  |
|  |  | RARA |  |
|  |  | RASA4B |  |
|  |  | RASGEF1C |  |
|  |  | ZC3H7B |  |
|  |  | ZC3H8 |  |
|  |  | TMEM232 |  |
|  |  | ZCCHC12 |  |
|  |  | ZCCHC18 |  |
|  |  | ZCCHC3 |  |
|  |  | ZCWPW1 |  |
|  |  | TOP3A |  |
|  |  | RBM19 |  |
|  |  | TP53TG3 |  |
|  |  | TPGS1 |  |
|  |  | RCBTB2 |  |
|  |  | UBALD2 |  |
|  |  | UBAP2L |  |
|  |  | USP39 |  |
|  |  | USP41 |  |
|  |  | USP43 |  |
|  |  | RECK |  |
|  |  | REEP4 |  |
|  |  | REG1A |  |
|  |  | RELA |  |
|  |  | RELT |  |
|  |  | REM1 |  |
|  |  | REM2 |  |
|  |  | REN |  |
|  |  | UBE2R2 |  |
|  |  | C10orf95 |  |
|  |  | C10orf99 |  |
|  |  | REXO1L1 |  |
|  |  | REXO2 |  |
|  |  | REXO4 |  |
|  |  | C11orf49 |  |
|  |  | C11orf54 |  |
|  |  | C11orf57 |  |
|  |  | RFNG |  |
|  |  | RFT1 |  |
|  |  | RFTN1 |  |
|  |  | RFWD2 |  |
|  |  | RFX1 |  |
|  |  | RGAG4 |  |
|  |  | VILL |  |
|  |  | RGMB |  |
|  |  | RGP1 |  |
|  |  | VMAC |  |
|  |  | VNN3 |  |
|  |  | RGS8 |  |
|  |  | RGS9 |  |
|  |  | RHBDD1 |  |
|  |  | RHBDD2 |  |
|  |  | RHBDF2 |  |
|  |  | RHBDL3 |  |
|  |  | RHCG |  |
|  |  | RHD |  |
|  |  | RHEB |  |
|  |  | RHOB |  |
|  |  | RHOBTB2 |  |
|  |  | TRMT2A |  |
|  |  | TRMT2B |  |
|  |  | RHOG |  |
|  |  | RHOT2 |  |
|  |  | VSTM5 |  |
|  |  | RHPN1 |  |
|  |  | CYS1 |  |
|  |  | RIMS4 |  |
|  |  | RIN1 |  |
|  |  | RING1 |  |
|  |  | RIPK2 |  |
|  |  | RIPPLY1 |  |
|  |  | RIPPLY3 |  |
|  |  | WDFY4 |  |
|  |  | WDR11 |  |
|  |  | SEC14L6 |  |
|  |  | WDR5 |  |
|  |  | WDR6 |  |
|  |  | WDR74 |  |
|  |  | C1orf170 |  |
|  |  | WDR81 |  |
|  |  | WDR83 |  |
|  |  | WDR83OS |  |
|  |  | WDTC1 |  |
|  |  | WFDC3 |  |
|  |  | VAMP2 |  |
|  |  | VAMP5 |  |
|  |  | WIBG |  |
|  |  | WISP2 |  |
|  |  | WNK4 |  |
|  |  | WNT11 |  |
|  |  | SEPT6 |  |
|  |  | WNT5B |  |
|  |  | SERF1B |  |
|  |  | WRNIP1 |  |
|  |  | WSCD2 |  |
|  |  | WTIP |  |
|  |  | XAF1 |  |
|  |  | RP11-1102P16.1 |  |
|  |  | XKR8 |  |
|  |  | DGAT1 |  |
|  |  | XPNPEP2 |  |
|  |  | VPS54 |  |
|  |  | XPO6 |  |
|  |  | RP11-268J15.5 |  |
|  |  | RP1-127H14.3 |  |
|  |  | RP11-343C2.12 |  |
|  |  | XYLT2 |  |
|  |  | YARS |  |
|  |  | YBX2 |  |
|  |  | YIF1A |  |
|  |  | YIF1B |  |
|  |  | SGCA |  |
|  |  | YWHAH |  |
|  |  | ZAP70 |  |
|  |  | ZBED1 |  |
|  |  | ZBP1 |  |
|  |  | ZNF501 |  |
|  |  | ZNF513 |  |
|  |  | ZNF517 |  |
|  |  | ZNF524 |  |
|  |  | ZNF526 |  |
|  |  | ZNF540 |  |
|  |  | RASL12 |  |
|  |  | ZNF561 |  |
|  |  | ZNF564 |  |
|  |  | RBBP8NL |  |
|  |  | RBCK1 |  |
|  |  | RBFA |  |
|  |  | RBFOX3 |  |
|  |  | ZNF653 |  |
|  |  | ZNF668 |  |
|  |  | ZNF691 |  |
|  |  | ZNF750 |  |
|  |  | ZNF75A |  |
|  |  | ZNF771 |  |
|  |  | ZNF778 |  |
|  |  | ZNF786 |  |
|  |  | ZNF799 |  |
|  |  | ZNF821 |  |
|  |  | CHRNA4 |  |
|  |  | CHRNA6 |  |
|  |  | CHRNB4 |  |
|  |  | CHRNE |  |
|  |  | MKNK1 |  |
|  |  | ZNF891 |  |
|  |  | ZNRF1 |  |
|  |  | CIDEB |  |
|  |  | ZZEF1 |  |
|  |  | CKAP5 |  |
|  |  | ZNF251 |  |
|  |  | ZNF276 |  |
|  |  | ZNF280B |  |
|  |  | TOR2A |  |
|  |  | TOR4A |  |
|  |  | TP53TG3B |  |
|  |  | TP53TG3C |  |
|  |  | ZNF335 |  |
|  |  | ZNF33A |  |
|  |  | TPCN1 |  |
|  |  | TPCN2 |  |
|  |  | CLINT1 |  |
|  |  | CLIP2 |  |
|  |  | TPMT |  |
|  |  | TPP2 |  |
|  |  | TPPP3 |  |
|  |  | SLC37A1 |  |
|  |  | ZNF407 |  |
|  |  | CLUH |  |
|  |  | CMKLR1 |  |
|  |  | CMPK2 |  |
|  |  | CMTM7 |  |
|  |  | CMTM8 |  |
|  |  | SLC47A2 |  |
|  |  | SLC48A1 |  |
|  |  | SLC4A1 |  |
|  |  | ZNF528 |  |
|  |  | ZNF529 |  |
|  |  | SLC6A6 |  |
|  |  | SLC7A4 |  |
|  |  | TRIM40 |  |
|  |  | TRIM45 |  |
|  |  | COL11A2 |  |
|  |  | COL17A1 |  |
|  |  | COL18A1 |  |
|  |  | COL1A1 |  |
|  |  | MTG1 |  |
|  |  | COL5A1 |  |
|  |  | COL6A1 |  |
|  |  | COL6A3 |  |
|  |  | COL8A2 |  |
|  |  | COMMD5 |  |
|  |  | COMT |  |
|  |  | COPG1 |  |
|  |  | COPS6 |  |
|  |  | COQ9 |  |
|  |  | CORO2A |  |
|  |  | CORO2B |  |
|  |  | CORT |  |
|  |  | COX14 |  |
|  |  | COX6B2 |  |
|  |  | CPA5 |  |
|  |  | CPLX1 |  |
|  |  | CPLX3 |  |
|  |  | CPLX4 |  |
|  |  | CPN2 |  |
|  |  | CPNE1 |  |
|  |  | CPNE2 |  |
|  |  | CPNE5 |  |
|  |  | CPNE7 |  |
|  |  | CPNE9 |  |
|  |  | CPSF3 |  |
|  |  | CPSF7 |  |
|  |  | CPT1B |  |
|  |  | CPXM2 |  |
|  |  | CRAT |  |
|  |  | CRB1 |  |
|  |  | CRB2 |  |
|  |  | CREB3L3 |  |
|  |  | CRELD1 |  |
|  |  | CRIP2 |  |
|  |  | RASSF1 |  |
|  |  | RASSF2 |  |
|  |  | ZNF57 |  |
|  |  | ZNF574 |  |
|  |  | TOM1 |  |
|  |  | TOMM34 |  |
|  |  | TOMM40L |  |
|  |  | RBM10 |  |
|  |  | TUBGCP6 |  |
|  |  | TULP3 |  |
|  |  | TUSC1 |  |
|  |  | TVP23C-CDRT4 |  |
|  |  | CSMD2 |  |
|  |  | BRD1 |  |
|  |  | TXN2 |  |
|  |  | TXNDC8 |  |
|  |  | TXNDC9 |  |
|  |  | TXNIP |  |
|  |  | TXNRD2 |  |
|  |  | TYR |  |
|  |  | UACA |  |
|  |  | UAP1L1 |  |
|  |  | UBA1 |  |
|  |  | UBAC1 |  |
|  |  | UBALD1 |  |
|  |  | CTCFL |  |
|  |  | UBAP1L |  |
|  |  | UBE2B |  |
|  |  | UBE2C |  |
|  |  | UBE2D4 |  |
|  |  | UBE2G2 |  |
|  |  | UBE2I |  |
|  |  | UBE2J2 |  |
|  |  | UBE2M |  |
|  |  | UBE2O |  |
|  |  | CTNS |  |
|  |  | CTPS1 |  |
|  |  | CTSA |  |
|  |  | CTSE |  |
|  |  | C11orf21 |  |
|  |  | C11orf45 |  |
|  |  | CTXN3 |  |
|  |  | CUBN |  |
|  |  | CUEDC1 |  |
|  |  | C11orf68 |  |
|  |  | UBL7 |  |
|  |  | UBOX5 |  |
|  |  | UBQLN3 |  |
|  |  | UBQLNL |  |
|  |  | CWF19L1 |  |
|  |  | UBR4 |  |
|  |  | UBXN10 |  |
|  |  | UCHL1 |  |
|  |  | C14orf119 |  |
|  |  | C14orf132 |  |
|  |  | UCMA |  |
|  |  | UCP3 |  |
|  |  | UFC1 |  |
|  |  | C14orf80 |  |
|  |  | UHRF2 |  |
|  |  | ULK3 |  |
|  |  | UMOD |  |
|  |  | C16orf59 |  |
|  |  | UMPS |  |
|  |  | UNC119 |  |
|  |  | UNC119B |  |
|  |  | UNC13A |  |
|  |  | C16orf92 |  |
|  |  | UPF2 |  |
|  |  | UPK1A |  |
|  |  | UPP1 |  |
|  |  | UPP2 |  |
|  |  | C17orf78 |  |
|  |  | C17orf80 |  |
|  |  | UQCC2 |  |
|  |  | UQCR10 |  |
|  |  | C19orf24 |  |
|  |  | USF2 |  |
|  |  | C19orf38 |  |
|  |  | USH1G |  |
|  |  | C19orf47 |  |
|  |  | USP19 |  |
|  |  | USP2 |  |
|  |  | USP20 |  |
|  |  | USP35 |  |
|  |  | USP36 |  |
|  |  | USP37 |  |
|  |  | DCLK2 |  |
|  |  | DCTD |  |
|  |  | DCTN1 |  |
|  |  | NOL6 |  |
|  |  | DDA1 |  |
|  |  | DDB1 |  |
|  |  | NOTUM |  |
|  |  | DDIT3 |  |
|  |  | DDR2 |  |
|  |  | DDT |  |
|  |  | DDX18 |  |
|  |  | DDX49 |  |
|  |  | DDX54 |  |
|  |  | DDX55 |  |
|  |  | DDX56 |  |
|  |  | DEC1 |  |
|  |  | DEDD2 |  |
|  |  | DEFB119 |  |
|  |  | DEGS1 |  |
|  |  | DEK |  |
|  |  | VPREB3 |  |
|  |  | VPS11 |  |
|  |  | C2orf68 |  |
|  |  | VPS26B |  |
|  |  | VPS28 |  |
|  |  | VPS33A |  |
|  |  | DFFA |  |
|  |  | NRARP |  |
|  |  | VPS45 |  |
|  |  | NRDE2 |  |
|  |  | NRF1 |  |
|  |  | VRK3 |  |
|  |  | VSIG10L |  |
|  |  | DGKQ |  |
|  |  | DHCR7 |  |
|  |  | DHFRL1 |  |
|  |  | VWA1 |  |
|  |  | VWA2 |  |
|  |  | VWA5A |  |
|  |  | VWA5B1 |  |
|  |  | RP11-676J12.7 |  |
|  |  | RP11-683L23.1 |  |
|  |  | VWF |  |
|  |  | WASF2 |  |
|  |  | RP13-996F3.4 |  |
|  |  | RP4-539M6.19 |  |
|  |  | DIRAS1 |  |
|  |  | RPA2 |  |
|  |  | DISP2 |  |
|  |  | RPH3A |  |
|  |  | RPIA |  |
|  |  | RPL12 |  |
|  |  | RPL13A |  |
|  |  | RPL23 |  |
|  |  | RPL27A |  |
|  |  | RPL28 |  |
|  |  | RPL3 |  |
|  |  | RPL31 |  |
|  |  | RPL36 |  |
|  |  | RPL38 |  |
|  |  | RPL3L |  |
|  |  | RPL8 |  |
|  |  | CREG1 |  |
|  |  | RPP30 |  |
|  |  | CRHR1 |  |
|  |  | CRIP1 |  |
|  |  | RPRD1B |  |
|  |  | RPRD2 |  |
|  |  | RPRM |  |
|  |  | RPS16 |  |
|  |  | RPS19BP1 |  |
|  |  | RPS2 |  |
|  |  | RAVER1 |  |
|  |  | TOB2 |  |
|  |  | TOM1L2 |  |
|  |  | TOMM20 |  |
|  |  | RPUSD1 |  |
|  |  | RPUSD2 |  |
|  |  | RRAGA |  |
|  |  | RRAS |  |
|  |  | RRNAD1 |  |
|  |  | RRP1 |  |
|  |  | RRP12 |  |
|  |  | RSAD1 |  |
|  |  | RSC1A1 |  |
|  |  | TAX1BP3 |  |
|  |  | URGCP-MRPS24 |  |
|  |  | UROC1 |  |
|  |  | USP18 |  |
|  |  | TBC1D3 |  |
|  |  | RUSC1 |  |
|  |  | RUVBL1 |  |
|  |  | USP5 |  |
|  |  | RXFP2 |  |
|  |  | OSGEPL1 |  |
|  |  | UVSSA |  |
|  |  | VAC14 |  |
|  |  | S100PBP |  |
|  |  | TCF19 |  |
|  |  | SARS |  |
|  |  | SART1 |  |
|  |  | SASH3 |  |
|  |  | VKORC1 |  |
|  |  | SBK2 |  |
|  |  | SCAMP2 |  |
|  |  | SCAMP3 |  |
|  |  | SCAMP4 |  |
|  |  | VPS37D |  |
|  |  | VPS8 |  |
|  |  | VWA3A |  |
|  |  | VWA5B2 |  |
|  |  | VWCE |  |
|  |  | WASH4P |  |
|  |  | WBSCR17 |  |
|  |  | WBSCR22 |  |
|  |  | SDSL |  |
|  |  | SEC14L3 |  |
|  |  | SEC14L4 |  |
|  |  | EIF2AK4 |  |
|  |  | THNSL1 |  |
|  |  | THNSL2 |  |
|  |  | SEC31A |  |
|  |  | WDR62 |  |
|  |  | SELE |  |
|  |  | SELP |  |
|  |  | WDR86 |  |
|  |  | TIMD4 |  |
|  |  | TIMELESS |  |
|  |  | SEMA3F |  |
|  |  | WFDC5 |  |
|  |  | WFIKKN2 |  |
|  |  | SEMA4G |  |
|  |  | TINF2 |  |
|  |  | TJAP1 |  |
|  |  | TK1 |  |
|  |  | TKT |  |
|  |  | TLCD2 |  |
|  |  | TLE1 |  |
|  |  | TLE2 |  |
|  |  | TLE3 |  |
|  |  | TLN1 |  |
|  |  | TLR10 |  |
|  |  | TLR5 |  |
|  |  | TLX2 |  |
|  |  | TMA7 |  |
|  |  | TMBIM4 |  |
|  |  | TMBIM6 |  |
|  |  | TMC3 |  |
|  |  | XG |  |
|  |  | SERTAD3 |  |
|  |  | SERTM1 |  |
|  |  | SETD1A |  |
|  |  | XPC |  |
|  |  | TMEM100 |  |
|  |  | SF3B2 |  |
|  |  | SFN |  |
|  |  | SFRP1 |  |
|  |  | SFTA2 |  |
|  |  | SFTPA2 |  |
|  |  | SFXN2 |  |
|  |  | SGK2 |  |
|  |  | SH2D1B |  |
|  |  | SH2D2A |  |
|  |  | SH2D5 |  |
|  |  | SH2D7 |  |
|  |  | SH3BP1 |  |
|  |  | SH3GL1 |  |
|  |  | ESYT3 |  |
|  |  | SH3YL1 |  |
|  |  | SHANK1 |  |
|  |  | SHANK3 |  |
|  |  | EVA1C |  |
|  |  | SHISA4 |  |
|  |  | EVPLL |  |
|  |  | SHISA7 |  |
|  |  | SHPK |  |
|  |  | SHQ1 |  |
|  |  | SHROOM4 |  |
|  |  | SIAH2 |  |
|  |  | SIDT1 |  |
|  |  | SIGLEC1 |  |
|  |  | SIL1 |  |
|  |  | SIN3B |  |
|  |  | SIPA1 |  |
|  |  | SIPA1L3 |  |
|  |  | PHGR1 |  |
|  |  | SIRPB2 |  |
|  |  | SIRT3 |  |
|  |  | SKA1 |  |
|  |  | SKAP1 |  |
|  |  | SKIV2L2 |  |
|  |  | PHYKPL |  |
|  |  | PI16 |  |
|  |  | SLAMF6 |  |
|  |  | SLBP |  |
|  |  | SLC11A1 |  |
|  |  | SLC12A1 |  |
|  |  | SLC12A9 |  |
|  |  | SLC16A3 |  |
|  |  | SLC17A9 |  |
|  |  | SLC18A1 |  |
|  |  | FAM131A |  |
|  |  | SLC18B1 |  |
|  |  | SLC19A3 |  |
|  |  | SLC1A4 |  |
|  |  | SLC1A6 |  |
|  |  | SLC1A7 |  |
|  |  | FAM153A |  |
|  |  | SLC22A16 |  |
|  |  | SLC22A17 |  |
|  |  | SLC22A2 |  |
|  |  | SLC22A25 |  |
|  |  | SLC22A4 |  |
|  |  | SLC22A9 |  |
|  |  | FAM167B |  |
|  |  | SLC24A2 |  |
|  |  | SLC24A5 |  |
|  |  | SLC25A10 |  |
|  |  | SLC25A17 |  |
|  |  | SLC25A22 |  |
|  |  | SLC25A23 |  |
|  |  | SLC25A25 |  |
|  |  | SLC25A31 |  |
|  |  | FAM189A1 |  |
|  |  | PLBD2 |  |
|  |  | SLC25A39 |  |
|  |  | SLC25A42 |  |
|  |  | SLC25A47 |  |
|  |  | SLC25A6 |  |
|  |  | SLC26A1 |  |
|  |  | SLC26A5 |  |
|  |  | SLC26A8 |  |
|  |  | SLC27A1 |  |
|  |  | SLC28A3 |  |
|  |  | SLC29A1 |  |
|  |  | SLC29A3 |  |
|  |  | SLC29A4 |  |
|  |  | SLC2A2 |  |
|  |  | SLC2A6 |  |
|  |  | SLC31A2 |  |
|  |  | SLC34A1 |  |
|  |  | SLC35A2 |  |
|  |  | SLC35A4 |  |
|  |  | SLC35B1 |  |
|  |  | PLXNB3 |  |
|  |  | PLXND1 |  |
|  |  | SLC35E2 |  |
|  |  | SLC35E2B |  |
|  |  | FAM83E |  |
|  |  | SLC38A10 |  |
|  |  | SLC38A11 |  |
|  |  | SLC39A13 |  |
|  |  | SLC39A4 |  |
|  |  | FANCD2 |  |
|  |  | SLC43A1 |  |
|  |  | SLC44A2 |  |
|  |  | SLC46A1 |  |
|  |  | POLM |  |
|  |  | POLR1C |  |
|  |  | POLR1E |  |
|  |  | POLR2A |  |
|  |  | SLC52A3 |  |
|  |  | SLC5A5 |  |
|  |  | SLC6A13 |  |
|  |  | SLC6A16 |  |
|  |  | SLC6A19 |  |
|  |  | SLC6A2 |  |
|  |  | SLC6A3 |  |
|  |  | POR |  |
|  |  | PORCN |  |
|  |  | POTED |  |
|  |  | POTEF |  |
|  |  | SLC7A8 |  |
|  |  | FBXO44 |  |
|  |  | SLC9A9 |  |
|  |  | SLC9C1 |  |
|  |  | SLCO4A1 |  |
|  |  | SLFN11 |  |
|  |  | SLFN13 |  |
|  |  | PPARD |  |
|  |  | PPARG |  |
|  |  | PPCDC |  |
|  |  | PPDPF |  |
|  |  | PPIE |  |
|  |  | PPIP5K1 |  |
|  |  | SMAD7 |  |
|  |  | SMAP2 |  |
|  |  | SMARCC2 |  |
|  |  | SMCO3 |  |
|  |  | PPP1R1A |  |
|  |  | PPP1R21 |  |
|  |  | FHOD3 |  |
|  |  | FIBCD1 |  |
|  |  | SMG8 |  |
|  |  | ZNF792 |  |
|  |  | SMTNL1 |  |
|  |  | ZNF865 |  |
|  |  | SNAI3 |  |
|  |  | SNAP23 |  |
|  |  | SNAPC4 |  |
|  |  | SNCG |  |
|  |  | SND1 |  |
|  |  | ZSCAN32 |  |
|  |  | ZSCAN4 |  |
|  |  | ZSCAN9 |  |
|  |  | CRKL |  |
|  |  | CRTAC1 |  |
|  |  | CRYAA |  |
|  |  | CRYBA1 |  |
|  |  | CRYBB1 |  |
|  |  | CRYGN |  |
|  |  | CRYGS |  |
|  |  | CRYZL1 |  |
|  |  | CSDC2 |  |
|  |  | CSF1 |  |
|  |  | CSF2RB |  |
|  |  | CSH2 |  |
|  |  | CSHL1 |  |
|  |  | SORBS3 |  |
|  |  | CSNK1G2 |  |
|  |  | CSNK2A1 |  |
|  |  | CSNK2B-LY6G5B-1181 | |
|  |  | CSRP1 |  |
|  |  | CST3 |  |
|  |  | CSTB |  |
|  |  | CSTF1 |  |
|  |  | CT62 |  |
|  |  | CTAG1A |  |
|  |  | CTAG1B |  |
|  |  | CTB-186H2.3 |  |
|  |  | CTC-349C3.1 |  |
|  |  | CTC-432M15.3 |  |
|  |  | SPA17 |  |
|  |  | CTD-2054N24.2 |  |
|  |  | CTD-2207O23.12 |  |
|  |  | CTD-2267D19.3 |  |
|  |  | CTD-2616J11.4 |  |
|  |  | SPATA5 |  |
|  |  | CTDNEP1 |  |
|  |  | CTDP1 |  |
|  |  | CTDSP1 |  |
|  |  | CTDSP2 |  |
|  |  | CTNNA2 |  |
|  |  | CTNNBIP1 |  |
|  |  | CTNNBL1 |  |
|  |  | CTNND1 |  |
|  |  | SPG20OS |  |
|  |  | SPG21 |  |
|  |  | SPG7 |  |
|  |  | SPHK1 |  |
|  |  | CTSW |  |
|  |  | CTU1 |  |
|  |  | SPNS2 |  |
|  |  | SPOCD1 |  |
|  |  | SPOCK2 |  |
|  |  | CUL1 |  |
|  |  | CUX1 |  |
|  |  | CUZD1 |  |
|  |  | CWC22 |  |
|  |  | CWC25 |  |
|  |  | SPRR2D |  |
|  |  | CX3CL1 |  |
|  |  | CXCL16 |  |
|  |  | CXCL9 |  |
|  |  | CXCR1 |  |
|  |  | CXCR2 |  |
|  |  | CXCR5 |  |
|  |  | CXorf21 |  |
|  |  | CXorf28 |  |
|  |  | CXorf36 |  |
|  |  | CXorf40A |  |
|  |  | CXorf40B |  |
|  |  | CXorf67 |  |
|  |  | CXXC11 |  |
|  |  | CYB561 |  |
|  |  | CYB561A3 |  |
|  |  | CYB5D2 |  |
|  |  | CYB5R3 |  |
|  |  | CYC1 |  |
|  |  | CYHR1 |  |
|  |  | CYP11A1 |  |
|  |  | CYP1A2 |  |
|  |  | CYP26B1 |  |
|  |  | CYP2C19 |  |
|  |  | CYP2C8 |  |
|  |  | CYP2S1 |  |
|  |  | CYP2W1 |  |
|  |  | CYP3A4 |  |
|  |  | CYP3A5 |  |
|  |  | CYP46A1 |  |
|  |  | CYP4A22 |  |
|  |  | CYP4F22 |  |
|  |  | NINJ1 |  |
|  |  | CYTH1 |  |
|  |  | CYTH4 |  |
|  |  | D2HGDH |  |
|  |  | DACT2 |  |
|  |  | DAK |  |
|  |  | DAPK2 |  |
|  |  | DAPK3 |  |
|  |  | DAZAP1 |  |
|  |  | DBH |  |
|  |  | DBNDD1 |  |
|  |  | DBNL |  |
|  |  | DCAF11 |  |
|  |  | DCAF16 |  |
|  |  | DCAKD |  |
|  |  | DCHS1 |  |
|  |  | DCK |  |
|  |  | ST8SIA5 |  |
|  |  | STAC |  |
|  |  | STAC2 |  |
|  |  | STAC3 |  |
|  |  | STARD3 |  |
|  |  | STARD7 |  |
|  |  | STARD9 |  |
|  |  | STAT2 |  |
|  |  | STIM1 |  |
|  |  | STIP1 |  |
|  |  | STK16 |  |
|  |  | STK32C |  |
|  |  | STK35 |  |
|  |  | DENND1A |  |
|  |  | DENND4B |  |
|  |  | DENND4C |  |
|  |  | DEPDC4 |  |
|  |  | DERL1 |  |
|  |  | DERL3 |  |
|  |  | STT3B |  |
|  |  | STUB1 |  |
|  |  | NRBP1 |  |
|  |  | STX1A |  |
|  |  | STX1B |  |
|  |  | NRGN |  |
|  |  | STXBP1 |  |
|  |  | DGKZ |  |
|  |  | DHCR24 |  |
|  |  | DHRS3 |  |
|  |  | NSDHL |  |
|  |  | DHRS4L2 |  |
|  |  | DHRS9 |  |
|  |  | DHX30 |  |
|  |  | DHX34 |  |
|  |  | DHX38 |  |
|  |  | DHX40 |  |
|  |  | NT5C3B |  |
|  |  | DIO3 |  |
|  |  | DIS3L2 |  |
|  |  | SV2A |  |
|  |  | DKC1 |  |
|  |  | DKFZP434O1614 |  |
|  |  | DKFZP761J1410 |  |
|  |  | DKFZP779J2370 |  |
|  |  | DLGAP1 |  |
|  |  | DLL1 |  |
|  |  | DLST |  |
|  |  | DLX2 |  |
|  |  | DLX3 |  |
|  |  | DMAP1 |  |
|  |  | DMBT1 |  |
|  |  | DMPK |  |
|  |  | DMRT2 |  |
|  |  | RPP25 |  |
|  |  | DMTN |  |
|  |  | RPS23 |  |
|  |  | RPS28 |  |
|  |  | RPS6KA2 |  |
|  |  | RPS6KA4 |  |
|  |  | RPS6KB2 |  |
|  |  | TAF1C |  |
|  |  | TAF1L |  |
|  |  | TAGLN2 |  |
|  |  | TAGLN3 |  |
|  |  | TANGO2 |  |
|  |  | TANGO6 |  |
|  |  | TAOK2 |  |
|  |  | CNN2 |  |
|  |  | TARM1 |  |
|  |  | DOCK9 |  |
|  |  | RSL24D1 |  |
|  |  | RSPO1 |  |
|  |  | RSPO4 |  |
|  |  | RTN2 |  |
|  |  | RTN4IP1 |  |
|  |  | RTN4R |  |
|  |  | RTP2 |  |
|  |  | RUFY3 |  |
|  |  | RUNDC3A |  |
|  |  | RWDD2B |  |
|  |  | RXFP3 |  |
|  |  | S100A11 |  |
|  |  | S100A13 |  |
|  |  | S100A2 |  |
|  |  | S100A3 |  |
|  |  | S100A5 |  |
|  |  | S100P |  |
|  |  | S1PR2 |  |
|  |  | SAA1 |  |
|  |  | SAE1 |  |
|  |  | SALL2 |  |
|  |  | SAMD1 |  |
|  |  | SAMD10 |  |
|  |  | SAMD11 |  |
|  |  | SAMD4A |  |
|  |  | SAPCD2 |  |
|  |  | PABPC1L |  |
|  |  | PABPC3 |  |
|  |  | PABPN1 |  |
|  |  | SAYSD1 |  |
|  |  | SBF1 |  |
|  |  | PACSIN3 |  |
|  |  | PADI1 |  |
|  |  | PADI2 |  |
|  |  | PADI3 |  |
|  |  | PAEP |  |
|  |  | PAGR1 |  |
|  |  | SCAMP5 |  |
|  |  | SCARA3 |  |
|  |  | SCARF1 |  |
|  |  | SCFD2 |  |
|  |  | SCGB3A1 |  |
|  |  | SCGN |  |
|  |  | SCLT1 |  |
|  |  | SCMH1 |  |
|  |  | SCNM1 |  |
|  |  | SCNN1D |  |
|  |  | TFCP2 |  |
|  |  | SCP2 |  |
|  |  | SCRN1 |  |
|  |  | SCUBE1 |  |
|  |  | SCUBE2 |  |
|  |  | SDAD1 |  |
|  |  | SDC1 |  |
|  |  | SDCBP2 |  |
|  |  | EFS |  |
|  |  | SDHAF2 |  |
|  |  | SDK1 |  |
|  |  | THAP7 |  |
|  |  | BTN3A2 |  |
|  |  | BTNL2 |  |
|  |  | SEC24C |  |
|  |  | EIF3J |  |
|  |  | EIF3L |  |
|  |  | EIF4E1B |  |
|  |  | SELM |  |
|  |  | SELO |  |
|  |  | SEMA4B |  |
|  |  | SEMA4C |  |
|  |  | SEMA4F |  |
|  |  | SEMA6B |  |
|  |  | EMID1 |  |
|  |  | PCNXL3 |  |
|  |  | SEPT12 |  |
|  |  | SEPT14 |  |
|  |  | SEPT4 |  |
|  |  | SEPT9 |  |
|  |  | SERF1A |  |
|  |  | ENG |  |
|  |  | SERHL2 |  |
|  |  | ENKD1 |  |
|  |  | SERINC4 |  |
|  |  | ENO2 |  |
|  |  | SERP2 |  |
|  |  | SERPINA1 |  |
|  |  | SERPINA4 |  |
|  |  | SERPINB10 |  |
|  |  | SERPINB7 |  |
|  |  | SERPIND1 |  |
|  |  | SERPINF2 |  |
|  |  | SERPING1 |  |
|  |  | SERTAD1 |  |
|  |  | SESN2 |  |
|  |  | SETD1B |  |
|  |  | PDGFRL |  |
|  |  | SEZ6 |  |
|  |  | SEZ6L2 |  |
|  |  | SF1 |  |
|  |  | TMEM108 |  |
|  |  | TMEM110-MUSTN1 | |
|  |  | TMEM120B |  |
|  |  | TMEM121 |  |
|  |  | TMEM127 |  |
|  |  | TMEM132A |  |
|  |  | TMEM132E |  |
|  |  | TMEM14E |  |
|  |  | TMEM150A |  |
|  |  | TMEM151A |  |
|  |  | TMEM151B |  |
|  |  | TMEM156 |  |
|  |  | TMEM161A |  |
|  |  | TMEM173 |  |
|  |  | TMEM176A |  |
|  |  | TMEM176B |  |
|  |  | TMEM18 |  |
|  |  | TMEM184A |  |
|  |  | TMEM187 |  |
|  |  | TMEM194A |  |
|  |  | TMEM198 |  |
|  |  | TMEM200B |  |
|  |  | TMEM202 |  |
|  |  | TMEM207 |  |
|  |  | TMEM209 |  |
|  |  | TMEM214 |  |
|  |  | TMEM216 |  |
|  |  | TMEM221 |  |
|  |  | TMEM225 |  |
|  |  | TMEM229B |  |
|  |  | APOBR |  |
|  |  | TMEM234 |  |
|  |  | TMEM235 |  |
|  |  | RTP4 |  |
|  |  | AQP2 |  |
|  |  | TMEM38A |  |
|  |  | TMEM41A |  |
|  |  | TMEM44 |  |
|  |  | TMEM45B |  |
|  |  | TMEM50A |  |
|  |  | TMEM51 |  |
|  |  | TMEM53 |  |
|  |  | TMEM55B |  |
|  |  | TMEM66 |  |
|  |  | TMEM72 |  |
|  |  | TMEM74B |  |
|  |  | TMEM75 |  |
|  |  | TMEM82 |  |
|  |  | TMEM86B |  |
|  |  | TMEM8A |  |
|  |  | TMEM9 |  |
|  |  | TMEM91 |  |
|  |  | TMIE |  |
|  |  | TMPRSS12 |  |
|  |  | TMPRSS2 |  |
|  |  | TMPRSS6 |  |
|  |  | TMUB1 |  |
|  |  | TNF |  |
|  |  | TNFAIP8L2 |  |
|  |  | TNFAIP8L3 |  |
|  |  | TNFRSF13B |  |
|  |  | TNFRSF14 |  |
|  |  | TNIP1 |  |
|  |  | TNK1 |  |
|  |  | TNK2 |  |
|  |  | TNNT1 |  |
|  |  | TNNT3 |  |
|  |  | TNPO2 |  |
|  |  | TNS4 |  |
|  |  | TP53TG3D |  |
|  |  | TP53TG5 |  |
|  |  | TP73 |  |
|  |  | SEMA4A |  |
|  |  | TPRN |  |
|  |  | SEPT5 |  |
|  |  | TPSAB1 |  |
|  |  | TPSB2 |  |
|  |  | TPST1 |  |
|  |  | TRAF7 |  |
|  |  | TRAPPC10 |  |
|  |  | TRAPPC3 |  |
|  |  | TRAPPC4 |  |
|  |  | TRAPPC6A |  |
|  |  | TRAPPC9 |  |
|  |  | TREH |  |
|  |  | TRIM26 |  |
|  |  | TRIM34 |  |
|  |  | TRIM38 |  |
|  |  | SFTPA1 |  |
|  |  | TRIM58 |  |
|  |  | TRIM62 |  |
|  |  | TRIM66 |  |
|  |  | TRIM67 |  |
|  |  | TRIM68 |  |
|  |  | TRIM6-TRIM34 |  |
|  |  | SGOL2 |  |
|  |  | SGSH |  |
|  |  | DOK7 |  |
|  |  | DOLPP1 |  |
|  |  | TRMT112 |  |
|  |  | TRMU |  |
|  |  | TRNP1 |  |
|  |  | TROAP |  |
|  |  | TRPC3 |  |
|  |  | TRPC4AP |  |
|  |  | TRPC6 |  |
|  |  | TRPM2 |  |
|  |  | TRPV1 |  |
|  |  | TRPV4 |  |
|  |  | TRPV5 |  |
|  |  | TSC22D3 |  |
|  |  | TSEN54 |  |
|  |  | TSFM |  |
|  |  | TSGA13 |  |
|  |  | TSPAN15 |  |
|  |  | TSPAN17 |  |
|  |  | TSPAN18 |  |
|  |  | SIDT2 |  |
|  |  | SIGLEC6 |  |
|  |  | SIGLEC7 |  |
|  |  | TSPEAR |  |
|  |  | TSPO |  |
|  |  | TSPO2 |  |
|  |  | TSPYL6 |  |
|  |  | TSR1 |  |
|  |  | TSR3 |  |
|  |  | TSSK6 |  |
|  |  | TSTA3 |  |
|  |  | TTC13 |  |
|  |  | TTC16 |  |
|  |  | TTC21B |  |
|  |  | TTC24 |  |
|  |  | TTC27 |  |
|  |  | TTC30B |  |
|  |  | TTC38 |  |
|  |  | TTC39C |  |
|  |  | TTC4 |  |
|  |  | TTI1 |  |
|  |  | TTLL1 |  |
|  |  | TTLL11 |  |
|  |  | TTLL12 |  |
|  |  | TTLL4 |  |
|  |  | TTLL6 |  |
|  |  | TTYH1 |  |
|  |  | TUBA4A |  |
|  |  | TUBAL3 |  |
|  |  | TUBB4A |  |
|  |  | TUBG1 |  |
|  |  | TUBGCP2 |  |
|  |  | BOLA3 |  |
|  |  | BORA |  |
|  |  | BPI |  |
|  |  | BPIFA1 |  |
|  |  | BPIFA2 |  |
|  |  | BRAP |  |
|  |  | BRAT1 |  |
|  |  | BRE |  |
|  |  | BRF1 |  |
|  |  | BRF2 |  |
|  |  | BRMS1 |  |
|  |  | BSCL2 |  |
|  |  | BSPRY |  |
|  |  | BST2 |  |
|  |  | BTBD19 |  |
|  |  | BTBD2 |  |
|  |  | BTBD6 |  |
|  |  | BTBD9 |  |
|  |  | BTN1A1 |  |
|  |  | BTN2A1 |  |
|  |  | BTN2A2 |  |
|  |  | BTN3A1 |  |
|  |  | BZRAP1 |  |
|  |  | SLC30A2 |  |
|  |  | C10orf82 |  |
|  |  | C10orf85 |  |
|  |  | C10orf90 |  |
|  |  | ELF2 |  |
|  |  | ELF3 |  |
|  |  | ELF4 |  |
|  |  | ELMOD3 |  |
|  |  | C11orf80 |  |
|  |  | C11orf86 |  |
|  |  | C11orf91 |  |
|  |  | C12orf43 |  |
|  |  | C12orf52 |  |
|  |  | C12orf55 |  |
|  |  | C12orf68 |  |
|  |  | C12orf75 |  |
|  |  | C12orf76 |  |
|  |  | C12orf79 |  |
|  |  | C13orf35 |  |
|  |  | C14orf144 |  |
|  |  | C14orf164 |  |
|  |  | C14orf177 |  |
|  |  | C14orf180 |  |
|  |  | C14orf28 |  |
|  |  | ENGASE |  |
|  |  | ENO1 |  |
|  |  | ENO4 |  |
|  |  | C15orf40 |  |
|  |  | C15orf52 |  |
|  |  | C15orf62 |  |
|  |  | C16orf11 |  |
|  |  | C16orf13 |  |
|  |  | C16orf54 |  |
|  |  | C16orf58 |  |
|  |  | C16orf70 |  |
|  |  | C16orf71 |  |
|  |  | C16orf74 |  |
|  |  | C16orf80 |  |
|  |  | C16orf95 |  |
|  |  | C17orf103 |  |
|  |  | C17orf49 |  |
|  |  | C17orf59 |  |
|  |  | C17orf62 |  |
|  |  | C17orf72 |  |
|  |  | C17orf77 |  |
|  |  | EPHA8 |  |
|  |  | C17orf82 |  |
|  |  | C19orf52 |  |
|  |  | C19orf54 |  |
|  |  | C19orf55 |  |
|  |  | C19orf57 |  |
|  |  | C19orf60 |  |
|  |  | C19orf69 |  |
|  |  | C19orf80 |  |
|  |  | C1orf106 |  |
|  |  | C1orf109 |  |
|  |  | C1orf111 |  |
|  |  | C1orf115 |  |
|  |  | C1orf158 |  |
|  |  | C1orf220 |  |
|  |  | C1orf228 |  |
|  |  | C1orf35 |  |
|  |  | C1orf64 |  |
|  |  | C1QA |  |
|  |  | C1QB |  |
|  |  | C1QL1 |  |
|  |  | C1QL2 |  |
|  |  | C1QTNF1 |  |
|  |  | C1QTNF1-AS1 |  |
|  |  | C1QTNF5 |  |
|  |  | C1QTNF6 |  |
|  |  | C1QTNF8 |  |
|  |  | C2 |  |
|  |  | C20orf144 |  |
|  |  | C20orf196 |  |
|  |  | C20orf26 |  |
|  |  | C20orf27 |  |
|  |  | C21orf128 |  |
|  |  | C21orf2 |  |
|  |  | C21orf33 |  |
|  |  | C21orf59 |  |
|  |  | C21orf90 |  |
|  |  | C22orf24 |  |
|  |  | C22orf34 |  |
|  |  | C22orf39 |  |
|  |  | C22orf46 |  |
|  |  | C2CD2L |  |
|  |  | C2CD3 |  |
|  |  | C2orf53 |  |
|  |  | C2orf57 |  |
|  |  | C2orf71 |  |
|  |  | C2orf76 |  |
|  |  | C2orf81 |  |
|  |  | C2orf82 |  |
|  |  | C3 |  |
|  |  | C3orf14 |  |
|  |  | C3orf18 |  |
|  |  | C3orf56 |  |
|  |  | C3orf72 |  |
|  |  | C3orf79 |  |
|  |  | C4A |  |
|  |  | C4B |  |
|  |  | C4orf17 |  |
|  |  | C5 |  |
|  |  | C5AR1 |  |
|  |  | C5orf49 |  |
|  |  | C5orf55 |  |
|  |  | SNX33 |  |
|  |  | SNX8 |  |
|  |  | SNX9 |  |
|  |  | WDR19 |  |
|  |  | WDR25 |  |
|  |  | WFDC10B |  |
|  |  | WFDC13 |  |
|  |  | WFDC8 |  |
|  |  | WFDC9 |  |
|  |  | WFIKKN1 |  |
|  |  | WFS1 |  |
|  |  | TACR3 |  |
|  |  | TADA2B |  |
|  |  | TADA3 |  |
|  |  | ADAD2 |  |
|  |  | SPIRE2 |  |
|  |  | WRAP73 |  |
|  |  | WRB |  |
|  |  | TAS1R3 |  |
|  |  | TAZ |  |
|  |  | TBC1D13 |  |
|  |  | TBC1D16 |  |
|  |  | TBC1D17 |  |
|  |  | TBC1D2 |  |
|  |  | TBC1D20 |  |
|  |  | TBC1D22A |  |
|  |  | TBC1D22B |  |
|  |  | TBC1D24 |  |
|  |  | TBC1D28 |  |
|  |  | TBC1D3G |  |
|  |  | TBCD |  |
|  |  | TBL1X |  |
|  |  | TBRG4 |  |
|  |  | TBX10 |  |
|  |  | TBX2 |  |
|  |  | TBXAS1 |  |
|  |  | TCAP |  |
|  |  | TCEB2 |  |
|  |  | TCF23 |  |
|  |  | TCF3 |  |
|  |  | TCF7 |  |
|  |  | TCF7L2 |  |
|  |  | TCN2 |  |
|  |  | TCP10L |  |
|  |  | TCP10L2 |  |
|  |  | TCTE1 |  |
|  |  | TCTE3 |  |
|  |  | TCTEX1D1 |  |
|  |  | TDRD9 |  |
|  |  | TDRKH |  |
|  |  | TECPR2 |  |
|  |  | TEFM |  |
|  |  | TEKT1 |  |
|  |  | TELO2 |  |
|  |  | ZBTB8OS |  |
|  |  | TERF2IP |  |
|  |  | TERT |  |
|  |  | TEX19 |  |
|  |  | TEX261 |  |
|  |  | TF |  |
|  |  | TFAP2A |  |
|  |  | TFAP2B |  |
|  |  | TFAP2C |  |
|  |  | TFAP2D |  |
|  |  | TFDP1 |  |
|  |  | TFDP3 |  |
|  |  | TFEB |  |
|  |  | TFF3 |  |
|  |  | TFR2 |  |
|  |  | TGIF2-C20orf24 |  |
|  |  | TGM4 |  |
|  |  | TH |  |
|  |  | THAP3 |  |
|  |  | ZFHX2 |  |
|  |  | THEM6 |  |
|  |  | ALDH3B1 |  |
|  |  | THOC3 |  |
|  |  | ALDH7A1 |  |
|  |  | THRA |  |
|  |  | THRAP3 |  |
|  |  | THTPA |  |
|  |  | TIAF1 |  |
|  |  | TICRR |  |
|  |  | CRYM |  |
|  |  | TIGD5 |  |
|  |  | CSF3 |  |
|  |  | TIMM10 |  |
|  |  | TIMM13 |  |
|  |  | TIMM44 |  |
|  |  | CST4 |  |
|  |  | CST6 |  |
|  |  | CT45A2 |  |
|  |  | CTAGE15 |  |
|  |  | CTAGE4 |  |
|  |  | CTAGE6 |  |
|  |  | CTAGE9 |  |
|  |  | CTC-260F20.3 |  |
|  |  | CTC-435M10.3 |  |
|  |  | CTD-2203A3.1 |  |
|  |  | CTD-2510F5.6 |  |
|  |  | TMC5 |  |
|  |  | TMC6 |  |
|  |  | TMC8 |  |
|  |  | TMCC2 |  |
|  |  | TMED9 |  |
|  |  | TMEM101 |  |
|  |  | TMEM105 |  |
|  |  | CDK3 |  |
|  |  | CDK9 |  |
|  |  | CDKL5 |  |
|  |  | CDKN1C |  |
|  |  | CDPF1 |  |
|  |  | CDRT4 |  |
|  |  | CDSN |  |
|  |  | CDT1 |  |
|  |  | CDX1 |  |
|  |  | CEACAM1 |  |
|  |  | CEACAM16 |  |
|  |  | CEACAM4 |  |
|  |  | CEBPE |  |
|  |  | CELF3 |  |
|  |  | CELSR1 |  |
|  |  | CEMP1 |  |
|  |  | CEND1 |  |
|  |  | CENPI |  |
|  |  | CENPM |  |
|  |  | CENPO |  |
|  |  | CEP164 |  |
|  |  | CEP85 |  |
|  |  | CEP89 |  |
|  |  | CERCAM |  |
|  |  | CERKL |  |
|  |  | CERS1 |  |
|  |  | CES2 |  |
|  |  | CES3 |  |
|  |  | CES4A |  |
|  |  | CETN2 |  |
|  |  | CFD |  |
|  |  | CGA |  |
|  |  | CGN |  |
|  |  | CHAD |  |
|  |  | CHCHD1 |  |
|  |  | CHCHD6 |  |
|  |  | CHD3 |  |
|  |  | CHD4 |  |
|  |  | CHD9 |  |
|  |  | CHEK2 |  |
|  |  | CHFR |  |
|  |  | CHI3L1 |  |
|  |  | CHML |  |
|  |  | CHMP4A |  |
|  |  | CHMP4B |  |
|  |  | CHMP4C |  |
|  |  | CHMP6 |  |
|  |  | CHMP7 |  |
|  |  | CHP1 |  |
|  |  | CHP2 |  |
|  |  | CHPF |  |
|  |  | CHPT1 |  |
|  |  | CHRD |  |
|  |  | THEMIS2 |  |
|  |  | CHRM1 |  |
|  |  | CHRNA1 |  |
|  |  | CHRNA10 |  |
|  |  | THOP1 |  |
|  |  | THRSP |  |
|  |  | CHST14 |  |
|  |  | CHST7 |  |
|  |  | TIE1 |  |
|  |  | CHTF8 |  |
|  |  | CHURC1-FNTB |  |
|  |  | CIB1 |  |
|  |  | CIB2 |  |
|  |  | CIDEC |  |
|  |  | CIITA |  |
|  |  | CILP |  |
|  |  | CISD3 |  |
|  |  | CISH |  |
|  |  | CIT |  |
|  |  | CITED2 |  |
|  |  | CITED4 |  |
|  |  | CKM |  |
|  |  | CKS1B |  |
|  |  | CLCN2 |  |
|  |  | CLDN2 |  |
|  |  | CLDN20 |  |
|  |  | CLDN23 |  |
|  |  | CLDN4 |  |
|  |  | CLDN5 |  |
|  |  | CLDN9 |  |
|  |  | CLEC12A |  |
|  |  | CLEC2B |  |
|  |  | CLEC2D |  |
|  |  | CLEC2L |  |
|  |  | CLEC4M |  |
|  |  | CLGN |  |
|  |  | CLLU1 |  |
|  |  | CLPTM1 |  |
|  |  | CLRN1 |  |
|  |  | TMEM119 |  |
|  |  | CMTR1 |  |
|  |  | CNN1 |  |
|  |  | CNNM1 |  |
|  |  | TMEM158 |  |
|  |  | CNPPD1 |  |
|  |  | CNR2 |  |
|  |  | CNTD2 |  |
|  |  | CNTN2 |  |
|  |  | CNTNAP1 |  |
|  |  | CNTNAP3 |  |
|  |  | CNTNAP3B |  |
|  |  | COASY |  |
|  |  | COBLL1 |  |
|  |  | COG4 |  |
|  |  | COIL |  |
|  |  | TMEM208 |  |
|  |  | RAB3IL1 |  |
|  |  | RAB40A |  |
|  |  | TMEM52 |  |
|  |  | TMEM52B |  |
|  |  | TMEM59L |  |
|  |  | TMEM61 |  |
|  |  | TMEM95 |  |
|  |  | ALDH4A1 |  |
|  |  | ALDH5A1 |  |
|  |  | ALDH9A1 |  |
|  |  | ALDOA |  |
|  |  | ALG1 |  |
|  |  | TNC |  |
|  |  | TNFSF12-TNFSF13 | |
|  |  | TNFSF13 |  |
|  |  | AMICA1 |  |
|  |  | ANHX |  |
|  |  | ANKRD30B |  |
|  |  | ANO9 |  |
|  |  | TRAIP |  |
|  |  | TRAPPC2L |  |
|  |  | TRIM28 |  |
|  |  | TRIM46 |  |
|  |  | TRIM50 |  |
|  |  | TRIM54 |  |
|  |  | TRIM56 |  |
|  |  | ARF3 |  |
|  |  | TRO |  |
|  |  | TRPM6 |  |
|  |  | TSPAN10 |  |
|  |  | TSPAN16 |  |
|  |  | ARHGEF19 |  |
|  |  | TSTD1 |  |
|  |  | TTC7B |  |
|  |  | TTLL3 |  |
|  |  | TUBB6 |  |
|  |  | TUBD1 |  |
|  |  | ASB16 |  |
|  |  | U82695.9 |  |
|  |  | UBASH3A |  |
|  |  | UBB |  |
|  |  | UBBP4 |  |
|  |  | UBN1 |  |
|  |  | UBQLN4 |  |
|  |  | UFD1L |  |
|  |  | ULBP1 |  |
|  |  | ULK1 |  |
|  |  | UNK |  |
|  |  | UPB1 |  |
|  |  | UPK2 |  |
|  |  | B4GALT7 |  |
|  |  | DPF1 |  |
|  |  | DPH2 |  |
|  |  | DPH7 |  |
|  |  | DPM2 |  |
|  |  | DPPA4 |  |
|  |  | DPYD |  |
|  |  | DPYSL4 |  |
|  |  | DRD2 |  |
|  |  | DRG2 |  |
|  |  | DRGX |  |
|  |  | DSCR3 |  |
|  |  | DSCR4 |  |
|  |  | DTX1 |  |
|  |  | DTX3 |  |
|  |  | DTX4 |  |
|  |  | DUOX2 |  |
|  |  | DUOXA2 |  |
|  |  | DUS1L |  |
|  |  | DUSP11 |  |
|  |  | DUSP13 |  |
|  |  | DUSP15 |  |
|  |  | DUSP2 |  |
|  |  | DUSP4 |  |
|  |  | DUXA |  |
|  |  | DVL3 |  |
|  |  | DYNLL1 |  |
|  |  | DZIP1L |  |
|  |  | E2F1 |  |
|  |  | E2F4 |  |
|  |  | EARS2 |  |
|  |  | ECHDC3 |  |
|  |  | ECHS1 |  |
|  |  | ECI1 |  |
|  |  | ECT2L |  |
|  |  | EDAR |  |
|  |  | EDARADD |  |
|  |  | EDF1 |  |
|  |  | EDN2 |  |
|  |  | EEF1G |  |
|  |  | EEFSEC |  |
|  |  | EEPD1 |  |
|  |  | EFCAB4A |  |
|  |  | EFCC1 |  |
|  |  | EFHC1 |  |
|  |  | EFHD2 |  |
|  |  | EFNA3 |  |
|  |  | EFNA4 |  |
|  |  | EGFL7 |  |
|  |  | EGFLAM |  |
|  |  | EHD1 |  |
|  |  | PAX2 |  |
|  |  | EIF2B1 |  |
|  |  | PAX7 |  |
|  |  | PBX1 |  |
|  |  | EIF3B |  |
|  |  | PBX4 |  |
|  |  | PBXIP1 |  |
|  |  | PC |  |
|  |  | PCBD1 |  |
|  |  | EIF4A3 |  |
|  |  | PCBP1 |  |
|  |  | EIF4G1 |  |
|  |  | ELAC2 |  |
|  |  | ELOVL1 |  |
|  |  | C11orf72 |  |
|  |  | EMC10 |  |
|  |  | EMC4 |  |
|  |  | EMG1 |  |
|  |  | EMILIN3 |  |
|  |  | EML5 |  |
|  |  | ENPP7 |  |
|  |  | ENTHD2 |  |
|  |  | ENTPD2 |  |
|  |  | ENTPD8 |  |
|  |  | EPB41L4A-AS2 |  |
|  |  | EPHA2 |  |
|  |  | EPHB1 |  |
|  |  | EPHX3 |  |
|  |  | EPN3 |  |
|  |  | EPOR |  |
|  |  | EPX |  |
|  |  | ERAL1 |  |
|  |  | ERC1 |  |
|  |  | ERCC1 |  |
|  |  | ERCC2 |  |
|  |  | ERGIC1 |  |
|  |  | ERI3 |  |
|  |  | ERV3-1 |  |
|  |  | ESAM |  |
|  |  | ESPN |  |
|  |  | ESPNL |  |
|  |  | ESRRA |  |
|  |  | ESYT1 |  |
|  |  | ETAA1 |  |
|  |  | ETFA |  |
|  |  | ETNK2 |  |
|  |  | ETV3L |  |
|  |  | EVA1A |  |
|  |  | EVC |  |
|  |  | EXO5 |  |
|  |  | EXOC3L2 |  |
|  |  | EXOC7 |  |
|  |  | EXOSC10 |  |
|  |  | EXOSC8 |  |
|  |  | EXT2 |  |
|  |  | EZH1 |  |
|  |  | F10 |  |
|  |  | F2RL2 |  |
|  |  | F3 |  |
|  |  | F8 |  |
|  |  | F8A1 |  |
|  |  | FADD |  |
|  |  | FADS6 |  |
|  |  | FAIM3 |  |
|  |  | FAM101A |  |
|  |  | FAM102A |  |
|  |  | FAM109B |  |
|  |  | FAM110D |  |
|  |  | FAM111B |  |
|  |  | FAM114A2 |  |
|  |  | FAM115A |  |
|  |  | FAM127B |  |
|  |  | FAM129B |  |
|  |  | C6orf118 |  |
|  |  | FAM131C |  |
|  |  | FAM132B |  |
|  |  | FAM136A |  |
|  |  | C7orf26 |  |
|  |  | C7orf31 |  |
|  |  | FAM153C |  |
|  |  | FAM155B |  |
|  |  | FAM156A |  |
|  |  | FAM156B |  |
|  |  | FAM160B2 |  |
|  |  | FAM163B |  |
|  |  | C9orf139 |  |
|  |  | FAM186A |  |
|  |  | FAM188B |  |
|  |  | FAM189A2 |  |
|  |  | FAM189B |  |
|  |  | FAM195A |  |
|  |  | FAM195B |  |
|  |  | FAM19A5 |  |
|  |  | FAM207A |  |
|  |  | FAM210A |  |
|  |  | FAM210B |  |
|  |  | FAM220A |  |
|  |  | FAM230A |  |
|  |  | FAM27E1 |  |
|  |  | FAM27E2 |  |
|  |  | FAM27E3 |  |
|  |  | FAM3D |  |
|  |  | CALCOCO1 |  |
|  |  | FAM46C |  |
|  |  | FAM49A |  |
|  |  | FAM50A |  |
|  |  | FAM50B |  |
|  |  | FAM53A |  |
|  |  | FAM57A |  |
|  |  | FAM57B |  |
|  |  | FAM63A |  |
|  |  | FAM65C |  |
|  |  | FAM71C |  |
|  |  | FAM71F1 |  |
|  |  | FAM73B |  |
|  |  | FAM83A |  |
|  |  | CAMSAP3 |  |
|  |  | CANT1 |  |
|  |  | FAM83G |  |
|  |  | FAM83H |  |
|  |  | FAM86B2 |  |
|  |  | FAM86C1 |  |
|  |  | FAM92B |  |
|  |  | CAPNS1 |  |
|  |  | CAPNS2 |  |
|  |  | CAPS |  |
|  |  | FANCE |  |
|  |  | FANCI |  |
|  |  | FARSA |  |
|  |  | FAS |  |
|  |  | FASTK |  |
|  |  | FATE1 |  |
|  |  | LCE3E |  |
|  |  | FBP1 |  |
|  |  | FBXL12 |  |
|  |  | FBXL15 |  |
|  |  | FBXL18 |  |
|  |  | FBXO17 |  |
|  |  | FBXO2 |  |
|  |  | FBXO22 |  |
|  |  | POU2AF1 |  |
|  |  | FBXO6 |  |
|  |  | FBXW4 |  |
|  |  | FBXW9 |  |
|  |  | FCAMR |  |
|  |  | FCGR3A |  |
|  |  | FCHO1 |  |
|  |  | FCRL1 |  |
|  |  | FCRL4 |  |
|  |  | FDXR |  |
|  |  | LILRA2 |  |
|  |  | FERMT1 |  |
|  |  | FERMT3 |  |
|  |  | FES |  |
|  |  | FFAR1 |  |
|  |  | FFAR2 |  |
|  |  | FFAR3 |  |
|  |  | FFAR4 |  |
|  |  | FGF16 |  |
|  |  | FGF19 |  |
|  |  | FGF22 |  |
|  |  | FGFBP1 |  |
|  |  | FGFR2 |  |
|  |  | FGFR4 |  |
|  |  | FGR |  |
|  |  | CCDC27 |  |
|  |  | CCDC42 |  |
|  |  | SMIM1 |  |
|  |  | SMIM3 |  |
|  |  | SMIM5 |  |
|  |  | SMIM7 |  |
|  |  | PPP2R2B |  |
|  |  | SMURF2 |  |
|  |  | SMYD1 |  |
|  |  | SMYD5 |  |
|  |  | PPT2 |  |
|  |  | SNAPC2 |  |
|  |  | SNAPC5 |  |
|  |  | SNCA |  |
|  |  | PRCD |  |
|  |  | SNPH |  |
|  |  | SNRNP35 |  |
|  |  | SNRPA1 |  |
|  |  | SNTA1 |  |
|  |  | SNX12 |  |
|  |  | SNX17 |  |
|  |  | SNX20 |  |
|  |  | SNX21 |  |
|  |  | SOHLH1 |  |
|  |  | SORCS2 |  |
|  |  | SOX8 |  |
|  |  | SP100 |  |
|  |  | SP5 |  |
|  |  | SPAG6 |  |
|  |  | SPATA19 |  |
|  |  | SPATA2L |  |
|  |  | SPATA33 |  |
|  |  | SPATC1 |  |
|  |  | SPATC1L |  |
|  |  | SPC24 |  |
|  |  | SPCS3 |  |
|  |  | SPDYE1 |  |
|  |  | SPDYE3 |  |
|  |  | SPEF1 |  |
|  |  | SPEM1 |  |
|  |  | SPEN |  |
|  |  | PSD |  |
|  |  | PSMA1 |  |
|  |  | SPIN3 |  |
|  |  | SPINT1 |  |
|  |  | CDC6 |  |
|  |  | SPON2 |  |
|  |  | SPR |  |
|  |  | SPRED3 |  |
|  |  | SPRN |  |
|  |  | SPRR2A |  |
|  |  | PSMG1 |  |
|  |  | SPRR2E |  |
|  |  | SPRR2F |  |
|  |  | SPRR3 |  |
|  |  | SPRY4 |  |
|  |  | SPRYD3 |  |
|  |  | SPSB2 |  |
|  |  | SPSB3 |  |
|  |  | SPSB4 |  |
|  |  | SPZ1 |  |
|  |  | SQSTM1 |  |
|  |  | SRBD1 |  |
|  |  | SRC |  |
|  |  | SRCIN1 |  |
|  |  | SRCRB4D |  |
|  |  | SRGAP2 |  |
|  |  | SRMS |  |
|  |  | SRP68 |  |
|  |  | SRP72 |  |
|  |  | SRPK3 |  |
|  |  | SRRM1 |  |
|  |  | SRRM2 |  |
|  |  | SRRM3 |  |
|  |  | SSBP3 |  |
|  |  | SSBP3-AS1 |  |
|  |  | SSBP4 |  |
|  |  | SSRP1 |  |
|  |  | SSTR3 |  |
|  |  | SSTR5 |  |
|  |  | SSUH2 |  |
|  |  | ST14 |  |
|  |  | ST3GAL1 |  |
|  |  | ST3GAL2 |  |
|  |  | ST3GAL3 |  |
|  |  | ST6GAL1 |  |
|  |  | ST6GAL2 |  |
|  |  | ST6GALNAC1 |  |
|  |  | ST6GALNAC2 |  |
|  |  | AADACL4 |  |
|  |  | AANAT |  |
|  |  | AAR2 |  |
|  |  | AARD |  |
|  |  | AARS |  |
|  |  | AARS2 |  |
|  |  | AATK |  |
|  |  | ABCA2 |  |
|  |  | ABCA3 |  |
|  |  | ABCB8 |  |
|  |  | ABCB9 |  |
|  |  | ABCC12 |  |
|  |  | ABCC4 |  |
|  |  | ABCC6 |  |
|  |  | ABCC9 |  |
|  |  | ABCF2 |  |
|  |  | ABCF3 |  |
|  |  | ABCG4 |  |
|  |  | ABHD16B |  |
|  |  | ABHD4 |  |
|  |  | STOM |  |
|  |  | STOML2 |  |
|  |  | STOML3 |  |
|  |  | STPG1 |  |
|  |  | STRIP2 |  |
|  |  | AC008060.7 |  |
|  |  | AC010327.2 |  |
|  |  | AC010441.1 |  |
|  |  | AC011484.1 |  |
|  |  | AC011755.1 |  |
|  |  | AC011997.1 |  |
|  |  | AC012360.2 |  |
|  |  | AC022498.1 |  |
|  |  | AC073610.5 |  |
|  |  | SUGP1 |  |
|  |  | SULT1A2 |  |
|  |  | SULT1A3 |  |
|  |  | SULT1A4 |  |
|  |  | SUPT16H |  |
|  |  | SUPT4H1 |  |
|  |  | SUPT5H |  |
|  |  | SUPT6H |  |
|  |  | SUSD3 |  |
|  |  | SUV39H1 |  |
|  |  | SUV420H2 |  |
|  |  | ACADM |  |
|  |  | SYCN |  |
|  |  | SYCP2L |  |
|  |  | SYN3 |  |
|  |  | SYNC |  |
|  |  | SYNGR1 |  |
|  |  | SYNPO2L |  |
|  |  | SYNRG |  |
|  |  | SYP |  |
|  |  | SYS1 |  |
|  |  | SYT12 |  |
|  |  | SYT17 |  |
|  |  | ACSS1 |  |
|  |  | ACTA1 |  |
|  |  | ACTBL2 |  |
|  |  | ACTN2 |  |
|  |  | ACTR1B |  |
|  |  | ACTR3C |  |
|  |  | ACTR8 |  |
|  |  | ADAM11 |  |
|  |  | ADAM15 |  |
|  |  | ADAM7 |  |
|  |  | ADAM8 |  |
|  |  | ADAMDEC1 |  |
|  |  | ADAMTS14 |  |
|  |  | ADAMTS2 |  |
|  |  | ADAMTS7 |  |
|  |  | ADAMTS8 |  |
|  |  | ADAMTSL1 |  |
|  |  | ADAMTSL2 |  |
|  |  | ADAMTSL5 |  |
|  |  | ADAP2 |  |
|  |  | ADARB1 |  |
|  |  | ADCK4 |  |
|  |  | ADCY3 |  |
|  |  | ADCY7 |  |
|  |  | ADGB |  |
|  |  | ADH6 |  |
|  |  | ADIPOR1 |  |
|  |  | ADIPOR2 |  |
|  |  | ADIRF |  |
|  |  | ADM2 |  |
|  |  | ADORA1 |  |
|  |  | ADORA2A |  |
|  |  | ADORA3 |  |
|  |  | ADPRH |  |
|  |  | ADPRHL1 |  |
|  |  | ADPRHL2 |  |
|  |  | ADPRM |  |
|  |  | ADRA1D |  |
|  |  | ADRBK1 |  |
|  |  | ADSS |  |
|  |  | ADTRP |  |
|  |  | AEBP1 |  |
|  |  | AFMID |  |
|  |  | AGAP2 |  |
|  |  | AGFG2 |  |
|  |  | AGPAT1 |  |
|  |  | AGT |  |
|  |  | AGTRAP |  |
|  |  | AHCY |  |
|  |  | AHDC1 |  |
|  |  | AHI1 |  |
|  |  | AHNAK |  |
|  |  | AHNAK2 |  |
|  |  | AHSA1 |  |
|  |  | AIFM1 |  |
|  |  | AIP |  |
|  |  | AK1 |  |
|  |  | AK8 |  |
|  |  | AKAP9 |  |
|  |  | AKNA |  |
|  |  | AKNAD1 |  |
|  |  | AKR1A1 |  |
|  |  | AKR1B1 |  |
|  |  | AKR1B10 |  |
|  |  | AKR1B15 |  |
|  |  | AKR1C2 |  |
|  |  | AKR1C4 |  |
|  |  | AKR1E2 |  |
|  |  | AKT1 |  |
|  |  | AL020996.1 |  |
|  |  | AL031666.2 |  |
|  |  | AL117190.3 |  |
|  |  | AL136531.1 |  |
|  |  | AL163636.6 |  |
|  |  | AL353791.1 |  |
|  |  | AL354898.1 |  |
|  |  | AL355390.1 |  |
|  |  | AL391421.1 |  |
|  |  | AL450307.1 |  |
|  |  | AL589765.1 |  |
|  |  | AL590822.1 |  |
|  |  | AL590822.2 |  |
|  |  | AL592284.1 |  |
|  |  | AL627171.2 |  |
|  |  | AL627309.1 |  |
|  |  | AL645730.2 |  |
|  |  | AL953854.2 |  |
|  |  | ALDH3A1 |  |
|  |  | CRNN |  |
|  |  | CRYBB2 |  |
|  |  | ALG11 |  |
|  |  | ALG12 |  |
|  |  | ALK |  |
|  |  | ALKBH4 |  |
|  |  | ALKBH6 |  |
|  |  | ALOX12 |  |
|  |  | ALOXE3 |  |
|  |  | ALPK2 |  |
|  |  | ALPL |  |
|  |  | ALPP |  |
|  |  | ALPPL2 |  |
|  |  | ALS2CL |  |
|  |  | AMBP |  |
|  |  | AMHR2 |  |
|  |  | AMN |  |
|  |  | AMOTL2 |  |
|  |  | AMPD2 |  |
|  |  | AMT |  |
|  |  | AMZ1 |  |
|  |  | ANAPC11 |  |
|  |  | ANAPC2 |  |
|  |  | ANKHD1 |  |
|  |  | ANKRD1 |  |
|  |  | ANKRD13D |  |
|  |  | ANKRD18A |  |
|  |  | ANKRD23 |  |
|  |  | ANKRD26 |  |
|  |  | ANKRD39 |  |
|  |  | ANKRD42 |  |
|  |  | ANKRD54 |  |
|  |  | ANKRD65 |  |
|  |  | ANKS4B |  |
|  |  | ANKS6 |  |
|  |  | ANO10 |  |
|  |  | ANP32A |  |
|  |  | ANXA5 |  |
|  |  | ANXA6 |  |
|  |  | AOAH |  |
|  |  | AOC3 |  |
|  |  | AP000695.1 |  |
|  |  | AP000769.1 |  |
|  |  | AP000867.1 |  |
|  |  | AP001579.1 |  |
|  |  | AP003068.23 |  |
|  |  | AP006621.5 |  |
|  |  | AP1B1 |  |
|  |  | AP1M1 |  |
|  |  | AP1M2 |  |
|  |  | AP2A2 |  |
|  |  | AP5S1 |  |
|  |  | AP5Z1 |  |
|  |  | APC2 |  |
|  |  | APCDD1 |  |
|  |  | APCDD1L |  |
|  |  | APEH |  |
|  |  | APH1A |  |
|  |  | APITD1-CORT |  |
|  |  | APLNR |  |
|  |  | APMAP |  |
|  |  | APOA1 |  |
|  |  | APOA4 |  |
|  |  | APOA5 |  |
|  |  | APOBEC2 |  |
|  |  | APOBEC3H |  |
|  |  | APOC4 |  |
|  |  | APOD |  |
|  |  | APOE |  |
|  |  | APOL3 |  |
|  |  | APOL4 |  |
|  |  | AQP6 |  |
|  |  | AQP8 |  |
|  |  | AQPEP |  |
|  |  | ARAP1 |  |
|  |  | ARF4 |  |
|  |  | ARF5 |  |
|  |  | ARFGAP1 |  |
|  |  | ARFGAP2 |  |
|  |  | ARFGEF2 |  |
|  |  | ARFRP1 |  |
|  |  | ARHGAP10 |  |
|  |  | DCTPP1 |  |
|  |  | ARHGAP25 |  |
|  |  | ARHGAP35 |  |
|  |  | ARHGAP8 |  |
|  |  | ARHGDIA |  |
|  |  | ARHGDIG |  |
|  |  | ARHGEF17 |  |
|  |  | ARHGEF37 |  |
|  |  | ARID3A |  |
|  |  | ARIH1 |  |
|  |  | ARIH2 |  |
|  |  | ARL13A |  |
|  |  | ARL2 |  |
|  |  | ARL6IP4 |  |
|  |  | ARMC5 |  |
|  |  | ARMC6 |  |
|  |  | ARMC7 |  |
|  |  | ARMCX6 |  |
|  |  | ARPC4-TTLL3 |  |
|  |  | ARPC5L |  |
|  |  | ARRDC1 |  |
|  |  | ARRDC2 |  |
|  |  | ARSA |  |
|  |  | ARSI |  |
|  |  | ART1 |  |
|  |  | ARVCF |  |
|  |  | AS3MT |  |
|  |  | ASB13 |  |
|  |  | ASB2 |  |
|  |  | ASB8 |  |
|  |  | ASCL5 |  |
|  |  | ASF1B |  |
|  |  | ASIC2 |  |
|  |  | ASPG |  |
|  |  | ASPSCR1 |  |
|  |  | ASRGL1 |  |
|  |  | ASTN2 |  |
|  |  | ATAD3C |  |
|  |  | ATCAY |  |
|  |  | ATG13 |  |
|  |  | ATG16L1 |  |
|  |  | ATG2A |  |
|  |  | ATG4B |  |
|  |  | ATG9A |  |
|  |  | ATG9B |  |
|  |  | ATHL1 |  |
|  |  | ATOH1 |  |
|  |  | ATP10B |  |
|  |  | ATP10D |  |
|  |  | ATP13A2 |  |
|  |  | ATP1A4 |  |
|  |  | ATP2A3 |  |
|  |  | ATP2B4 |  |
|  |  | ATP5D |  |
|  |  | ATP5E |  |
|  |  | ATP6AP1 |  |
|  |  | ATP6V0C |  |
|  |  | ATP6V1E1 |  |
|  |  | ATPAF2 |  |
|  |  | ATPIF1 |  |
|  |  | ATXN10 |  |
|  |  | AURKAIP1 |  |
|  |  | AVPR1B |  |
|  |  | AVPR2 |  |
|  |  | B3GAT1 |  |
|  |  | B3GNT4 |  |
|  |  | B3GNT7 |  |
|  |  | B4GALT2 |  |
|  |  | B4GALT5 |  |
|  |  | BACE1 |  |
|  |  | BAG3 |  |
|  |  | BAG5 |  |
|  |  | BAHD1 |  |
|  |  | BAI1 |  |
|  |  | BAI2 |  |
|  |  | BAI3 |  |
|  |  | BAIAP2 |  |
|  |  | BAIAP3 |  |
|  |  | BANK1 |  |
|  |  | BANP |  |
|  |  | BATF3 |  |
|  |  | BCAM |  |
|  |  | BCAR1 |  |
|  |  | BCAS1 |  |
|  |  | BCAT2 |  |
|  |  | BCDIN3D |  |
|  |  | BCKDK |  |
|  |  | BCL2L1 |  |
|  |  | BCL2L10 |  |
|  |  | BCL2L2-PABPN1 |  |
|  |  | BCL7B |  |
|  |  | BDH1 |  |
|  |  | BDH2 |  |
|  |  | BDKRB2 |  |
|  |  | BEST4 |  |
|  |  | BFSP2 |  |
|  |  | BHLHA15 |  |
|  |  | BHLHE22 |  |
|  |  | BHLHE40 |  |
|  |  | BHMT2 |  |
|  |  | BID |  |
|  |  | BIK |  |
|  |  | BIN3 |  |
|  |  | BIRC5 |  |
|  |  | BLID |  |
|  |  | BLMH |  |
|  |  | BLOC1S3 |  |
|  |  | BMP2 |  |
|  |  | BNIPL |  |
|  |  | EEF1D |  |
|  |  | EFCAB13 |  |
|  |  | EFCAB4B |  |
|  |  | EFNA1 |  |
|  |  | EFTUD2 |  |
|  |  | EHBP1L1 |  |
|  |  | EIF1AD |  |
|  |  | EIF3G |  |
|  |  | ELAVL3 |  |
|  |  | ELAVL4 |  |
|  |  | KIF18B |  |
|  |  | KIF19 |  |
|  |  | KIF23 |  |
|  |  | KIF24 |  |
|  |  | KIR2DL4 |  |
|  |  | KLC4 |  |
|  |  | ENTHD1 |  |
|  |  | ENTPD3 |  |
|  |  | KLHDC8B |  |
|  |  | EPHA1 |  |
|  |  | EPPK1 |  |
|  |  | KLKB1 |  |
|  |  | EPS8L3 |  |
|  |  | ERF |  |
|  |  | ERP29 |  |
|  |  | ESRP2 |  |
|  |  | ESRRB |  |
|  |  | EVI2A |  |
|  |  | EXOC4 |  |
|  |  | LAGE3 |  |
|  |  | EXOSC5 |  |
|  |  | EXTL2 |  |
|  |  | LAS1L |  |
|  |  | EYA2 |  |
|  |  | LCMT1 |  |
|  |  | LCN10 |  |
|  |  | C6orf123 |  |
|  |  | C6orf136 |  |
|  |  | C6orf203 |  |
|  |  | C6orf223 |  |
|  |  | C7orf43 |  |
|  |  | C7orf50 |  |
|  |  | C7orf57 |  |
|  |  | C8orf22 |  |
|  |  | C8orf34 |  |
|  |  | C8orf49 |  |
|  |  | C8orf58 |  |
|  |  | C8orf86 |  |
|  |  | C9orf152 |  |
|  |  | C9orf169 |  |
|  |  | C9orf170 |  |
|  |  | C9orf171 |  |
|  |  | C9orf3 |  |
|  |  | C9orf62 |  |
|  |  | C9orf78 |  |
|  |  | C9orf96 |  |
|  |  | CA7 |  |
|  |  | CABLES1 |  |
|  |  | CABP1 |  |
|  |  | SPIN2A |  |
|  |  | SPINT2 |  |
|  |  | SPINT3 |  |
|  |  | SPNS3 |  |
|  |  | SPRR1A |  |
|  |  | YDJC |  |
|  |  | YIPF2 |  |
|  |  | YIPF3 |  |
|  |  | YIPF7 |  |
|  |  | ZBTB4 |  |
|  |  | ZCCHC17 |  |
|  |  | CCDC53 |  |
|  |  | ZDHHC5 |  |
|  |  | ZFAND1 |  |
|  |  | ZFAND2A |  |
|  |  | ZFAND6 |  |
|  |  | CCER1 |  |
|  |  | CCL1 |  |
|  |  | CCL11 |  |
|  |  | CCL13 |  |
|  |  | CCL22 |  |
|  |  | CCL24 |  |
|  |  | CCL25 |  |
|  |  | CCL28 |  |
|  |  | CCM2L |  |
|  |  | ZGLP1 |  |
|  |  | ZMAT1 |  |
|  |  | ZMPSTE24 |  |
|  |  | ZNF131 |  |
|  |  | ZNF136 |  |
|  |  | ZNF14 |  |
|  |  | ZNF16 |  |
|  |  | ZNF211 |  |
|  |  | ZNF223 |  |
|  |  | ZNF304 |  |
|  |  | ZNF320 |  |
|  |  | CDCA5 |  |
|  |  | CDCA8 |  |
|  |  | CDH12 |  |
|  |  | CDH15 |  |
|  |  | CDH16 |  |
|  |  | CDH22 |  |
|  |  | CDH23 |  |
|  |  | CDH24 |  |
|  |  | CDH3 |  |
|  |  | CDHR2 |  |
|  |  | CDHR3 |  |
|  |  | CDHR4 |  |
|  |  | CDIP1 |  |
|  |  | CDK10 |  |
|  |  | CDK16 |  |
|  |  | CDK2 |  |
|  |  | CDK2AP1 |  |
|  |  | A1BG |  |
|  |  | A4GALT |  |
|  |  | AATF |  |
|  |  | ABCC10 |  |
|  |  | ABCD4 |  |
|  |  | ABCG5 |  |
|  |  | ABHD11 |  |
|  |  | ABHD12B |  |
|  |  | ABI3 |  |
|  |  | ABR |  |
|  |  | AC002472.13 |  |
|  |  | AC003102.1 |  |
|  |  | AC005481.5 |  |
|  |  | AC005609.1 |  |
|  |  | AC006486.1 |  |
|  |  | AC006946.15 |  |
|  |  | AC007952.5 |  |
|  |  | AC007952.6 |  |
|  |  | AC008394.1 |  |
|  |  | AC010536.1 |  |
|  |  | AC013269.5 |  |
|  |  | AC040160.1 |  |
|  |  | AC069547.2 |  |
|  |  | AC074212.3 |  |
|  |  | AC079354.1 |  |
|  |  | AC091801.1 |  |
|  |  | AC092850.1 |  |
|  |  | AC093802.1 |  |
|  |  | AC102948.2 |  |
|  |  | AC104841.2 |  |
|  |  | AC105020.1 |  |
|  |  | AC106017.1 |  |
|  |  | AC112693.2 |  |
|  |  | AC112715.2 |  |
|  |  | AC129492.6 |  |
|  |  | AC138655.1 |  |
|  |  | AC139100.2 |  |
|  |  | AC144568.2 |  |
|  |  | AC174470.1 |  |
|  |  | ACAA1 |  |
|  |  | ACAA2 |  |
|  |  | ACACB |  |
|  |  | ACADS |  |
|  |  | ACAP1 |  |
|  |  | ACAT1 |  |
|  |  | ACBD4 |  |
|  |  | ACCS |  |
|  |  | ACCSL |  |
|  |  | ACE |  |
|  |  | ACHE |  |
|  |  | ACIN1 |  |
|  |  | ACKR2 |  |
|  |  | ACOT2 |  |
|  |  | ACOX3 |  |
|  |  | ACP5 |  |
|  |  | ACRBP |  |
|  |  | ACSBG2 |  |
|  |  | ACSF3 |  |
|  |  | ADAM17 |  |
|  |  | ADAMTS9 |  |
|  |  | ADCK3 |  |
|  |  | ADM5 |  |
|  |  | ADRA2C |  |
|  |  | ADRB3 |  |
|  |  | AGAP10 |  |
|  |  | AHSG |  |
|  |  | AIF1L |  |
|  |  | AIM1L |  |
|  |  | AIPL1 |  |
|  |  | AKAP17A |  |
|  |  | AKAP5 |  |
|  |  | ALAD |  |
|  |  | MYBPC3 |  |
|  |  | MYBPH |  |
|  |  | MYCL |  |
|  |  | ALKBH7 |  |
|  |  | ALMS1 |  |
|  |  | ALOX5 |  |
|  |  | ALX3 |  |
|  |  | AMBRA1 |  |
|  |  | MYPOP |  |
|  |  | AMZ2 |  |
|  |  | NAA60 |  |
|  |  | NAALADL1 |  |
|  |  | NADK |  |
|  |  | NAGPA |  |
|  |  | ANKLE1 |  |
|  |  | NBPF9 |  |
|  |  | NCAM1 |  |
|  |  | ANO7 |  |
|  |  | ANTXRL |  |
|  |  | AP2S1 |  |
|  |  | AQP12A |  |
|  |  | NHLH1 |  |
|  |  | ARHGAP44 |  |
|  |  | ARHGDIB |  |
|  |  | ARHGEF10L |  |
|  |  | NME3 |  |
|  |  | ARL11 |  |
|  |  | ARL8A |  |
|  |  | ARTN |  |
|  |  | NR2C2 |  |
|  |  | ASCL2 |  |
|  |  | ASGR1 |  |
|  |  | ASGR2 |  |
|  |  | ASPRV1 |  |
|  |  | NUAK1 |  |
|  |  | ATP2C2 |  |
|  |  | DMRTB1 |  |
|  |  | DMRTC2 |  |
|  |  | DMWD |  |
|  |  | DNAH10 |  |
|  |  | DNAH17 |  |
|  |  | DNAH17-AS1 |  |
|  |  | DNAJA3 |  |
|  |  | DNAJB12 |  |
|  |  | DNAJB2 |  |
|  |  | DNAJB5 |  |
|  |  | DNAJB6 |  |
|  |  | DNAJC14 |  |
|  |  | DNAJC30 |  |
|  |  | DNAJC5 |  |
|  |  | DNAJC8 |  |
|  |  | DNASE1L1 |  |
|  |  | DNM1 |  |
|  |  | DNPEP |  |
|  |  | OMP |  |
|  |  | ONECUT3 |  |
|  |  | OPN1MW |  |
|  |  | OPN1MW2 |  |
|  |  | OPN1SW |  |
|  |  | OPN5 |  |
|  |  | OPRD1 |  |
|  |  | OR1L8 |  |
|  |  | OR4D5 |  |
|  |  | OR4N2 |  |
|  |  | OR52W1 |  |
|  |  | ORM1 |  |
|  |  | ORM2 |  |
|  |  | INHBC |  |
|  |  | INMT |  |
|  |  | OSBPL7 |  |
|  |  | OSCAR |  |
|  |  | OSER1 |  |
|  |  | OSGIN1 |  |
|  |  | OSR1 |  |
|  |  | OSTC |  |
|  |  | OTOF |  |
|  |  | OTOP2 |  |
|  |  | OTOP3 |  |
|  |  | OTP |  |
|  |  | OTUB1 |  |
|  |  | OTX1 |  |
|  |  | OVOL1 |  |
|  |  | OXLD1 |  |
|  |  | OXSR1 |  |
|  |  | P2RX6 |  |
|  |  | P2RY10 |  |
|  |  | P2RY8 |  |
|  |  | P4HA3 |  |
|  |  | IQSEC3 |  |
|  |  | IRAK1 |  |
|  |  | IRF4 |  |
|  |  | IRF5 |  |
|  |  | ISCA2 |  |
|  |  | PALM |  |
|  |  | PAN2 |  |
|  |  | PAN3 |  |
|  |  | PAPL |  |
|  |  | PAPLN |  |
|  |  | PAPSS2 |  |
|  |  | PARK2 |  |
|  |  | PARM1 |  |
|  |  | PARP12 |  |
|  |  | PARP16 |  |
|  |  | PARVB |  |
|  |  | PARVG |  |
|  |  | PATE3 |  |
|  |  | PATE4 |  |
|  |  | PBOV1 |  |
|  |  | PBX2 |  |
|  |  | PCBP2 |  |
|  |  | PCBP4 |  |
|  |  | KANK1 |  |
|  |  | KAZALD1 |  |
|  |  | PCDHB16 |  |
|  |  | KCNAB3 |  |
|  |  | KCNC2 |  |
|  |  | PCOLCE2 |  |
|  |  | PCSK4 |  |
|  |  | PCSK9 |  |
|  |  | PCTP |  |
|  |  | PCYOX1L |  |
|  |  | PCYT2 |  |
|  |  | PDCD1 |  |
|  |  | PDCD11 |  |
|  |  | PDE1B |  |
|  |  | PDE4C |  |
|  |  | PDE6G |  |
|  |  | KCNK13 |  |
|  |  | PDF |  |
|  |  | PDGFB |  |
|  |  | KCNK6 |  |
|  |  | PDHA1 |  |
|  |  | PDIA3 |  |
|  |  | PDIK1L |  |
|  |  | PDK2 |  |
|  |  | PDLIM1 |  |
|  |  | PDLIM4 |  |
|  |  | PDPN |  |
|  |  | PDX1 |  |
|  |  | PDXP |  |
|  |  | PDZD4 |  |
|  |  | PELI3 |  |
|  |  | PELP1 |  |
|  |  | PEMT |  |
|  |  | PEPD |  |
|  |  | PER1 |  |
|  |  | PES1 |  |
|  |  | PEX6 |  |
|  |  | PFAS |  |
|  |  | PFKFB4 |  |
|  |  | PFKL |  |
|  |  | PGLS |  |
|  |  | PGLYRP4 |  |
|  |  | PGM1 |  |
|  |  | PGM5 |  |
|  |  | PGS1 |  |
|  |  | PHB |  |
|  |  | PHB2 |  |
|  |  | PHC2 |  |
|  |  | PHF13 |  |
|  |  | PHF17 |  |
|  |  | PHF2 |  |
|  |  | PHF23 |  |
|  |  | PHKA2 |  |
|  |  | PHLDB1 |  |
|  |  | PHOX2A |  |
|  |  | PHOX2B |  |
|  |  | PHPT1 |  |
|  |  | PI3 |  |
|  |  | PI4K2A |  |
|  |  | PICK1 |  |
|  |  | PID1 |  |
|  |  | PIGC |  |
|  |  | PIGL |  |
|  |  | PIGQ |  |
|  |  | PIGS |  |
|  |  | PIGZ |  |
|  |  | PIK3IP1 |  |
|  |  | PIK3R2 |  |
|  |  | PIK3R5 |  |
|  |  | PILRB |  |
|  |  | PIN1 |  |
|  |  | PIN4 |  |
|  |  | PINK1 |  |
|  |  | PINX1 |  |
|  |  | PISD |  |
|  |  | PKD1 |  |
|  |  | PKDCC |  |
|  |  | PKDREJ |  |
|  |  | PKN3 |  |
|  |  | PKNOX2 |  |
|  |  | PKP3 |  |
|  |  | PLA2G2F |  |
|  |  | PLA2G3 |  |
|  |  | PLA2G4B |  |
|  |  | PLA2G5 |  |
|  |  | PLAC1 |  |
|  |  | PLAC9 |  |
|  |  | PLAGL2 |  |
|  |  | PLB1 |  |
|  |  | PLCXD2 |  |
|  |  | PLCZ1 |  |
|  |  | PLD2 |  |
|  |  | PLEC |  |
|  |  | PLEKHA4 |  |
|  |  | PLEKHB1 |  |
|  |  | PLEKHF1 |  |
|  |  | PLEKHG3 |  |
|  |  | PLEKHG4 |  |
|  |  | PLEKHM1 |  |
|  |  | PLEKHM2 |  |
|  |  | PLGRKT |  |
|  |  | PLIN4 |  |
|  |  | PLIN5 |  |
|  |  | PLSCR4 |  |
|  |  | PLXDC1 |  |
|  |  | KRTAP5-6 |  |
|  |  | KRTAP9-3 |  |
|  |  | KRTAP9-4 |  |
|  |  | KRTAP9-9 |  |
|  |  | PMF1 |  |
|  |  | PMS2 |  |
|  |  | PNCK |  |
|  |  | PNMA3 |  |
|  |  | PNMAL2 |  |
|  |  | PNPLA2 |  |
|  |  | PNPLA3 |  |
|  |  | PNPLA5 |  |
|  |  | PNPLA6 |  |
|  |  | PODN |  |
|  |  | PODNL1 |  |
|  |  | POFUT2 |  |
|  |  | POLA2 |  |
|  |  | POLD4 |  |
|  |  | POLDIP3 |  |
|  |  | POLL |  |
|  |  | LBX1 |  |
|  |  | LCAT |  |
|  |  | LCE1C |  |
|  |  | LCE1E |  |
|  |  | LCE2A |  |
|  |  | LCE2C |  |
|  |  | LCN1 |  |
|  |  | POLR3C |  |
|  |  | POLR3H |  |
|  |  | POM121 |  |
|  |  | POM121L12 |  |
|  |  | POM121L7 |  |
|  |  | POMC |  |
|  |  | POMT1 |  |
|  |  | PON3 |  |
|  |  | LECT2 |  |
|  |  | POP4 |  |
|  |  | POP5 |  |
|  |  | LEKR1 |  |
|  |  | LENG8 |  |
|  |  | LEP |  |
|  |  | POTEM |  |
|  |  | CBLN4 |  |
|  |  | POU2F3 |  |
|  |  | LETM1 |  |
|  |  | POU5F1B |  |
|  |  | CBX2 |  |
|  |  | PP13004 |  |
|  |  | PP13439 |  |
|  |  | PPAN-P2RY11 |  |
|  |  | PPAP2C |  |
|  |  | PPAPDC1A |  |
|  |  | LHX3 |  |
|  |  | LIF |  |
|  |  | CCDC114 |  |
|  |  | CCDC115 |  |
|  |  | LILRA4 |  |
|  |  | LILRA6 |  |
|  |  | LILRB3 |  |
|  |  | PPM1M |  |
|  |  | PPP1R10 |  |
|  |  | LLGL1 |  |
|  |  | LLGL2 |  |
|  |  | PPP1R26 |  |
|  |  | PPP1R27 |  |
|  |  | PPP1R32 |  |
|  |  | PPP1R37 |  |
|  |  | PPP1R9B |  |
|  |  | PPP2CB |  |
|  |  | CCDC79 |  |
|  |  | CCDC81 |  |
|  |  | CCDC94 |  |
|  |  | PPP6R2 |  |
|  |  | PPT1 |  |
|  |  | LPCAT3 |  |
|  |  | PQLC2 |  |
|  |  | CCL26 |  |
|  |  | PRDM12 |  |
|  |  | PRDM4 |  |
|  |  | PRDX2 |  |
|  |  | PRDX6 |  |
|  |  | PRELP |  |
|  |  | PREX1 |  |
|  |  | PRG2 |  |
|  |  | PRICKLE4 |  |
|  |  | PRIM1 |  |
|  |  | PRKAG3 |  |
|  |  | PRKAR1B |  |
|  |  | LRRC16B |  |
|  |  | PRKCSH |  |
|  |  | PRKCZ |  |
|  |  | CD200 |  |
|  |  | CD207 |  |
|  |  | PRMT7 |  |
|  |  | PROB1 |  |
|  |  | PROC |  |
|  |  | PRODH |  |
|  |  | PROK1 |  |
|  |  | PROM2 |  |
|  |  | PROP1 |  |
|  |  | PROSC |  |
|  |  | PRPF38A |  |
|  |  | PRPF4 |  |
|  |  | PRPF6 |  |
|  |  | PRPS1 |  |
|  |  | PRPS2 |  |
|  |  | PRR13 |  |
|  |  | PRR23C |  |
|  |  | PRR26 |  |
|  |  | PRRT2 |  |
|  |  | PRRX2 |  |
|  |  | PRSS27 |  |
|  |  | PRSS42 |  |
|  |  | PRSS45 |  |
|  |  | PRSS46 |  |
|  |  | PRSS56 |  |
|  |  | PRSS8 |  |
|  |  | PRX |  |
|  |  | PSAP |  |
|  |  | LYPD1 |  |
|  |  | LYPD2 |  |
|  |  | LYPLA2 |  |
|  |  | PSMA4 |  |
|  |  | PSMA7 |  |
|  |  | PSMB7 |  |
|  |  | PSMC3IP |  |
|  |  | PSMC4 |  |
|  |  | PSMD10 |  |
|  |  | PSMD13 |  |
|  |  | PSMD2 |  |
|  |  | PSMD5 |  |
|  |  | PSME4 |  |
|  |  | PTAFR |  |
|  |  | PTCHD2 |  |
|  |  | PTDSS2 |  |
|  |  | PTGDR2 |  |
|  |  | PTGES |  |
|  |  | PTGES2 |  |
|  |  | PTGFRN |  |
|  |  | PTGIR |  |
|  |  | PTGR1 |  |
|  |  | PTH1R |  |
|  |  | PTK2B |  |
|  |  | PTK6 |  |
|  |  | PTK7 |  |
|  |  | PTMS |  |
|  |  | PTP4A3 |  |
|  |  | PTPN18 |  |
|  |  | PTPN23 |  |
|  |  | PTPRN |  |
|  |  | PTPRU |  |
|  |  | PTRF |  |
|  |  | PUM1 |  |
|  |  | PUS1 |  |
|  |  | PVALB |  |
|  |  | PVRIG |  |
|  |  | PVRL4 |  |
|  |  | PWP2 |  |
|  |  | PXDC1 |  |
|  |  | PXDN |  |
|  |  | PXK |  |
|  |  | PYCARD |  |
|  |  | PYCR1 |  |
|  |  | PYGB |  |
|  |  | PYY |  |
|  |  | QDPR |  |
|  |  | QPRT |  |
|  |  | QRICH1 |  |
|  |  | QRSL1 |  |
|  |  | R3HDM4 |  |
|  |  | RAB19 |  |
|  |  | RAB1B |  |
|  |  | RAB24 |  |
|  |  | RAB26 |  |
|  |  | RAB33A |  |
|  |  | RAB34 |  |
|  |  | RAB3D |  |
|  |  | RAB40B |  |
|  |  | RAB40C |  |
|  |  | RAB43 |  |
|  |  | ABHD6 |  |
|  |  | ABL1 |  |
|  |  | AC004466.1 |  |
|  |  | AC005606.1 |  |
|  |  | RAD9A |  |
|  |  | AC019294.1 |  |
|  |  | AC090616.2 |  |
|  |  | AC106876.2 |  |
|  |  | AC124890.1 |  |
|  |  | AC132186.1 |  |
|  |  | AC145676.2 |  |
|  |  | ACOT11 |  |
|  |  | ACPT |  |
|  |  | CLPSL1 |  |
|  |  | CLPSL2 |  |
|  |  | CLSTN3 |  |
|  |  | CMA1 |  |
|  |  | CNOT10 |  |
|  |  | CNTFR |  |
|  |  | COL1A2 |  |
|  |  | COL7A1 |  |
|  |  | COLEC11 |  |
|  |  | COMMD3 |  |
|  |  | COMMD7 |  |
|  |  | COPS5 |  |
|  |  | COPZ1 |  |
|  |  | COQ5 |  |
|  |  | COQ7 |  |
|  |  | CORO1B |  |
|  |  | CORO6 |  |
|  |  | CP |  |
|  |  | CPN1 |  |
|  |  | CPSF1 |  |
|  |  | CPXCR1 |  |
|  |  | CR769776.1 |  |
|  |  | HGFAC |  |
|  |  | HGS |  |
|  |  | HHAT |  |
|  |  | HHIPL1 |  |
|  |  | CSF1R |  |
|  |  | CSF3R |  |
|  |  | CSK |  |
|  |  | HIPK4 |  |
|  |  | HIRA |  |
|  |  | HIRIP3 |  |
|  |  | HIST1H2AG |  |
|  |  | HIST1H2BD |  |
|  |  | HIST1H2BN |  |
|  |  | HIST1H3A |  |
|  |  | HIST1H4K |  |
|  |  | HIST2H2BE |  |
|  |  | HIST2H3A |  |
|  |  | HIST2H3C |  |
|  |  | HIST2H4B |  |
|  |  | HIST3H2A |  |
|  |  | HIVEP2 |  |
|  |  | HK1 |  |
|  |  | HK3 |  |
|  |  | HKR1 |  |
|  |  | HLA-DOB |  |
|  |  | HLA-DPB1 |  |
|  |  | HLA-DQB2 |  |
|  |  | HLA-DRB5 |  |
|  |  | HMBS |  |
|  |  | HMCES |  |
|  |  | HMCN2 |  |
|  |  | CTLA4 |  |
|  |  | CTRC |  |
|  |  | CTSD |  |
|  |  | CTSL |  |
|  |  | CXXC1 |  |
|  |  | CYB5A |  |
|  |  | CYB5R1 |  |
|  |  | CYP2A13 |  |
|  |  | CYP4A11 |  |
|  |  | CYP7A1 |  |
|  |  | DAND5 |  |
|  |  | DAPL1 |  |
|  |  | DAPP1 |  |
|  |  | DARS |  |
|  |  | DARS2 |  |
|  |  | DAZ2 |  |
|  |  | DAZ3 |  |
|  |  | DBP |  |
|  |  | DCAF8 |  |
|  |  | DCLRE1A |  |
|  |  | IFITM10 |  |
|  |  | DDC |  |
|  |  | DDN |  |
|  |  | DDR1 |  |
|  |  | DDTL |  |
|  |  | DDX39A |  |
|  |  | DDX42 |  |
|  |  | DEAF1 |  |
|  |  | DEDD |  |
|  |  | DEF6 |  |
|  |  | DENND5A |  |
|  |  | DES |  |
|  |  | IL2RA |  |
|  |  | DGAT2 |  |
|  |  | DHH |  |
|  |  | INO80E |  |
|  |  | DLGAP4 |  |
|  |  | DLX4 |  |
|  |  | DMRTA2 |  |
|  |  | DNAAF2 |  |
|  |  | DNAAF3 |  |
|  |  | DNAH8 |  |
|  |  | DNAJC4 |  |
|  |  | DNAJC5G |  |
|  |  | DNASE2 |  |
|  |  | DND1 |  |
|  |  | DOHH |  |
|  |  | DOK1 |  |
|  |  | DOK3 |  |
|  |  | DOK5 |  |
|  |  | JMJD7-PLA2G4B |  |
|  |  | JMJD8 |  |
|  |  | DPH6 |  |
|  |  | DPT |  |
|  |  | DRD5 |  |
|  |  | DUSP14 |  |
|  |  | DUSP23 |  |
|  |  | DUSP26 |  |
|  |  | DYNLRB1 |  |
|  |  | EBP |  |
|  |  | EDC4 |  |
|  |  | KDM4D |  |
|  |  | SLC22A31 |  |
|  |  | SLC25A19 |  |
|  |  | SLC25A20 |  |
|  |  | SLC25A34 |  |
|  |  | SLC3A2 |  |
|  |  | SLC45A2 |  |
|  |  | SLC6A9 |  |
|  |  | SLC7A7 |  |
|  |  | SLC9A3R1 |  |
|  |  | SMARCD2 |  |
|  |  | SMG9 |  |
|  |  | SMIM4 |  |
|  |  | SMO |  |
|  |  | SNAI1 |  |
|  |  | SNCB |  |
|  |  | SNRNP70 |  |
|  |  | SNRPD2 |  |
|  |  | SNRPE |  |
|  |  | SNW1 |  |
|  |  | SNX19 |  |
|  |  | FAM118B |  |
|  |  | LGI4 |  |
|  |  | FAM13A |  |
|  |  | FAM154A |  |
|  |  | CACFD1 |  |
|  |  | CACNA1A |  |
|  |  | CACNA1H |  |
|  |  | CACNA1I |  |
|  |  | CACNA1S |  |
|  |  | CACNB1 |  |
|  |  | CACNG4 |  |
|  |  | CACNG7 |  |
|  |  | CACTIN |  |
|  |  | LMCD1 |  |
|  |  | CADM3 |  |
|  |  | CALB2 |  |
|  |  | CALHM1 |  |
|  |  | CALM2 |  |
|  |  | CALM3 |  |
|  |  | CALML3 |  |
|  |  | CAMK2A |  |
|  |  | CAMK2B |  |
|  |  | CAMKK2 |  |
|  |  | CAMKV |  |
|  |  | CAPN1 |  |
|  |  | CAPN11 |  |
|  |  | CAPN12 |  |
|  |  | CAPN5 |  |
|  |  | CAPN6 |  |
|  |  | CARD11 |  |
|  |  | CARD17 |  |
|  |  | CARHSP1 |  |
|  |  | CARKD |  |
|  |  | CARM1 |  |
|  |  | CARNS1 |  |
|  |  | CARS |  |
|  |  | CASKIN1 |  |
|  |  | CASKIN2 |  |
|  |  | CASP1 |  |
|  |  | CASP10 |  |
|  |  | CASP16 |  |
|  |  | CASR |  |
|  |  | CASS4 |  |
|  |  | CASZ1 |  |
|  |  | CATSPERG |  |
|  |  | CBLN1 |  |
|  |  | CBLN3 |  |
|  |  | CBR4 |  |
|  |  | CBS |  |
|  |  | CBX8 |  |
|  |  | CBY1 |  |
|  |  | CCAR2 |  |
|  |  | CCBL2 |  |
|  |  | CCDC103 |  |
|  |  | CCDC107 |  |
|  |  | CCDC124 |  |
|  |  | CCDC129 |  |
|  |  | CCDC130 |  |
|  |  | CCDC137 |  |
|  |  | CCDC140 |  |
|  |  | CCDC144NL |  |
|  |  | CCDC151 |  |
|  |  | CCDC160 |  |
|  |  | CCDC167 |  |
|  |  | CCDC42B |  |
|  |  | LYPD5 |  |
|  |  | CCDC57 |  |
|  |  | CCDC61 |  |
|  |  | CCDC67 |  |
|  |  | CCDC86 |  |
|  |  | CCDC88B |  |
|  |  | CCDC88C |  |
|  |  | CCDC92 |  |
|  |  | CCDC97 |  |
|  |  | MAF1 |  |
|  |  | MAFB |  |
|  |  | MAFF |  |
|  |  | MAG |  |
|  |  | MAGEA2 |  |
|  |  | CCNI2 |  |
|  |  | CCNL2 |  |
|  |  | CCR1 |  |
|  |  | CCR5 |  |
|  |  | CCR7 |  |
|  |  | CCZ1 |  |
|  |  | CCZ1B |  |
|  |  | CD151 |  |
|  |  | CD164L2 |  |
|  |  | CD177 |  |
|  |  | CD180 |  |
|  |  | CD27 |  |
|  |  | CD276 |  |
|  |  | CD300LD |  |
|  |  | CD300LF |  |
|  |  | CD320 |  |
|  |  | CD37 |  |
|  |  | CD3D |  |
|  |  | CD40 |  |
|  |  | CD5L |  |
|  |  | CD6 |  |
|  |  | CD7 |  |
|  |  | CD72 |  |
|  |  | CD74 |  |
|  |  | CD79A |  |
|  |  | CD93 |  |
|  |  | CD96 |  |
|  |  | CD97 |  |
|  |  | CD99 |  |
|  |  | CDAN1 |  |
|  |  | CDC14B |  |
|  |  | CDC23 |  |
|  |  | CDC25B |  |
|  |  | CDC37L1 |  |
|  |  | CDC40 |  |
|  |  | CDC42BPB |  |
|  |  | CDC42BPG |  |
|  |  | CDC42EP1 |  |
|  |  | CDC42EP2 |  |
|  |  | MAX |  |
|  |  | MAZ |  |
|  |  | MB |  |
|  |  | MB21D2 |  |
|  |  | MBD3 |  |
|  |  | MBD6 |  |
|  |  | MCAM |  |
|  |  | MCM2 |  |
|  |  | MCM7 |  |
|  |  | MCM8 |  |
|  |  | MED11 |  |
|  |  | MED13L |  |
|  |  | MED15 |  |
|  |  | MED29 |  |
|  |  | MEFV |  |
|  |  | MEGF6 |  |
|  |  | MEOX1 |  |
|  |  | METAP1D |  |
|  |  | METRN |  |
|  |  | METTL10 |  |
|  |  | METTL12 |  |
|  |  | METTL13 |  |
|  |  | METTL21B |  |
|  |  | METTL22 |  |
|  |  | METTL6 |  |
|  |  | METTL7B |  |
|  |  | MFAP1 |  |
|  |  | MFAP4 |  |
|  |  | MFGE8 |  |
|  |  | MFN2 |  |
|  |  | MFNG |  |
|  |  | MFRP |  |
|  |  | MFSD6L |  |
|  |  | MFSD7 |  |
|  |  | MGAT3 |  |
|  |  | MGAT5B |  |
|  |  | MGMT |  |
|  |  | MGST1 |  |
|  |  | MIA |  |
|  |  | MICAL1 |  |
|  |  | MICU1 |  |
|  |  | MIEN1 |  |
|  |  | MIER2 |  |
|  |  | MIF4GD |  |
|  |  | MIIP |  |
|  |  | MINK1 |  |
|  |  | MINOS1-NBL1 |  |
|  |  | MIOX |  |
|  |  | MIP |  |
|  |  | MIS18A |  |
|  |  | MISP |  |
|  |  | MKI67IP |  |
|  |  | MKRN3 |  |
|  |  | MKS1 |  |
|  |  | MLC1 |  |
|  |  | MLIP |  |
|  |  | MLLT11 |  |
|  |  | MLPH |  |
|  |  | MLST8 |  |
|  |  | MLX |  |
|  |  | MLXIPL |  |
|  |  | MMAB |  |
|  |  | GINS3 |  |
|  |  | MMEL1 |  |
|  |  | MMP11 |  |
|  |  | MMP17 |  |
|  |  | MMP2 |  |
|  |  | MMP23B |  |
|  |  | MMP24 |  |
|  |  | MMP3 |  |
|  |  | MMP8 |  |
|  |  | MMS19 |  |
|  |  | MOB2 |  |
|  |  | MOB3C |  |
|  |  | MOCOS |  |
|  |  | MOCS1 |  |
|  |  | MOGS |  |
|  |  | MON1A |  |
|  |  | MORC2 |  |
|  |  | MORN3 |  |
|  |  | MOV10 |  |
|  |  | MOV10L1 |  |
|  |  | MPC1 |  |
|  |  | MPDU1 |  |
|  |  | MPEG1 |  |
|  |  | MPHOSPH9 |  |
|  |  | MPI |  |
|  |  | MPL |  |
|  |  | MPST |  |
|  |  | MPZ |  |
|  |  | MRAS |  |
|  |  | MRC2 |  |
|  |  | MRGPRF |  |
|  |  | MRO |  |
|  |  | MROH1 |  |
|  |  | MROH6 |  |
|  |  | MRPL10 |  |
|  |  | MRPL12 |  |
|  |  | MRPL14 |  |
|  |  | MRPL23 |  |
|  |  | MRPL24 |  |
|  |  | MRPL37 |  |
|  |  | MRPL40 |  |
|  |  | MRPL48 |  |
|  |  | MRPL49 |  |
|  |  | MRPL53 |  |
|  |  | MRPS12 |  |
|  |  | MRPS14 |  |
|  |  | MRPS2 |  |
|  |  | MRPS34 |  |
|  |  | MRPS35 |  |
|  |  | MRPS7 |  |
|  |  | MRTO4 |  |
|  |  | MS4A3 |  |
|  |  | MSANTD3 |  |
|  |  | MSC |  |
|  |  | MSH5 |  |
|  |  | MSLN |  |
|  |  | MSMP |  |
|  |  | MSS51 |  |
|  |  | MST1R |  |
|  |  | MT1H |  |
|  |  | MT2A |  |
|  |  | MT3 |  |
|  |  | MT-ATP6 |  |
|  |  | MTCP1 |  |
|  |  | MTERF |  |
|  |  | MTF1 |  |
|  |  | GPR133 |  |
|  |  | MTG2 |  |
|  |  | MTHFD1L |  |
|  |  | MTHFSD |  |
|  |  | MTMR11 |  |
|  |  | MTNR1B |  |
|  |  | MUC1 |  |
|  |  | MUC12 |  |
|  |  | MUC13 |  |
|  |  | MUC20 |  |
|  |  | MUC21 |  |
|  |  | MUC5B |  |
|  |  | MUC6 |  |
|  |  | MURC |  |
|  |  | MUSTN1 |  |
|  |  | MVB12B |  |
|  |  | MVK |  |
|  |  | MXRA8 |  |
|  |  | MYADML2 |  |
|  |  | MYBBP1A |  |
|  |  | MYBL2 |  |
|  |  | GRB2 |  |
|  |  | MYH14 |  |
|  |  | MYL1 |  |
|  |  | MYL3 |  |
|  |  | MYLIP |  |
|  |  | MYO1A |  |
|  |  | MYO1C |  |
|  |  | MYO1D |  |
|  |  | MYO1F |  |
|  |  | MYO1G |  |
|  |  | MYO3A |  |
|  |  | MYO5C |  |
|  |  | MYO9B |  |
|  |  | MYOF |  |
|  |  | GSG1 |  |
|  |  | MYRF |  |
|  |  | MYZAP |  |
|  |  | GTF2F1 |  |
|  |  | GTF2I |  |
|  |  | GTF3C2 |  |
|  |  | GTPBP6 |  |
|  |  | GUCA1B |  |
|  |  | GUCD1 |  |
|  |  | NANOS2 |  |
|  |  | NAPA |  |
|  |  | NAPRT1 |  |
|  |  | GYPC |  |
|  |  | NASP |  |
|  |  | NAT10 |  |
|  |  | NBL1 |  |
|  |  | NBPF11 |  |
|  |  | HADHA |  |
|  |  | HAGH |  |
|  |  | HAGHL |  |
|  |  | HAMP |  |
|  |  | HAND1 |  |
|  |  | NCAPD2 |  |
|  |  | NCAPH2 |  |
|  |  | NCF1 |  |
|  |  | NCF4 |  |
|  |  | NCK1 |  |
|  |  | NCKAP5L |  |
|  |  | NCMAP |  |
|  |  | NCOA1 |  |
|  |  | NCSTN |  |
|  |  | HCN2 |  |
|  |  | NDOR1 |  |
|  |  | NDST1 |  |
|  |  | NDUFA11 |  |
|  |  | NDUFA4 |  |
|  |  | NDUFAF3 |  |
|  |  | NDUFB10 |  |
|  |  | NDUFS7 |  |
|  |  | NDUFS8 |  |
|  |  | NEB |  |
|  |  | NECAB2 |  |
|  |  | NECAB3 |  |
|  |  | NEDD4 |  |
|  |  | NEIL2 |  |
|  |  | NELFA |  |
|  |  | NELFCD |  |
|  |  | NEO1 |  |
|  |  | NES |  |
|  |  | NEU3 |  |
|  |  | NEURL1B |  |
|  |  | NEUROD2 |  |
|  |  | NFE2 |  |
|  |  | NFIC |  |
|  |  | NFKB1 |  |
|  |  | NFRKB |  |
|  |  | NFS1 |  |
|  |  | NGFR |  |
|  |  | NGRN |  |
|  |  | NHLRC4 |  |
|  |  | NHP2 |  |
|  |  | NHSL2 |  |
|  |  | NID1 |  |
|  |  | NIPAL4 |  |
|  |  | NISCH |  |
|  |  | NIT1 |  |
|  |  | NKAIN4 |  |
|  |  | NKD2 |  |
|  |  | NKPD1 |  |
|  |  | NKX2-1 |  |
|  |  | NKX3-2 |  |
|  |  | NKX6-3 |  |
|  |  | NLGN2 |  |
|  |  | NLRP1 |  |
|  |  | NLRP3 |  |
|  |  | NLRP6 |  |
|  |  | NMB |  |
|  |  | NME1-NME2 |  |
|  |  | NME2 |  |
|  |  | NME6 |  |
|  |  | NME9 |  |
|  |  | NMNAT3 |  |
|  |  | NMT1 |  |
|  |  | NNMT |  |
|  |  | NOC2L |  |
|  |  | NODAL |  |
|  |  | HOMEZ |  |
|  |  | HOOK1 |  |
|  |  | NOL9 |  |
|  |  | NOMO1 |  |
|  |  | NOMO2 |  |
|  |  | NOMO3 |  |
|  |  | NOP16 |  |
|  |  | NOS1AP |  |
|  |  | NOS2 |  |
|  |  | NOSIP |  |
|  |  | NOTO |  |
|  |  | HOXB9 |  |
|  |  | NOX1 |  |
|  |  | NPAP1 |  |
|  |  | NPAS1 |  |
|  |  | NPEPPS |  |
|  |  | NPHP4 |  |
|  |  | NPM2 |  |
|  |  | NPR2 |  |
|  |  | NPRL3 |  |
|  |  | NPTXR |  |
|  |  | NPY4R |  |
|  |  | NR1H2 |  |
|  |  | NR1H3 |  |
|  |  | NR1I2 |  |
|  |  | NR1I3 |  |
|  |  | NR2C2AP |  |
|  |  | NR2F6 |  |
|  |  | NR5A1 |  |
|  |  | HSD17B8 |  |
|  |  | HSD3B1 |  |
|  |  | HSD3B2 |  |
|  |  | HSD3B7 |  |
|  |  | HSF1 |  |
|  |  | HSP90AB1 |  |
|  |  | HSPA12B |  |
|  |  | NRL |  |
|  |  | NRM |  |
|  |  | NRN1 |  |
|  |  | NRROS |  |
|  |  | HTR1D |  |
|  |  | NSFL1C |  |
|  |  | NSMAF |  |
|  |  | NSMCE1 |  |
|  |  | NSMF |  |
|  |  | HYAL3 |  |
|  |  | HYDIN |  |
|  |  | NTAN1 |  |
|  |  | NTM |  |
|  |  | NTN4 |  |
|  |  | NTNG2 |  |
|  |  | NTRK3 |  |
|  |  | NTSR2 |  |
|  |  | NUAK2 |  |
|  |  | NUBP2 |  |
|  |  | NUCB1 |  |
|  |  | NUCB2 |  |
|  |  | NUDC |  |
|  |  | NUDT1 |  |
|  |  | NUDT15 |  |
|  |  | NUDT16L1 |  |
|  |  | NUDT8 |  |
|  |  | NUMA1 |  |
|  |  | NUMBL |  |
|  |  | NUP205 |  |
|  |  | NUP98 |  |
|  |  | NUSAP1 |  |
|  |  | NUTF2 |  |
|  |  | NUTM1 |  |
|  |  | NUTM2B |  |
|  |  | NUTM2D |  |
|  |  | NUTM2G |  |
|  |  | NVL |  |
|  |  | NXPH4 |  |
|  |  | NYAP1 |  |
|  |  | OAF |  |
|  |  | OAS2 |  |
|  |  | OAZ1 |  |
|  |  | OAZ2 |  |
|  |  | ODC1 |  |
|  |  | ODF3 |  |
|  |  | ODF3L1 |  |
|  |  | ODF3L2 |  |
|  |  | OGDH |  |
|  |  | OGFOD3 |  |
|  |  | OGFR |  |
|  |  | OLFM1 |  |
|  |  | IL2RB |  |
|  |  | IL3 |  |
|  |  | BARHL1 |  |
|  |  | BAX |  |
|  |  | BCAP31 |  |
|  |  | BCKDHA |  |
|  |  | BDKRB1 |  |
|  |  | BLK |  |
|  |  | BMPER |  |
|  |  | BOC |  |
|  |  | BOK |  |
|  |  | BPNT1 |  |
|  |  | BRK1 |  |
|  |  | ITPR2 |  |
|  |  | BTBD10 |  |
|  |  | C10orf55 |  |
|  |  | C10orf62 |  |
|  |  | C11orf34 |  |
|  |  | C11orf63 |  |
|  |  | C11orf83 |  |
|  |  | C12orf60 |  |
|  |  | KCNE1L |  |
|  |  | C12orf77 |  |
|  |  | KCNH3 |  |
|  |  | KCNH6 |  |
|  |  | KCNIP1 |  |
|  |  | C14orf79 |  |
|  |  | C16orf89 |  |
|  |  | C17orf67 |  |
|  |  | C19orf70 |  |
|  |  | C1orf127 |  |
|  |  | C1orf159 |  |
|  |  | C1orf216 |  |
|  |  | C1orf234 |  |
|  |  | C1orf68 |  |
|  |  | C20orf24 |  |
|  |  | C2orf42 |  |
|  |  | C2orf66 |  |
|  |  | C3orf35 |  |
|  |  | C4orf26 |  |
|  |  | C5orf28 |  |
|  |  | C5orf38 |  |
|  |  | KLF16 |  |
|  |  | C6orf141 |  |
|  |  | KLHL25 |  |
|  |  | C7orf55 |  |
|  |  | C8orf31 |  |
|  |  | KLK10 |  |
|  |  | C8orf47 |  |
|  |  | C8orf59 |  |
|  |  | C9orf37 |  |
|  |  | C9orf89 |  |
|  |  | CA4 |  |
|  |  | CABP2 |  |
|  |  | CACNB3 |  |
|  |  | CACNG1 |  |
|  |  | KRTAP24-1 |  |
|  |  | CALML5 |  |
|  |  | CALY |  |
|  |  | KXD1 |  |
|  |  | LAD1 |  |
|  |  | LAIR1 |  |
|  |  | CAPG |  |
|  |  | CAPN13 |  |
|  |  | CAPN3 |  |
|  |  | LARGE |  |
|  |  | CARD10 |  |
|  |  | CARD14 |  |
|  |  | CASP7 |  |
|  |  | CAT |  |
|  |  | CBFA2T3 |  |
|  |  | LEPREL2 |  |
|  |  | CBWD3 |  |
|  |  | CBWD6 |  |
|  |  | CBWD7 |  |
|  |  | CBY3 |  |
|  |  | CC2D2A |  |
|  |  | CCDC116 |  |
|  |  | CCDC120 |  |
|  |  | CCDC13 |  |
|  |  | CCDC135 |  |
|  |  | CCDC142 |  |
|  |  | CCDC146 |  |
|  |  | CCDC15 |  |
|  |  | CCDC155 |  |
|  |  | CCDC19 |  |
|  |  | CCDC24 |  |
|  |  | LMAN2 |  |
|  |  | LMAN2L |  |
|  |  | CCDC78 |  |
|  |  | LOH12CR2 |  |
|  |  | CCHCR1 |  |
|  |  | FLRT1 |  |
|  |  | CCL16 |  |
|  |  | CCL18 |  |
|  |  | CCL3 |  |
|  |  | CCNI |  |
|  |  | SLC17A7 |  |
|  |  | CCT7 |  |
|  |  | LRRC41 |  |
|  |  | CD300A |  |
|  |  | CD300C |  |
|  |  | CD300LG |  |
|  |  | CD3E |  |
|  |  | CD53 |  |
|  |  | CD63 |  |
|  |  | CD81 |  |
|  |  | CD83 |  |
|  |  | FUT6 |  |
|  |  | CD8B |  |
|  |  | CDA |  |
|  |  | CDC16 |  |
|  |  | CDC42SE2 |  |
|  |  | GABARAPL1 |  |
|  |  | CDH17 |  |
|  |  | CDHR5 |  |
|  |  | CDK1 |  |
|  |  | CDK11A |  |
|  |  | CDK11B |  |
|  |  | CDK5R2 |  |
|  |  | CDK7 |  |
|  |  | CEACAM19 |  |
|  |  | CEACAM21 |  |
|  |  | CECR1 |  |
|  |  | CELSR3 |  |
|  |  | CERS4 |  |
|  |  | CETP |  |
|  |  | CFL1 |  |
|  |  | CHD1L |  |
|  |  | CHD8 |  |
|  |  | CHID1 |  |
|  |  | CHMP2A |  |
|  |  | RAB4B |  |
|  |  | RAB5C |  |
|  |  | RABL2A |  |
|  |  | RABL2B |  |
|  |  | RAC2 |  |
|  |  | CHST15 |  |
|  |  | RALGAPA1 |  |
|  |  | GPR62 |  |
|  |  | RBP4 |  |
|  |  | RFX2 |  |
|  |  | RHBDF1 |  |
|  |  | RHBG |  |
|  |  | RHO |  |
|  |  | RHOA |  |
|  |  | RIC8A |  |
|  |  | RIIAD1 |  |
|  |  | RILPL2 |  |
|  |  | RIPK3 |  |
|  |  | RNASE8 |  |
|  |  | RNASET2 |  |
|  |  | HES2 |  |
|  |  | RNF186 |  |
|  |  | RNF207 |  |
|  |  | RNF208 |  |
|  |  | RNF26 |  |
|  |  | RNF40 |  |
|  |  | RP1 |  |
|  |  | RP11-178L8.4 |  |
|  |  | RP11-20I23.1 |  |
|  |  | RP11-298I3.5 |  |
|  |  | RP11-318A15.7 |  |
|  |  | RP11-363G10.2 |  |
|  |  | RP11-794P6.2 |  |
|  |  | RP11-863K10.7 |  |
|  |  | RP11-89N17.1 |  |
|  |  | RP11-94B19.4 |  |
|  |  | RP1L1 |  |
|  |  | RP5-1052I5.2 |  |
|  |  | RPL11 |  |
|  |  | RPL4 |  |
|  |  | RPL41 |  |
|  |  | RPN2 |  |
|  |  | RPS11 |  |
|  |  | RPS19 |  |
|  |  | RSPH4A |  |
|  |  | RTEL1 |  |
|  |  | RTN1 |  |
|  |  | ICA1 |  |
|  |  | S100A16 |  |
|  |  | S1PR5 |  |
|  |  | SAGE1 |  |
|  |  | NEIL1 |  |
|  |  | SAP130 |  |
|  |  | SCD5 |  |
|  |  | SCEL |  |
|  |  | SCG3 |  |
|  |  | SCGB1A1 |  |
|  |  | SCGB2A1 |  |
|  |  | SCLY |  |
|  |  | SCN7A |  |
|  |  | SCNN1B |  |
|  |  | SCRN2 |  |
|  |  | SDIM1 |  |
|  |  | SDR16C5 |  |
|  |  | SEC11C |  |
|  |  | SEC13 |  |
|  |  | SEC61A1 |  |
|  |  | SERPINB13 |  |
|  |  | SERPINE3 |  |
|  |  | SET |  |
|  |  | SEZ6L |  |
|  |  | SF3A2 |  |
|  |  | SH2B1 |  |
|  |  | SHARPIN |  |
|  |  | SHC2 |  |
|  |  | SHF |  |
|  |  | NXF2 |  |
|  |  | NXF2B |  |
|  |  | OASL |  |
|  |  | KHSRP |  |
|  |  | KIAA0020 |  |
|  |  | KIAA0195 |  |
|  |  | KIAA0226 |  |
|  |  | KIAA0247 |  |
|  |  | KIAA0319L |  |
|  |  | KIAA0368 |  |
|  |  | KIAA0895L |  |
|  |  | KIAA0907 |  |
|  |  | KIAA0922 |  |
|  |  | KIAA1045 |  |
|  |  | KIAA1324 |  |
|  |  | KIAA1324L |  |
|  |  | KIAA1755 |  |
|  |  | KIAA1875 |  |
|  |  | KIAA1919 |  |
|  |  | KIAA2013 |  |
|  |  | KIF12 |  |
|  |  | PAH |  |
|  |  | HEBP1 |  |
|  |  | KIF26A |  |
|  |  | KIF2C |  |
|  |  | KIF4B |  |
|  |  | KIF7 |  |
|  |  | KIFC2 |  |
|  |  | KIFC3 |  |
|  |  | KIR3DL1 |  |
|  |  | KIR3DL2 |  |
|  |  | KIR3DL3 |  |
|  |  | KIR3DX1 |  |
|  |  | KIRREL3 |  |
|  |  | KLF17 |  |
|  |  | KLHDC3 |  |
|  |  | KLHDC7A |  |
|  |  | KLHDC7B |  |
|  |  | KLHL30 |  |
|  |  | KLHL36 |  |
|  |  | KLHL38 |  |
|  |  | PCBP3 |  |
|  |  | KLK12 |  |
|  |  | KLK2 |  |
|  |  | KLK4 |  |
|  |  | KLK5 |  |
|  |  | KLK9 |  |
|  |  | HMG20B |  |
|  |  | KLRG2 |  |
|  |  | KMT2D |  |
|  |  | KNCN |  |
|  |  | KNDC1 |  |
|  |  | KNOP1 |  |
|  |  | KPRP |  |
|  |  | KREMEN1 |  |
|  |  | KRIT1 |  |
|  |  | KRT1 |  |
|  |  | KRT20 |  |
|  |  | KRT34 |  |
|  |  | KRT5 |  |
|  |  | KRT71 |  |
|  |  | KRT73 |  |
|  |  | KRT74 |  |
|  |  | KRT75 |  |
|  |  | KRT76 |  |
|  |  | KRT78 |  |
|  |  | KRT79 |  |
|  |  | KRT82 |  |
|  |  | KRTAP10-1 |  |
|  |  | KRTAP10-11 |  |
|  |  | KRTAP10-4 |  |
|  |  | KRTAP10-5 |  |
|  |  | KRTAP10-6 |  |
|  |  | KRTAP10-8 |  |
|  |  | KRTAP10-9 |  |
|  |  | KRTAP12-1 |  |
|  |  | KRTAP1-5 |  |
|  |  | KRTAP2-1 |  |
|  |  | KRTAP2-2 |  |
|  |  | KRTAP2-3 |  |
|  |  | KRTAP4-6 |  |
|  |  | KRTAP5-2 |  |
|  |  | KRTAP5-4 |  |
|  |  | KRTAP5-7 |  |
|  |  | KRTAP5-8 |  |
|  |  | KRTAP6-3 |  |
|  |  | KRTAP9-2 |  |
|  |  | KRTAP9-8 |  |
|  |  | KRTDAP |  |
|  |  | KY |  |
|  |  | L1CAM |  |
|  |  | LAMC3 |  |
|  |  | LAMP3 |  |
|  |  | LAMTOR1 |  |
|  |  | LAMTOR3 |  |
|  |  | LAPTM4B |  |
|  |  | LAPTM5 |  |
|  |  | LASP1 |  |
|  |  | LBX2 |  |
|  |  | LCE1A |  |
|  |  | LCE1B |  |
|  |  | LCE1D |  |
|  |  | LCE1F |  |
|  |  | LCE3D |  |
|  |  | LCN2 |  |
|  |  | LCNL1 |  |
|  |  | LCORL |  |
|  |  | LDHD |  |
|  |  | PFDN1 |  |
|  |  | SOCS1 |  |
|  |  | SOCS3 |  |
|  |  | SPAG11A |  |
|  |  | SPAG8 |  |
|  |  | SPATA20 |  |
|  |  | SPATA8 |  |
|  |  | SPERT |  |
|  |  | PIGU |  |
|  |  | SSBP1 |  |
|  |  | SSH3 |  |
|  |  | SSR2 |  |
|  |  | STAB1 |  |
|  |  | STAG3 |  |
|  |  | STK11 |  |
|  |  | STX5 |  |
|  |  | STX8 |  |
|  |  | SUMF1 |  |
|  |  | SUSD2 |  |
|  |  | SYK |  |
|  |  | SYNE2 |  |
|  |  | SYNE3 |  |
|  |  | SYNGR3 |  |
|  |  | SYT3 |  |
|  |  | TAC1 |  |
|  |  | TAC3 |  |
|  |  | TAS2R20 |  |
|  |  | TBC1D10C |  |
|  |  | TBC1D31 |  |
|  |  | TBCB |  |
|  |  | TBKBP1 |  |
|  |  | TBX21 |  |
|  |  | TCEA3 |  |
|  |  | TDP2 |  |
|  |  | TESC |  |
|  |  | TFAP2E |  |
|  |  | TGFBI |  |
|  |  | PRPF8 |  |
|  |  | THEM5 |  |
|  |  | THYN1 |  |
|  |  | TICAM1 |  |
|  |  | TIMM8B |  |
|  |  | TIPIN |  |
|  |  | TLCD1 |  |
|  |  | TLR9 |  |
|  |  | TLX1 |  |
|  |  | TM6SF2 |  |
|  |  | PTPRA |  |
|  |  | ISYNA1 |  |
|  |  | TMEM174 |  |
|  |  | RAB20 |  |
|  |  | JPH2 |  |
|  |  | JPH4 |  |
|  |  | GPR171 |  |
|  |  | RAB6C |  |
|  |  | GPR35 |  |
|  |  | GPR42 |  |
|  |  | GPR55 |  |
|  |  | GPR61 |  |
|  |  | GPR65 |  |
|  |  | GPR68 |  |
|  |  | GPR78 |  |
|  |  | GPR88 |  |
|  |  | GPR97 |  |
|  |  | GPRC5C |  |
|  |  | GPS1 |  |
|  |  | GPSM1 |  |
|  |  | GPSM3 |  |
|  |  | GPT2 |  |
|  |  | GPX1 |  |
|  |  | GPX3 |  |
|  |  | GPX4 |  |
|  |  | GRAMD3 |  |
|  |  | LMOD3 |  |
|  |  | LMX1B |  |
|  |  | FKBP5 |  |
|  |  | GRID1 |  |
|  |  | GRID2IP |  |
|  |  | GRIN1 |  |
|  |  | GRIN2C |  |
|  |  | GRINA |  |
|  |  | GRK6 |  |
|  |  | GRK7 |  |
|  |  | GRM4 |  |
|  |  | GRTP1 |  |
|  |  | GSDMA |  |
|  |  | LRFN1 |  |
|  |  | GSG2 |  |
|  |  | GSTM1 |  |
|  |  | GSTM2 |  |
|  |  | GSTZ1 |  |
|  |  | LRRC14 |  |
|  |  | LRRC14B |  |
|  |  | GTSF1 |  |
|  |  | LRRC29 |  |
|  |  | LRRC38 |  |
|  |  | GUK1 |  |
|  |  | GUSB |  |
|  |  | GYG1 |  |
|  |  | GYLTL1B |  |
|  |  | HAAO |  |
|  |  | HABP2 |  |
|  |  | HADH |  |
|  |  | LRRN2 |  |
|  |  | HAO2 |  |
|  |  | HAPLN3 |  |
|  |  | HAPLN4 |  |
|  |  | HAUS5 |  |
|  |  | HAUS7 |  |
|  |  | HAVCR2 |  |
|  |  | HBEGF |  |
|  |  | HBG1 |  |
|  |  | HBM |  |
|  |  | HCAR2 |  |
|  |  | HCAR3 |  |
|  |  | LTBP3 |  |
|  |  | HCFC1 |  |
|  |  | HCG27 |  |
|  |  | HCK |  |
|  |  | HCLS1 |  |
|  |  | HDAC1 |  |
|  |  | HDAC11 |  |
|  |  | HDAC3 |  |
|  |  | HDAC7 |  |
|  |  | HDGF |  |
|  |  | HDGFL1 |  |
|  |  | HDHD3 |  |
|  |  | HELZ2 |  |
|  |  | HERPUD2 |  |
|  |  | HEXDC |  |
|  |  | HEXIM1 |  |
|  |  | HEXIM2 |  |
|  |  | HFE |  |
|  |  | HHIPL2 |  |
|  |  | HHLA3 |  |
|  |  | HIC1 |  |
|  |  | HID1 |  |
|  |  | HIF3A |  |
|  |  | HINFP |  |
|  |  | HIP1R |  |
|  |  | HIPK2 |  |
|  |  | MAP1LC3C |  |
|  |  | MAP2K2 |  |
|  |  | HLA-DQB1 |  |
|  |  | MAPK3 |  |
|  |  | HMGA1 |  |
|  |  | HMHA1 |  |
|  |  | HMOX2 |  |
|  |  | HMSD |  |
|  |  | HN1L |  |
|  |  | HNF1A |  |
|  |  | HNMT |  |
|  |  | HNRNPUL1 |  |
|  |  | HOGA1 |  |
|  |  | HOOK2 |  |
|  |  | HORMAD1 |  |
|  |  | HOXA1 |  |
|  |  | HOXA3 |  |
|  |  | HOXB13 |  |
|  |  | HOXB5 |  |
|  |  | HOXC11 |  |
|  |  | HOXC4 |  |
|  |  | HOXC5 |  |
|  |  | HOXC6 |  |
|  |  | HOXC8 |  |
|  |  | HOXD1 |  |
|  |  | HPCAL1 |  |
|  |  | HPCAL4 |  |
|  |  | HPD |  |
|  |  | HPS3 |  |
|  |  | HPS5 |  |
|  |  | HPSE2 |  |
|  |  | HRH3 |  |
|  |  | HRH4 |  |
|  |  | HS1BP3 |  |
|  |  | HS3ST4 |  |
|  |  | HSCB |  |
|  |  | MED8 |  |
|  |  | HSPB1 |  |
|  |  | HSPB7 |  |
|  |  | HTR3A |  |
|  |  | HTR3E |  |
|  |  | HTRA1 |  |
|  |  | HTRA2 |  |
|  |  | CIZ1 |  |
|  |  | HYOU1 |  |
|  |  | IAPP |  |
|  |  | ICAM1 |  |
|  |  | ID1 |  |
|  |  | IDH3A |  |
|  |  | IDO2 |  |
|  |  | IDS |  |
|  |  | IER3 |  |
|  |  | IER5 |  |
|  |  | IFFO1 |  |
|  |  | IFI30 |  |
|  |  | IFNAR2 |  |
|  |  | IFNG |  |
|  |  | IFNGR2 |  |
|  |  | IFNK |  |
|  |  | IFNLR1 |  |
|  |  | IFRD2 |  |
|  |  | IFT46 |  |
|  |  | IFT81 |  |
|  |  | ATP8B3 |  |
|  |  | ATRIP |  |
|  |  | ATXN2L |  |
|  |  | IL27 |  |
|  |  | GRAP |  |
|  |  | IL36RN |  |
|  |  | IL4R |  |
|  |  | IL9R |  |
|  |  | ILDR1 |  |
|  |  | ILVBL |  |
|  |  | IMPA2 |  |
|  |  | IMPDH1 |  |
|  |  | INF2 |  |
|  |  | GRM8 |  |
|  |  | INO80B |  |
|  |  | INO80C |  |
|  |  | INPP5J |  |
|  |  | INPP5K |  |
|  |  | INSM1 |  |
|  |  | INTS3 |  |
|  |  | INTS9 |  |
|  |  | IP6K1 |  |
|  |  | IP6K3 |  |
|  |  | IQCE |  |
|  |  | IQCG |  |
|  |  | IQCK |  |
|  |  | IQSEC2 |  |
|  |  | GUCA2B |  |
|  |  | IRF2 |  |
|  |  | IRF2BP1 |  |
|  |  | GYS1 |  |
|  |  | GZMK |  |
|  |  | H1FNT |  |
|  |  | H2AFJ |  |
|  |  | H2BFM |  |
|  |  | ISG20L2 |  |
|  |  | ISL2 |  |
|  |  | ISM2 |  |
|  |  | ISY1 |  |
|  |  | ISY1-RAB43 |  |
|  |  | ITFG2 |  |
|  |  | ITFG3 |  |
|  |  | ITGA10 |  |
|  |  | ITGA11 |  |
|  |  | ITGA2B |  |
|  |  | ITGAL |  |
|  |  | ITGAM |  |
|  |  | ITGAX |  |
|  |  | ITGB2 |  |
|  |  | ITGB5 |  |
|  |  | ITIH1 |  |
|  |  | ITM2C |  |
|  |  | ITPA |  |
|  |  | ITPKB |  |
|  |  | HDDC2 |  |
|  |  | ITPRIPL1 |  |
|  |  | JAG2 |  |
|  |  | JMJD4 |  |
|  |  | JOSD2 |  |
|  |  | KANSL1 |  |
|  |  | KAT8 |  |
|  |  | KATNB1 |  |
|  |  | KAZN |  |
|  |  | KBTBD12 |  |
|  |  | KCNA2 |  |
|  |  | KCNA3 |  |
|  |  | KCNA7 |  |
|  |  | KCNC3 |  |
|  |  | KCND1 |  |
|  |  | KCNF1 |  |
|  |  | MYH8 |  |
|  |  | KCNIP2 |  |
|  |  | KCNIP3 |  |
|  |  | KCNJ12 |  |
|  |  | KCNJ5 |  |
|  |  | KCNK3 |  |
|  |  | KCNK7 |  |
|  |  | KCNMB4 |  |
|  |  | KCNN1 |  |
|  |  | KCNQ1 |  |
|  |  | KCNQ4 |  |
|  |  | KCNRG |  |
|  |  | KCTD10 |  |
|  |  | KCTD11 |  |
|  |  | KCTD17 |  |
|  |  | HOXA11 |  |
|  |  | KDM4B |  |
|  |  | KDM8 |  |
|  |  | KIAA1257 |  |
|  |  | IGF2 |  |
|  |  | KLHL35 |  |
|  |  | IL13RA1 |  |
|  |  | IL17REL |  |
|  |  | IL19 |  |
|  |  | IL25 |  |
|  |  | KRI1 |  |
|  |  | KRT83 |  |
|  |  | KRT85 |  |
|  |  | KRTAP10-2 |  |
|  |  | KRTAP10-3 |  |
|  |  | KRTAP11-1 |  |
|  |  | KRTAP12-2 |  |
|  |  | KRTAP13-2 |  |
|  |  | KRTAP4-5 |  |
|  |  | LALBA |  |
|  |  | LAMA1 |  |
|  |  | LAMP1 |  |
|  |  | IRX4 |  |
|  |  | LAT |  |
|  |  | LAYN |  |
|  |  | LCN8 |  |
|  |  | LDLRAD1 |  |
|  |  | LDLRAD2 |  |
|  |  | LDLRAP1 |  |
|  |  | LDOC1 |  |
|  |  | LEPREL4 |  |
|  |  | LGALS3BP |  |
|  |  | LHFPL4 |  |
|  |  | LHPP |  |
|  |  | GPR157 |  |
|  |  | JUN |  |
|  |  | KAT5 |  |
|  |  | LIME1 |  |
|  |  | LIMS3 |  |
|  |  | LIMS3L |  |
|  |  | LINC00923 |  |
|  |  | LINGO1 |  |
|  |  | LINGO4 |  |
|  |  | LINS |  |
|  |  | LIPC |  |
|  |  | LITAF |  |
|  |  | LMF2 |  |
|  |  | LMLN |  |
|  |  | FKBP4 |  |
|  |  | FKBP6 |  |
|  |  | FKBP8 |  |
|  |  | LOR |  |
|  |  | LOXL3 |  |
|  |  | FLJ20373 |  |
|  |  | LPAR5 |  |
|  |  | LPHN1 |  |
|  |  | FLYWCH1 |  |
|  |  | LPIN3 |  |
|  |  | LPXN |  |
|  |  | LRG1 |  |
|  |  | LRGUK |  |
|  |  | LRP1 |  |
|  |  | FOSB |  |
|  |  | LRP5 |  |
|  |  | FOXH1 |  |
|  |  | KCTD8 |  |
|  |  | LRRC25 |  |
|  |  | LRRC3B |  |
|  |  | LRRC3DN |  |
|  |  | FRAT2 |  |
|  |  | LRRC46 |  |
|  |  | LRRC47 |  |
|  |  | FRMD1 |  |
|  |  | LRRC56 |  |
|  |  | LRRC59 |  |
|  |  | LRRC63 |  |
|  |  | FSCN1 |  |
|  |  | LRTOMT |  |
|  |  | LSM2 |  |
|  |  | FUOM |  |
|  |  | LSM7 |  |
|  |  | FUS |  |
|  |  | LSMEM2 |  |
|  |  | LSP1 |  |
|  |  | LURAP1 |  |
|  |  | LY6H |  |
|  |  | LY6K |  |
|  |  | LY86 |  |
|  |  | LYAR |  |
|  |  | LYL1 |  |
|  |  | FZR1 |  |
|  |  | LYZ |  |
|  |  | LZTS1 |  |
|  |  | LZTS3 |  |
|  |  | M6PR |  |
|  |  | MAD1L1 |  |
|  |  | MAD2L1BP |  |
|  |  | MADD |  |
|  |  | MAGEB2 |  |
|  |  | MAL |  |
|  |  | MALL |  |
|  |  | MAML1 |  |
|  |  | MAMSTR |  |
|  |  | MAN2B2 |  |
|  |  | MANEAL |  |
|  |  | MANSC1 |  |
|  |  | MAP1LC3A |  |
|  |  | MAP2K3 |  |
|  |  | MAP2K5 |  |
|  |  | MAP3K10 |  |
|  |  | MAP3K11 |  |
|  |  | MAP4 |  |
|  |  | MAP6D1 |  |
|  |  | MAPK4 |  |
|  |  | GEN1 |  |
|  |  | MAPK8IP1 |  |
|  |  | MAPK8IP3 |  |
|  |  | MAPK9 |  |
|  |  | MAPKAP1 |  |
|  |  | MAPKAPK3 |  |
|  |  | MAPKBP1 |  |
|  |  | MAPRE2 |  |
|  |  | MAPRE3 |  |
|  |  | MARCH10 |  |
|  |  | MARCH4 |  |
|  |  | MARK4 |  |
|  |  | MASP2 |  |
|  |  | MATN4 |  |
|  |  | MC1R |  |
|  |  | MCCC2 |  |
|  |  | MCHR1 |  |
|  |  | MCM10 |  |
|  |  | CHRNA3 |  |
|  |  | LEFTY2 |  |
|  |  | LGALS9 |  |
|  |  | CLDN18 |  |
|  |  | CLEC4G |  |
|  |  | CLN6 |  |
|  |  | GPR179 |  |
|  |  | GPR4 |  |
|  |  | MITD1 |  |
|  |  | GPR82 |  |
|  |  | GPR98 |  |
|  |  | GPT |  |
|  |  | GPX5 |  |
|  |  | MMRN2 |  |
|  |  | LRIT2 |  |
|  |  | GSDMB |  |
|  |  | GSTA1 |  |
|  |  | GSTA3 |  |
|  |  | GSTA4 |  |
|  |  | GSTCD |  |
|  |  | GSX2 |  |
|  |  | GTF3C1 |  |
|  |  | GTF3C5 |  |
|  |  | LRWD1 |  |
|  |  | HAND2 |  |
|  |  | HAUS4 |  |
|  |  | HBA2 |  |
|  |  | HCRTR2 |  |
|  |  | MAGEA2B |  |
|  |  | HEPN1 |  |
|  |  | MVD |  |
|  |  | MAP3K15 |  |
|  |  | MYD88 |  |
|  |  | HMGCL |  |
|  |  | HMGCS2 |  |
|  |  | HMOX1 |  |
|  |  | HNRNPDL |  |
|  |  | HOXC13 |  |
|  |  | HOXD9 |  |
|  |  | HPCA |  |
|  |  | HPR |  |
|  |  | FUT7 |  |
|  |  | FXN |  |
|  |  | HS3ST2 |  |
|  |  | NCDN |  |
|  |  | HSD17B7 |  |
|  |  | HSF4 |  |
|  |  | HSFX2 |  |
|  |  | HSPA5 |  |
|  |  | HSPB8 |  |
|  |  | GAGE1 |  |
|  |  | NDUFA7 |  |
|  |  | IAH1 |  |
|  |  | GANAB |  |
|  |  | GAREML |  |
|  |  | IDH3G |  |
|  |  | IER5L |  |
|  |  | NEK11 |  |
|  |  | GCG |  |
|  |  | GCM1 |  |
|  |  | IGFBP2 |  |
|  |  | IGFBP3 |  |
|  |  | IGFBP4 |  |
|  |  | IGFN1 |  |
|  |  | IGHMBP2 |  |
|  |  | IGLON5 |  |
|  |  | GDF9 |  |
|  |  | IHH |  |
|  |  | IK |  |
|  |  | IKBKB |  |
|  |  | IKBKE |  |
|  |  | GFI1B |  |
|  |  | IL17RE |  |
|  |  | GFRA3 |  |
|  |  | GFRA4 |  |
|  |  | GGA1 |  |
|  |  | IL1B |  |
|  |  | IL1RN |  |
|  |  | IL21R |  |
|  |  | IL27RA |  |
|  |  | IL31RA |  |
|  |  | IL32 |  |
|  |  | GLP2R |  |
|  |  | INSL4 |  |
|  |  | INTS1 |  |
|  |  | GLYR1 |  |
|  |  | GMDS |  |
|  |  | GNAI2 |  |
|  |  | GNL3L |  |
|  |  | ISLR |  |
|  |  | ISLR2 |  |
|  |  | GORASP1 |  |
|  |  | GOSR2 |  |
|  |  | ITGA5 |  |
|  |  | ITGB7 |  |
|  |  | ITIH3 |  |
|  |  | ITPKA |  |
|  |  | GPD1 |  |
|  |  | GPER1 |  |
|  |  | GPIHBP1 |  |
|  |  | GPN1 |  |
|  |  | GPR153 |  |
|  |  | NUB1 |  |
|  |  | GPR25 |  |
|  |  | LDLRAD3 |  |
|  |  | NUTM2A |  |
|  |  | KCNG1 |  |
|  |  | FKBP10 |  |
|  |  | KCNK15 |  |
|  |  | FLOT2 |  |
|  |  | FMO6P |  |
|  |  | KCNT1 |  |
|  |  | KCTD13 |  |
|  |  | FOXI1 |  |
|  |  | FOXI2 |  |
|  |  | FOXK1 |  |
|  |  | FOXO4 |  |
|  |  | OSBPL5 |  |
|  |  | OTOG |  |
|  |  | OXCT2 |  |
|  |  | P4HTM |  |
|  |  | PABPC1L2B |  |
|  |  | FXYD2 |  |
|  |  | PABPN1L |  |
|  |  | LRRC3C |  |
|  |  | GAB4 |  |
|  |  | PALM3 |  |
|  |  | GALNTL6 |  |
|  |  | GAS8 |  |
|  |  | PARP2 |  |
|  |  | GATS |  |
|  |  | GBX2 |  |
|  |  | PAXIP1-AS2 |  |
|  |  | GDF2 |  |
|  |  | PCCA |  |
|  |  | GEMIN7 |  |
|  |  | GEMIN8 |  |
|  |  | GFI1 |  |
|  |  | GGCX |  |
|  |  | PCED1B |  |
|  |  | PCF11 |  |
|  |  | PCID2 |  |
|  |  | PCNT |  |
|  |  | GIGYF2 |  |
|  |  | PDE1C |  |
|  |  | PDE2A |  |
|  |  | GJB5 |  |
|  |  | GJD3 |  |
|  |  | PDE6A |  |
|  |  | PDIA4 |  |
|  |  | MYO1H |  |
|  |  | MED16 |  |
|  |  | GNAZ |  |
|  |  | GNB1L |  |
|  |  | GNG11 |  |
|  |  | GNG3 |  |
|  |  | GNG7 |  |
|  |  | PET112 |  |
|  |  | PEX11G |  |
|  |  | PEX14 |  |
|  |  | LEMD1 |  |
|  |  | LENEP |  |
|  |  | GPA33 |  |
|  |  | LGALS9B |  |
|  |  | LGALS9C |  |
|  |  | LMO2 |  |
|  |  | LOXHD1 |  |
|  |  | MMP10 |  |
|  |  | LPIN1 |  |
|  |  | FAM46D |  |
|  |  | GFER |  |
|  |  | LRRC66 |  |
|  |  | LRRC71 |  |
|  |  | LRRTM1 |  |
|  |  | LRSAM1 |  |
|  |  | MRPL28 |  |
|  |  | MRPL46 |  |
|  |  | LTA4H |  |
|  |  | LUZPP1 |  |
|  |  | GLIS1 |  |
|  |  | LYPD3 |  |
|  |  | LYSMD1 |  |
|  |  | MSTO1 |  |
|  |  | LZTS2 |  |
|  |  | MAN2B1 |  |
|  |  | MANBAL |  |
|  |  | MAP1LC3B2 |  |
|  |  | MAPK12 |  |
|  |  | MYOM2 |  |
|  |  | MARK3 |  |
|  |  | FRG1B |  |
|  |  | FRMD8 |  |
|  |  | FRMPD3 |  |
|  |  | FRS3 |  |
|  |  | FSIP2 |  |
|  |  | FTH1 |  |
|  |  | FTSJ2 |  |
|  |  | FUCA1 |  |
|  |  | FUT3 |  |
|  |  | FUT5 |  |
|  |  | NAT9 |  |
|  |  | FXYD4 |  |
|  |  | FXYD6 |  |
|  |  | FXYD7 |  |
|  |  | FYB |  |
|  |  | FZD10 |  |
|  |  | FZD2 |  |
|  |  | GABARAP |  |
|  |  | GABRA5 |  |
|  |  | GABRP |  |
|  |  | GADD45G |  |
|  |  | GADD45GIP1 |  |
|  |  | GADL1 |  |
|  |  | GAL |  |
|  |  | GALNT16 |  |
|  |  | GAS2L2 |  |
|  |  | GATA4 |  |
|  |  | GBF1 |  |
|  |  | GBP5 |  |
|  |  | GCDH |  |
|  |  | GCFC2 |  |
|  |  | GCGR |  |
|  |  | GCK |  |
|  |  | GDF5 |  |
|  |  | GDF5OS |  |
|  |  | GDPD4 |  |
|  |  | GEMIN2 |  |
|  |  | MPHOSPH8 |  |
|  |  | GFOD2 |  |
|  |  | GHDC |  |
|  |  | GHITM |  |
|  |  | GHRL |  |
|  |  | GIMAP4 |  |
|  |  | GIMAP5 |  |
|  |  | PSEN2 |  |
|  |  | GIPC3 |  |
|  |  | GIPR |  |
|  |  | GJA5 |  |
|  |  | GJB4 |  |
|  |  | GJB6 |  |
|  |  | GKAP1 |  |
|  |  | GLB1 |  |
|  |  | GLB1L2 |  |
|  |  | GLB1L3 |  |
|  |  | GLDN |  |
|  |  | GLI4 |  |
|  |  | GLIPR2 |  |
|  |  | GLIS2 |  |
|  |  | GLOD4 |  |
|  |  | GLOD5 |  |
|  |  | NOB1 |  |
|  |  | GLS2 |  |
|  |  | GLT1D1 |  |
|  |  | GLT8D1 |  |
|  |  | GLTPD1 |  |
|  |  | GLYAT |  |
|  |  | GMEB2 |  |
|  |  | GNA11 |  |
|  |  | GNB2 |  |
|  |  | GNG4 |  |
|  |  | GNGT2 |  |
|  |  | GGT1 |  |
|  |  | GOLGA1 |  |
|  |  | GOT1 |  |
|  |  | GP1BB |  |
|  |  | GPANK1 |  |
|  |  | GPATCH1 |  |
|  |  | GPATCH3 |  |
|  |  | GPATCH8 |  |
|  |  | GPBAR1 |  |
|  |  | GPC1 |  |
|  |  | NSA2 |  |
|  |  | GPR132 |  |
|  |  | GPR139 |  |
|  |  | NUP214 |  |
|  |  | METTL1 |  |
|  |  | NUTM2E |  |
|  |  | NUTM2F |  |
|  |  | PGAM5 |  |
|  |  | NXPE4 |  |
|  |  | FKBP1C |  |
|  |  | FLCN |  |
|  |  | FLJ00104 |  |
|  |  | FLJ00388 |  |
|  |  | FLJ00418 |  |
|  |  | FLNB |  |
|  |  | FLT4 |  |
|  |  | FMNL1 |  |
|  |  | OGG1 |  |
|  |  | FMOD |  |
|  |  | FNTB |  |
|  |  | OR10W1 |  |
|  |  | OR4X2 |  |
|  |  | ORAI1 |  |
|  |  | FOXQ1 |  |
|  |  | FPGS |  |
|  |  | FPR1 |  |
|  |  | IL1F10 |  |
|  |  | MMP25 |  |
|  |  | FXR2 |  |
|  |  | FXYD3 |  |
|  |  | FXYD6-FXYD2 |  |
|  |  | NGEF |  |
|  |  | MPPED1 |  |
|  |  | GABRA3 |  |
|  |  | GABRE |  |
|  |  | GABRG3 |  |
|  |  | GAK |  |
|  |  | PLEKHA5 |  |
|  |  | GALNT6 |  |
|  |  | MRPL51 |  |
|  |  | MRPL9 |  |
|  |  | GATA5 |  |
|  |  | GBGT1 |  |
|  |  | GCHFR |  |
|  |  | GEM |  |
|  |  | MTAP |  |
|  |  | POF1B |  |
|  |  | GGT2 |  |
|  |  | FGF3 |  |
|  |  | GH2 |  |
|  |  | GHRHR |  |
|  |  | GLTP |  |
|  |  | GLTSCR2 |  |
|  |  | GMPR2 |  |
|  |  | MZT1 |  |
|  |  | MEP1B |  |
|  |  | GOLGA6C |  |
|  |  | METTL3 |  |
|  |  | MFSD10 |  |
|  |  | MFSD11 |  |
|  |  | MGC4294 |  |
|  |  | IGFALS |  |
|  |  | NCOR2 |  |
|  |  | IL18R1 |  |
|  |  | NEDD8 |  |
|  |  | MMP14 |  |
|  |  | NENF |  |
|  |  | NEURL4 |  |
|  |  | PKD1L2 |  |
|  |  | MOB3A |  |
|  |  | MORN5 |  |
|  |  | NFKBIE |  |
|  |  | PLA2G2A |  |
|  |  | MRAP |  |
|  |  | PLD3 |  |
|  |  | MRPS5 |  |
|  |  | MS4A8 |  |
|  |  | PMM1 |  |
|  |  | PNOC |  |
|  |  | MTFP1 |  |
|  |  | POC1B |  |
|  |  | POLDIP2 |  |
|  |  | FNDC9 |  |
|  |  | FOXF2 |  |
|  |  | GNA15 |  |
|  |  | MYT1 |  |
|  |  | PEX16 |  |
|  |  | NAB2 |  |
|  |  | NABP2 |  |
|  |  | NANP |  |
|  |  | PPP2R5D |  |
|  |  | NBPF10 |  |
|  |  | NBPF12 |  |
|  |  | NBPF20 |  |
|  |  | NCALD |  |
|  |  | NCBP2 |  |
|  |  | PRAMEF26 |  |
|  |  | NCLN |  |
|  |  | PRKACG |  |
|  |  | PIK3R6 |  |
|  |  | PIM2 |  |
|  |  | NEK8 |  |
|  |  | PRMT5 |  |
|  |  | NELFB |  |
|  |  | PRNP |  |
|  |  | NEURL |  |
|  |  | FAM47E |  |
|  |  | NEUROG1 |  |
|  |  | FAM83C |  |
|  |  | PLA2G1B |  |
|  |  | PRRG2 |  |
|  |  | NINJ2 |  |
|  |  | PSAPL1 |  |
|  |  | NKX1-2 |  |
|  |  | NLRC3 |  |
|  |  | PLIN2 |  |
|  |  | PTGES3L |  |
|  |  | FEZF1 |  |
|  |  | PTPMT1 |  |
|  |  | PNPLA7 |  |
|  |  | PFN2 |  |
|  |  | PPP1R1B |  |
|  |  | OC90 |  |
|  |  | POLR2L |  |
|  |  | NRXN2 |  |
|  |  | FOXJ2 |  |
|  |  | PPBP |  |
|  |  | PPIF |  |
|  |  | PPM1N |  |
|  |  | PPP1R11 |  |
|  |  | PITX3 |  |
|  |  | PIWIL4 |  |
|  |  | PPP1R14B |  |
|  |  | GP2 |  |
|  |  | PLA1A |  |
|  |  | PGLYRP3 |  |
|  |  | OAS3 |  |
|  |  | OBP2A |  |
|  |  | OBP2B |  |
|  |  | OCSTAMP |  |
|  |  | OGFOD2 |  |
|  |  | OLIG2 |  |
|  |  | OPALIN |  |
|  |  | FBP2 |  |
|  |  | PRKCD |  |
|  |  | PRKCG |  |
|  |  | PIM3 |  |
|  |  | FAM228B |  |
|  |  | PNMA6C |  |
|  |  | FAM35A |  |
|  |  | FAM81B |  |
|  |  | PRR15 |  |
|  |  | PLCB2 |  |
|  |  | PLCB3 |  |
|  |  | FAT2 |  |
|  |  | FBF1 |  |
|  |  | PLEK |  |
|  |  | FBXL13 |  |
|  |  | FBXL14 |  |
|  |  | FOXD4L1 |  |
|  |  | PLVAP |  |
|  |  | PLXNB2 |  |
|  |  | FCGR3B |  |
|  |  | FOXP3 |  |
|  |  | PMPCA |  |
|  |  | PNMA5 |  |
|  |  | PNMA6A |  |
|  |  | FAM46B |  |
|  |  | PGLYRP1 |  |
|  |  | POLR2E |  |
|  |  | FLNC |  |
|  |  | PRAMEF9 |  |
|  |  | PRELID1 |  |
|  |  | PSMG2 |  |
|  |  | PRG3 |  |
|  |  | FAM3B |  |
|  |  | GOLGA8M |  |
|  |  | MID1IP1 |  |
|  |  | PPP5C |  |
|  |  | MIDN |  |
|  |  | PRB3 |  |
|  |  | PRC1 |  |
|  |  | PHOSPHO1 |  |
|  |  | PSME1 |  |
|  |  | FAM27D1 |  |
|  |  | PSTPIP2 |  |
|  |  | FBXO39 |  |
|  |  | PYDC1 |  |
|  |  | FBXO7 |  |
|  |  | PRAMEF15 |  |
|  |  | FDX1L |  |
|  |  | FAM219B |  |
|  |  | FAM3A |  |
|  |  | FASTKD1 |  |
|  |  | PRAMEF11 |  |
|  |  | PSCA |  |
|  |  | FCRLB |  |
|  |  | PRAMEF6 |  |
|  |  | FARP2 |  |
|  |  | PRAMEF4 |  |
|  |  | FAM178A |  |
|  |  | FAM178B |  |
|  |  | PRAMEF5 |  |
|  |  | STAT5A |  |
|  |  | YPEL5 |  |
|  |  | CCDC148 |  |
|  |  | H3F3A |  |
|  |  | SPACA1 |  |
|  |  | SULT2A1 |  |
|  |  | C20orf202 |  |
|  |  | C4orf3 |  |
|  |  | SLC39A12 |  |
|  |  | C1QL4 |  |
|  |  | C10orf2 |  |
|  |  | TANK |  |
|  |  | SLC5A9 |  |
|  |  | SYNE4 |  |
|  |  | ANKHD1-EIF4EBP3 | |
|  |  | C18orf8 |  |
|  |  | SOX18 |  |
|  |  | NBPF3 |  |
|  |  | SPATA31A6 |  |
|  |  | CD1E |  |
|  |  | SYCE2 |  |
|  |  | SPATA31A1 |  |
|  |  | SPATA31A3 |  |
|  |  | SSX3 |  |
|  |  | SNX32 |  |
|  |  | STAP1 |  |
|  |  | SSX2B |  |
|  |  | BTN3A3 |  |
|  |  | FERMT2 |  |
|  |  | AL139099.1 |  |
|  |  | SPI1 |  |
|  |  | SPO11 |  |
|  |  | SUSD5 |  |
|  |  | FBXW5 |  |
|  |  | SVEP1 |  |
|  |  | BCHE |  |
|  |  | ANAPC16 |  |
|  |  | SRPX |  |
|  |  | FOXA1 |  |
|  |  | FOXD4L2 |  |
|  |  | FOXD4L4 |  |
|  |  | FOXN1 |  |
|  |  | HOXD3 |  |
|  |  | TAF11 |  |
|  |  | XPO1 |  |
|  |  | PARP4 |  |
|  |  | C2orf48 |  |
|  |  | C2orf80 |  |
|  |  | RRM2B |  |
|  |  | C5orf22 |  |
|  |  | C5orf42 |  |
|  |  | SLITRK6 |  |
|  |  | SMAGP |  |
|  |  | C17orf104 |  |
|  |  | FOXD4L5 |  |
|  |  | ERCC3 |  |
|  |  | NBPF15 |  |
|  |  | ASCL4 |  |
|  |  | GJC3 |  |
|  |  | ATG4A |  |
|  |  | ATL2 |  |
|  |  | SOX10 |  |
|  |  | SPAG17 |  |
|  |  | CCT4 |  |
|  |  | C9orf135 |  |
|  |  | CD33 |  |
|  |  | SPP2 |  |
|  |  | SSX2 |  |
|  |  | BRINP3 |  |
|  |  | BTG4 |  |
|  |  | HAUS8 |  |
|  |  | HIST1H1C |  |
|  |  | C2orf61 |  |
|  |  | YPEL3 |  |
|  |  | TBC1D26 |  |
|  |  | TCHH |  |
|  |  | NAT8 |  |
|  |  | CAPRIN2 |  |
|  |  | NBPF1 |  |
|  |  | NBPF14 |  |
|  |  | GLRX2 |  |
|  |  | GMNN |  |
|  |  | AC005477.1 |  |
|  |  | GNB3 |  |
|  |  | AC026703.1 |  |
|  |  | C8orf17 |  |
|  |  | C8orf56 |  |
|  |  | GPR113 |  |
|  |  | ADAD1 |  |
|  |  | CAPN9 |  |
|  |  | PRICKLE3 |  |
|  |  | H1F0 |  |
|  |  | AIRE |  |
|  |  | AK9 |  |
|  |  | POPDC2 |  |
|  |  | ALAS2 |  |
|  |  | ALDH16A1 |  |
|  |  | ALOX15 |  |
|  |  | TOM1L1 |  |
|  |  | TOMM7 |  |
|  |  | ANAPC10 |  |
|  |  | ANKRD62 |  |
|  |  | APOC2 |  |
|  |  | APOC4-APOC2 |  |
|  |  | SMYD3 |  |
|  |  | ASIC3 |  |
|  |  | LYPD6B |  |
|  |  | EXOSC3 |  |
|  |  | ATP1A1 |  |
|  |  | ATP6V1G2 |  |
|  |  | B4GALNT4 |  |
|  |  | UPF3B |  |
|  |  | BBS5 |  |
|  |  | BGLAP |  |
|  |  | METTL2A |  |
|  |  | FBXO18 |  |
|  |  | MEX3D |  |
|  |  | MGME1 |  |
|  |  | WASF1 |  |
|  |  | C12orf74 |  |
|  |  | C14orf105 |  |
|  |  | C15orf26 |  |
|  |  | FN3KRP |  |
|  |  | MRAP2 |  |
|  |  | MRPL32 |  |
|  |  | MS4A6A |  |
|  |  | MTERFD2 |  |
|  |  | MTERFD3 |  |
|  |  | MTFR2 |  |
|  |  | MYL6 |  |
|  |  | MYOG |  |
|  |  | MYOM1 |  |
|  |  | N6AMT1 |  |
|  |  | ARL1 |  |
|  |  | NBPF24 |  |
|  |  | NDP |  |
|  |  | NDUFA1 |  |
|  |  | NEFH |  |
|  |  | NEK2 |  |
|  |  | NET1 |  |
|  |  | NEU1 |  |
|  |  | NFKBID |  |
|  |  | NKAIN3 |  |
|  |  | NLRP7 |  |
|  |  | NLRX1 |  |
|  |  | NOP58 |  |
|  |  | NOXRED1 |  |
|  |  | NQO1 |  |
|  |  | NSMCE4A |  |
|  |  | NUPR1 |  |
|  |  | OLAH |  |
|  |  | OLFM4 |  |
|  |  | FPR3 |  |
|  |  | FRAT1 |  |
|  |  | OR2C3 |  |
|  |  | OR52H1 |  |
|  |  | OR5M3 |  |
|  |  | FSCB |  |
|  |  | OXA1L |  |
|  |  | P2RX4 |  |
|  |  | PA2G4 |  |
|  |  | PAF1 |  |
|  |  | HOXC10 |  |
|  |  | GAL3ST3 |  |
|  |  | GAL3ST4 |  |
|  |  | HTATIP2 |  |
|  |  | TBR1 |  |
|  |  | TCF24 |  |
|  |  | TCHHL1 |  |
|  |  | TCP1 |  |
|  |  | N6AMT2 |  |
|  |  | FAM110B |  |
|  |  | ABCA8 |  |
|  |  | ABCB6 |  |
|  |  | AC016586.1 |  |
|  |  | AC018755.1 |  |
|  |  | ACAD10 |  |
|  |  | ACAD11 |  |
|  |  | TMEM140 |  |
|  |  | ACSM5 |  |
|  |  | ACTL7A |  |
|  |  | TMEM190 |  |
|  |  | TMEM79 |  |
|  |  | AKR7L |  |
|  |  | AL359878.1 |  |
|  |  | TNFRSF11B |  |
|  |  | ALDH1A1 |  |
|  |  | DNAJC9 |  |
|  |  | DPEP2 |  |
|  |  | DSCC1 |  |
|  |  | DSG1 |  |
|  |  | TRANK1 |  |
|  |  | ANXA1 |  |
|  |  | ANXA3 |  |
|  |  | TRMT6 |  |
|  |  | TSC22D4 |  |
|  |  | TSEN2 |  |
|  |  | TSSC1 |  |
|  |  | TTC36 |  |
|  |  | TTC6 |  |
|  |  | TTI2 |  |
|  |  | TUBA1A |  |
|  |  | UBL4A |  |
|  |  | UGT2B15 |  |
|  |  | UNC13B |  |
|  |  | UNC93A |  |
|  |  | UROS |  |
|  |  | USP48 |  |
|  |  | VSIG8 |  |
|  |  | VTCN1 |  |
|  |  | VWA7 |  |
|  |  | KDM4E |  |
|  |  | RNF17 |  |
|  |  | WDR4 |  |
|  |  | WDR61 |  |
|  |  | WDR75 |  |
|  |  | WFDC6 |  |
|  |  | WNT8A |  |
|  |  | YEATS4 |  |
|  |  | YME1L1 |  |
|  |  | C9 |  |
|  |  | CACNA2D4 |  |
|  |  | CAND2 |  |
|  |  | CASP14 |  |
|  |  | CASP6 |  |
|  |  | CCDC117 |  |
|  |  | CCDC158 |  |
|  |  | CCDC173 |  |
|  |  | CCDC8 |  |
|  |  | CCDC87 |  |
|  |  | CCL14 |  |
|  |  | ZNF28 |  |
|  |  | CD46 |  |
|  |  | CDKN1B |  |
|  |  | CENPC |  |
|  |  | CEP192 |  |
|  |  | CHCHD10 |  |
|  |  | CHGB |  |
|  |  | CHRNB3 |  |
|  |  | CHST5 |  |
|  |  | CLEC17A |  |
|  |  | CLEC1A |  |
|  |  | CLEC4E |  |
|  |  | PAIP1 |  |
|  |  | CNBD1 |  |
|  |  | CNGA4 |  |
|  |  | CNPY4 |  |
|  |  | PCDHB4 |  |
|  |  | TENM2 |  |
|  |  | CRHBP |  |
|  |  | TEX14 |  |
|  |  | CT45A5 |  |
|  |  | CT45A6 |  |
|  |  | CTC-360G5.8 |  |
|  |  | TIMM21 |  |
|  |  | CXorf24 |  |
|  |  | GOLT1A |  |
|  |  | TM4SF4 |  |
|  |  | TM7SF2 |  |
|  |  | GPN2 |  |
|  |  | TMCO4 |  |
|  |  | TMEM132C |  |
|  |  | TMEM139 |  |
|  |  | TMEM155 |  |
|  |  | GPX2 |  |
|  |  | GPX8 |  |
|  |  | TMEM211 |  |
|  |  | TMEM255A |  |
|  |  | TMEM42 |  |
|  |  | DLG1 |  |
|  |  | HADHB |  |
|  |  | TMSB15B |  |
|  |  | TNIP2 |  |
|  |  | TNN |  |
|  |  | HES7 |  |
|  |  | TOPBP1 |  |
|  |  | TOPORS |  |
|  |  | PPWD1 |  |
|  |  | PRAC1 |  |
|  |  | TRABD |  |
|  |  | HMGN1 |  |
|  |  | TREML4 |  |
|  |  | EID1 |  |
|  |  | EIF2S3L |  |
|  |  | EIF3M |  |
|  |  | EIF4EBP3 |  |
|  |  | EMX2 |  |
|  |  | ERCC6-PGBD3 |  |
|  |  | F7 |  |
|  |  | FAM150A |  |
|  |  | FAM154B |  |
|  |  | PCDHB3 |  |
|  |  | FGFBP2 |  |
|  |  | FGL1 |  |
|  |  | FIGF |  |
|  |  | FIZ1 |  |
|  |  | FNTA |  |
|  |  | FOXJ1 |  |
|  |  | FRZB |  |
|  |  | PGRMC1 |  |
|  |  | GALR1 |  |
|  |  | GATA1 |  |
|  |  | GBP3 |  |
|  |  | GBP6 |  |
|  |  | C1orf87 |  |
|  |  | C1QTNF3 |  |
|  |  | C5orf27 |  |
|  |  | C6orf57 |  |
|  |  | C6orf99 |  |
|  |  | C7orf63 |  |
|  |  | C7orf69 |  |
|  |  | CACNA1G |  |
|  |  | CCDC153 |  |
|  |  | CCDC175 |  |
|  |  | CCDC176 |  |
|  |  | CCDC51 |  |
|  |  | CCDC54 |  |
|  |  | CCDC7 |  |
|  |  | CCDC71L |  |
|  |  | CCM2 |  |
|  |  | CCRL2 |  |
|  |  | CCT6B |  |
|  |  | CDCA3 |  |
|  |  | IDI1 |  |
|  |  | IFIT1B |  |
|  |  | IFT20 |  |
|  |  | IKBIP |  |
|  |  | IL12RB1 |  |
|  |  | CROCC |  |
|  |  | CRX |  |
|  |  | CRYL1 |  |
|  |  | CTPS2 |  |
|  |  | CTSK |  |
|  |  | IZUMO3 |  |
|  |  | CYP2A6 |  |
|  |  | CYP4V2 |  |
|  |  | DAD1 |  |
|  |  | DCDC1 |  |
|  |  | DDX43 |  |
|  |  | DEFB105A |  |
|  |  | DEFB105B |  |
|  |  | DEFB107A |  |
|  |  | DEFB107B |  |
|  |  | DEFB134 |  |
|  |  | DHRS7 |  |
|  |  | DIRAS3 |  |
|  |  | KIAA1731 |  |
|  |  | DNAAF1 |  |
|  |  | DNAJB7 |  |
|  |  | DVL2 |  |
|  |  | DZANK1 |  |
|  |  | E4F1 |  |
|  |  | LGMN |  |
|  |  | LHX8 |  |
|  |  | LINC00632 |  |
|  |  | LIPA |  |
|  |  | LNP1 |  |
|  |  | LPPR1 |  |
|  |  | LRFN2 |  |
|  |  | LRRC23 |  |
|  |  | LRRC31 |  |
|  |  | LRRCC1 |  |
|  |  | LUZP4 |  |
|  |  | LYG1 |  |
|  |  | MAGEA11 |  |
|  |  | MAGEF1 |  |
|  |  | MAGEL2 |  |
|  |  | MAP4K5 |  |
|  |  | MCM3 |  |
|  |  | MED12 |  |
|  |  | MEP1A |  |
|  |  | SLC17A4 |  |
|  |  | MIS12 |  |
|  |  | MNAT1 |  |
|  |  | SLC36A3 |  |
|  |  | SLC39A8 |  |
|  |  | SLC46A3 |  |
|  |  | ZCRB1 |  |
|  |  | ZIM3 |  |
|  |  | ZNF107 |  |
|  |  | FAM21A |  |
|  |  | FAM21B |  |
|  |  | FAM21C |  |
|  |  | FBXL22 |  |
|  |  | FCGR2A |  |
|  |  | ZNF506 |  |
|  |  | ZNF598 |  |
|  |  | FLJ30594 |  |
|  |  | GABARAPL3 |  |
|  |  | CNDP2 |  |
|  |  | GALK2 |  |
|  |  | GEMIN4 |  |
|  |  | PDE1A |  |
|  |  | PDLIM7 |  |
|  |  | PDZD9 |  |
|  |  | PDZRN4 |  |
|  |  | PGBD3 |  |
|  |  | PGBD5 |  |
|  |  | PHF5A |  |
|  |  | PIAS3 |  |
|  |  | PIGF |  |
|  |  | PIR |  |
|  |  | PLAGL1 |  |
|  |  | PMF1-BGLAP |  |
|  |  | GUCA1C |  |
|  |  | POU5F1 |  |
|  |  | HEATR2 |  |
|  |  | PPP3R2 |  |
|  |  | HINT1 |  |
|  |  | PRKCDBP |  |
|  |  | PRKRA |  |
|  |  | PRPF19 |  |
|  |  | PRSS35 |  |
|  |  | PRSS36 |  |
|  |  | PRSS37 |  |
|  |  | PSMD1 |  |
|  |  | PXMP2 |  |
|  |  | RAB38 |  |
|  |  | RAB42 |  |
|  |  | RAD54L |  |
|  |  | RANBP17 |  |
|  |  | IRX5 |  |
|  |  | RBM26 |  |
|  |  | RBM44 |  |
|  |  | RBPJL |  |
|  |  | RCN3 |  |
|  |  | REC8 |  |
|  |  | RFX8 |  |
|  |  | RGS7 |  |
|  |  | RLN3 |  |
|  |  | RNF121 |  |
|  |  | RP11-181C3.1 |  |
|  |  | RP11-215A19.2 |  |
|  |  | RP11-701P16.2 |  |
|  |  | RP11-849H4.2 |  |
|  |  | RPL17-C18orf32 |  |
|  |  | RPL26L1 |  |
|  |  | RPS3 |  |
|  |  | RPSAP58 |  |
|  |  | ZBTB12 |  |
|  |  | C12orf54 |  |
|  |  | KRT12 |  |
|  |  | KRT17 |  |
|  |  | KRTAP3-2 |  |
|  |  | KRTAP8-1 |  |
|  |  | L3MBTL1 |  |
|  |  | ZC3HC1 |  |
|  |  | ZDHHC11B |  |
|  |  | C19orf67 |  |
|  |  | ZNF19 |  |
|  |  | ZNF263 |  |
|  |  | ZNF273 |  |
|  |  | ZNF337 |  |
|  |  | ZNF385A |  |
|  |  | ZNF408 |  |
|  |  | ZNF587B |  |
|  |  | ZNF614 |  |
|  |  | ZNF669 |  |
|  |  | ZNF781 |  |
|  |  | ZNF793 |  |
|  |  | ZSCAN18 |  |
|  |  | ZZZ3 |  |
|  |  | MCF2 |  |
|  |  | CLIP3 |  |
|  |  | COL6A5 |  |
|  |  | CRADD |  |
|  |  | AADAT |  |
|  |  | ABCA4 |  |
|  |  | ABHD14B |  |
|  |  | AC023590.1 |  |
|  |  | AC120194.1 |  |
|  |  | AGXT |  |
|  |  | NBAS |  |
|  |  | AKIRIN2 |  |
|  |  | TCP11 |  |
|  |  | TDRD10 |  |
|  |  | TEX12 |  |
|  |  | ANK1 |  |
|  |  | AP001885.1 |  |
|  |  | APBB3 |  |
|  |  | APOB |  |
|  |  | ARMCX1 |  |
|  |  | OBSCN |  |
|  |  | ATN1 |  |
|  |  | ATP5L |  |
|  |  | ATP6V0D2 |  |
|  |  | AVIL |  |
|  |  | BLVRB |  |
|  |  | BUD13 |  |
|  |  | C10orf112 |  |
|  |  | C15orf37 |  |
|  |  | C15orf43 |  |
|  |  | C17orf50 |  |
|  |  | LDB3 |  |
|  |  | POLE4 |  |
|  |  | POLR3B |  |
|  |  | PRAMEF13 |  |
|  |  | PRAMEF14 |  |
|  |  | PROM1 |  |
|  |  | PSMA2 |  |
|  |  | PSMA3 |  |
|  |  | CDKL4 |  |
|  |  | MESP2 |  |
|  |  | CENPN |  |
|  |  | CGREF1 |  |
|  |  | IQCH |  |
|  |  | IRG1 |  |
|  |  | ISG15 |  |
|  |  | CLEC5A |  |
|  |  | JRKL |  |
|  |  | KATNAL2 |  |
|  |  | KCNV2 |  |
|  |  | KIAA1239 |  |
|  |  | CPT2 |  |
|  |  | KLK13 |  |
|  |  | KRT81 |  |
|  |  | LACC1 |  |
|  |  | LARP6 |  |
|  |  | LCK |  |
|  |  | CYP4X1 |  |
|  |  | DAGLB |  |
|  |  | DCAF4L2 |  |
|  |  | DDX27 |  |
|  |  | DDX60 |  |
|  |  | DEPDC7 |  |
|  |  | DHRS13 |  |
|  |  | DHX9 |  |
|  |  | SEL1L3 |  |
|  |  | DNAJC19 |  |
|  |  | DNAJC5B |  |
|  |  | DNMT1 |  |
|  |  | DOC2A |  |
|  |  | DRC1 |  |
|  |  | DSG4 |  |
|  |  | DYDC1 |  |
|  |  | DYRK4 |  |
|  |  | ECM2 |  |
|  |  | ECSIT |  |
|  |  | ELMO3 |  |
|  |  | KRT25 |  |
|  |  | KRT35 |  |
|  |  | KRTAP22-2 |  |
|  |  | LAMB1 |  |
|  |  | SEC61B |  |
|  |  | LMBR1L |  |
|  |  | LMOD2 |  |
|  |  | LRP5L |  |
|  |  | LRRC26 |  |
|  |  | LRRC70 |  |
|  |  | LRRC8D |  |
|  |  | LSR |  |
|  |  | LY6D |  |
|  |  | LY6G6E |  |
|  |  | SLC14A2 |  |
|  |  | LZTR1 |  |
|  |  | MAP2K1 |  |
|  |  | MARCH11 |  |
|  |  | SMCO4 |  |
|  |  | GLYATL3 |  |
|  |  | GNG5 |  |
|  |  | GPR143 |  |
|  |  | GPR150 |  |
|  |  | GSAP |  |
|  |  | GTF2H3 |  |
|  |  | H2AFY2 |  |
|  |  | HCST |  |
|  |  | HDDC3 |  |
|  |  | HIST1H3G |  |
|  |  | NFKBIL1 |  |
|  |  | NFX1 |  |
|  |  | NFYC |  |
|  |  | NKX6-2 |  |
|  |  | HUS1 |  |
|  |  | HYAL2 |  |
|  |  | IFITM3 |  |
|  |  | IFNA10 |  |
|  |  | IFNA16 |  |
|  |  | IFNA17 |  |
|  |  | IFNA2 |  |
|  |  | IFNA4 |  |
|  |  | IFNA6 |  |
|  |  | IFT140 |  |
|  |  | IL1R2 |  |
|  |  | IL22RA1 |  |
|  |  | IL22RA2 |  |
|  |  | INPP5E |  |
|  |  | JSRP1 |  |
|  |  | JUP |  |
|  |  | KB-1980E6.3 |  |
|  |  | KCNE2 |  |
|  |  | KCNMA1 |  |
|  |  | KDELR3 |  |
|  |  | KIAA0754 |  |
|  |  | ZNF577 |  |
|  |  | PFKFB1 |  |
|  |  | UTP11L |  |
|  |  | RSU1 |  |
|  |  | PHF11 |  |
|  |  | RTTN |  |
|  |  | SARS2 |  |
|  |  | SEMA5B |  |
|  |  | MTFMT |  |
|  |  | SLC13A2 |  |
|  |  | ZNF133 |  |
|  |  | SLC25A43 |  |
|  |  | ZNF354A |  |
|  |  | ZNF44 |  |
|  |  | RPL10L |  |
|  |  | ZNF7 |  |
|  |  | SMPDL3A |  |
|  |  | MKRN1 |  |
|  |  | MNDA |  |
|  |  | MRPL39 |  |
|  |  | MS4A7 |  |
|  |  | MSANTD1 |  |
|  |  | MUC16 |  |
|  |  | MYEOV2 |  |
|  |  | MYH15 |  |
|  |  | MYL9 |  |
|  |  | NBPF16 |  |
|  |  | RP11-219B4.5 |  |
|  |  | NOL11 |  |
|  |  | CHRNA5 |  |
|  |  | OAS1 |  |
|  |  | OR10H5 |  |
|  |  | SERPINA9 |  |
|  |  | OSGEP |  |
|  |  | SF3B14 |  |
|  |  | P2RX1 |  |
|  |  | TRPM5 |  |
|  |  | PCGF6 |  |
|  |  | PCSK1N |  |
|  |  | USP4 |  |
|  |  | VAMP7 |  |
|  |  | SCCPDH |  |
|  |  | SEPT2 |  |
|  |  | SFR1 |  |
|  |  | SFRP4 |  |
|  |  | SFTA3 |  |
|  |  | RIT2 |  |
|  |  | SLC26A3 |  |
|  |  | SLC27A5 |  |
|  |  | SLC2A13 |  |
|  |  | TBCE |  |
|  |  | RP11-171N4.2 |  |
|  |  | SMCO1 |  |
|  |  | RTN3 |  |
|  |  | SAT1 |  |
|  |  | SNX7 |  |
|  |  | RAB41 |  |
|  |  | RBM11 |  |
|  |  | REG4 |  |
|  |  | RELL1 |  |
|  |  | RGS21 |  |
|  |  | SIRT6 |  |
|  |  | TAF12 |  |
|  |  | RNF183 |  |
|  |  | CRYGB |  |
|  |  | CTHRC1 |  |
|  |  | RP11-706O15.1 |  |
|  |  | RUFY1 |  |
|  |  | SCARA5 |  |
|  |  | SCGB3A2 |  |
|  |  | SCN11A |  |
|  |  | SDHD |  |
|  |  | SEMA7A |  |
|  |  | SERPINF1 |  |
|  |  | SFXN3 |  |
|  |  | CYLC2 |  |
|  |  | CPB2 |  |
|  |  | TUBB3 |  |
|  |  | CSNK2A2 |  |
|  |  | SSX1 |  |
|  |  | VIM |  |
|  |  | VMO1 |  |
|  |  | CLCF1 |  |
|  |  | SSX5 |  |
|  |  | TBCC |  |
|  |  | TBL1Y |  |
|  |  | SUPT20H |  |
|  |  | COL22A1 |  |
|  |  | SYTL3 |  |
|  |  | TAC4 |  |
|  |  | COL14A1 |  |
|  |  | TMEM125 |  |
|  |  | AL031663.2 |  |
|  |  | ARPC1A |  |
|  |  | C1orf85 |  |
|  |  | CD5 |  |
|  |  | CLN3 |  |
|  |  | AC135983.2 |  |
|  |  | AC016559.1 |  |
|  |  | CEACAM7 |  |
|  |  | AC006372.1 |  |
|  |  | AC069547.1 |  |
|  |  | AC009365.3 |  |
|  |  | AES |  |
|  |  | IL36G |  |
|  |  | CENPBD1 |  |
|  |  | NBPF6 |  |
|  |  | MICALL2 |  |
|  |  | AP000322.53 |  |
|  |  | C1orf94 |  |
|  |  | CLLU1OS |  |
|  |  | AC004076.7 |  |
|  |  | ODAM |  |
|  |  | ACO2 |  |
|  |  | CARD16 |  |
|  |  | CCDC74A |  |
|  |  | ARHGEF26 |  |
|  |  | ADAMTSL4 |  |
|  |  | PSG2 |  |
|  |  | PSG9 |  |
|  |  | ARMCX2 |  |
|  |  | AC000003.2 |  |
|  |  | ASS1 |  |
|  |  | AGR3 |  |
|  |  | ARMCX4 |  |
|  |  | AIFM3 |  |
|  |  | NDRG2 |  |
|  |  | HEPACAM2 |  |
|  |  | HES1 |  |
|  |  | HPGDS |  |
|  |  | CETN1 |  |
|  |  | NR0B2 |  |
|  |  | AQP5 |  |
|  |  | CIDEA |  |
|  |  | MRFAP1L1 |  |
|  |  | MRPL11 |  |
|  |  | MSMO1 |  |
|  |  | MSRB2 |  |
|  |  | PRPF39 |  |
|  |  | ACOT4 |  |
|  |  | ACSM1 |  |
|  |  | CCDC12 |  |
|  |  | ADCY10 |  |
|  |  | PRR20D |  |
|  |  | PRR20E |  |
|  |  | PRR5 |  |
|  |  | LTK |  |
|  |  | PRSS57 |  |
|  |  | CCL3L3 |  |
|  |  | AHSP |  |
|  |  | HEXB |  |
|  |  | NEK5 |  |
|  |  | CEACAM5 |  |
|  |  | NLRP12 |  |
|  |  | CELF5 |  |
|  |  | NOL8 |  |
|  |  | NPC1 |  |
|  |  | NPEPL1 |  |
|  |  | NPFFR2 |  |
|  |  | NPIPA5 |  |
|  |  | NPPC |  |
|  |  | NUDT18 |  |
|  |  | MRI1 |  |
|  |  | MRPL54 |  |
|  |  | MRPS17 |  |
|  |  | OMD |  |
|  |  | OR4A5 |  |
|  |  | ORC6 |  |
|  |  | OVGP1 |  |
|  |  | ATRAID |  |
|  |  | ATXN2 |  |
|  |  | ADAM33 |  |
|  |  | PRR16 |  |
|  |  | PRR20A |  |
|  |  | PRR20B |  |
|  |  | PRR20C |  |
|  |  | BBS12 |  |
|  |  | NAT1 |  |
|  |  | BLM |  |
|  |  | NCCRP1 |  |
|  |  | NDUFAB1 |  |
|  |  | C10orf40 |  |
|  |  | C11orf88 |  |
|  |  | NHLH2 |  |
|  |  | NKG2-E |  |
|  |  | NKX2-4 |  |
|  |  | NME8 |  |
|  |  | NMU |  |
|  |  | C21orf62 |  |
|  |  | NPIPA7 |  |
|  |  | C21orf88 |  |
|  |  | C2orf50 |  |
|  |  | C3orf27 |  |
|  |  | C5orf34 |  |
|  |  | C7orf34 |  |
|  |  | C9orf117 |  |
|  |  | DNAL4 |  |
|  |  | C9orf57 |  |
|  |  | DOCK2 |  |
|  |  | CA1 |  |
|  |  | CA10 |  |
|  |  | CA5A |  |
|  |  | CALHM3 |  |
|  |  | PAMR1 |  |
|  |  | CASC5 |  |
|  |  | CASP8 |  |
|  |  | CASQ2 |  |
|  |  | CATSPER4 |  |
|  |  | CCDC104 |  |
|  |  | PKD2L2 |  |
|  |  | CCDC181 |  |
|  |  | FLII |  |
|  |  | CCDC74B |  |
|  |  | CCDC77 |  |
|  |  | CCKBR |  |
|  |  | FOLR2 |  |
|  |  | CCT2 |  |
|  |  | CCT3 |  |
|  |  | FOXR2 |  |
|  |  | CD68 |  |
|  |  | CDH26 |  |
|  |  | CDK4 |  |
|  |  | CDY2A |  |
|  |  | PLA2G4F |  |
|  |  | PLA2G6 |  |
|  |  | GNB2L1 |  |
|  |  | CNGB1 |  |
|  |  | MT-ATP8 |  |
|  |  | MX1 |  |
|  |  | MYBPHL |  |
|  |  | MYCBP2 |  |
|  |  | HAL |  |
|  |  | HAPLN2 |  |
|  |  | HARBI1 |  |
|  |  | HAX1 |  |
|  |  | HDAC6 |  |
|  |  | HHEX |  |
|  |  | HIBADH |  |
|  |  | HIGD2A |  |
|  |  | HIST1H2AH |  |
|  |  | HIST1H2AI |  |
|  |  | HIST1H2AK |  |
|  |  | HIST1H4C |  |
|  |  | HIST1H4E |  |
|  |  | RBMY1B |  |
|  |  | RFC4 |  |
|  |  | PPID |  |
|  |  | hsa-miR-1199 |  |
|  |  | HSPB6 |  |
|  |  | HTATSF1 |  |
|  |  | HTR1E |  |
|  |  | HTR5A-AS1 |  |
|  |  | IFIH1 |  |
|  |  | IGSF1 |  |
|  |  | IGSF21 |  |
|  |  | IL12B |  |
|  |  | IL17A |  |
|  |  | IL23A |  |
|  |  | IL26 |  |
|  |  | IL31 |  |
|  |  | IL5 |  |
|  |  | OLR1 |  |
|  |  | PHKG1 |  |
|  |  | OR10H1 |  |
|  |  | OR2D2 |  |
|  |  | OR2H1 |  |
|  |  | OR51Q1 |  |
|  |  | ORAI3 |  |
|  |  | IQCJ-SCHIP1 |  |
|  |  | JTB |  |
|  |  | KANSL2 |  |
|  |  | KBTBD4 |  |
|  |  | BLOC1S4 |  |
|  |  | PHYH |  |
|  |  | BRAF |  |
|  |  | BUB1B |  |
|  |  | C16orf47 |  |
|  |  | C17orf74 |  |
|  |  | C19orf68 |  |
|  |  | C1orf162 |  |
|  |  | C1R |  |
|  |  | PSMD8 |  |
|  |  | C21orf49 |  |
|  |  | C2CD4B |  |
|  |  | PIPOX |  |
|  |  | FAM177B |  |
|  |  | FAM209A |  |
|  |  | FAM209B |  |
|  |  | PITRM1 |  |
|  |  | C8orf74 |  |
|  |  | C9orf131 |  |
|  |  | LIPH |  |
|  |  | LPA |  |
|  |  | LRFN3 |  |
|  |  | LRFN4 |  |
|  |  | LRP11 |  |
|  |  | LRRC10B |  |
|  |  | LRRC18 |  |
|  |  | LSM4 |  |
|  |  | LSMD1 |  |
|  |  | LYPD8 |  |
|  |  | LYZL6 |  |
|  |  | MACF1 |  |
|  |  | MAEL |  |
|  |  | MAL2 |  |
|  |  | PFN4 |  |
|  |  | MCCD1 |  |
|  |  | MDK |  |
|  |  | ME3 |  |
|  |  | MEOX2 |  |
|  |  | MGARP |  |
|  |  | PRL |  |
|  |  | PRLHR |  |
|  |  | MKL1 |  |
|  |  | MOG |  |
|  |  | OR2A7 |  |
|  |  | OR6C74 |  |
|  |  | PASK |  |
|  |  | PATE1 |  |
|  |  | PBK |  |
|  |  | PCDH12 |  |
|  |  | PCDH15 |  |
|  |  | PCDHB11 |  |
|  |  | PCIF1 |  |
|  |  | PDCD1LG2 |  |
|  |  | PDCD7 |  |
|  |  | PFDN2 |  |
|  |  | PI4KB |  |
|  |  | PLA2G4D |  |
|  |  | PNKD |  |
|  |  | PPIL3 |  |
|  |  | PPM1J |  |
|  |  | PRKDC |  |
|  |  | PRMT8 |  |
|  |  | PRRT1 |  |
|  |  | PRSS33 |  |
|  |  | PSMA6 |  |
|  |  | PTPRCAP |  |
|  |  | RACGAP1 |  |
|  |  | RAD17 |  |
|  |  | RAG2 |  |
|  |  | RECQL |  |
|  |  | REEP6 |  |
|  |  | REP15 |  |
|  |  | RFXANK |  |
|  |  | RGCC |  |
|  |  | RHOXF1 |  |
|  |  | RINL |  |
|  |  | RNF133 |  |
|  |  | RNF39 |  |
|  |  | RNH1 |  |
|  |  | RP11-160N1.10 |  |
|  |  | RP11-386G21.2 |  |
|  |  | RP11-389E17.1 |  |
|  |  | RP11-597K23.2 |  |
|  |  | RPL23A |  |
|  |  | RPL35 |  |
|  |  | RPSA |  |
|  |  | RUFY4 |  |
|  |  | S100A6 |  |
|  |  | SAAL1 |  |
|  |  | SAMD7 |  |
|  |  | SAP30BP |  |
|  |  | SCAND1 |  |
|  |  | SCGB1D4 |  |
|  |  | SCHIP1 |  |
|  |  | SERAC1 |  |
|  |  | SETD4 |  |
|  |  | SFT2D1 |  |
|  |  | SH3D21 |  |
|  |  | CNPY2 |  |
|  |  | COL3A1 |  |
|  |  | COPZ2 |  |
|  |  | COQ6 |  |
|  |  | CPNE6 |  |
|  |  | CRCT1 |  |
|  |  | CREB3 |  |
|  |  | CSF2 |  |
|  |  | CYP4F31P |  |
|  |  | CYP4Z1 |  |
|  |  | DCAF8L1 |  |
|  |  | DCLK3 |  |
|  |  | DDAH2 |  |
|  |  | DECR1 |  |
|  |  | DEFB127 |  |
|  |  | DHODH |  |
|  |  | DIS3L |  |
|  |  | DPH5 |  |
|  |  | DQX1 |  |
|  |  | DSCAML1 |  |
|  |  | DUOXA1 |  |
|  |  | DUSP21 |  |
|  |  | DUSP27 |  |
|  |  | DYDC2 |  |
|  |  | DYNLRB2 |  |
|  |  | DYNLT3 |  |
|  |  | EBNA1BP2 |  |
|  |  | ELAC1 |  |
|  |  | ELOF1 |  |
|  |  | ELOVL3 |  |
|  |  | EML2 |  |
|  |  | EMR1 |  |
|  |  | ENAM |  |
|  |  | PMS1 |  |
|  |  | ETFB |  |
|  |  | EVC2 |  |
|  |  | FABP9 |  |
|  |  | TMEM249 |  |
|  |  | FAM43A |  |
|  |  | FAM96B |  |
|  |  | FANCD2OS |  |
|  |  | FBRS |  |
|  |  | FBXO43 |  |
|  |  | FBXO46 |  |
|  |  | FCGR2B |  |
|  |  | FCRL5 |  |
|  |  | FGD2 |  |
|  |  | FIBP |  |
|  |  | PNMAL1 |  |
|  |  | FLAD1 |  |
|  |  | FOXD4L3 |  |
|  |  | FOXD4L6 |  |
|  |  | FPGT-TNNI3K |  |
|  |  | FTL |  |
|  |  | G0S2 |  |
|  |  | POMK |  |
|  |  | GIPC1 |  |
|  |  | GLE1 |  |
|  |  | POU1F1 |  |
|  |  | PPA2 |  |
|  |  | GRM2 |  |
|  |  | QRFPR |  |
|  |  | R3HDML |  |
|  |  | RAD51AP1 |  |
|  |  | RASIP1 |  |
|  |  | RBM12B-AS1 |  |
|  |  | RBMY1A1 |  |
|  |  | RBMY1D |  |
|  |  | RBMY1E |  |
|  |  | RBMY1F |  |
|  |  | RBMY1J |  |
|  |  | REG3A |  |
|  |  | RGN |  |
|  |  | RIOK1 |  |
|  |  | RNASEK-C17orf49 | |
|  |  | RP11-10J21.3 |  |
|  |  | RP11-162A12.2 |  |
|  |  | INSL3 |  |
|  |  | IRF9 |  |
|  |  | PPP1R14D |  |
|  |  | KCNJ4 |  |
|  |  | KCNS3 |  |
|  |  | KHDRBS1 |  |
|  |  | KHK |  |
|  |  | KIAA0226L |  |
|  |  | KIFAP3 |  |
|  |  | KLK15 |  |
|  |  | KLK7 |  |
|  |  | KRT2 |  |
|  |  | KRT4 |  |
|  |  | KRT6B |  |
|  |  | KRT9 |  |
|  |  | KRTAP5-5 |  |
|  |  | KRTAP9-6 |  |
|  |  | LAMB4 |  |
|  |  | LELP1 |  |
|  |  | LGALS1 |  |
|  |  | LGI1 |  |
|  |  | LGR5 |  |
|  |  | SLC18A3 |  |
|  |  | SLC27A6 |  |
|  |  | SLC35D3 |  |
|  |  | SLC35G2 |  |
|  |  | SLC36A2 |  |
|  |  | SLC4A9 |  |
|  |  | SMPD4 |  |
|  |  | SNAI2 |  |
|  |  | SNRPF |  |
|  |  | SOD1 |  |
|  |  | SPATA31E1 |  |
|  |  | SPICE1 |  |
|  |  | SPINK13 |  |
|  |  | SPINK7 |  |
|  |  | SRA1 |  |
|  |  | SSX4 |  |
|  |  | SSX4B |  |
|  |  | SSX7 |  |
|  |  | ST7-OT4 |  |
|  |  | STEAP1B |  |
|  |  | STMN4 |  |
|  |  | STX10 |  |
|  |  | TARSL2 |  |
|  |  | TBC1D10A |  |
|  |  | TFPT |  |
|  |  | TJP2 |  |
|  |  | TMA16 |  |
|  |  | TMEM138 |  |
|  |  | TMPRSS11E |  |
|  |  | TNNI3K |  |
|  |  | TOMM5 |  |
|  |  | TP53 |  |
|  |  | TRAP1 |  |
|  |  | TRIM3 |  |
|  |  | TRIM31 |  |
|  |  | TRIM5 |  |
|  |  | TRNAU1AP |  |
|  |  | TRUB2 |  |
|  |  | TTLL10 |  |
|  |  | TTR |  |
|  |  | TYK2 |  |
|  |  | UBA3 |  |
|  |  | UBTF |  |
|  |  | UBXN1 |  |
|  |  | UBXN11 |  |
|  |  | UCN |  |
|  |  | ULBP3 |  |
|  |  | UQCRC2 |  |
|  |  | USP16 |  |
|  |  | USP29 |  |
|  |  | VSX1 |  |
|  |  | WBSCR27 |  |
|  |  | WDPCP |  |
|  |  | WDR64 |  |
|  |  | WFDC11 |  |
|  |  | ZBBX |  |
|  |  | ZCCHC5 |  |
|  |  | ZNF132 |  |
|  |  | ZNF30 |  |
|  |  | ZNF32 |  |
|  |  | ZNF487 |  |
|  |  | ZNF565 |  |
|  |  | ZNF586 |  |
|  |  | ZNF665 |  |
|  |  | ZNF680 |  |
|  |  | ZNF774 |  |
|  |  | ZNF829 |  |
|  |  | ZSCAN16 |  |
|  |  | ZSWIM8 |  |
|  |  | SIRPD |  |
|  |  | SIRT4 |  |
|  |  | SLC16A13 |  |
|  |  | SLC43A3 |  |
|  |  | SMCO2 |  |
|  |  | SMIM22 |  |
|  |  | SOWAHD |  |
|  |  | SPRY2 |  |
|  |  | ST7 |  |
|  |  | STRA6 |  |
|  |  | SUN3 |  |
|  |  | TCEAL7 |  |
|  |  | TDRD5 |  |
|  |  | TECR |  |
|  |  | TEX38 |  |
|  |  | TOP3B |  |
|  |  | TRIM55 |  |
|  |  | TRIML1 |  |
|  |  | PCK2 |  |
|  |  | TTC23 |  |
|  |  | TTC9C |  |
|  |  | TUBA1B |  |
|  |  | UGT2B10 |  |
|  |  | UGT2B17 |  |
|  |  | VAX2 |  |
|  |  | VDAC1 |  |
|  |  | VIT |  |
|  |  | VSIG1 |  |
|  |  | PDC |  |
|  |  | RP11-324D17.1 |  |
|  |  | RP11-332O19.5 |  |
|  |  | RP11-379H8.1 |  |
|  |  | RPL32 |  |
|  |  | RPS20 |  |
|  |  | RPS3A |  |
|  |  | RYR1 |  |
|  |  | SAMD13 |  |
|  |  | SBSN |  |
|  |  | SCG5 |  |
|  |  | SERPINA10 |  |
|  |  | SERPINE2 |  |
|  |  | SH3GLB2 |  |
|  |  | SHBG |  |
|  |  | SHFM1 |  |
|  |  | SHMT2 |  |
|  |  | SLC12A8 |  |
|  |  | AC007431.1 |  |
|  |  | AC117834.1 |  |
|  |  | ACAN |  |
|  |  | ACOT12 |  |
|  |  | ACTG1 |  |
|  |  | ADAM30 |  |
|  |  | AL356356.1 |  |
|  |  | AL391152.1 |  |
|  |  | ANKRD61 |  |
|  |  | ANO2 |  |
|  |  | AP1S1 |  |
|  |  | ARHGEF1 |  |
|  |  | ARHGEF40 |  |
|  |  | ATP2A1 |  |
|  |  | ATP4B |  |
|  |  | AXDND1 |  |
|  |  | BARX2 |  |
|  |  | BATF |  |
|  |  | BRINP2 |  |
|  |  | C12orf10 |  |
|  |  | C12orf45 |  |
|  |  | C20orf173 |  |
|  |  | C2orf70 |  |
|  |  | C5orf54 |  |
|  |  | C8orf48 |  |
|  |  | C9orf173 |  |
|  |  | CACNG3 |  |
|  |  | CALML6 |  |
|  |  | CASP12 |  |
|  |  | CCDC102A |  |
|  |  | CCDC169 |  |
|  |  | CCDC28B |  |
|  |  | CCDC66 |  |
|  |  | CCDC91 |  |
|  |  | CD79B |  |
|  |  | CDKL3 |  |
|  |  | CDRT1 |  |
|  |  | CENPE |  |
|  |  | CLDN7 |  |
|  |  | CLEC3A |  |
|  |  | CLP1 |  |
|  |  | CLRN3 |  |
|  |  | CLUL1 |  |
|  |  | CNTRL |  |
|  |  | COL4A2 |  |
|  |  | CRELD2 |  |
|  |  | CSN2 |  |
|  |  | CTA-299D3.8 |  |
|  |  | CTD-2116N17.1 |  |
|  |  | CTSH |  |
|  |  | CTSZ |  |
|  |  | CUTC |  |
|  |  | CXCL17 |  |
|  |  | CXCR3 |  |
|  |  | CYP4B1 |  |
|  |  | DENND1B |  |
|  |  | DENND4A |  |
|  |  | DOCK1 |  |
|  |  | DPCR1 |  |
|  |  | EEF1E1 |  |
|  |  | EIF2A |  |
|  |  | ELF5 |  |
|  |  | ENO3 |  |
|  |  | EPN1 |  |
|  |  | EPS8L1 |  |
|  |  | ERAS |  |
|  |  | ERP27 |  |
|  |  | EVA1B |  |
|  |  | F11 |  |
|  |  | FAM187A |  |
|  |  | FAM20C |  |
|  |  | FAM65A |  |
|  |  | FAM69C |  |
|  |  | FCER1G |  |
|  |  | FCRLA |  |
|  |  | FGD1 |  |
|  |  | FGF18 |  |
|  |  | FKBP3 |  |
|  |  | FLJ14816 |  |
|  |  | FNDC4 |  |
|  |  | FOXC2 |  |
|  |  | FOXS1 |  |
|  |  | FSHB |  |
|  |  | GBP1 |  |
|  |  | GCAT |  |
|  |  | GDPD2 |  |
|  |  | GGT7 |  |
|  |  | GP5 |  |
|  |  | GPR1 |  |
|  |  | GPRIN1 |  |
|  |  | GSTO1 |  |
|  |  | GTF3A |  |
|  |  | GUCY2C |  |
|  |  | HMX3 |  |
|  |  | HOMER2 |  |
|  |  | ICT1 |  |
|  |  | IFNGR1 |  |
|  |  | IGLL1 |  |
|  |  | IL6 |  |
|  |  | IMP3 |  |
|  |  | IQCF6 |  |
|  |  | ISM1 |  |
|  |  | KCTD1 |  |
|  |  | KEAP1 |  |
|  |  | KIAA1211L |  |
|  |  | KLF1 |  |
|  |  | KRCC1 |  |
|  |  | KRT31 |  |
|  |  | KRTAP1-3 |  |
|  |  | KRTAP13-1 |  |
|  |  | KRTAP21-2 |  |
|  |  | LAMA3 |  |
|  |  | LEPRE1 |  |
|  |  | LGALS12 |  |
|  |  | LMTK3 |  |
|  |  | LRIT1 |  |
|  |  | LRRC17 |  |
|  |  | LRRC8E |  |
|  |  | LTB4R |  |
|  |  | MANBA |  |
|  |  | MARVELD3 |  |
|  |  | MBIP |  |
|  |  | MCEE |  |
|  |  | METTL17 |  |
|  |  | MRFAP1 |  |
|  |  | MT1G |  |
|  |  | MYH11 |  |
|  |  | MYO19 |  |
|  |  | MYO7B |  |
|  |  | NBPF8 |  |
|  |  | NDUFA6 |  |
|  |  | NEXN |  |
|  |  | NOP2 |  |
|  |  | NRTN |  |
|  |  | NXF3 |  |
|  |  | NXF5 |  |
